# Leveraging Electron‐Deficient Iminium Intermediates in a General Synthesis of Valuable Amines

**DOI:** 10.1002/anie.202115435

**Published:** 2022-03-16

**Authors:** Che‐Sheng Hsu, Carlos R. Gonçalves, Veronica Tona, Amandine Pons, Marcel Kaiser, Nuno Maulide

**Affiliations:** ^1^ University of Vienna Institute of Organic Chemistry Währinger Strasse 38 1090 Vienna Austria; ^2^ Swiss Tropical and Public Health Institute Socinstrasse 57 4002 Basel Switzerland

**Keywords:** Acid-Mediated Reaction, Amines, Hydroaminoalkylation, Iminium Ions, Synthetic Methods

## Abstract

The development of reactions converting alkenes and alkynes into valuable building blocks remains one of the main goals of synthetic chemistry. Herein, we present the leveraging of highly electron‐deficient iminium ions, rare and fleeting intermediates, into a general amine synthesis. This enables the preparation of amines bearing e.g. valuable α‐trifluoromethyl moieties under mild conditions. This broad concept is highlighted by the late‐stage amination of quinine into a biologically interesting new analogue.

## Graphical abstract

The use of aminals as precursors for iminium ions has been limited to methylene derivatives, thus hampering its broader use. By employing electron‐deficient iminium ions an obvious hurdle of the methodology is overcome, whilst generating highly desirable motifs. This broad concept is highlighted by the late‐stage amination of quinine into a biologically interesting new analogue.

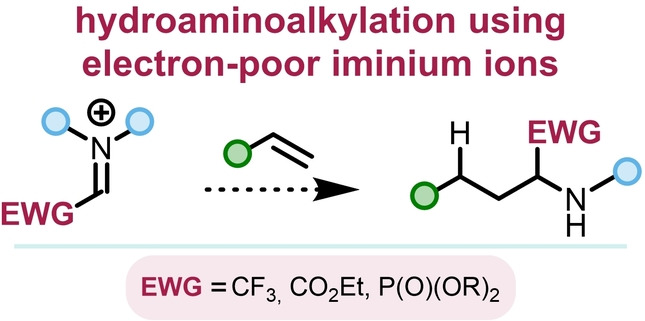

Iminium ions are privileged intermediates for the synthesis of amines,^[1]^ involved in efficient processes such as the Mannich reaction,^[2]^ reductive amination^[3]^ or nucleophilic additions.[^4^, ^5^, ^6^] In 2018, Doyle reported a three‐component coupling of amines, aryl aldehydes and bromides/triflates that affords tertiary amines via nickel‐catalyzed reduction of an iminium intermediate, generated in situ by condensation (Scheme 1a).^[7]^ In the same year, the Gaunt group developed photocatalytic conditions for a multicomponent hydroaminoalkylation reaction (Scheme 1b).^[8]^ The reaction, applied to the total syntheses of (−)‐FR901483 and (+)‐TAN1251C, proceeds through SET reduction of a transient iminium ion, once more generated in situ by condensation of an amine with an aldehyde or ketone.^[9]^ Last year, the same group developed conditions for a radical‐based synthesis of amines via radical addition to a transient iminium intermediate (Scheme 1c).^[10]^ Intriguingly, these three state‐of‐the‐art methods all require the deployment of a stoichiometric reducing agent (Scheme 1).

**Scheme 1:**
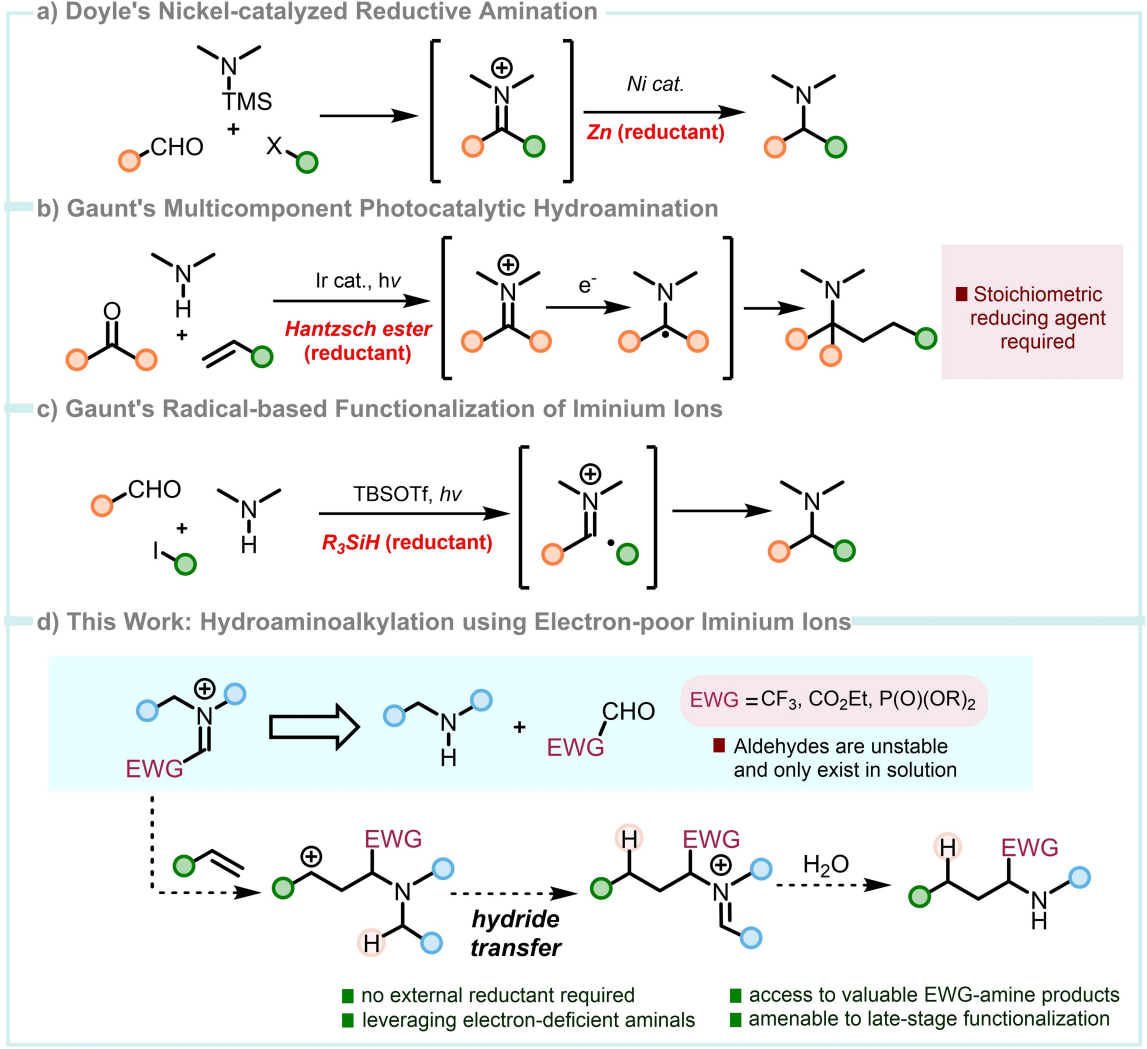
a,b,c) Modern methods for the synthesis of amines. d) Development of a metal‐free hydroaminoalkylation of olefins and alkynes.

The use of a carbonyl condensation approach to generate the pivotal iminium intermediate, while flexible, restricts access to highly electron‐deficient iminium ions such as those shown in Scheme 1d. Indeed, such species are scarcely described in the literature,[^11^, ^12^, ^13^] but notably have not been used in conjunction with unactivated alkenes. This is unsurprising given that aldehydes such as e.g. (2,2,2‐trifluoro)acetaldehyde are fleeting species, commonly only available as the hydrate/hemiacetal—yet, the possibility to access amination products carrying a CF_3_‐group is of potentially enormous value for drug discovery and materials science alike, due to the interesting properties imparted by the trifluoromethyl substituent.[^14^, ^15^, ^16^]

We considered a different approach towards these reactive intermediates, namely their in situ preparation from aminals, themselves stable precursors.[^17^, ^18^, ^19^] It is noteworthy that such intermediates might allow access to a range of highly functionalized amines that cannot be obtained by any of the previously mentioned methodologies (Scheme 1d).

Here, we present the leveraging of highly electron‐deficient iminium ions in the synthesis of valuable trifluoromethylated amines, aminoesters and aminophosphonates by hydroaminoalkylation of unactivated alkenes and alkynes. Our concept relies on the pairing of a rapid intermolecular aza‐Prins‐like reaction of these electron‐deficient species with a stereoselective internal reduction event (1,5‐hydride transfer)[^18^, ^20^, ^21^, ^22^, ^23^] ensuring that C−C bond formation is coupled to reduction in a redox‐neutral manner and, contrasting the methods described before, without requiring an external reductant (Scheme 1d). This strategy enables the preparation of a broad range of valuable amines and late‐stage functionalization of complex architectures.

We focused our investigation on commercially available, CF_3_‐substituted aminal **A** (shown in Scheme 2), originally developed by Dolbier for the synthesis of propargylic and allylic α‐trifluoromethylamines,^[11]^ as an ideal benchmark to test our hypothesis. We quickly found that the increased electrophilicity and reactivity of **A** enabled the use of exceptionally mild reaction conditions. In particular, at temperatures as low as –10 °C, we observed the combination of **A** with unactivated alkenes to deliver the desired secondary α‐trifluoromethylamines (Scheme 2A; see Supporting Information for the full optimization). As known methods to access α‐trifluoromethylamines are often multistep procedures,[^24^, ^25^, ^26^, ^27^, ^28^, ^29^, ^30^, ^31^, ^32^, ^33^] or rely on unstable reagents under oxidative conditions,[^34^, ^35^, ^36^] the coupling of **A** with readily available alkene feedstocks provides a direct solution to this synthetic challenge. The scope of this reaction proved to be wide: both linear (**1 a**, **2 a**) and cyclic alkenes (**3 a**, **4 a**) were converted to the α‐trifluoromethylaminated products in good to excellent yields (Scheme 2A) and high functional‐group tolerance was observed when halides (**5 a**), esters (**6 a**) and even a free alcohol (**7 a**) were shown to be well tolerated Alkynes also reacted smoothly (**8 a**, **9 a** and **11 a**) and in stereoselective manner, delivering di‐ and trisubstituted olefinic products as single double‐bond isomers.[^12^, ^37^] An enyne substrate displayed selectivity for the triple bond (**10 a**), a useful chemoselectivity trait. Moreover, a terminal alkyne was found to react preferentially to an internal one (**12 a**).

**Scheme 2:**
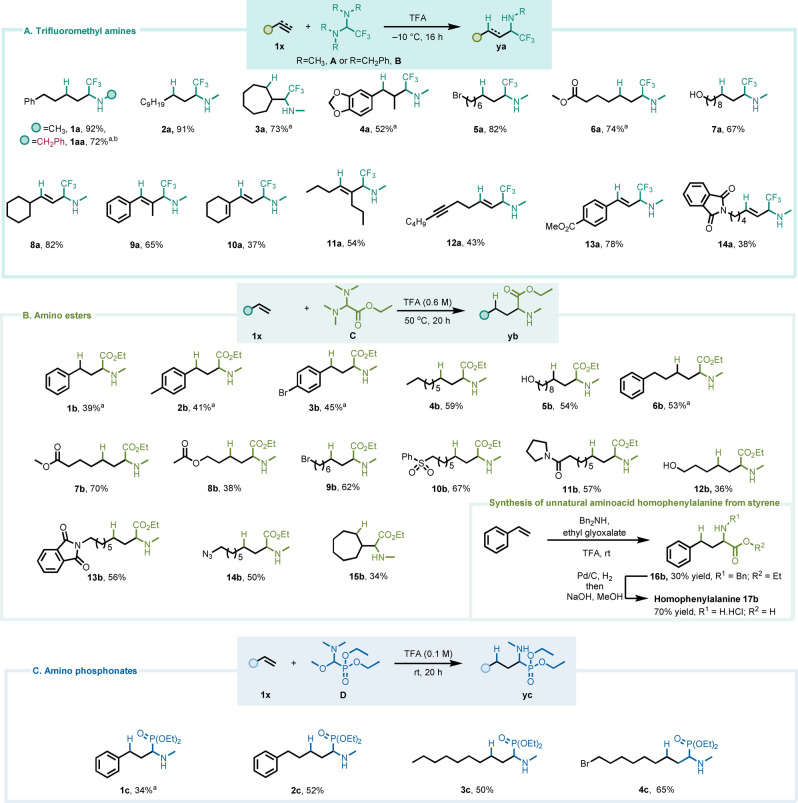
Substrate scope for the synthesis of α‐trifluoromethyl, α‐carboethoxy and α‐phosphonyl amines by hydroaminoalkylation of alkenes and alkynes. [a] Reaction conducted at 20 °C. [b] Reaction using in situ generated Aminal **B** instead of **A**—See Supporting Information for details.

A remarkable example is portrayed in **1 aa**, where an in situ generated benzyl aminal **B** could be employed to yield a benzylamine product. This result further highlights the practicability of the method, as diversely substituted aminals can be rapidly synthesized and used without further purification.

After exploring the scope of possible substrates for trifluoromethyl‐aminoalkylation, we turned our attention towards different highly electron‐deficient iminium ions. In particular, we recognized the appeal of an unprecedented direct formation of α‐amino acid derivatives from unactivated alkenes. This is a long sought‐after transformation in organic synthesis, with a direct, one‐step strategy yet to emerge.^[38]^

In the event, carbethoxyaminal^[39]^ **C** (Scheme 2B) proved suitable for this task in reaction with alkenes under gentle heating (see Supporting Information for the full optimization). Electron‐rich (**1 b**, **2 b**) and ‐poor styrenes (**3 b**) performed comparably well and were converted to the corresponding α‐amino acid derivatives in moderate yields. The functional‐group tolerance of the process was not affected, and it was possible to obtain α‐amino esters bearing a hydroxyl (**5 b**), an ester (**7 b**) or a sulfone (**10 b**) moiety along the aliphatic chain.

Even a primary bromide (**9 b**) did not undergo nucleophilic substitution under the reported conditions, and an azide (**14 b**) also remained untouched. As a sample application, the presented method enables a straightforward synthesis of the unnatural α‐amino acid homophenylalanine (**17 b**) in only three steps from styrene: as shown, the hydroaminalkylation can, in this case, be conducted as a 3‐component coupling of a secondary amine, a glyoxalate and an olefin (Scheme 2B).

Finally, setting our vistas on α‐aminophosphonates as targets, we found that acidolysis of the hemiaminal **D** (Scheme 2C) also resulted in the selective formation of an iminium species competent for redox‐neutral coupling to alkenes.^[40]^ As for the preceding cases, both styrenes and aliphatic alkenes afforded the desired adducts (**1 c**–**4 c**) in good yields.

The methods laid out herein offer opportunities for application to biological settings. As shown in Scheme 3A and B, a trifluoromethylated derivative of the 1^st^ generation anti‐histaminic chlorpheniramine could be swiftly prepared in a tandem hydroaminoalkylation/Eschweiler–Clarke reaction (**16 a**). In a similar fashion, an analog resembling the famed antimalarial drug chloroquine was also successfully synthesized (**15 a**). The natural product quinine was also successfully converted to an α‐trifluoromethylamine derivative (**17 a**) in an interesting example of late‐stage functionalization. Due to quinine's well‐known antimalarial activity,[^41^, ^42^] we were intrigued by this analog and its potential as an antimalarial agent. Quinine analog **17 a** was used to treat two distinct cell‐lines, the first one containing P. falciparum and the second containing L6 rat cells, in order to check for its antimalarial activity and cytotoxicity, respectively. **17 a** achieved potent in vitro inhibition (IC_50_=24 nM) of the growth of P. falciparum and the evaluation of its effect on L6 rat cells showed it to be non‐cytotoxic. Importantly, the antimalarial activity exhibited by **17 a** is comparable to that of chloroquine sulphate, a drug used to treat similar diseases.^[43]^

**Scheme 3:**
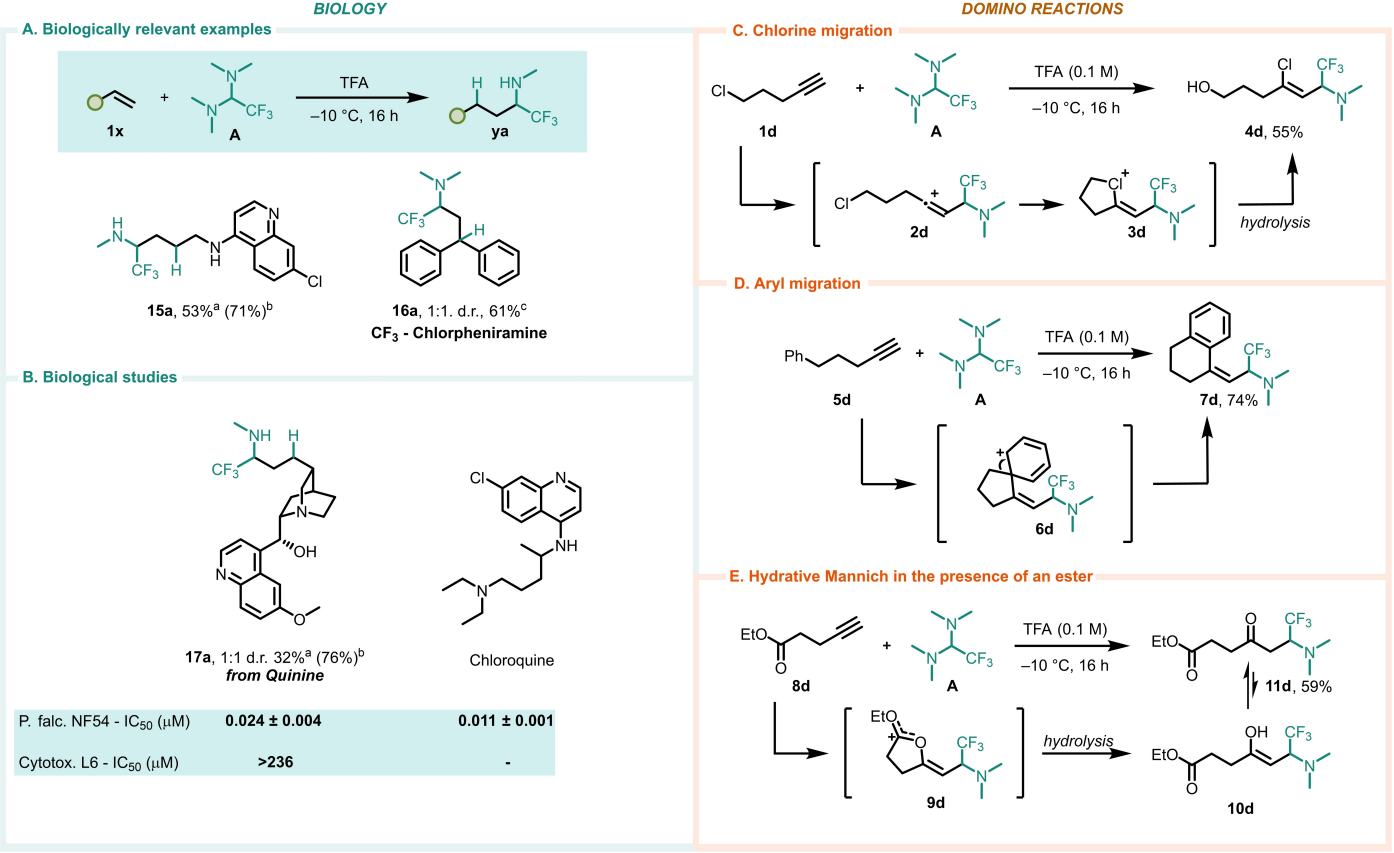
A, B) Selected applications to biological problems. C–E) Mechanistic probes. [a] Reaction conducted at 75 °C. [b] Based on recovered starting material. [c] With 10 equiv of paraformaldehyde and HCOOH at 100 °C—See Supporting Information for details.

Finally, integration of these reactions into interesting domino processes presents useful synthetic opportunities (Scheme 3C–E). For instance, halogen or arene migration processes enable relatively simple (alkyne **5 d**) and commercially available (5‐chloropentyne (**1 d**)) starting materials to directly deliver aminated products of higher complexity (Scheme 3C and D). Strategic positioning of an ester moiety (**8 d**) leads to interception and delivers products of a formal hydrative Mannich transform (Scheme 3E).

In summary, we have presented an approach leveraging highly electron‐deficient iminium ions which allows their coupling with unactivated unsaturated partners without requiring a reductant. In doing so, we provide one‐step synthetic routes to trifluoromethylated amines, aminoesters and aminophosphonates of potentially high value for medicinal, pharmaceutical and materials chemistry. The described late‐stage derivatization of quinine suggests a general strategic deployment of this reaction to bioactive substances carrying an unsaturation.

## Conflict of interest

The authors declare no conflict of interest.

## Supporting information

As a service to our authors and readers, this journal provides supporting information supplied by the authors. Such materials are peer reviewed and may be re‐organized for online delivery, but are not copy‐edited or typeset. Technical support issues arising from supporting information (other than missing files) should be addressed to the authors.

Supporting Information

## Data Availability

The data that support the findings of this study are available from the corresponding author upon reasonable request.
