# Supplementary material for: Leveraging Electron‐Deficient Iminium Intermediates in a General Synthesis of Valuable Amines
Source: Angew Chem Int Ed Engl. 2022 Mar 16;61(20):e202115435. doi: 10.1002/anie.202115435 (PMC9311413; doi:10.1002/anie.202115435)
Supplement: Supplementary file 1 — Supporting Information [file ANIE-61-0-s001.pdf]

Angewandte Chemie
Eine Zeitschrift der Gesellschaft Deutscher Chemiker

Supporting Information

Leveraging Electron-Deficient Iminium Intermediates in a General Synthesis of Valuable Amines

*C.-S. Hsu, C. R. Gonçalves, V. Tona, A. Pons, M. Kaiser, N. Maulide**

Supporting Information

Table of Contents

1. General Information 2

2. Procedures for the Synthesis of Starting Materials 3

   2.1. Experimental procedures for the Synthesis of Aminals 3

   2.2. Other starting materials 6

3. General experimental procedures for Hydroaminoalkylation 11

   3.1. General procedure for the preparation of α-trifluromethylamines using aminal A or D 11

   3.2. General procedure for the preparation of α-aminoesters using ethyl 2,2-bis(dimethylamino)acetate (B - aminal) 12

   3.3. General procedure for the preparation of α-aminophosphonates using diethyl ((dimethylamino)(methoxy)methyl)phosphonate (C - hemiaminal) 13

4. Characterization 14

   4.1. α – trifluoromethylamines 14

   4.2. α – aminoesters 24

   4.3. α – aminophosphonates 32

   4.4. α – trifluoromethylamines derived from mechanistic elucidation 34

   4.5. Direct synthesis of Homophenylalanine (17b) from corresponding alkene 36

5. Biological studies 37

   5.1. Antimalarial and cytotoxicity assay 38

6. Spectral Data 40

1. General Information

All glassware was oven dried at 100 °C before use. All solvents were distilled from appropriate drying agents prior to use or directly taken from commercial sealed bottles under an atmosphere of argon. All reagents were used as received from commercial suppliers unless otherwise stated. Neat infrared spectra were recorded using a Perkin-Elmer Spectrum 100 FT-IR spectrometer. Wavenumbers (ν) are reported in cm^{-1}. Mass spectra were obtained using a Finnigan MAT 8200 or (70 eV) or an Agilent 5973 (70 eV) spectrometer, using electrospray ionization (ESI). All ^{1}H NMR, ^{13}C NMR and ^{19}F NMR experiments were recorded using Bruker AV-400, AV-600 and AV-700 spectrometers at 300 K. Chemical shifts (δ) are quoted in ppm and coupling constants (*J*) are quoted in Hz. The 7.26 ppm resonance of residual $CHCl_3$ for proton spectra and 77.16 ppm resonance for carbon spectra were used as internal references. For residual MeOH the selected resonance was 3.31 ppm in the proton spectra and 49.0 ppm in the carbon spectra. ^{1}H NMR splitting patterns were designated as singlet (s), doublet (d), triplet (t), quartet (q), pentet (p) or combinations thereof, as well as broad signal (br). Splitting patterns that could not be interpreted were designated as multiplet (m). Reaction progress was monitored by thin layer chromatography (TLC) performed on aluminum plates coated with kieselgel F254 with 0.2 mm thickness. Visualization was achieved by a combination of ultraviolet light (254 nm) and acidic potassium permanganate. Flash column chromatography was performed using silica gel 60 (230–400 mesh, Merck and co.). Staining agents were freshly prepared when purifying α–trilfluoromethylamines (low staining ability). **Aminal A** - 2,2,2-trifluoro-*N*,*N*,*N*',*N*'-tetramethylethane-1,1-diamine – was purchased from FluoroChem and Apollo Scientific.

2. Procedures for the Synthesis of Starting Materials

2.1. Experimental procedures for the Synthesis of Aminals

N,N,N',N'-tetrabenzyl-2,2,2-trifluoroethane-1,1-diamine– hemiaminal B

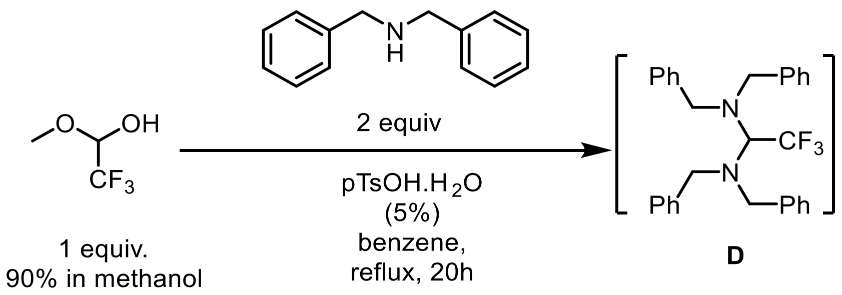

To a round bottom flask containing trifluoroacetaldehyde methyl hemiacetal(0.56 mL, 5.25 mmol, 1.00 equiv., 90% in methanol) was added dibenzylamine (1.92 mL, 10 mmol, 2.00 equiv) and benzene (20ml) at 0 °C. Afterwards pTsOH.H_2O (48mg, 5%) was added to the solution. A Dean-stark apparatus was then assembled and the reaction heated to reflux for 20h. After cooling, the solution was evaporated and used as a mixture in the upcoming reactions.

Hemiaminal D was used as such and after considering full conversion – crude:

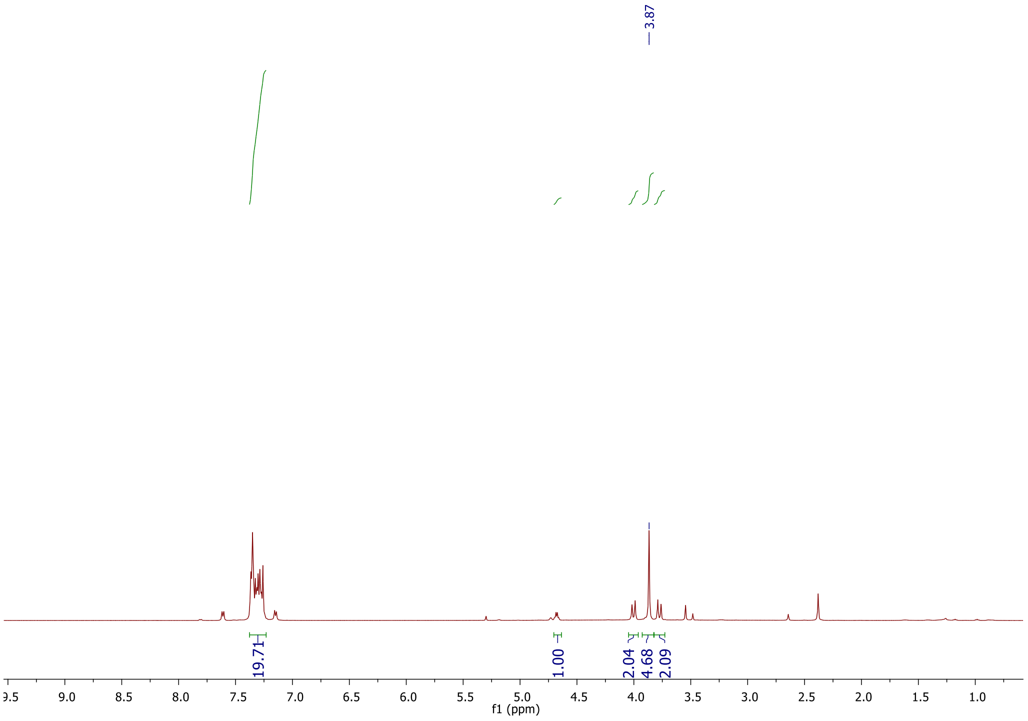

ethyl 2,2-bis(dimethylamino)acetate – aminal C

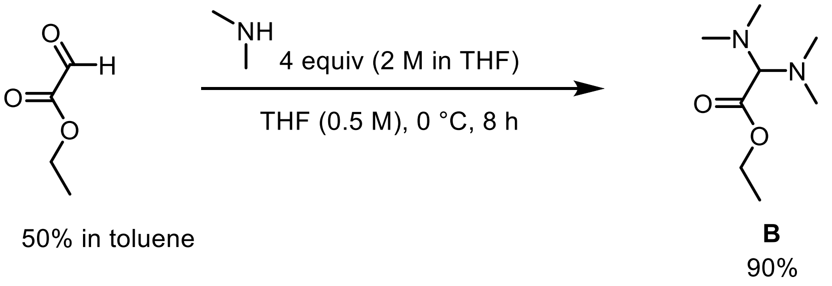

A solution of ethyl glyoxalate (2 mL, 10 mmol, 1 equiv, 50% in toluene) was gently heated to 60 °C for 1 h. After that time, the solution was cooled to 0 °C, diluted with THF (3 mL) and dimethylamine (15 mL, 40 mmol, 4 equiv, 2 M in THF) was added. After 8 h, the solution was passed through a short plug of basic alumina and flushed with diethyl ether as eluent. Evaporation of the solvent afforded the product in 90% yield as a yellow oil. Spectral data and experimental procedure in accordance with the literature.[1]

^{1}H NMR (600 MHz, $CDCl_3$): δ 4.24 (q, *J* = 7.1 Hz, 2H), 3.12 (s, 1H), 2.28 (s, 12H), 1.31 (t, *J* = 7.1 Hz, 3H).

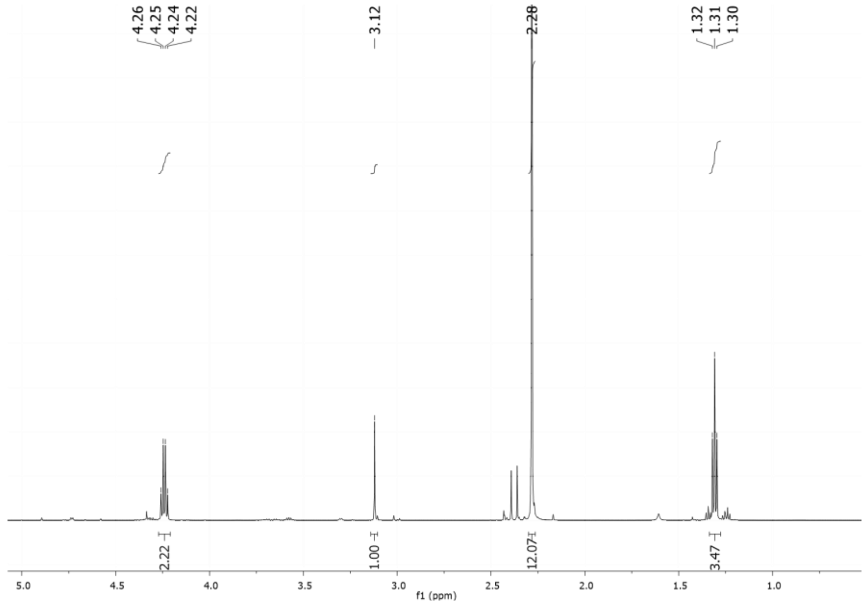

[1] B. Merla, H. J. Grumbach, N. Risch, Synthesis **1998**, *11*, 1609–1614.

diethyl ((dimethylamino)(methoxy)methyl)phosphonate – hemiaminal D

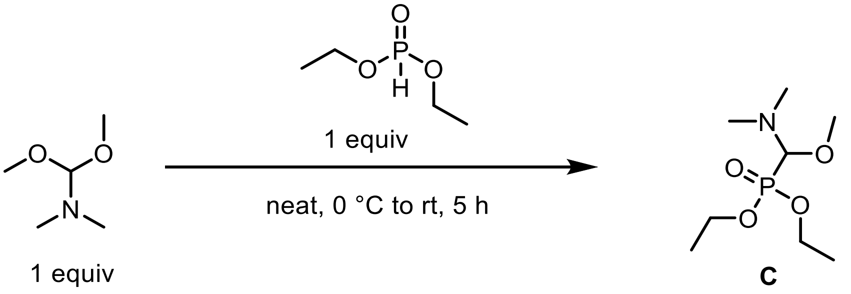

C

78%

To a round bottom flask containing dimethylformamide dimethyl acetal (11.9 g, 0.10 mol, 1.00 equiv) was added diethyl phosphonate (13.8 g, 0.10 mol, 1.00 equiv) at 0 °C. After stirring for 5 h at room temperature, the crude mixture was distilled using Kugelrohr distillation to afford the hemiaminal C as a colourless liquid (78%). Spectral data and experimental procedure are in accordance with the literature.[2]
^{1}**H NMR (600 MHz, CDCl₃):** δ 4.25 – 4.13 (m, 4H), 4.10 – 4.08 (m, 1H), 3.48 (s, 3H), 2.54 (s, 6H), 1.37 – 1.28 (m, 6H). *Hemiaminal C was used as such.*

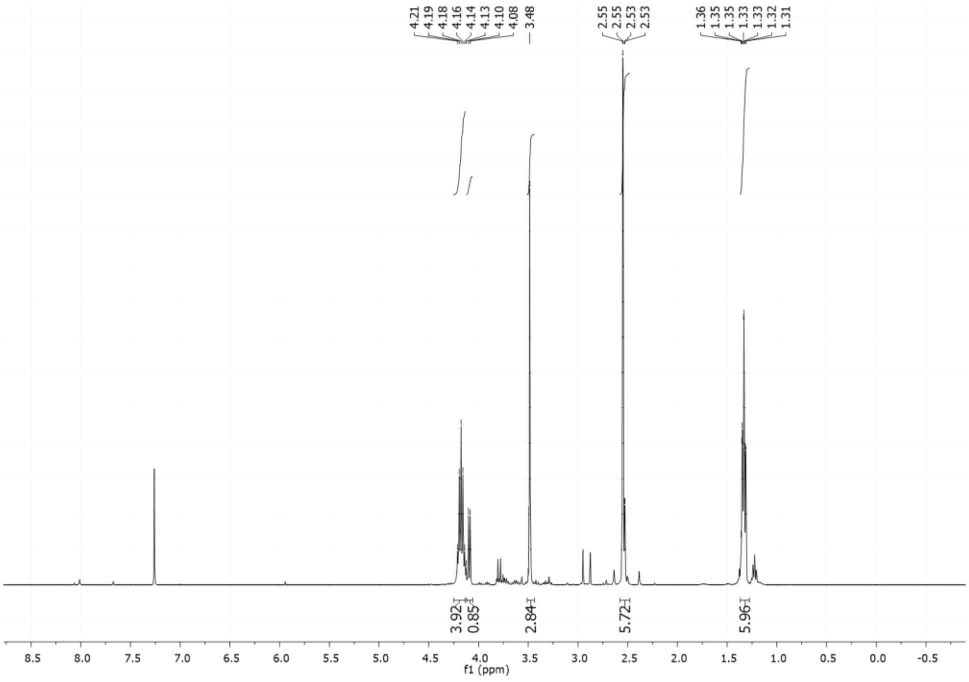

[2] N. Risch, S. Piper, A. Winter, A. Lefarth-Risse, *Eur. J. Org. Chem.* **2005**, 387–394.

2.2. Other starting materials

Unless otherwise stated, all starting material are commercially available.

(oct-7-en-1-ylsulfonyl)benzene - used in the synthesis of compound **10b**

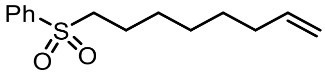

To a solution of 8-bromo-hexene (0.96 g, 5.0 mmol, 1.0 equiv) in DMF (10 mL, 0.44 M) was added sodium benzenesulfinate (0.98 g, 6.0 mmol, 1.2 equiv). After 6 h, diethyl ether (20 mL) was added to the reaction mixture and the resulting solution was washed with brine (3 x 20 mL). The dried (Na_2SO_4) extract was concentrated *in vacuo* and purified by chromatography over silica gel, eluting with 2-10% EtOAc / heptane to give the desired sulfone (0.7 g, 67%) as a colorless oil. Spectral data is in accordance with the literature.[3]

^{1}H NMR (600 MHz, CDCl$_3$): δ 7.91 (dd, *J* = 5.3, 3.3 Hz, 2H), 7.65 (ddd, *J* = 6.6, 3.9, 1.2 Hz, 1H), 7.57 (dd, *J* = 10.4, 4.7 Hz, 2H), 5.76 (ddt, *J* = 16.9, 10.2, 6.7 Hz, 1H), 5.04 – 4.86 (m, 2H), 3.17 – 2.99 (m, 2H), 2.00 (app q, *J* = 6.9 Hz, 2H), 1.80 – 1.62 (m, 2H), 1.39 – 1.19 (m, 6H).

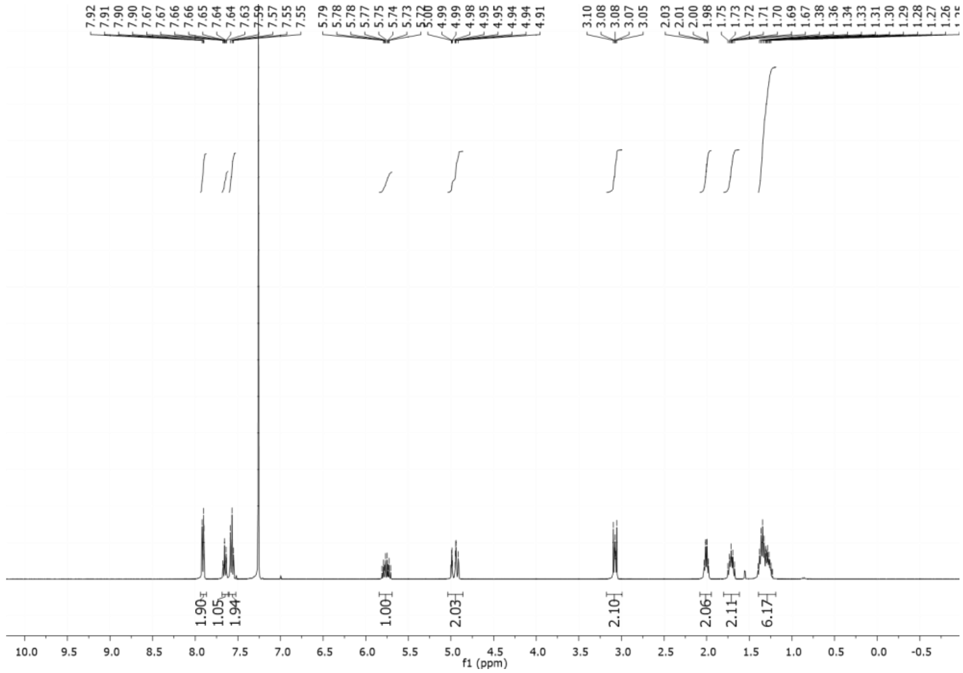

[3] A. C. Bonaparte, M. P. Betush, B. M. Panseri, D. J. Mastarone, R. K. Murphy, S. S. Murphree, A. C. Bonaparte, *Org. Lett.* **2011**, *6,* 1447-1449.

2-(oct-7-en-1-yl)isoindoline-1,3-dione - used in the synthesis of compound **13b**

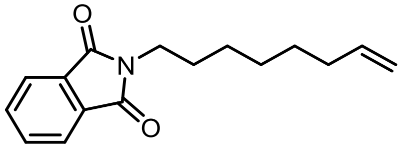

A suspension of 8-bromo-1-octene (1.14 g, 5.96 mmol, 1.00 equiv) and potassium phthalimide (1.21 g, 6.35 mmol, 1.00 equiv) in dry DMF (0.44 M, 14 mL) was heated at 60 °C for 24 h. The resulting cloudy pale-yellow mixture was allowed to cool to room temperature and then solids were filtered off. Brine (10 mL) was added and the mixture was extracted with diethyl ether (3 x 20 mL). The combined ether extracts were washed with 20 mL of brine and then dried over K_2CO_3. The ether solution was filtered and the solvent removed *in vacuo* to give the title compound (1.2g, 80%) as a pale-yellow liquid. Spectral data is in accordance with the literature.[4]

^{1}H NMR (600 MHz, $CDCl_3$): δ 7.87 – 7.72 (m, 2H), 7.72 – 7.61 (m, 2H), 5.74 (ddtd, *J* = 10.1, 8.0, 6.7, 1.3 Hz, 1H), 4.98 – 4.82 (m, 2H), 3.72 – 3.56 (m, 2H), 1.99 (app q, *J* = 6.5 Hz, 2H), 1.69 – 1.55 (m, 2H), 1.39 – 1.24 (m, 6H).

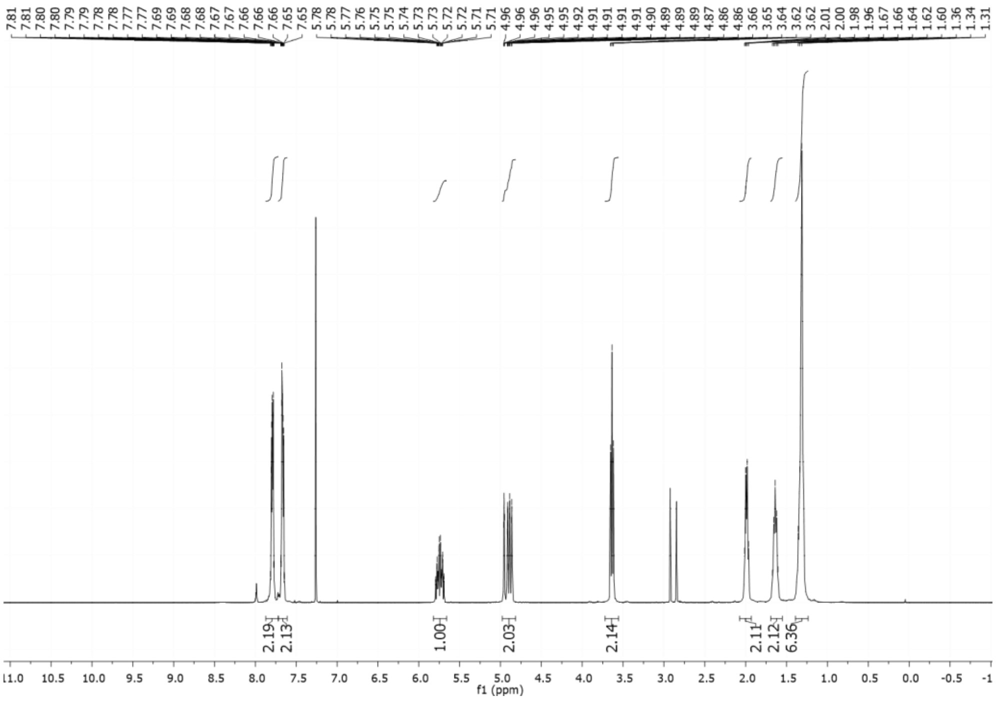

[4] F. Zhang, S. Das, A. J. Walkinshaw, A. Casitas, M. Taylor, M. G. Suero, M. J. Gaunt, *J. Am. Chem. Soc.* **2014**, *136,* 8851-8854.

8-azidooct-1-ene - used in the synthesis of compound **14b**

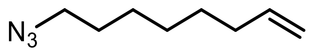

NaN_3 (0.78 g, 11.9 mmol, 2.00 equiv) was added in one portion to a stirred solution of 8-bromo-1-octene (1.14 g, 5.96 mmol, 1.00 equiv) in dry DMF (0.44 M, 14.0 mL) at 80 °C. After 16 h at 80 °C, the solution was poured into H_2O (50.0 mL). The reaction mixture was extracted with ethyl acetate (3 x 25.0 mL) and the combined organic phases were washed with brine (10.0 mL). The organic phase was dried over anhydrous $MgSO_4$ and concentrated *in vacuo*. The title compound was isolated (0.80 g, 90%) as a colorless oil. Spectral data is in accordance with the literature. [5]

^{1}H NMR (600 MHz, $CDCl_3$): δ 5.80 (ddt, *J* = 16.9, 10.2, 6.7 Hz, 1H), 5.05 – 4.90 (m, 2H), 3.26 (t, *J* = 6.9 Hz, 2H), 2.05 (td, *J* = 6.9, 1.2 Hz, 2H), 1.60 (dt, *J* = 14.1, 6.9 Hz, 2H), 1.41 – 1.30 (m, 6H).

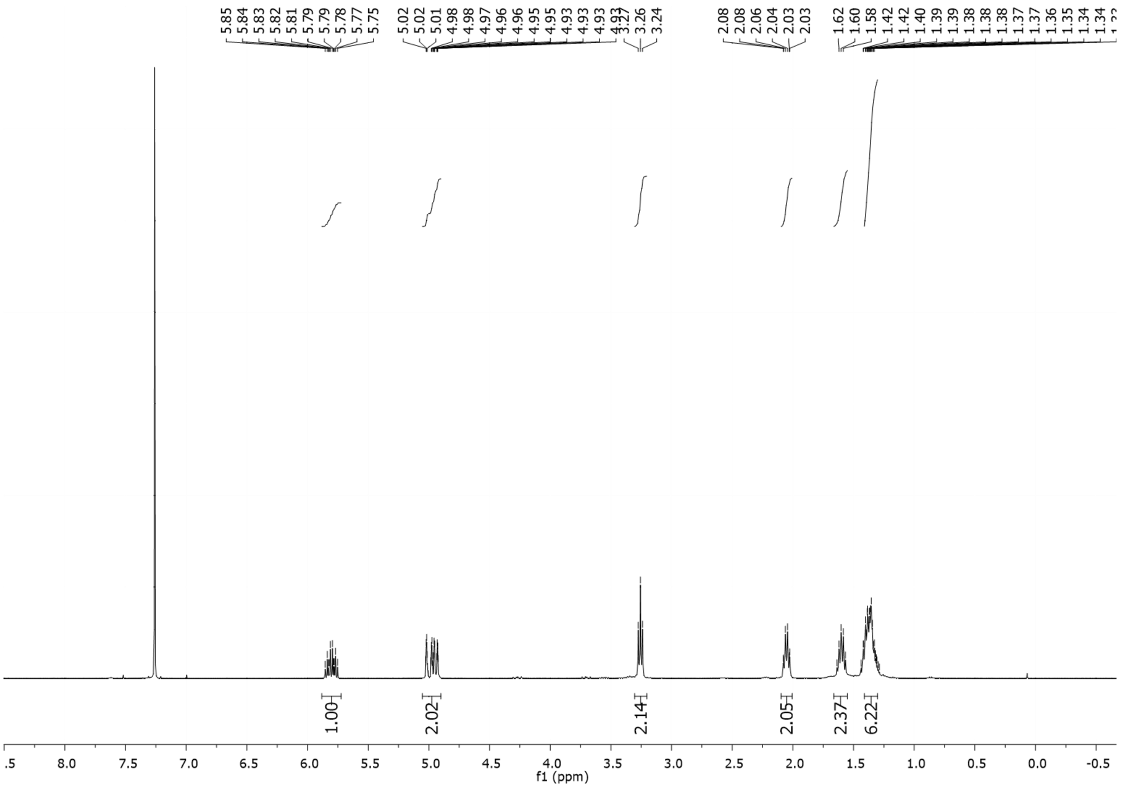

[5] M. Steinmann, M. Wagner, F. R. Wurm, *Chem.Eur. J.* **2016**, *22,* 17329-17338.

***N*-allyl-7-chloroquinolin-4-amine** - used in the synthesis of compound **15a**

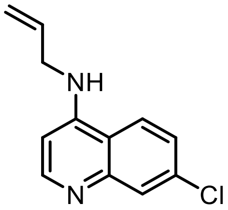

A round-bottom flask containing 10 mL of ethanol was charged with 4,7-dichloroquinoline (990 mg, 5 mmol, 1 equiv), allylamine (1.13 mL, 15.0 mmol, 3.00 equiv.) and triethylamine (2.09 mL, 15.0 mmol, 3.00 equiv). The reaction mixture was heated at 90°C for 16 hours. After cooling to room temperature, the solvent was removed under reduced pressure and the resulting solid was purified by column chromatography. The title compound was isolated in 63% as a yellow solid. Spectral data is in accordance with the literature.[6]

^{1}H NMR (600 MHz, $CDCl_3$): δ 8.54 (d, *J* = 5.3 Hz, 1H), 7.97 (d, *J* = 2.1 Hz, 1H), 7.68 (d, *J* = 8.9 Hz, 1H), 7.38 (dd, *J* = 8.9, 2.2 Hz, 1H), 6.43 (d, *J* = 5.3 Hz, 1H), 6.00 (ddt, *J* = 17.1, 10.6, 5.4 Hz, 1H), 5.36 (ddd, *J* = 17.2, 2.9, 1.6 Hz, 1H), 5.29 – 5.27 (m, 1H), 5.13 (brs, 1H), 3.99 (m, 2H).

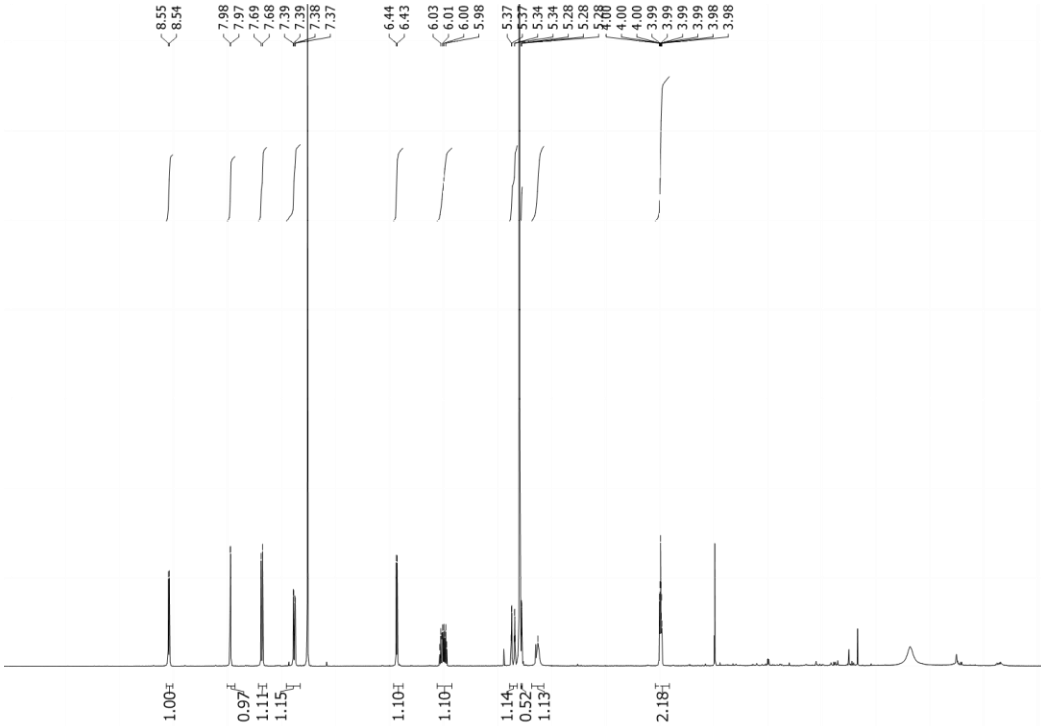

[6] Experimental procedure based on M. V. de Souza, K. C. Pais, C. R. Kaiser, M. A. Peralta, M. de L Ferreira, M. C. Lourenço, *Bioorg. Med. Chem.* **2009**, *17,* 1474-1480.

2-(1-(4-chlorophenyl)vinyl)pyridine – used in the synthesis of compound **16a**

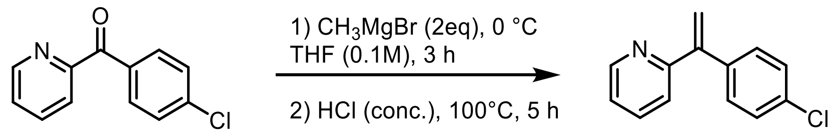

To a 0°C THF solution of (4-chlorophenyl)(pyridin-2-yl)methanone (1.0 g, 4.59 mmol, 1.00 equiv) was added, dropwise, methyl magnesium bromide (3.0 mL of a 3 M solution in Et_2O, 9.19 mmol, 2.00 equiv). After immediate color change, the solution was stirred at room temperature for 3 h. The solution was then diluted with NH_4Cl and extracted with ethyl acetate (3 x 25.0 mL). The combined organic phases were dried over anhydrous $MgSO_4$ and concentrated *in vacuo*. The resulting yellow oil was used in the following step without purification. To a round-bottom flask containing the previously obtained tertiary alcohol, were added 10 mL of concentrated HCl (36%). The resulting solution was then heated to 100°C. After 15 h, the acid was neutralized by addition of a 1 M NaOH solution, and subsequently extracted with DCM (3 x 25.0 mL). The combined organic phases were dried over anhydrous $MgSO_4$ and concentrated *in vacuo*. The title compound was obtained in 55% as a yellow oil after column chromatography.[7]

^{1}H NMR (600 MHz, $CDCl_3$): δ 8.58 – 8.57 (m, 1H), 8.02 – 8.00 (m, 1H), 7.61 – 7.57 (m, 1H), 7.46 – 7.39 (m, 1H), 7.28 – 7.26 (m, 2H), 7.22 – 7.20 (m, 1H), 7.18 – 7.14 (m, 1H), 5.90 (d, *J* = 1.1 Hz, 1H), 5.54 (d, *J* = 1.1 Hz, 1H).

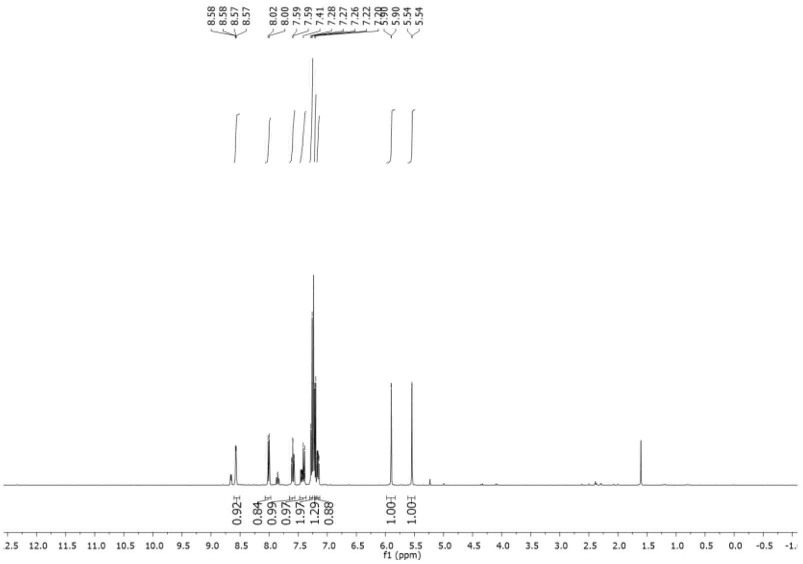

[7] H. Yang, E. Wang, P. Yang, H. Lv, X. Zhang, *Org. Lett.* **2017**, *19*, 5062–5065.

3. General experimental procedures for Hydroaminoalkylation

3.1. General procedure for the preparation of α-triflurομethylamines using aminal A or B

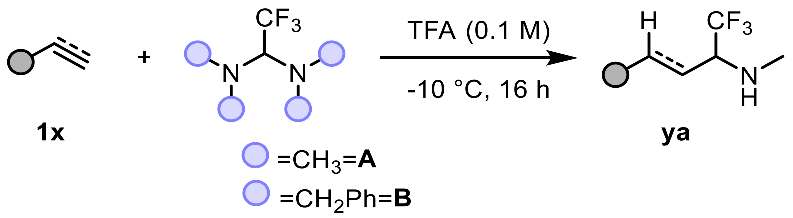

To a flame dried Schlenk flask charged with the aminal (**A or B**, 0.14 mL, 0.8 mmol, 4.00 equiv) was added, at -10 °C (using a cryostat – isopropanol), trifluoroacetic acid (2.0 mL, 0.1 M). After 5 min, the corresponding alkene/alkyne (0.2 mmol, 1.00 equiv) was added to the solution. The reaction was vigorously stirred at this temperature (-10 °C unless otherwise stated) for 16 h, after which it was allowed to warm to 0 °C. Then, aqueous NaOH (1 M) was added until a pH >7 was reached. The resulting biphasic mixture was separated and the aqueous phase was extracted with dichloromethane (3 x 100 mL/mmol). The combined organic phases were then dried over anhydrous K_2CO_3 and filtered. The filtrate was concentrated under reduced pressure to afford the crude product, which was purified by flash column chromatography on silica gel with dichloromethane/DMA system (DMA = dichloromethane/MeOH/NH_4OH 24:1:0.15) to afford the analytically pure desired product.

Optimization of the reaction conditions:

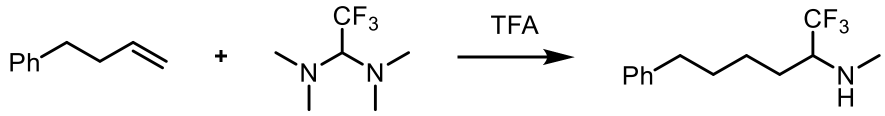

	Temperature (°C)	Time (h)	Conc. (M)	Product (% ^{1}H-NMR)
**1**	75	10	0.6	57
**2**	20	10	0.6	53
**3**	0	10	0.6	61
**4**	-10	10	0.6	64
**5**	-10	15	0.6	70
**6**	-10	13	0.3	55
**7**	-10	13	0.1	80
**8**	-10	16	0.1	90

3.2. General procedure for the preparation of α-aminoesters using ethyl 2,2-bis(dimethylamino)acetate (C - aminal)

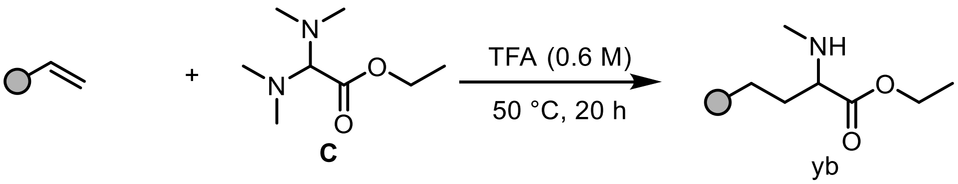

To a flame dried Schlenk flask charged with the aminal (**C**, 0.8 mmol, 4.00 equiv) was added, at -10 °C (using a cryostat – isopropanol), trifluoroacetic acid (0.33 mL, 0.6 M). After 5 min, the corresponding alkene (0.2 mmol, 1.00 equiv) was added to the solution. The reaction was heated to 50 °C and vigorously stirred (unless otherwise stated) for 20 h, after which it was cooled to 0 °C. Then, aqueous NaOH (1 M) was added until a pH >7 was reached. The resulting biphasic mixture was separated and the aqueous phase extracted with dichloromethane (3 x 100 mL/mmol). The combined organic phases were then dried over anhydrous K_2CO_3 and filtered. The filtrate was concentrated under reduced pressure to afford the crude product, which was purified by flash column chromatography on silica gel with dichloromethane/DMA system (DMA = dichloromethane/MeOH/NH_4OH 24:1:0.15) to afford the analytically pure desired product.

Optimization of the reaction conditions:

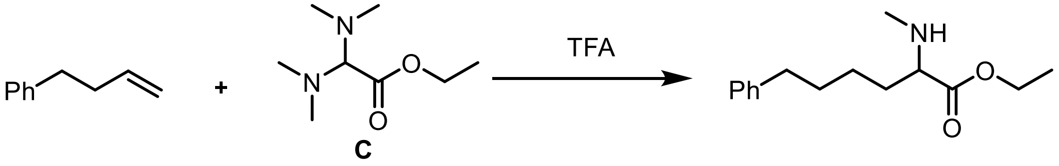

	Temperature (°C)	TFA (M)	Conversion (%^{1}HNMR)	Product (% ^{1}H-NMR)
**1**	-10	0.1	-	-
**2**	20	0.1	50	5
**3**	50	0.1	100	45
**4**	35	0.6	65	33
**5**	35	0.1	35	20
**6**	50	0.6	100	63

3.3. General procedure for the preparation of α-aminophosphonates using diethyl ((dimethylamino)(methoxy)methyl)phosphonate (D - hemiaminal)

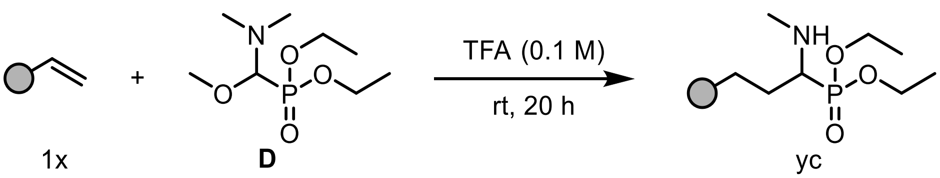

To a flame dried Schlenk flask charged with hemiaminal (**C**, 0.8 mmol, 4.00 equiv) was added, at 0°C, trifluoroacetic acid (2.0 mL, 0.1 M). After 5 min, the corresponding alkene (0.2 mmol, 1.00 equiv) was added to the solution. The reaction was vigorously stirred at rt (unless otherwise stated) for 20 h, after which it was cooled to 0 °C. Then, aqueous saturated $NaHCO_3$ was added until a pH >7 was reached. The resulting biphasic mixture was separated and the aqueous phase extracted with dichloromethane (3 x 100 mL/mmol). The combined organic phases were then dried over anhydrous K_2CO_3 and filtered. The filtrate was concentrated under reduced pressure to afford the crude product, which was purified by flash column chromatography on silica gel with dichloromethane/DMA system (DMA = dichloromethane/MeOH/NH_4OH 24:1:0.15) to afford the analytically pure desired product.

Optimization of the reaction conditions:

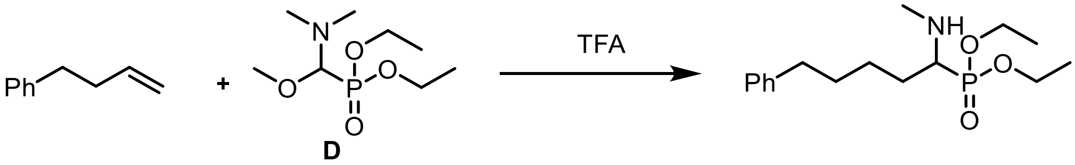

	Temperature (°C)	TFA (M)	Conversion (% ^{1}H-NMR)	Product (% ^{1}H-NMR)
**1**	-10	0.1	70	15
**2**	rt	0.1	100	52
**3**	rt	0.6	100	-
**4**	50	0.1	100	33

4. Characterization

4.1. α – trifluoromethylamines

1,1,1-trifluoro-*N*-methyl-6-phenylhexan-2-amine (1a)

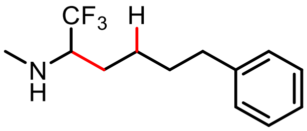

The title compound was isolated in 92% yield (48.0 mg) as a yellow oil.

^{1}H NMR (600 MHz, CDCl$_3$): δ 7.30 – 7.28 (m, 2H), 7.20 – 7.18 (m, 3H), 2.87 (dqd, *J* = 15.2, 7.6, 3.8 Hz, 1H), 2.69 – 2.58 (m, 2H), 2.52 (m, 3H), 1.76 – 1.61 (m, 3H), 1.55 (m, 1H) Hz), 1.52 – 1.37 (m, 2H), 1.07 (br s, 1H); **^{13}C NMR (151 MHz, CDCl$_3$)**: δ 142.4, 128.5, 128.2, 127.3 (q, *J* = 288 Hz), 125.9, 61.0 (q, *J* = 30 Hz), 35.8, 35.0, 31.4, 28.5, 25.4; **^{19}F NMR (565 MHz, CDCl$_3$): δ** -74.64 (d, *J* = 8.0 Hz); **IR (neat, cm^{-1}):** 2925, 2855, 2360, 2341, 1466; 1266; **HRMS (ESI$^+$):** calculated for [M+H]$^+$ $C_{13}H_{19}NF_3^+$: 246.1464, found 246.1468.

1,1,1-trifluoro-*N*-methyl-6-phenylhexan-2-amine (1aa)

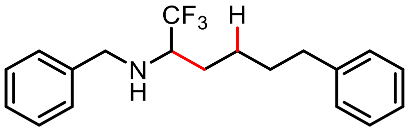

The title compound was isolated in 72% yield (46.0 mg) as a yellow oil, using Aminal B

^{1}H NMR (600 MHz, CDCl$_3$): δ 7.42 – 7.34 (m, 1H), 7.34 – 7.27 (m, 6H), 7.21 – 7.13 (m, 3H), 3.99 (d, *J* = 13.2 Hz, 1H), 3.80 (d, *J* = 13.2 Hz, 1H), 3.02 – 2.96 (m, 1H), 2.64 – 2.53 (m, 2H), 1.72 – 1.63 (m, 1H), 1.61 – 1.52 (m, 2H), 1.51 – 1.43 (m, 1H), 1.39 – 1.24 (m, 2H).**^{13}C NMR (151 MHz, CDCl$_3$)**: δ 142.2, 139.9, 128.9, 128.7, 128.4 (3C), 128.3 (2C), 127.2 (q, *J = 285 Hz*), 127.2 (2C), 125.8, 58.2 (q, *J = 27 Hz*), 51.9, 35.6, 31.1, 28.7 (2C), 25.1.

142.4, 128.5, 128.2, 127.3 (q, *J* = 288 Hz), 125.9, 61.0 (q, *J* = 30 Hz), 35.8, 35.0, 31.4, 28.5, 25.4; **^{19}F NMR (565 MHz, CDCl$_3$): δ** -74.65 (d, *J* = 6.0 Hz); **IR (neat, cm^{-1}):** 3064, 3028, 2930, 2860, 1494, 1265, 1146; **HRMS (ESI$^+$):** calculated for [M+H]$^+$ $C_{19}H_{22}NF_3^+$: 322.1777, found 322.1769

1,1,1-trifluoro-*N*-methyldodecan-2-amine (2a)

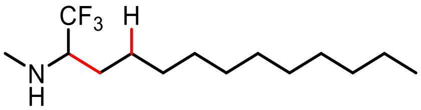

The title compound was isolated in 91% yield (53.0 mg) as a yellow oil.

^{1}H NMR (600 MHz, $CDCl_3$): δ 2.91 – 2.82 (m, 1H), 2.53 (s, 3H), 1.70 – 1.62 (m, 1H), 1.54 – 1.42 (m, 2H), 1.42 – 1.34 (m, 2H), 1.34 – 1.30 (m, 2H), 1.30 – 1.29 (m, 2H), 1.28 (d, *J* = 6.2 Hz, 3H), 1.27 – 1.25 (m, 9H), 1.01 (br s, 1H), 0.88 (t, *J* = 7.0 Hz, 3H); **^{13}C NMR (151 MHz, $CDCl_3$)**: δ 127.2 (q, *J* = 284.5 Hz), 61.0 (q, *J* = 26.7 Hz), 34.9, 31.9, 29.6 (2C), 29.5, 29.4, 29.3, 28.5, 28.4, 25.7, 22.7, 14.1; **^{19}F NMR (565 MHz, $CDCl_3$): δ** -74.69 (d, *J* = 8.0 Hz); **IR (neat, cm^{-1}):** 2925, 2855, 2360, 2341, 1466; 1266; **HRMS (ESI$^+$):** calculated for $[M+H]^+$ $C_{14}H_{29}NF_3^+$: 268.2247, found 268.2250.

1-cycloheptyl-2,2,2-trifluoro-*N*-methylethan-1-amine (3a)

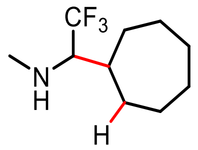

The title compound was isolated in 73% yield (31.0 mg) as a yellow oil. Reaction was performed at room temperature.

^{1}H NMR (600 MHz, $CDCl_3$): δ 2.84 – 2.74 (m, 1H), 2.55 (s, 3H), 1.93 – 1.85 (m, 1H), 1.77 – 1.64 (m, 4H), 1.62 – 1.54 (m, 3H), 1.54 – 1.38 (m, 4H), 1.36 – 1.28 (m, 1H), 1.07 (br s, 1H); **^{13}C NMR (151 MHz, $CDCl_3$)**: δ 127.4 (q, *J* = 286.6 Hz), 67.0 (q, *J* = 24.5 Hz), 39.7 , 36.6 , 32.6 , 28.3 , 28.2 , 27.7 , 27.4 , 27.2; **^{19}F NMR (565 MHz, $CDCl_3$)**: δ -70.20 (d, *J* = 8.3 Hz); **IR (neat, cm^{-1}):** 2924, 2852, 2359, 2341, 1730, 1464, 1274; **HRMS (ESI$^+$):** calculated for $[M+H]^+$ $C_{10}H_{19}NF_3^+$: 210.1464, found 210.1467.

4-(benzo[d][1,3]dioxol-5-yl)-1,1,1-trifluoro-*N*,3-dimethylbutan-2-amine (4a)

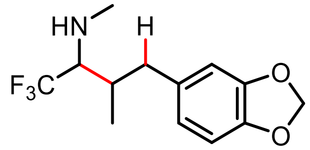

The title compound was isolated in 52% yield as a yellow oil (29.0 mg, d.r. of 1:1.3 – crude ^{1}HNMR). Reaction was performed at room temperature.

^{1}H NMR (600 MHz, $CDCl_3$): δ 6.92 – 6.76 (m, 3H), 6.02 – 5.95 (m, 2H), 4.96 (d, *J* = 4.2 Hz, 0.4H, d2), 4.70 (d, *J* = 4.2 Hz, 0.4H, d2), 3.37 (qd, *J* = 7.7, 2.3 Hz, 0.6H. d1), 3.16 (qd, *J* = 8.1, 2.4 Hz, 0.6H, d1), 2.66 (s, 1.3H, d2), 2.55 (s, 1.7H, d1), 2.23 – 2.09 (m, 1H), 1.52 (br s, 1H), 1.39 – 1.24 (m, 1H), 1.22 – 1.16 (d, *J* = 6.8 Hz, 1.7H, d1), 0.81 (d, *J* = 6.8 Hz, 1.3H, d2); **^{13}C NMR (151 MHz, $CDCl_3$):** δ 148.1 , 146.8 , 138.4 , 126.7 (q, 295.0 Hz), 118.8 , 108.4 , 106.2 , 101.2 , 77.87 , 60.0 (q, *J* = 25.8 Hz), 38.3 , 35.2 , 11.9; **^{19}F NMR (565 MHz, $CDCl_3$):** δ -69.39 (d, *J* = 8.2 Hz); **IR (neat, cm^{-1})**; 2969, 2929, 1661, 1619, 1447, 1416; **HRMS (ESI$^+$):** calculated for $[M+H]^+$ $C_{13}H_{17}O_2F_3^+$: 276.1206, found 276.1199.

10-bromo-1,1,1-trifluoro-*N*-methyldecan-2-amine (5a)

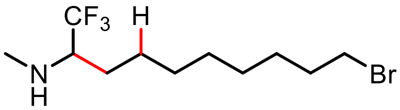

The title compound was isolated in 82% yield (37.0 mg) as a yellow oil.

^{1}H NMR (600 MHz, CDCl$_3$): δ 3.41 (t, *J* = 6.8 Hz, 2H), 2.86 (ddd, *J* = 12.0, 7.8, 3.9 Hz, 1H), 2.53 (s, 3H), 1.88 – 1.83 (m, 2H), 1.67 – 1.66 (m, 1H), 1.45 – 1.32 (m, 11H), 1.02 (br s, 1H); **^{13}C NMR (151 MHz, CDCl$_3$):** δ 127.3 (q, *J* = 284.6 Hz), 61.1 (q, J = 26.8), 35.0, 34.1, 32.9, 29.5, 29.3, 28.8, 28.6 (q, *J* = 1.7 Hz), 28.2, 25.8; **^{19}F NMR (565 MHz, CDCl$_3$):** δ -74. 62 (d, *J* = 7.6 Hz); **IR (neat, cm^{-1}):** 2928, 2857, 1461, 1263, 1146, 1111; **HRMS (ESI$^+$)**: calculated for $[M+H]^+$ $C_{11}H_{22}F_3Br^+$: 304.0882, found 304.0884.

Methyl 8,8,8-trifluoro-7-(methylamino)octanoate (6a)

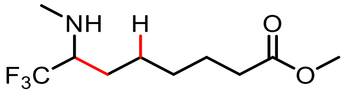

The title compound was isolated in 74% yield (35.0 mg) as a yellow oil. Reaction was performed at room temperature.

^{1}H NMR (600 MHz, CDCl$_3$): δ 3.67 (s, 3H), 2.90 – 2.82 (m, 1H), 2.52 (q, *J* = 1.1 Hz, 3H), 2.32 (t, *J* = 7.5 Hz, 2H), 1.73 – 1.61 (m, 3H), 1.56 – 1.47 (m, 1H), 1.47 – 1.29 (m, 4H), 1.00 (br s, 1H); **^{13}C NMR (151 MHz, CDCl$_3$):** δ 174.4, 127.5 (q, *J* = 284.6 Hz), 61.2 (q, *J* = 26.8 Hz), 51.9, 35.2, 34.3, 29.3, 28.7, 25.7, 25.1; **^{19}F NMR (565 MHz, CDCl$_3$):** δ -74.59 (d, *J* = 7.6 Hz); **IR (neat, cm^{-1}):** 2950, 2893, 1736, 1452; **HRMS (ESI$^+$):** calculated for $[M+H]^+$ $C_{10}H_{19}NO_2F_3^+$: 242.1362, found 242.1367.

12,12,12-trifluoro-11-(methylamino)dodecan-1-ol (7a)

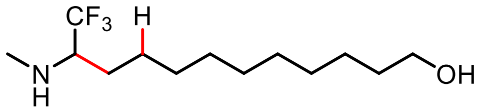

The title compound was isolated in 67% yield (32.0 mg) as a yellow oil.

^{1}H NMR (600 MHz, CDCl$_3$): δ 3.63 (t, *J* = 6.6 Hz, 2H), 2.91 – 2.87 (m, 1H), 2.53 (s, 3H), 1.75 (br s, 1H), 1.71 – 1.65 (m, 1H), 1.60 – 1.54 (m, 2H), 1.51 – 1.44 (m, 2H), , 1.39 – 1.29 (m, 14H); **^{13}C NMR (151 MHz, CDCl$_3$):** δ 127.2 (q, *J* = 285 Hz), 63.2, 61.0 (q, *J* = 27.0 Hz), 34.9, 32.9 , 29.7 , 29.6 (2C), 29.5 , 29.5 , 28.5, 25.9, 25.8; **^{19}F NMR (565 MHz, CDCl$_3$)**: δ -74.36 (d, *J* = 5.1 Hz); **IR (neat, cm^{-1}):** 3338, 2925, 2855, 1676, 1463, 1264, 1148, 1117, 1056; **HRMS (ESI$^+$):** [M+H]$^+$ calculated for $C_{13}H_{27}NOF_3^+$: 270.2039, found 270.2042.

(*E*)-4-cyclohexyl-1,1,1-trifluoro-*N*-methylbut-3-en-2-amine (8a)

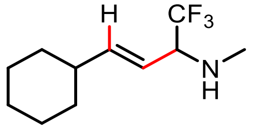

The title compound was isolated in 82% yield (36.0 mg) as a yellow oil.

^{1}H NMR (600 MHz, CDCl$_3$): 5.76 (dd, *J* = 15.5, 6.6 Hz, 1H), 5.24 (ddd, *J* = 15.6, 8.3, 1.4 Hz, 1H), 3.36 (app p, *J* = 7.5 Hz, 1H), 2.43 (s, 3H), 2.08 – 1.96 (m, 1H), 1.77 – 1.70 (m, 3H), 1.69 – 1.62 (m, 1H), 1.56 (s, 1H), 1.30 – 1.24 (m, 2H), 1.19 – 1.05 (m, 4H); **^{13}C NMR (151 MHz, CDCl$_3$):** δ 144.5, 125.7 (q, *J* = 285 Hz*)*, 119.8, 64.5 (q, *J* = 28 Hz), 40.6 , 34.1 , 32.8 , 32.7 , 32.7 , 26.2 , 26.0; **^{19}F NMR (565 MHz, CDCl$_3$)** δ -75.25 (d, *J* = 7.0 Hz); **IR (neat, cm^{-1}):** 2925, 2852, 2360, 2340, 1735; 1450; 1274; **HRMS (ESI$^+$):** calculated for [M+H]$^+$ $C_{11}H_{19}F_3N^+$: 222.1464, found 222.1466.

(*E*)-1,1,1-trifluoro-*N*,3-dimethyl-4-phenylbut-3-en-2-amine (9a)

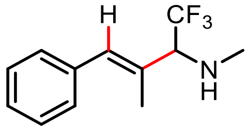

The title compound was isolated in 65% yield (29.0 mg) as a yellow oil.

^{1}H NMR (600 MHz, CDCl$_3$): δ 7.40 – 7.34 (m, 2H), 7.34 – 7.30 (m, 2H), 7.29 – 7.25 (m, 1H), 6.61 (s, 1H), 3.60 (q, *J* = 7.7 Hz, 1H), 2.47 (s, 3H), 1.92 (s, 3H); **^{13}C NMR (151 MHz, CDCl$_3$)**: δ 136.6, 132.9, 130.6, 129.1 (2C), 128.2 (2C), 127.1, 125.5 (q, *J* = 283 Hz), 69.7 (q, *J* = 28 Hz), 34.0, 13.6; **^{19}F NMR (565 MHz, CDCl$_3$)**: δ -72.6 (d, *J* = 7.6 Hz); **IR (neat, cm^{-1})**: 1447, 1350, 1257, 1154, 1134, 1091, 1014, 836, 752, 697. **HRMS (ESI^{+})**: calculated for $[M+H]^+$ $C_{12}H_{15}F_3N^+$: 230.1151, found: 230.1154.

(*E*)-4-(cyclohex-1-en-1-yl)-1,1,1-trifluoro-*N*-methylbut-3-en-2-amine (10a)

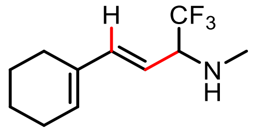

The title compound was isolated in 37% yield (14.0 mg) as a yellow oil.

^{1}H NMR (600 MHz, CDCl$_3$): δ 6.31 (d, *J* = 15.7 Hz, 1H), 5.83 (s, 1H), 5.32 (dd, *J* = 15.8, 8.5 Hz, 1H), 3.46 (p, *J* = 7.4 Hz, 1H), 2.45 (s, 3H), 2.17 – 2.11 (m, 4H), 1.72 – 1.66 (m, 2H), 1.64 – 1.58 (m, 2H), 1.40 (br s, 1H); **^{13}C NMR (151 MHz, CDCl$_3$)**: δ 140.3, 134.8, 132.0, 125.8 (q, *J* = 282 Hz), 117.3, 64.7 (q, *J* = 29 Hz), 34.3, 26.0, 24.5, 22.5, 22.4 ; **^{19}F NMR (565 MHz, CDCl$_3$)**: δ -75.1 (d, *J* = 7.2 Hz); **IR (neat, cm^{-1})**: 2928, 2860, 1650, 1450, 1365, 1261, 1155, 1127, 1101, 966, 792; **HRMS (ESI^{+})**: calculated for $[M-NHMe]^+$ $C_{10}H_{12}F_3^{+}$: 189.0886, found: 189.0889.

(*Z*)-1,1,1-Trifluoro-*N*-methyl-3-propylhept-3-en-2-amine (11a)

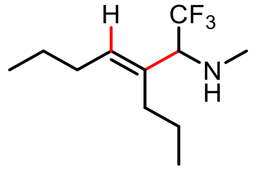

The title compound was isolated in 54% yield (25.0 mg) as a yellow oil.

^{1}H NMR (600 MHz, CDCl$_3$): δ 5.54 (t, *J* = 7.2 Hz, 1H), 3.34 (q, *J* = 7.7 Hz, 1H), 2.39 (s, 3H), 2.16 – 2.05 (m, 3H), 1.94 (ddd, *J* = 13.9, 10.6, 5.2 Hz, 1H), 1.47 – 1.36 (m, 4H), 0.92 (t, *J* = 7.3 Hz, 6H); **^{13}C NMR (151 MHz, CDCl$_3$)**: δ 132.9, 132.7, 125.9 (q, *J* = 282 Hz), 67.2 (q, *J* = 28 Hz), 34.5, 31.3, 30.0, 22.8, 22.1, 14.5, 13.9; **^{19}F NMR (565 MHz, CDCl$_3$)**: δ -73.2 (d, *J* = 7.6 Hz); **IR (neat , cm^{-1})**:, 2961, 2873, 1459, 1353, 1261, 1156, 1126, 1094, 708; **HRMS (ESI^{+})**: calculated for $[M+H]^+$ $C_{11}H_{211}F_3N^+$: 224.1621, found: 224.1613.

(*E*)-1,1,1-Trifluoro-N-methyldodec-3-en-7-yn-2-amine (12a)

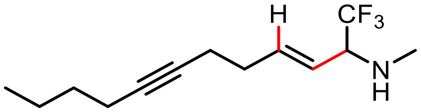

The title compound was isolated in 43% yield (21.0 mg) as a yellow oil.

^{1}H NMR (600 MHz, $CDCl_3$): δ 5.86 (dt, *J* = 15.3, 6.2 Hz, 1H), 5.36 (dd, *J* = 15.3, 8.3 Hz, 1H), 3.41 (p, *J* = 7.4 Hz, 1H), 2.45 (s, 3H), 2.29 – 2.25 (m, 4H), 2.15 – 2.11 (m, 2H), 1.47 – 1.42 (m, 2H), 1.41 – 1.36 (m, 2H), 0.90 (t, *J* = 7.2 Hz, 3H) ; **^{13}C NMR (151 MHz, $CDCl_3$)**: δ 136.9, 125.7 (q, *J* = 282 Hz), 123.6, 81.3, 78.9, 64.3 (q, *J* = 29 Hz), 34.1, 32.1, 31.3, 22.2, 18.8, 18.5, 13.7; **^{19}F NMR (565 MHz, $CDCl_3$)**: δ -75.2 (d, *J* = 7.2 Hz); **IR (neat, cm^{-1})**: 2932, 2864, 1455, 1365, 1259, 1180, 1156, 1106, 969, 695; **HRMS (ESI^+)**: calculated for $[M+H]^+$ $C_{13}H_{21}F_3N^+$: 248.1621, found: 248.1625.

Methyl (*E*)-4-(4,4,4-trifluoro-3-(methylamino)but-1-en-1-yl)benzoate (13a)

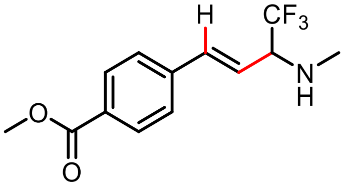

The title compound was isolated in 78% yield (36.0 mg) as a yellow oil.

^{1}H NMR (600 MHz, $CDCl_3$): δ 8.01 (d, *J* = 8.3 Hz, 2H), 7.46 (d, *J* = 8.3 Hz, 2H), 6.75 (d, *J* = 15.9 Hz, 1H), 6.14 (dd, *J* = 15.9, 8.0 Hz, 1H), 3.91 (s, 3H), 3.67 – 3.62 (m, 1H), 2.51 (s, 3H); **^{13}C NMR (151 MHz, $CDCl_3$)**: δ 166.8, 140.2, 135.5, 130.1 (2C), 130.0, 126.7 (2C), 125.5 (q, *J* = 282 Hz), 124.3, 64.4 (q, *J* = 29 Hz), 52.3, 34.3; **^{19}F NMR (565 MHz, $CDCl_3$)**: δ -74.6 (d, *J* = 7.1 Hz); **IR (neat, cm^{-1})**: 2954, 1715, 1608, 1436, 1277, 1156, 1102, 971, 762; **HRMS (ESI^+)**: calculated for $[M+H]^+$ $C_{13}H_{15}F_3NO_2^+$: 274.1049, found: 274.1043.

(*E*)-2-(8,8,8-Trifluoro-7-(methylamino)oct-5-en-1-yl)isoindoline-1,3-dione (14a)

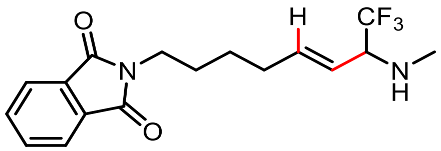

The title compound was isolated in 38% yield (26.0 mg) as a yellow oil.

^{1}H NMR (600 MHz, CDCl$_3$): δ 7.86 – 7.81 (m, 3H), 7.73 – 7.68 (m, 2H), 5.78 (dt, *J* = 15.4, 6.8 Hz, 1H), 5.33 – 5.27 (m, 1H), 3.69 (t, *J* = 7.2 Hz, 2H), 3.40 – 3.55 (m, 1H), 2.43 (s, 3H), 2.20 – 2.09 (m, 2H), 1.76 – 1.63 (m, 2H), 1.52 – 1.41 (m, 2H); **^{13}C NMR (151MHz, CDCl$_3$)**: δ 168.6 (2C), 137.9, 134.0 (2C), 132.3 (2C), 125.7 (q, *J* = 282 Hz), 123.3 (2C), 123.1, 64.3 (q, *J* = 29 Hz), 37.8, 34.2, 32.0, 28.1, 26.2; **^{19}F NMR (565 MHz, CDCl$_3$)**: δ -75.2 (d, *J* = 7.2 Hz); **IR (neat, cm^{-1})**: 2939, 2861, 1771, 1706, 1396, 1369, 1264, 1154, 1130, 1102, 923, 718; **HRMS (ESI$^+$)**: calculated for [M+H]$^+$ $C_{17}H_{20}F_3N_2O_2^+$: 341.1471, found: 341.1473.

***N*^{1}-(7-chloroquinolin-4-yl)-5,5,5-trifluoro-*N*^{4}-methylpentane-1,4-diamine (15a)**

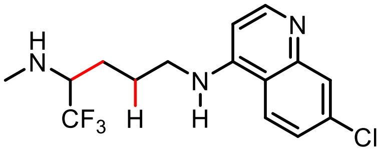

The title compound was isolated in 61% yield as a yellow oil (40.0 mg, 71% based on recovery of starting material). Reaction was performed at 75 °C.

^{1}H NMR (600 MHz, CDCl$_3$): δ 8.52 – 8.51 (m, 1H), 7.98 – 7.97 (m, 1H), 7.69 – 7.68 (m, 1H), 7.38 – 7.37 (m, 1H), 6.41 – 6.40 (m, 1H), 5.68 (br s, 1H), 3.38 – 3.35 (m, 2H), 3.02 (dd, $J_{H,H}$ = 9.8 Hz, 3.7 Hz; q, $J_{H,F}$ = 7.4 Hz, 1H), 2.57 (s, 3H), 2.00 – 1.94 (m, 2H), 1.90 (dddd, *J* = 14.1 Hz, 9.0 Hz, 6.9 Hz, 3.7 Hz, 1H), 1.64 (dddd, *J* = 14.0 Hz, 9.8 Hz, 8.4 Hz, 5.6 Hz, 1H); **^{13}C NMR (151 MHz, CDCl$_3$):** δ 151.5, 150.0, 148.5, 135.2, 128.4, 126.9 (*J* = 286 Hz), 125.5, 121.0, 117.1, 99.0, 60.3 (*J* = 26.7 Hz), 43.2, 34.1, 26.1, 25.0; **IR (neat, cm^{-1}):** 3176, 2927, 2885, 2368, 2341, 1621; 1509; 1329; 1227; 1104; **^{19}F NMR (565 MHz, CDCl$_3$):** δ -73.84 (d, *J* = 7.0 Hz); **HRMS (ESI$^+$):** calculated for [M+H]$^+$ $C_{15}H_{18}ClN_3F_3^+$: 332.1136, found 332.1137.

4-(4-chlorophenyl)-1,1,1-trifluoro-*N,N*-dimethyl-4-(pyridin-2-yl)butan-2-amine (16a)

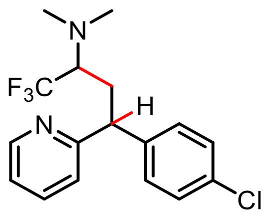

The title compound was isolated in 61% yield as a yellow oil (41.0 mg, 1:1 diastereomeric ratio). Reaction was performed at 75 °C.

^{1}H NMR (600 MHz, $CDCl_3$): δ 8.60 – 8.57 (m, 1H), 7.59 – 7.54 (m, 1H), 7.32 – 7.28 (m, 3H), 7.25 – 7.22 (m, 1H), 7.16 (m, d2, 0.5H), 7.13 – 7.10 (m, 1.5H), 4.32 – 4.30 (m, 2H), 2.77 (dd, *J* = 10.8 Hz, 4.0 Hz, 0.5H, d2), 2.70 (dd, 11.1 Hz, 3.7 Hz, 0.5H, d1), 2.66 – 2.62 (m, 0.5H, d2), 2.45 – 2.43 (m, 0.5H, d1), 2.41 (s, 3H, d1), 2.38 (s, 3H, d2), 2.36 – 2.34 (m, 0.5H, d1), 2.17 – 2.12 (m, 0.5H, d2); **^{13}C NMR (151 MHz, $CDCl_3$):** δ 159.7, 162.7 (d1), 161.7 (d2), 149.7 (d2), 149.3 (d1), 142.2 (d2), 140.9 (d1), 136.5 (d1, d2), 131.4, 132.6 (d1), 132.4 (d2), 129.8 (d1), 129.3 (d2), 128.8 (d1), 128.6 (d2)123.6 (d2), 122.9 (d1), 121.7 (d2), 121.6 (d1), 62.4 (q, *J* = 24.7 Hz), 62.2 (q, *J* = 24.7 Hz), 48.4 (d1), 48.2 (d2), 40.9 (d2), 40.9 (d1), 31.4 (d2), 31.1 (d1); **^{19}F NMR (565 MHz, $CDCl_3$):** δ -68.08 (d, *J* = 7.7 Hz, d1), -68.29 (d, *J* = 7.7 Hz, d2); **HRMS (ESI$^+$):** calculated for $[M+H]^+$ $C_{17}H_{19}ClN_2F_3^+$: 343.1183, found 343.1186.

(*1R*)-(6-methoxyquinolin-4-yl)((1S,2R,4S,5R)-5-(4,4,4-trifluoro-3-(methylamino)butyl)quinuclidin-2-yl)methanol (17a)

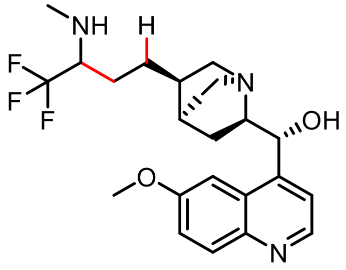

The title compound was isolated in 32% yield as a yellow solid (28.0 mg, 76% based on recovery of starting material). Reaction was performed at 75 °C. Diastereoisomers were not clearly visible by **^{1}H-NMR** or **^{13}C-NMR**. Diasteriomeric ratio was calculated using crude ^{19}F-NMR – 1.5 to 1 ratio d1/d2.

^{1}H NMR (600 MHz, MeOD): δ 8.66 (d, *J* = 4.6 Hz, 1H), 7.97 – 7.91 (m, 1H), 7.70 (d, *J* = 4.6 Hz, 1H), 7.42 (dd, *J* = 7.8, 2.4 Hz, 2H), 5.62 (s, 1H), 4.72 (s, 1H), 4.59 (br s, OH), 4.02 – 3.93 (m, 3H), 3.73 (s, 1H), 3.20 – 3.15 (m, 2H), 2.87 (dtd, *J* = 11.6, 7.9, 3.8 Hz, 1H), 2.75 (td, *J* = 12.0, 4.9 Hz, 1H), 2.51 – 2.41 (m, 1H), 2.34

(s, 3H), 1.98– 1.97 (m, 2H), 1.81 – 1.80 (m, 1H), 1.66 (br s, 1H), 1.61 – 1.52 (m, 2H), 1.51 - 1.42 (m, 2H), 1.41 – 1.27 (m, 2H); **^{13}C NMR (151 MHz, MeOD)**: δ 159.7, 150.3, 148.2, 144.8, 131.4, 128.4 (*J* = 282 Hz) 128.1, 120.1, 102.5, 74.4, 71.9, 61.9 (*J* = 27 Hz, confor), 61.0 (d1), 60.9 (d2), 59.4 (d2), 59.2 (d1), 58.9 (d1), 58.8 (d2), 56.5, 44.3, 36.5 (d2), 36.4 (d1), 34.8 (d2), 34.7 (d1), 31.6, 31.4 (confor), 28.4 (d2) , 28.4 (d1), 27.3 (d2), 27.3 (d1), 27.2, 26.8, 21.2; **^{19}F NMR (565 MHz, $CDCl_3$):** δ -75.4 (d, *J* = 7.9 Hz, 1.5H, d1), -74.6 (d, *J* = 7.9 Hz, d2); **IR (neat, cm^{-1}):** 3176, 2927, 2885, 2368, 2341, 1621; 1509; 1329; 1227; 1104; **HRMS (ESI^+):** calculated for $[M+H]^+$ $C_{23}H_{31}N_3O_2F_3^+$: 438.2363, found 438.2367.

4.2. α – aminoesters

Ethyl 2-(methylamino)-4-phenylbutanoate (1b)

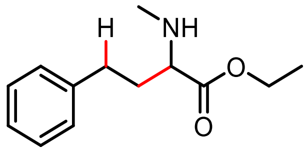

The title compound was isolated in 39% yield (17.0 mg) as a yellow oil. Reaction was conducted at rt.

^{1}H NMR (600 MHz, $CDCl_3$): δ 7.30 – 7.24 (m, 2H), 7.21 – 7.17 (m, 3H), 4.20 (q, *J* = 7.0 Hz, 2H), 3.15 (t, *J* = 6.8 Hz, 1H), 2.75 – 2.65 (m, 2H), 2.38 (s, 3H), 2.01 – 1.94 (m, 1H), 1.91 – 1.84 (m, 1H), 1.29 (t, *J* = 7.0 Hz, 3H); **^{13}C NMR (151 MHz, $CDCl_3$)** δ 175.2, 141.4, 128.4 (2C), 128.3 (2C), 125.9, 62.7, 60.6, 34.9, 34.7, 32.0, 14.4; **IR (neat, cm^{-1}):** 2980, 1739, 1663, 1203, 1028, 751, 701; **HRMS (ESI$^+$):** calculated for $[M+H]^+$ $C_{13}H_{20}NO_2^+$: 222.1489, found 222.1487.

ethyl 2-(methylamino)-4-(p-tolyl)butanoate (2b)

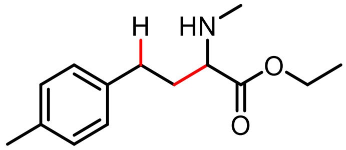

The title compound was isolated in 41% yield (19.0 mg) as a yellow oil.

^{1}H NMR (600 MHz, $CDCl_3$): δ 7.10 - 7.07 (m, 4H), 4.20 (q, *J* = 6.5 Hz, 2H), 3.15 (t, *J* = 6.6 Hz, 1H), 2.70 – 2.61 (m, 2H), 2.38 (s, 3H), 2.31 (s, 3H), 1.98 - 1.91 (m, 1H), 1.90 - 1.83 (m, 1H), 1.54 (br s, 1H), 1.30 – 1.25 (m, 3H); **^{13}C NMR (151 MHz, $CDCl_3$):** δ 175.4, 138.5, 135.6, 129.2 (2C), 128.5 (2C), 62.9, 60.8, 35.2, 34.9, 31.7, 21.1, 14.5; **IR (neat, cm^{-1})**: 2980, 2933, 2860, 1731, 1516, 1180, 1037; **HRMS (ESI+):** calculated for $[M+H]^+$ $C_{14}H_{22}NO_2^+$: 236.1645, found 236.1644.

ethyl 4-(4-bromophenyl)-2-(methylamino)butanoate (3b)

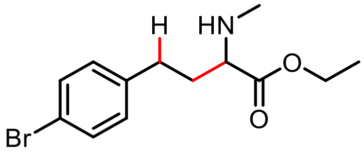

The title compound was isolated in 45% yield (27.0 mg) as a yellow oil.

^{1}H NMR (600 MHz, $CDCl_3$): δ 7.40 (d, *J* = 8.7 Hz, 2H), 7.06 (d, *J* = 8.3 Hz, 2H), 4.19 (q, *J* =7.2 Hz, 2H), 3.10 (t, *J* = 6.8 Hz, 1H), 2.70 - 2.61 (m, 2H), 2.37 (s, 3H), 1.97 - 1.89 (m, 1H), 1.88 - 1.80 (m, 1H), 1.29 (t, *J* = 7.2 Hz, 3H); **^{13}C NMR (151 MHz, $CDCl_3$):** δ 175.2, 140.5, 131.6 (2C), 130.4 (2C), 119.9, 62.6, 60.8, 34.9, 34.8, 31.6, 14.5; **IR (neat, cm^{-1})**: 2978, 2931, 2856, 2799, 1729, 1645, 1487, 1180, 1072, 940, 765.; **HRMS (ESI^+):** calculated for $[M+H]^+$ $C_{13}H_{19}{}^{79}BrNO_2{}^+$: 300.0594, found 300.0591.

ethyl 2-(methylamino)undecanoate (4b)

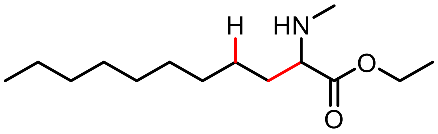

The title compound was isolated in 59% yield (28.0 mg) as a yellow oil.

^{1}H NMR (600 MHz, $CDCl_3$): δ 4.20 (q, *J* = 7.2 Hz, 2H), 3.12 (t, *J* = 6.8 Hz, 1H), 2.37 (s, 3H), 1.66 - 1.53 (m, 2H), 1.37 - 1.21 (m, 17H), 0.87 (t, *J* = 7.0 Hz, 3H); **^{13}C NMR (151 MHz, $CDCl_3$):** δ 175.6, 63.5, 60.6, 34.9, 33.6, 32.0, 30.0, 29.6, 29.5, 29.6, 25.9, 22.8, 14.5, 14.2; **IR (neat, cm^{-1}):** 2924, 2855, 1733, 1608, 1370, 1339; **HRMS (ESI^+):** calculated for $[M+H]^+$ $C_{14}H_{30}NO_2{}^+$: 244.2271, found 244.2269.

ethyl 12-hydroxy-2-(methylamino)dodecanoate (5b)

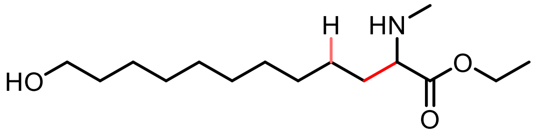

The title compound was isolated in 54% yield (29.0 mg) as a yellow oil.

^{1}H NMR (600 MHz, $CDCl_3$): δ 4.20 (q, *J* = 6.9 Hz, 2H), 3.63 (t, *J* = 6.5 Hz, 2H), 3.12 (t, *J* = 6.7 Hz, 1H), 2.36 (s, 3H), 1.64 - 1.53 (m, 5H), 1.39 - 1.23 (m, 18H); **^{13}C NMR (151 MHz, $CDCl_3$):** δ 175.6, 63.5, 63.2, 60.6, 34.9, 33.6 (2C), 32.9, 29.7, 29.6, 29.5, 29.4, 25.9, 25.8, 14.5; **IR (neat, cm^{-1}):** 2928, 2854, 1731, 1452, 1182, 1110.; **HRMS (ESI$^+$):** calculated for $[M+H]^+$ $(C_{15}H_{32}NO_3)^+$: 274.2377, found 274.2375.

Ethyl 2-(methylamino)-6-phenylhexanoate (6b)

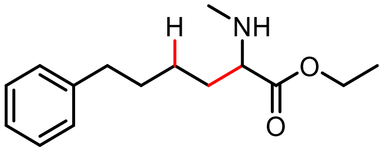

The title compound was isolated in 63% yield (32.0 mg) as a yellow oil. Reaction was conducted at rt.

^{1}H NMR (600 MHz, $CDCl_3$): δ 7.28 – 7.26 (m, 1H), 7.25 – 7.24 (m, 2H), 7.21 – 7.14 (m, 3H), 4.18 (q, *J* = 7.1 Hz, 2H), 3.12 (t, *J* = 6.7 Hz, 1H), 2.60 (t, *J* = 7.9 Hz, 2H), 2.36 (s, 3H), 1.70 – 1.58 (m, 4H), 1.46 – 1.34 (m, 2H), 1.26 (t, *J* = 7.1 Hz, 3H); **^{13}C NMR (151 MHz, $CDCl_3$):** δ 175.4, 142.4, 128.4 (2C), 128.3 (2C), 125.7, 63.2, 60.5, 35.7, 34.8, 33.3, 31.3, 25.4, 14.3; **IR (neat, cm^{-1}):** 2978, 1730, 1495, 1179, 1028, 749, 699; **HRMS (ESI$^+$):** calculated for $[M+H]^+$ $C_{15}H_{24}NO_2$+: 250.1802, found 250.1800.

1-ethyl 8-methyl 2-(methylamino)octanedioate (7b)

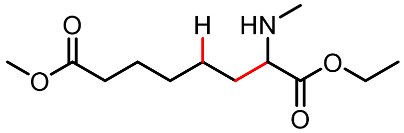

The title compound was isolated in 70% yield (34.0 mg) as a yellow oil.

^{1}H NMR (600 MHz, CDCl$_3$): δ 4.19 (q, *J* = 7.2 Hz, 2H), 3.66 (s, 3H), 3.11 (t, *J* = 6.8 Hz, 1H), 2.36 (s, 3H), 2.30 (t, *J* = 7.5 Hz, 2H), 1.67 - 1.54 (m, 4H), 1.43 - 1.31 (m, 4H), 1.28 (t, *J* = 7.2 Hz, 3H); **^{13}C NMR (151 MHz, CDCl3):** δ 175.5, 174.3, 63.4, 60.7, 51.6, 34.9, 34.1, 33.3, 29.1, 25.6, 24.9, 14.5; **IR (neat, cm^{-1}):** 2938, 2860, 1732, 1440, 1178, 1027; **HRMS (ESI$^+$):** calculated for $[M+H]^+$ $C_{12}H_{24}NO_4^+$: 246.1700, found 246.1697.

ethyl 6-acetoxy-2-(methylamino)hexanoate (8b)

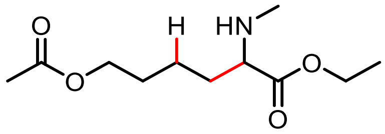

The title compound was isolated in 38% yield (18.0 mg) as a yellow oil.

^{1}H NMR (600 MHz, CDCl$_3$): δ 4.20 (q, *J* = 7.2 Hz, 2H), 4.05 (t, *J* = 6.8 Hz, 2H), 3.12 (t, *J* = 6.6 Hz, 1H), 2.37 (s, 3H), 2.04 (s, 3H), 1.63 (d, *J* = 7.2 Hz, 5H), 1.49 - 1.37 (m, 2H), 1.29 (t, *J* = 7.2 Hz, 3H); **^{13}C NMR (151 MHz, CDCl$_3$):** δ 175.4, 171.3, 64.4, 63.3, 60.7, 34.9, 33.1, 28.6, 22.5, 21.1, 14.5; **IR (neat, cm^{-1}):** 3454, 2985, 2945, 2880, 1734, 1275, 1259, 1135, 1102; **HRMS (ESI$^+$):** calculated for $[M+H]^+$ $C_{11}H_{22}NO_4^+$: 232.1543, found 232.1540.

ethyl 10-bromo-2-(methylamino)decanoate (9b)

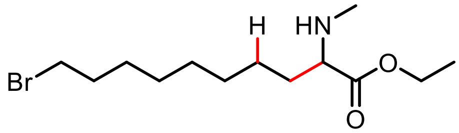

The title compound was isolated in 62% yield (38.0 mg) as a yellow oil.

^{1}H NMR (600 MHz, $CDCl_3$) δ 4.20 (q, *J* = 6.7 Hz, 2H), 3.40 (t, *J* = 6.8 Hz, 2H), 3.12 (t, *J* = 6.6 Hz, 1H), 2.37 (s, 3H), 1.84 (quin, *J* = 7.2 Hz, 2H), 1.66 – 1.56 (m, 2H), 1.57 - 1.50 (m, 2H), 1.41 - 1.28 (m, 12H); **^{13}C NMR (151 MHz, $CDCl_3$):** δ 175.6, 63.5, 60.6, 34.9, 34.1, 33.5, 32.9, 29.5, 29.4, 28.8, 28.3, 25.9, 14.5; **IR (neat, cm^{-1}):** 3419, 2927, 2854, 1737, 1464, 1371, 1213, 1158, 1021; **HRMS (ESI^+):** calculated for $[M+H]^+$ $C_{13}H_{27}BrNO_2^+$: 308.1220, found 308.1216.

ethyl 2-(methylamino)-10-(phenylsulfonyl)decanoate (10b)

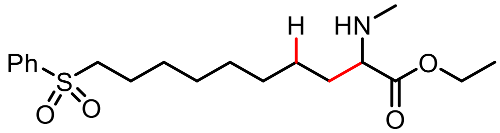

The title compound was isolated in 67% yield (50.0 mg) as a yellow oil.

^{1}H NMR (600 MHz, $CDCl_3$): δ 7.91 (d, *J* = 7.5 Hz, 2H), 7.65 (d, *J* = 7.2 Hz, 1H), 7.61 - 7.49 (m, 2H), 4.19 (q, *J* = 6.9 Hz, 2H), 3.13 - 3.01 (m, 3H), 2.36 (s, 3H), 1.69 (dt, *J* = 7.4, 4.0 Hz, 2H), 1.63 - 1.48 (m, 4H), 1.39 - 1.18 (m, 12H); **^{13}C NMR (151 MHz, $CDCl_3$):** δ 175.5, 139.4, 133.7, 129.4 (2C), 128.2 (2C), 63.4, 60.6, 56.4, 34.9, 33.5, 29.4, 29.2, 29.0, 28.4, 25.8, 22.8, 14.5; **IR (neat, cm^{-1})**: 2929, 2855, 1729, 1447, 1304, 1180, 1146, 1025, 749, 690; **HRMS (ESI+):** calculated for $[M+H]^+$ $C_{19}H_{32}NO_4S^+$: 370.2047, found 270.2044.

ethyl 2-(methylamino)-11-oxo-11-(pyrrolidin-1-yl)undecanoate (11b)

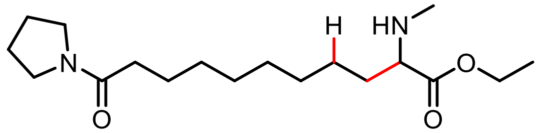

The title compound was isolated in 57% yield (37.0 mg) as a yellow oil.

^{1}H NMR (600 MHz, $CDCl_3$): δ 4.19 (q, *J* = 6.9 Hz, 2H), 3.45 (t, *J* = 7.0 Hz, 2H), 3.40 (t, *J* = 7.0 Hz, 2H), 3.11 (t, *J* = 6.8 Hz, 1H), 2.36 (s, 3H), 2.24 (t, *J* = 7.7 Hz, 2H), 1.94 (p, *J* = 6.8 Hz, 2H), 1.84 (p, *J* = 6.9 Hz, 2H), 1.68 - 1.48 (m, 6H), 1.38 - 1.18 (m, 12H); **^{13}C NMR (151 MHz, $CDCl_3$):** δ 175.6, 172.0, 63.5, 60.6, 46.7, 45.7, 35.0, 34.9, 33.6, 29.7, 29.6, 29.5, 29.4, 26.3, 25.9, 25.1, 24.6, 14.5; **IR (neat, cm^{-1})**: 2924, 2854, 1730, 1641, 1432, 1179, 1029; **HRMS (ESI$^+$):** calculated for $[M+H]^+$ $C_{18}H_{35}N_2O_3^+$: 327.2642, found 327.2640.

ethyl 7-hydroxy-2-(methylamino)heptanoate (12b)

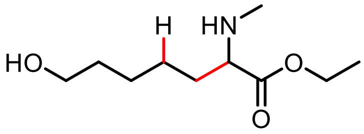

The title compound was isolated in 36% yield (15.0 mg) as a yellow oil.

^{1}H NMR (600 MHz, $CDCl_3$): δ 4.20 (q, *J* = 7.2 Hz, 2H), 3.63 (t, *J* = 6.6 Hz, 2H), 3.13 (t, *J* = 6.6 Hz, 1H), 2.37 (s, 3H), 1.70 - 1.53 (m, 5H), 1.44 - 1.33 (m, 5H), 1.29 (t, *J* = 7.2 Hz, 3H); **^{13}C NMR (151 MHz, $CDCl_3$):** δ 175.5, 63.3, 62.9, 60.7, 34.9, 33.4, 32.6, 25.7, 25.6, 14.5; **IR (neat, cm^{-1})**: 3396, 2940, 2868, 1764 1656, 1457, 1198, 1058, 963; **HRMS (ESI+):** calculated for $[M+H]^+$ $C_{10}H_{22}NO_3^+$: 204.1594, found 204.1593.

ethyl 10-(1,3-dioxoisoindolin-2-yl)-2-(methylamino)decanoate (13b)

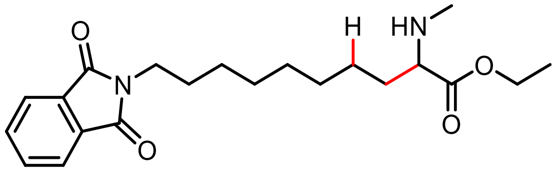

The title compound was isolated in 56% yield (42.0 mg) as an orange oil.

^{1}H NMR (600 MHz, $CDCl_3$): δ 7.83 (dd, *J* = 5.3, 3.0 Hz, 2H), 7.70 (dd, *J* = 5.3, 3.0 Hz, 2H), 4.19 (q, *J* = 6.7 Hz, 2H), 3.66 (t, *J* = 7.3 Hz, 2H), 3.10 (t, *J* = 6.6 Hz, 1H), 2.39 - 2.32 (m, 3H), 1.69 - 1.62 (m, 2H), 1.62 - 1.49 (m,

4H), 1.36 - 1.21 (m, 12H); **^{13}C NMR (151 MHz, CDCl$_3$):** δ 175.6, 168.6, 134.0, 132.3, 123.3, 63.5, 60.6, 38.2, 34.9, 33.5, 29.5, 29.4, 29.2, 28.7, 26.9, 25.8, 14.5; **IR (neat, cm^{-1})**: 2932, 2857, 1773, 1713, 1438, 1180, 764, 721.; **HRMS (ESI$^+$):** calculated for [M+H]$^+$ $C_{21}H_{31}N_2O_4^+$: 375.2278, found 375.2275.

ethyl 10-azido-2-(methylamino)decanoate (14b)

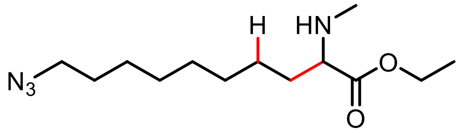

The title compound was isolated in 50% yield (27.0 mg) as a yellow oil.

^{1}H NMR (600 MHz, CDCl$_3$): δ 4.20 (q, J = 6.9 Hz, 2H), 3.25 (t, J = 7.0 Hz, 2H), 3.12 (t, J = 6.6 Hz, 1H), 2.37 (s, 3H), 1.66 - 1.54 (m, 4H), 1.34 (dd, J = 13.2, 6.0 Hz, 4H), 1.32 - 1.23 (m, 10H); **^{13}C NMR (151 MHz, CDCl$_3$):** δ 175.6, 63.5, 60.6, 51.6, 34.9, 33.5, 29.5, 29.4, 29.2, 29.0, 26.8, 25.9, 14.5; **IR(neat, cm^{-1})**: 2931, 2856, 2154, 2095, 1731, 1455, 1180, 1028; **HRMS (ESI$^+$):** calculated for [M+H]$^+$ $C_{13}H_{27}N_4O_2^+$: 271.2129, found 271.2128.

ethyl 2-cycloheptyl-2-(methylamino)acetate (15b)

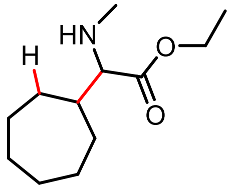

The title compound was isolated in 34% yield (15.0 mg) as a yellow oil.

^{1}H NMR (600 MHz, CDCl$_3$): δ 4.20 (m, 2H), 2.93 (d, J = 5.6 Hz, 1H), 2.35 (s, 3H), 1.76 – 1.73 (m, 2H), 1.68 – 1.65 (m, 3H), 1.57 – 1.54 (m, 4H), 1.48 – 1.44 (m, , 2H), 1.39 - 1.32 (m, 3H), 1.29 (t, J = 7.2 Hz, 3H); **^{13}C NMR (151 MHz, CDCl$_3$):** δ 175.2, 69.5, 60.5, 43.0, 35.5, 31.5, 30.2, 28.6, 28.1, 27.0, 26.9, 14.6; **IR (neat, cm^{-1}):** 2922, 2855, 1729, 1453, 1178, 1105; **HRMS (ESI$^+$):** calculated for [M+H]$^+$ $C_{12}H_{24}NO_2^+$: 214.1802, found 214.1800.

ethyl 2-(benzylamino)-4-phenylbutanoate (16b)

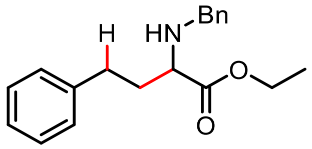

The title compound was isolated in 30% yield (18.0 mg) as a yellow oil.

^{1}H NMR (600 MHz, $CDCl_3$): δ 7.36 - 7.30 (m, 4H), 7.28 – 7.27 (br s, 2H), 7.26 - 7.24 (m, 1H), 7.20 - 7.15 (m, 3H), 4.19 (qd, *J* = 7.2, 1.7 Hz, 2H), 3.83 (d, *J* = 12.9 Hz, 1H), 3.63 (d, *J* = 12.9 Hz, 1H), 3.27 (dd, *J* = 7.5, 5.8 Hz, 1H), 2.80 - 2.74 (m, 1H), 2.73 - 2.64 (m, 1H), 2.02 - 1.95 (m, 1H), 1.93 - 1.86 (m, 1H), 1.29 (t, *J* = 7.1 Hz, 3 H); **^{13}C NMR (151 MHz, $CDCl_3$):** δ 175.5, 141.7, 140.1, 128.6 (2C), 128.5 (4C), 128.4 (2C), 127.2, 126.1, 60.8, 60.4, 52.3, 35.3, 32.2, 14.5; **IR (neat, cm^{-1}):** 3027, 2932, 2854, 1729, 1454, 1132, 1027, 749, 698.; **HRMS (ESI$^+$):** calculated for $[M+H]^+$ $C_{19}H_{24}NO_2^+$: 298.1802, found 298.1799.

4.3. α – aminophosphonates

Diethyl (1-(methylamino)-3-phenylpropyl)phosphonate (1c)

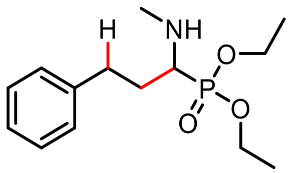

The title compound was isolated in 34% yield (19.0 mg) as a yellow oil. Reaction conducted at -15°C.

^{1}H NMR (600 MHz, CDCl$_3$): δ 7.32 – 7.25 (m, 2H), 7.23 – 7.16 (m, 3H), 4.16 – 4.09 (m, 4H), 2.90 – 2.83 (m, 1H), 2.78 – 2.70 (m, 2H), 2.52 (d, $^4J_{H,P}$ = 1.3 Hz, 3H), 2.13 – 2.03 (m, 1H), 1.93 – 1.83 (m, 1H), 1.34 – 1.30 (m, 6H); **^{13}C NMR (151 MHz, CDCl$_3$):** δ 141.6, 128.5 (2C), 128.4 (2C), 125.9, 61.9 (d, $^2J_{C,P}$ = 7.3 Hz), 61.8 (d, $^2J_{C,P}$ = 7.3 Hz), 55.9 (d, $^1J_{C,P}$ = 146.6 Hz), 35.1 (d, $^3J_{C,P}$ = 6.2 Hz), 32.2 (d, $^3J_{C,P}$ = 11.2 Hz), 31.1 (d, $^4J_{C,P}$ = 2.7 Hz), 16.5 (d, $^3J_{C,P}$ = 5.4 Hz, 2C); **^{31}P NMR (243 MHz, CDCl$_3$):** δ 28.6; **IR (neat, cm^{-1})**: 3393, 2982, 1645, 1152, 1022, 967, 801; **HRMS (ESI$^+$):** calculated for $[M+H]^+$ $C_{14}H_{25}NO_3P^+$: 286.1567, found 286.1560.

Diethyl (1-(methylamino)-3-phenylpropyl)phosphonate (2c)

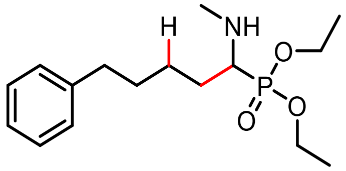

The title compound was isolated in 52% yield (32.0 mg) as a yellow oil.

^{1}H NMR (600 MHz, CDCl$_3$): δ 7.31 – 7.23 (m, 2H), 7.21 – 7.13 (m, 3H), 4.18 – 4.08 (m, 4H), 2.75 – 2.68 (m, 1H), 2.67 – 2.58 (m, 2H), 2.51 (d, $^4J_{H,P}$ = 1.3 Hz, 3H), 1.86 – 1.75 (m, 1H), 1.69 – 1.54 (m, 4H), 1.50 – 1.38 (m, 2H), 1.32 (td, J = 7.2, $^4J_{H,P}$ = 1.1 Hz, 6H); **^{13}C NMR (151 MHz, CDCl$_3$):** δ 142.5, 128.4 (2C), 128.3 (2C), 125.7, 61.9 (d, $^2J_{C,P}$ = 7.2 Hz), 61.8 (d, $^2J_{C,P}$ = 7.1 Hz), 56.8 (d, $^1J_{C,P}$ = 148.4 Hz), 35.8, 35.3 (d, $^3J_{C,P}$ = 6.9 Hz), 31.4, 29.3 (d, $^4J_{C,P}$ = 1.6 Hz), 25.9 (d, $^3J_{C,P}$ = 10.6 Hz), 16.5 (dd, $^3J_{C,P}$ = 5.6, 1.5 Hz, 2C); **^{31}P NMR (243 MHz, CDCl$_3$):** δ 28.6; **IR (neat, cm^{-1})**: 3463, 2983, 2933, 1676, 1602, 1152, 1024, 749; **HRMS (ESI$^+$):** calculated for $[M+H]^+$ $C_{16}H_{29}NO_3P^+$: 314.1880, found 314.1879.

diethyl (1-(methylamino)decyl)phosphonate (3c)

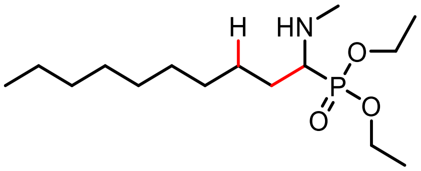

The title compound was isolated in 50% yield (30.0 mg) as a yellow oil.

^{1}H NMR (600 MHz, CDCl$_3$): δ 4.19 - 4.08 (m, 4H), 2.73 – 2.69 (m, 1H), 2.51 (s, 3H), 1.75 (ddd, *J* = 18.5, 8.6, 4.9 Hz, 1H), 1.60 - 1.45 (m, 2H), 1.32 (t, *J* = 7.2 Hz, $^{4}J_{H,P}$ = 1.1 Hz, 6H), 1.30 - 1.20 (m, 12H), 1.14 (bs, 1H), 0.87 (t, *J* = 7.0 Hz, 3H); **^{13}C NMR (125 MHz, CDCl$_3$):** δ 61.9 (d, $^{2}J_{C,P}$ = 7.1 Hz), 61.8 (d, $^{2}J_{C,P}$ = 7.1 Hz), 57.0 (d, $^{1}J_{C,P}$ = 148.3 Hz), 35.5 (d, $^{3}J_{C,P}$ = 7.1 Hz), 32.0, 29.7, 29.6, 29.6, 29.5 (d, $^{4}J_{C,P}$ = 1.5 Hz), 29.4, 26.4 (d, $^{3}J_{C,P}$ = 10.7 Hz), 22.8, 16.7 (dd, $^{3}J_{C,P}$ = 5.6, 1.5 Hz, 2C), 14.2; **^{31}P NMR (243 MHz, CDCl$_3$):** δ 28.9; **IR (neat, cm^{-1})**: 3494, 2952, 2854, 1674, 1275, 1021, 957, 764; **HRMS (ESI$^+$):** calculated for $[M+H]^+$ $C_{15}H_{35}NO_3P^+$: 308.2349, found 308.2344.

diethyl (9-bromo-1-(methylamino)nonyl)phosphonate (4c)

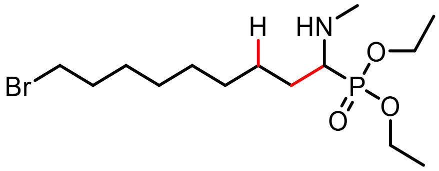

The title compound was isolated in 65% yield (48.0 mg) as a yellow oil.

^{1}H NMR (600 MHz, CDCl$_3$): δ 4.18 - 4.09 (m, 4H), 3.40 (t, *J* = 6.8 Hz, 2H), 2.71 (ddd, *J* = 12.4, 8.1, 4.7 Hz, 1H), 2.52 (s, 3H), 1.88 - 1.81 (m, 2H), 1.81 - 1.71 (m, 1H), 1.61 - 1.47 (m, 2H), 1.45 - 1.36 (m, 4H), 1.35 - 1.28 (m, 12H); **^{13}C NMR (125 MHz, CDCl$_3$):** δ 62.0 (d, $^{2}J_{C,P}$ = 7.2 Hz), 61.9 (d, $^{2}J_{C,P}$ = 7.2 Hz), 57.0 (d, $^{1}J_{C,P}$ = 148.3 Hz), 35.5 (d, $^{3}J_{C,P}$ = 6.7 Hz), 34.2, 32.9, 29.6, 29.5 (d, $^{4}J_{C,P}$ = 1.5 Hz), 29.4, 28.8, 28.3, 26.4 (d, $^{3}J_{C,P}$ = 10.8 Hz), 16.7 (dd, $^{3}J_{C,P}$ = 5.8, 1.7 Hz, 2C); **^{31}P NMR (243 MHz, CDCl$_3$):** δ 28.8; **IR (neat, cm^{-1})**: 3420, 2927, 2854, 1669, 1465, 1214, 1042, 1022, 947, 750; **HRMS (ESI$^+$):** calculated for $[M+H]^+$ $C_{14}H_{32}{}^{79}BrNO_3P^+$: 372.1298, found 372.1296.

4.4. α – trifluoromethylamines derived from mechanistic elucidation

(*Z*)-4-Chloro-6-(dimethylamino)-7,7,7-trifluorohept-4-en-1-ol (4d)

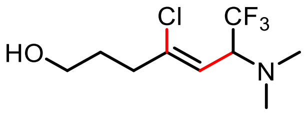

The title compound was isolated in 55% yield (27.0 mg) as a yellow oil.

^{1}H NMR (600 MHz, $CDCl_3$): δ 5.85 (d, *J* = 9.0 Hz, 1H), 3.84 (dq, *J* = 8.5, 7.9 Hz, 1H), 3.71 (br s, 1s), 3.60 (dt, *J* = 11.0, 4.6 Hz, 1H), 3.52 (ddd, *J* = 11.5, 9.4, 3.9 Hz, 1H), 2.69 (ddd, *J* = 17.2, 9.4, 6.1 Hz, 1H), 2.46 (s, 6H), 2.43 – 2.39 (m, 1H), 1.91 – 1.80 (m, 2H); **^{13}C NMR (151 MHz, $CDCl_3$)**: δ 142.8, 126.3 (q, *J* = 289 Hz), 119.9 , 62.7 (q, *J* = 27 Hz), 59.4, 41.3, 41.3, 30.0, 29.2; **^{19}F NMR (659 MHz, $CDCl_3$)**: δ -67.9 (d, *J* = 7.6 Hz) ; **IR (neat, cm^{-1})**: 3386, 2946, 1648, 1459, 1373, 1254, 1157, 1110, 1037, 984, 657; **HRMS (ESI^+)**: calculated for $[M+H]^+$ $C_9H_{16}ClF_3NO^+$: 246.0867, found: 246.0855.

(*Z*)-3-(3,4-Dihydronaphthalen-1(2H)-ylidene)-1,1,1-trifluoro-*N*,*N*-dimethylpropan-2-amine (7d)

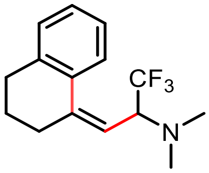

The title compound was isolated in 74% yield (33.0 mg) as a yellow oil.

^{1}H NMR (600 MHz, $CDCl_3$): δ 7.66 – 7.60 (m, 1H), 7.25 – 7.18 (m, 2H), 7.15 – 7.11 (m, 1H), 6.05 (d, *J* = 9.8 Hz, 1H), 3.95 (dq, *J* = 9.8, 8.1 Hz, 1H), 2.84 (t, *J* = 6.3 Hz, 2H), 2.61 – 2.56 (m, 2H), 2.46 (s, 6H), 1.92 – 1.86 (m, 2H) ; **^{13}C NMR (151 MHz, $CDCl_3$)**: δ 143.0, 138.1, 135.2, 129.2, 128.1, 126.4 (d, *J* = 289 Hz), 126.3, 124.5, 112.6, 62.8 (q, *J* = 28 Hz), 42.4, 42.4, 30.3, 27.2, 23.4; **^{19}F NMR (659 MHz, $CDCl_3$)**: δ -70.5 (d, *J* = 8 Hz); **IR (neat, cm^{-1})**: 2939, 2870, 1456, 1260, 1152, 1103, 1033, 754; **HRMS (ESI^+)**: calculated for $[M-NMe_2]^+$ $C_{13}H_{12}F_3^+$: 225.0886, found: 225.0896;

Ethyl 6-(dimethylamino)-7,7,7-trifluoro-4-oxoheptanoate (11d)

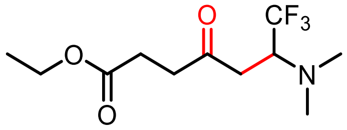

The title compound was isolated in 59% yield (34.0 mg) as a yellow oil.

^{1}H NMR (700 MHz, $CDCl_3$): δ 4.12 (q, *J* = 7.1 Hz, 2H), 3.76 – 3.74 (m, 1H), 2.81 (dd, *J* = 16.7, 8.6 Hz, 1H), 2.77 (q, *J* = 6.5 Hz, 2H), 2.63 – 2.58 (m, 3H), 2.43 – 2.39 (m, 6H), 1.24 (t, *J* = 7.1 Hz, 3H); **^{13}C NMR (175 MHz, $CDCl_3$)**: δ 205.0, 172.7, 127.1 (q, *J* = 290 Hz), 60.9, 60.7 (q, *J* = 26 Hz), 41.6 (2C), 39.1, 37.9, 28.1, 14.3; **^{19}F NMR (660 MHz, $CDCl_3$)**: δ -69.6 (d, *J* = 8 Hz); **IR (neat, cm^{-1})**: 2986, 1722, 1374, 1300, 1262, 1187, 1166, 1150, 1093, 1030. **HRMS (ESI^+)**: calculated for $[M+Na]^+$ $C_{11}H_{19}F_3NNaO_3^+$: 292.1131, found: 292.1139;

4.5. Direct synthesis of Homophenylalanine (17b) from corresponding alkene

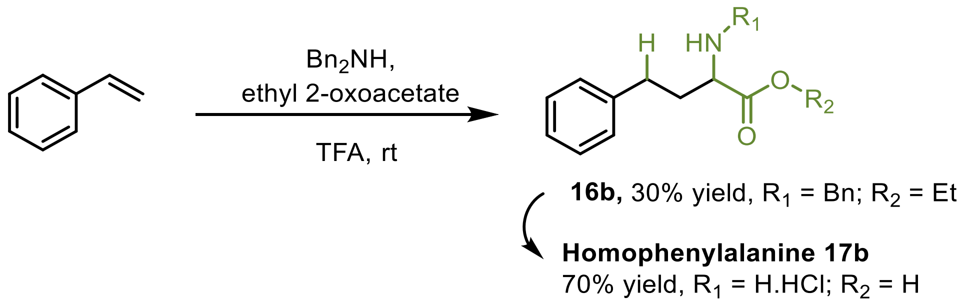

Compound **ethyl 2-(benzylamino)-4-phenylbutanoate (16b)** (21.0 mg, 0.07 mmol, 1 equiv) was dissolved in absolute MeOH (2.0 mL, 0.035M). After the addition of 5% Pd/C (2 mg), the flask was evacuated and flushed with Hydrogen gas (three times). The hydrogenation was carried out at 25 °C for 2 h. After pooling two identical batches, the catalyst was filtered off and the MeOH was removed at reduced pressure without purification to next step. The crude mixture was dissolved in methanol (0.7 mL) and aqueous 1 M NaOH (0.6 mL, 5.00 equiv) was added. The solution was heated at 45 °C for 3 h until complete consumption of starting material (checked by TLC). Then aqueous 2 M HCl (~1 mL) was added to this solution until pH was below 7, the resulting mixture was cooled to 0 °C after which a white precipitate formed. The precipitate was collected by vacuum filtration to afford the title compound as a white solid (10.5 mg after drying).

Hydrochloride salt of **2-amino-4-phenylbutanoate**

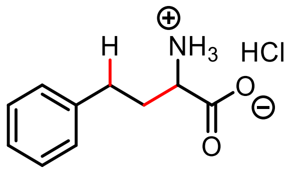

The title compound was isolated in 70% yield (10.5 mg) as a yellow solid.

^{1}H NMR (600 MHz, CDCl$_3$): δ 7.52 - 7.44 (m, 2H), 7.44 - 7.35 (m, 3H), 4.18 (t, *J* = 6.4 Hz, 1H), 2.96 - 2.80 (m, 2H), 2.42 - 2.37 (m, 1H), 2.36 – 2.30 (m, 1H); **^{13}C NMR (151 MHz, CDCl$_3$):** δ 172.1, 140.2, 129.0 (2C), 128.6 (2C), 126.8, 52.6, 31.6, 30.5; **IR (neat, cm^{-1})**: 3100 (br), 2643, 1598, 1510, 1415; **HRMS (ESI$^+$):** calculated for $[M]^-$ $C_{10}H_{13}ClNO_2^-$: 214.0635, found 214.0632.

5. Biological studies

Table S1. Antimalarial activity (IC_{50} μM)

	P. falciparum NF54				Cytotoxicity L6 cells		
compound	repl1	repl2	repl3	**mean ± SD**	repl1	repl2	**mean ± SD**
17a	0.033	0.028	0.026	**0.029 ± 0.004**	>236	>236	**>236**

Figure S1. Dose response curve (A) *P.falciparum* NF54, (B) cytotoxicity L6 cells

A

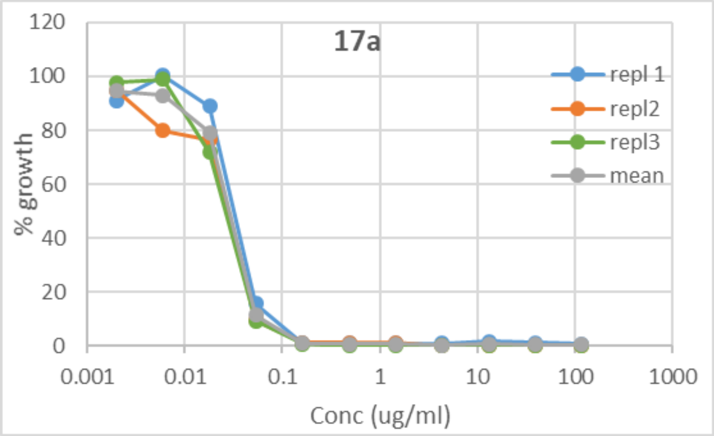

B

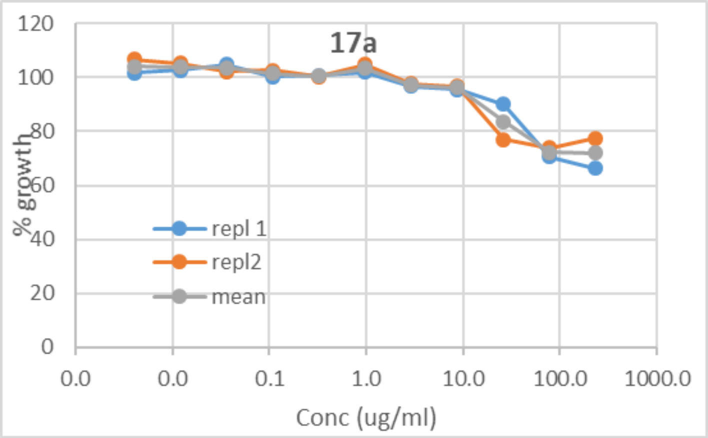

5.1. Antimalarial and cytotoxicity assay

Activity against P. falciparum. *In vitro* activity against erythrocytic stages of P. falciparum was determined using a 3H-hypoxanthine incorporation assay,[8] using the drug sensitive NF54 strain[9] and the standard drugs chloroquine (Sigma C6628) and artesunate (Sigma A3731). Compounds were dissolved in DMSO at 10 mg/ml and further diluted in medium before added to parasite cultures incubated in RPMI 1640 medium without hypoxanthine, supplemented with HEPES (5.94 g/L), NaHCO3 (2.1 g/L), neomycin (100 U/mL), AlbumaxR (5 g/L) and washed human red cells A+ at 2.5% haematocrit (0.3% parasitaemia). Serial drug dilutions of eleven 3-fold dilution steps covering a range from 100 to 0.002 µg/ml were prepared. The 96-well plates were incubated in a humidified atmosphere at 37 °C; 4% CO_2, 3% O_2, 93% N_2. After 48 h 50 µL of 3H-hypoxanthine (=0.5 µCi) was added to each well of the plate. The plates were incubated for a further 24 h under the same conditions. The plates were then harvested with a Betaplate™ cell harvester (Wallac, Zurich, Switzerland), and the red blood cells transferred onto a glass fibre filter then washed with distilled water. The dried filters were inserted into a plastic foil with 10 ml of scintillation fluid, and counted in a Betaplate™ liquid scintillation counter (Wallac, Zurich, Switzerland). IC_{50} values were calculated from sigmoidal inhibition curves by linear regression[10] (Huber 1993) using Microsoft Excel. Chloroquine and artemisinin are used as control.

In vitro cytotoxicity with L-6 cells. Assays were performed in 96-well microtiter plates, each well containing 100 µL of RPMI 1640 medium supplemented with 1% L-glutamine (200 mM) and 10% fetal bovine serum,

---

[8] R. E. Desjardins, C. J. Canfield, J. D. Haynes, J. D. Chulay, *Antimicrob. Agents. Chemother.* **1979**, *16*, 710-718; H. Matile, J. R. L. Pink, Plasmodium falciparum malaria parasite cultures and their use in immunology. In I. Lefkovits, B. Pernis, ed. Immunological Methods. (Academic Press, San Diego), **1990**, 221-224.

[9] T. Ponnudurai, A. D. Leeuwenberg, J. H. Meuwissen, *Trop. Geogr. Med.* **1981**, *33,* 50-54.

[10] W. Huber, J. C. Koella, *Acta Trop.* **1993**, *55,* 257–261.

and 4000 L-6 cells (a primary cell line derived from rat skeletal myoblasts).[11] Serial drug dilutions of eleven 3-fold dilution steps covering a range from 100 to 0.002 µg/mK were prepared. After 70h of incubation the plates were inspected under an inverted microscope to assure growth of the controls and sterile conditions. 10µL of resazurin was then added to each well and the plates incubated for another 2 hours. Then the plates were read with a Spectramax Gemini XS microplate fluorometer (Molecular Devices Cooperation, Sunnyvale, CA, USA) using an excitation wave length of 536 nm and an emission wave length of 588 nm. The IC_{50} values were calculated by linear regression[12] and 4-parameter logistic regression from the sigmoidal dose inhibition curves using SoftmaxPro software (Molecular Devices Cooperation, Sunnyvale, CA, USA). Podophyllotoxin (Sigma P4405) is used as control.

[11] C. Page, C. M. Page, C. Noel, (1993). *Int. J. Oncol.* **1993**, *3*, 473–476; S. A. Ahmed, R. M. Gogal, J. E. Walsh, (1994). *J. Immunol. Methods.* **1994**, *170*, 211–224.

[12] W. Huber, J. C. Koella, *Acta Trop.* **1993**, *55*, 257–261.

6. Spectral Data

1,1,1-trifluoro-*N*-methyl-6-phenylhexan-2-amine (1a)

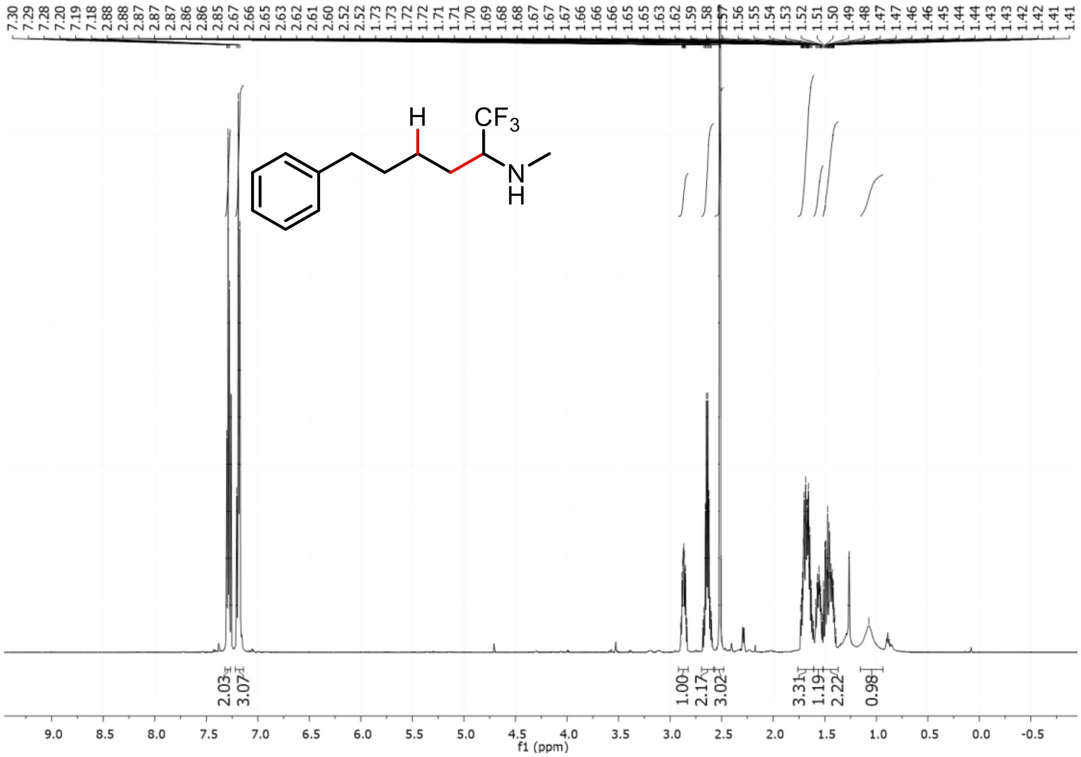

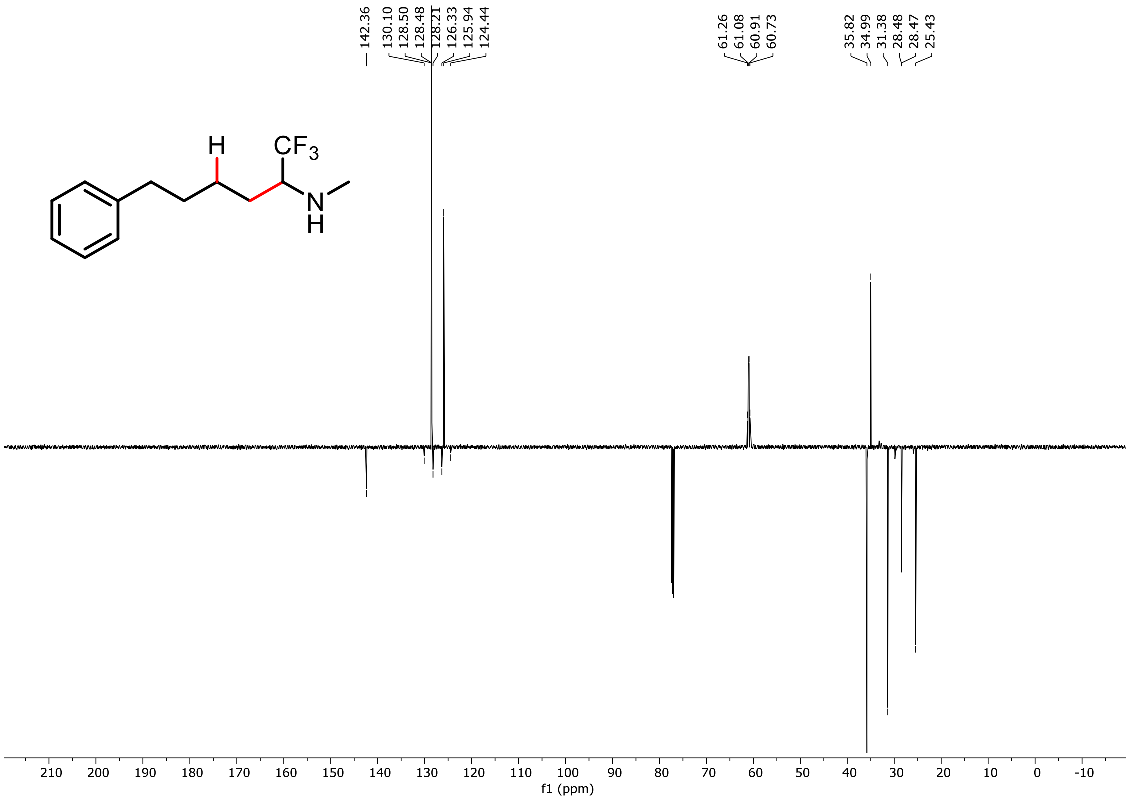

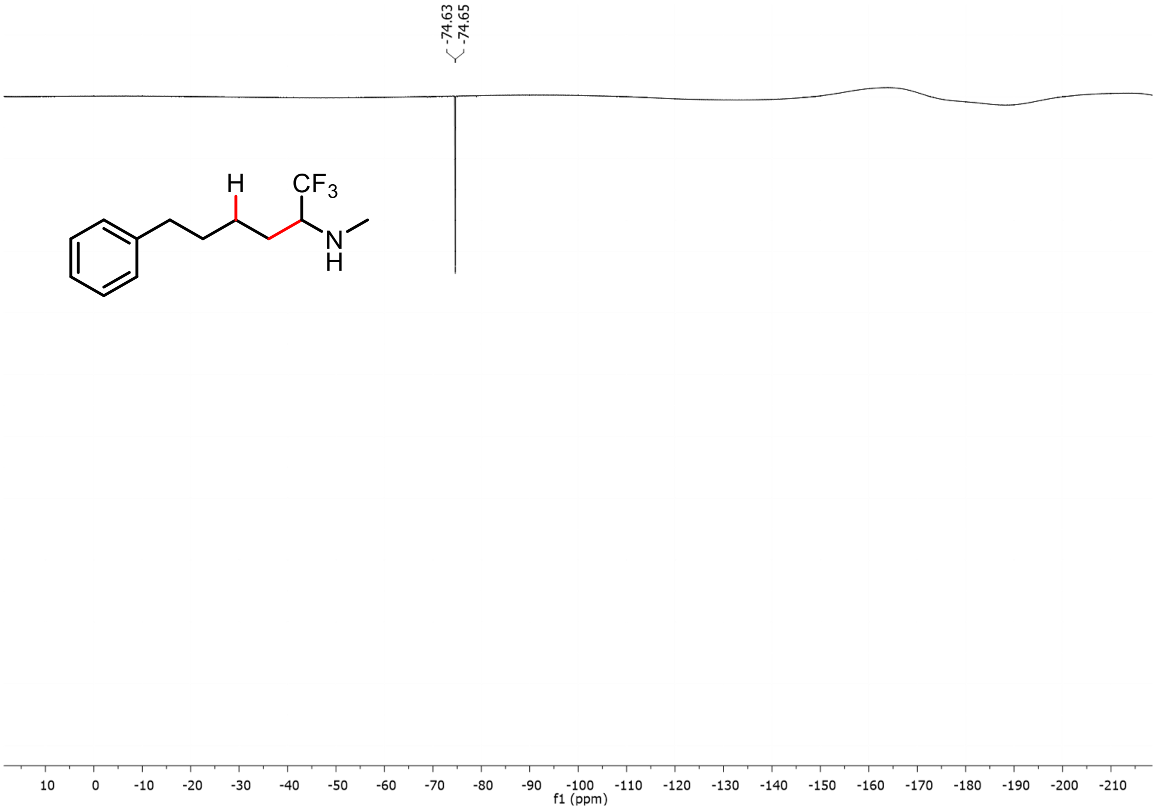
-74.63
-74.65
H
CF3
N
H
10 0 -10 -20 -30 -40 -50 -60 -70 -80 -90 -100 -110 -120 -130 -140 -150 -160 -170 -180 -190 -200 -210
f1 (ppm)

N-benzyl-1,1,1-trifluoro-6-phenylhexan-2-amineamine (1aa)

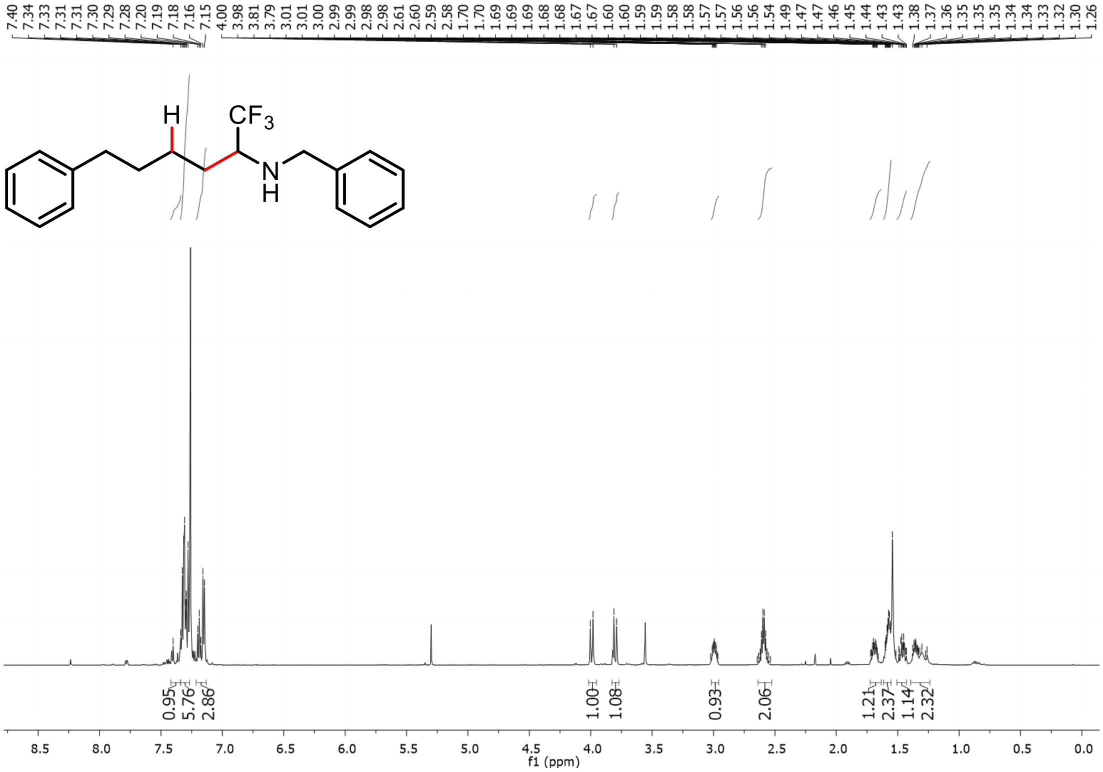

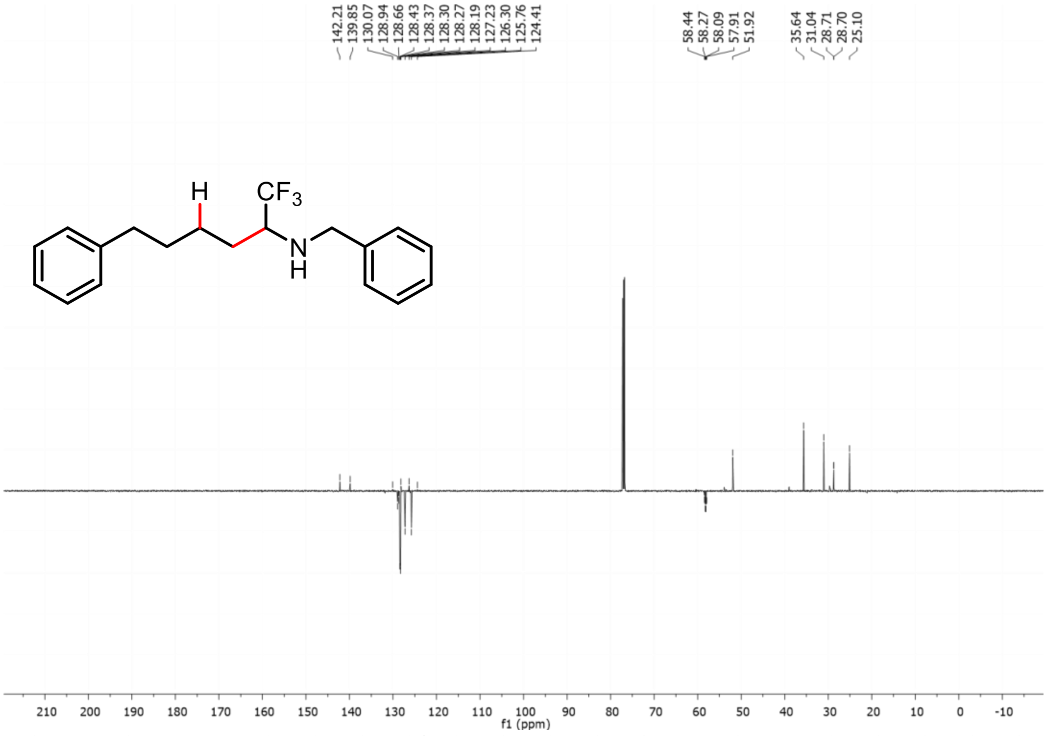
142.21
139.85
130.07
128.94
128.66
128.43
128.37
128.30
128.27
128.19
127.23
126.30
125.76
124.41
58.44
58.27
58.09
57.91
51.92
35.64
31.04
28.71
28.70
25.10
H
CF3
N
H
f1 (ppm)

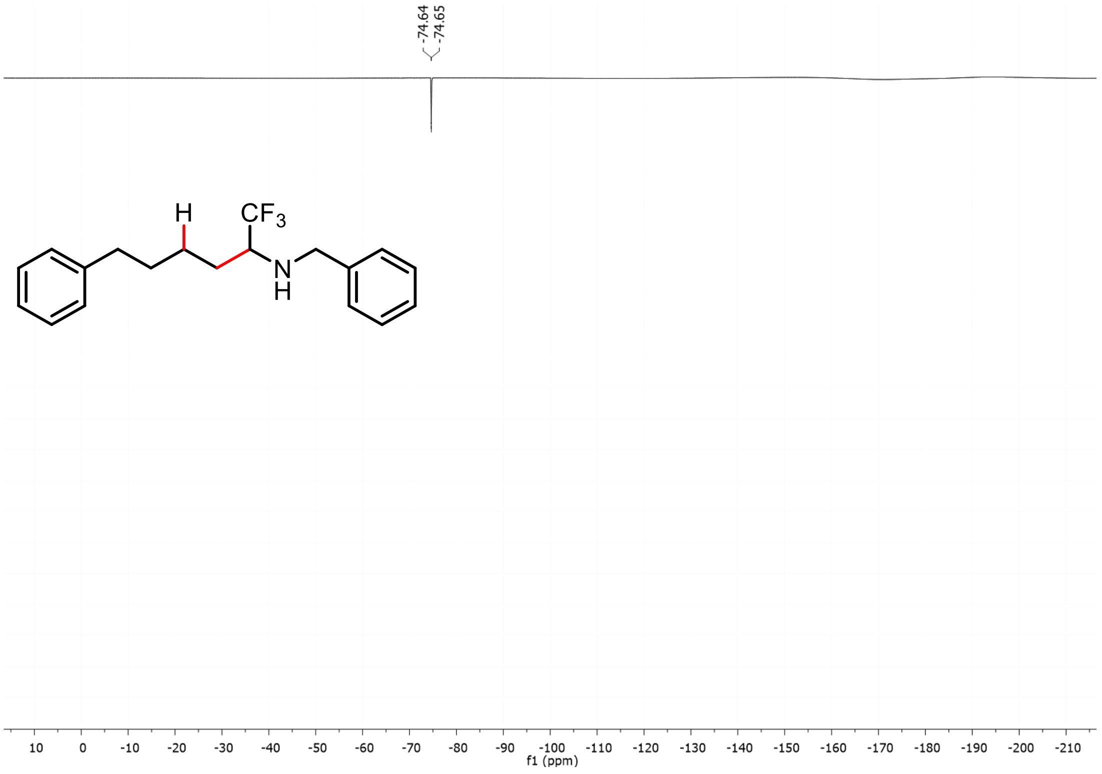
-74.64
-74.65
H
CF3
N
H
f1 (ppm)

1,1,1-trifluoro-N-methyldodecan-2-amine (2a)

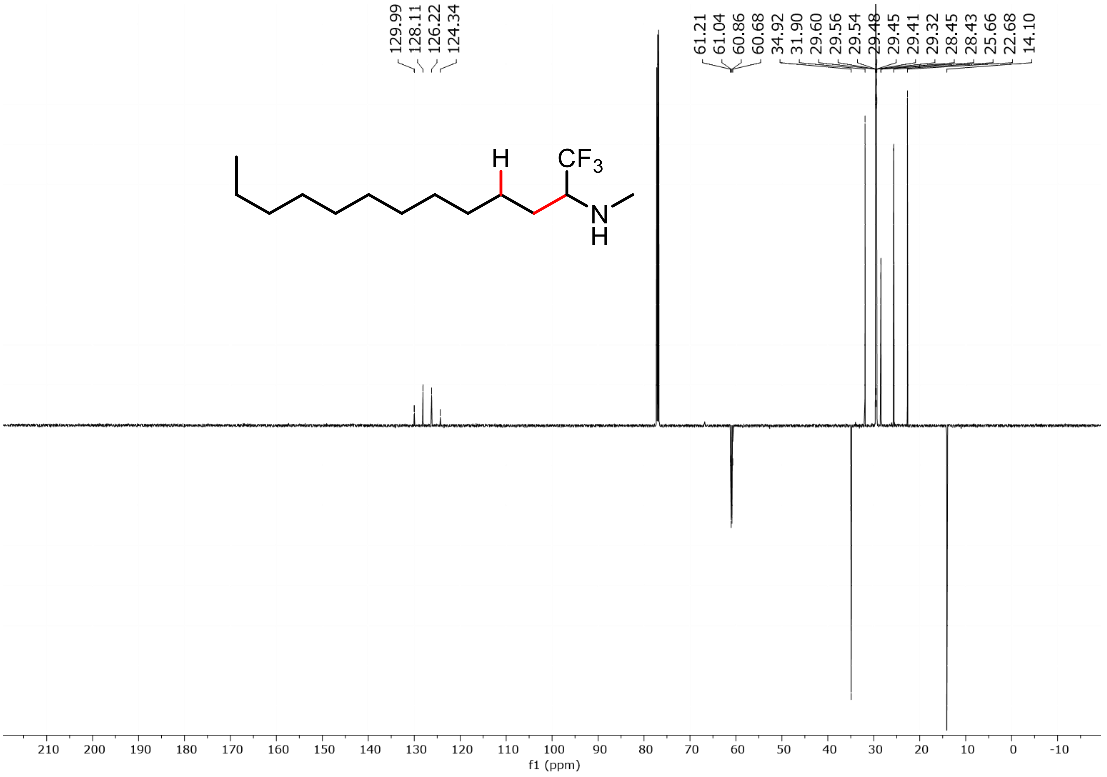

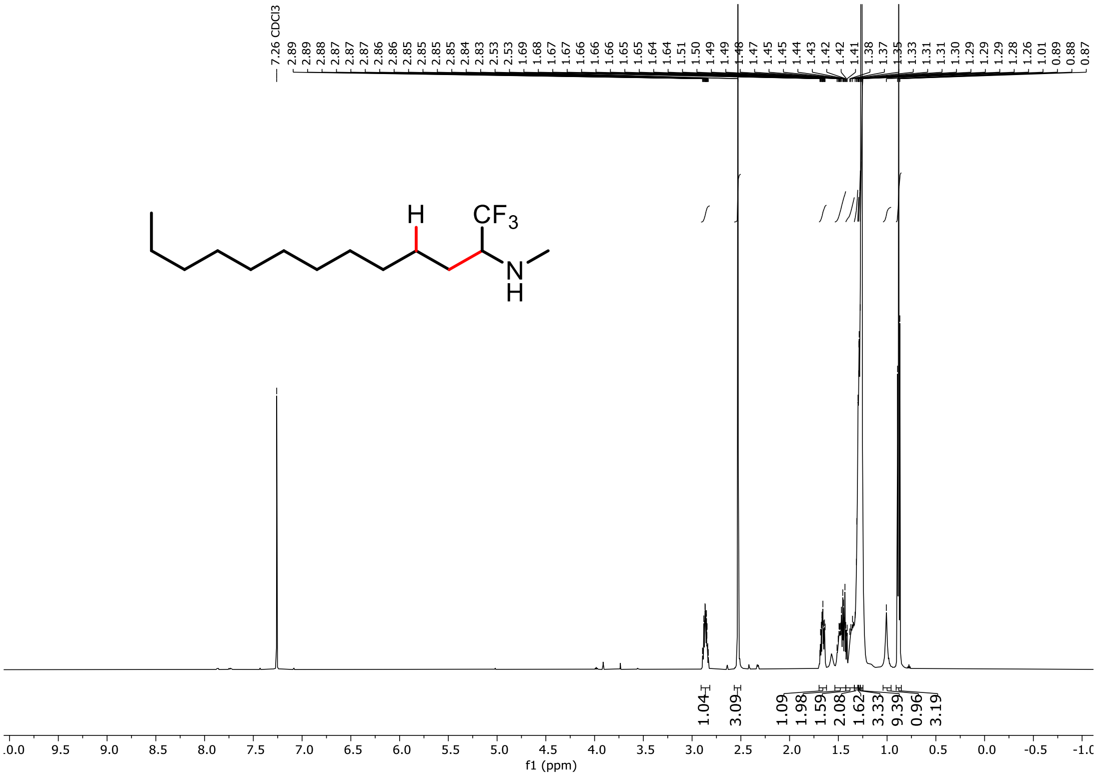

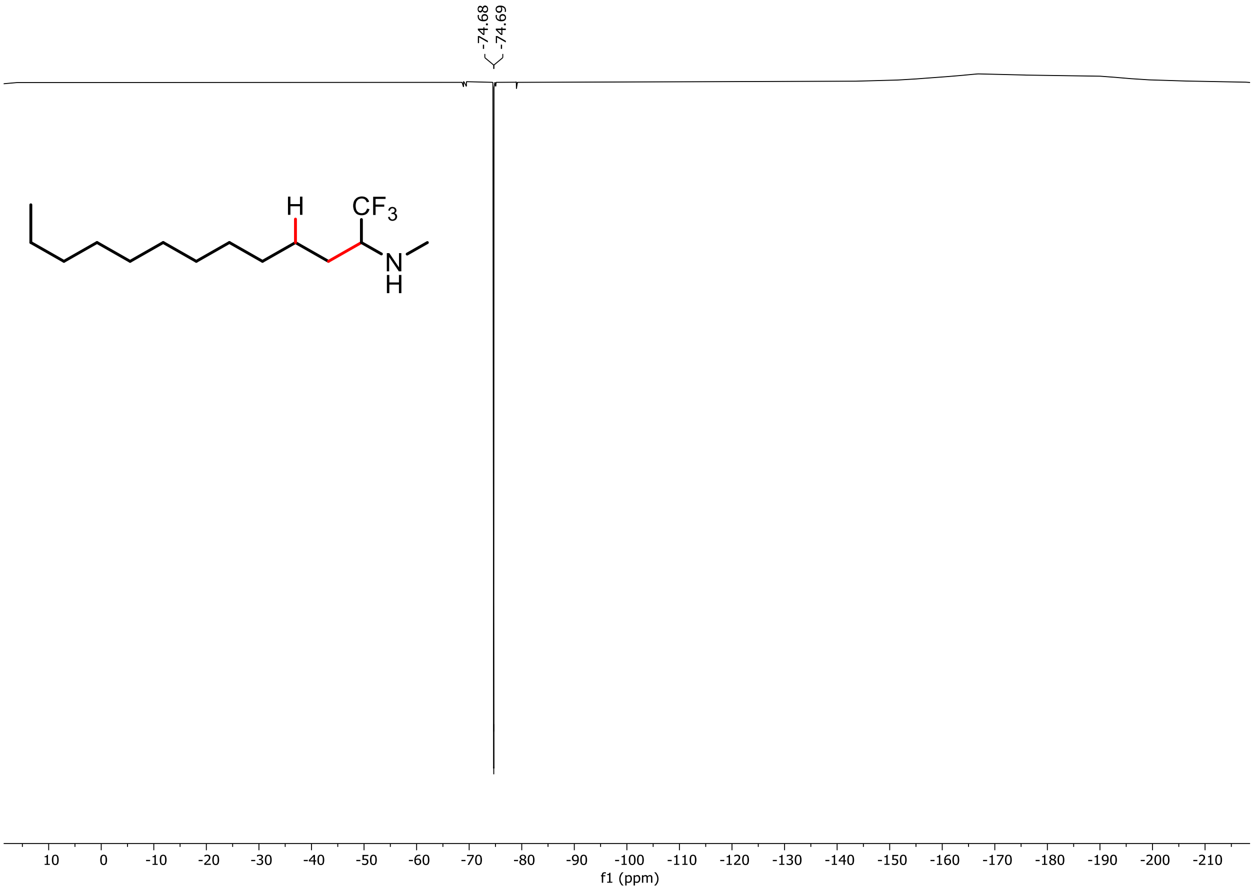
-74.68
-74.69
H
CF3
N
H
10
0
-10
-20
-30
-40
-50
-60
-70
-80
-90
-100
-110
-120
-130
-140
-150
-160
-170
-180
-190
-200
-210
f1 (ppm)

1-cycloheptyl-2,2,2-trifluoro-*N*-methylethan-1-amine (3a)

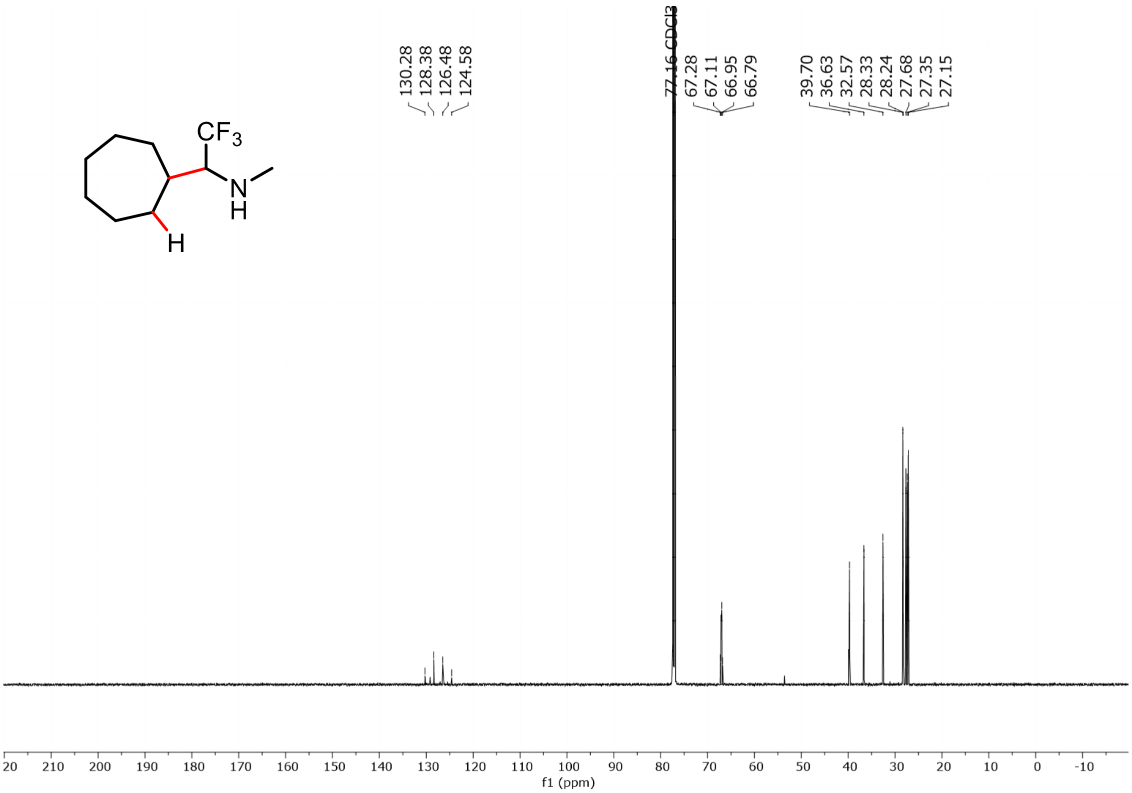

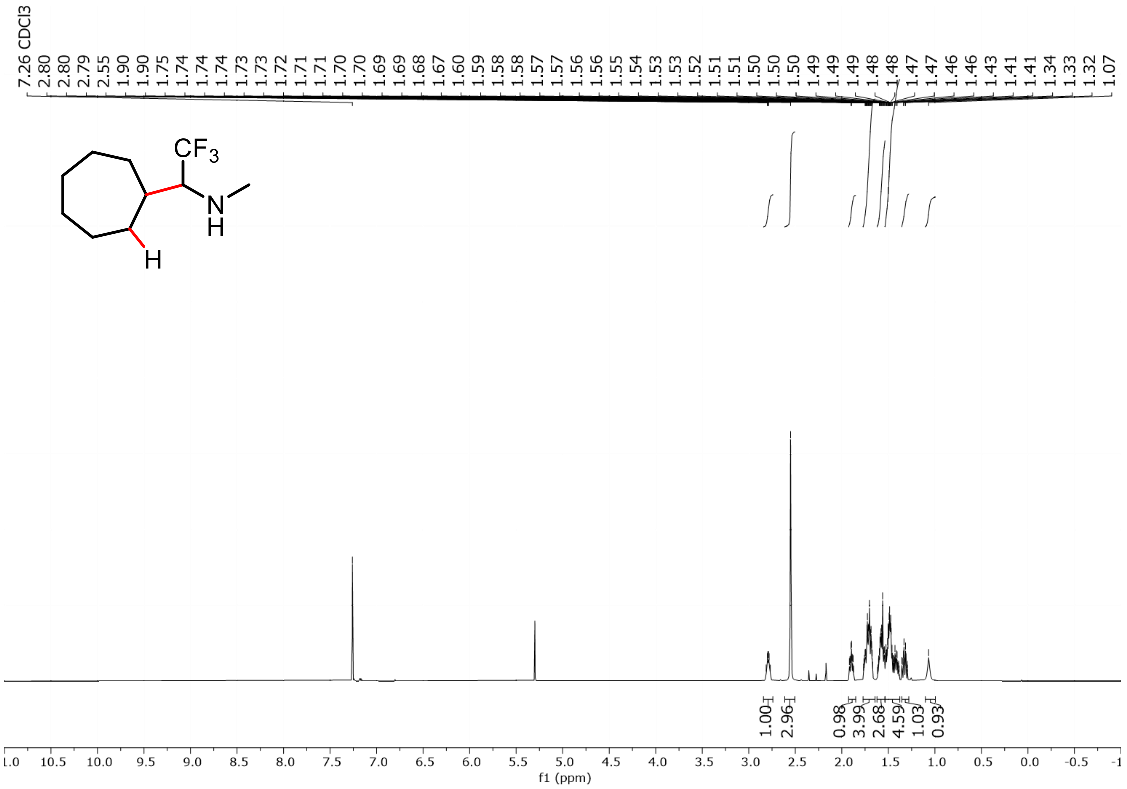

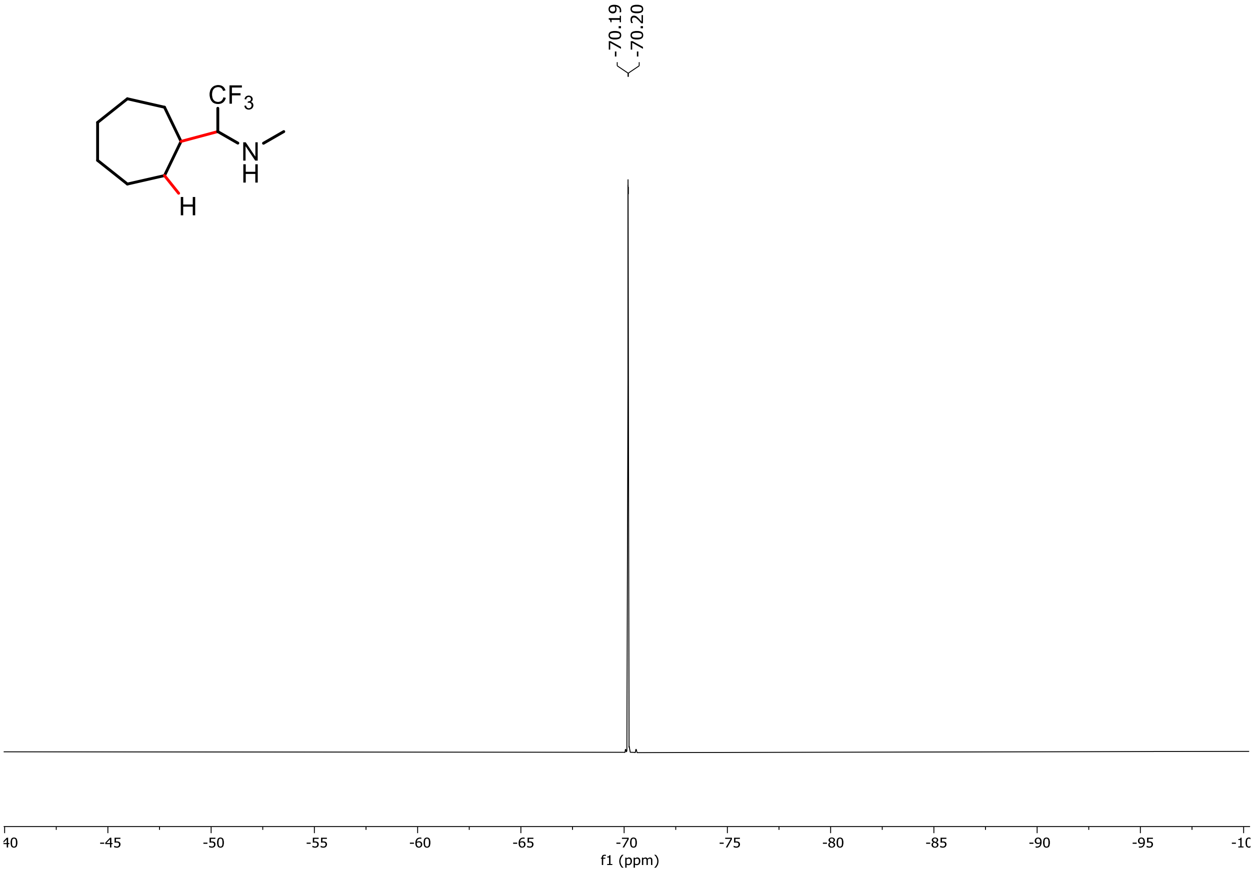
CF3
N
H
H
-70.19
-70.20
40
-45
-50
-55
-60
-65
-70
-75
-80
-85
-90
-95
-10
f1 (ppm)

4-(benzo[d][1,3]dioxol-5-yl)-1,1,1-trifluoro-*N*,3-dimethylbutan-2-amine (4a)

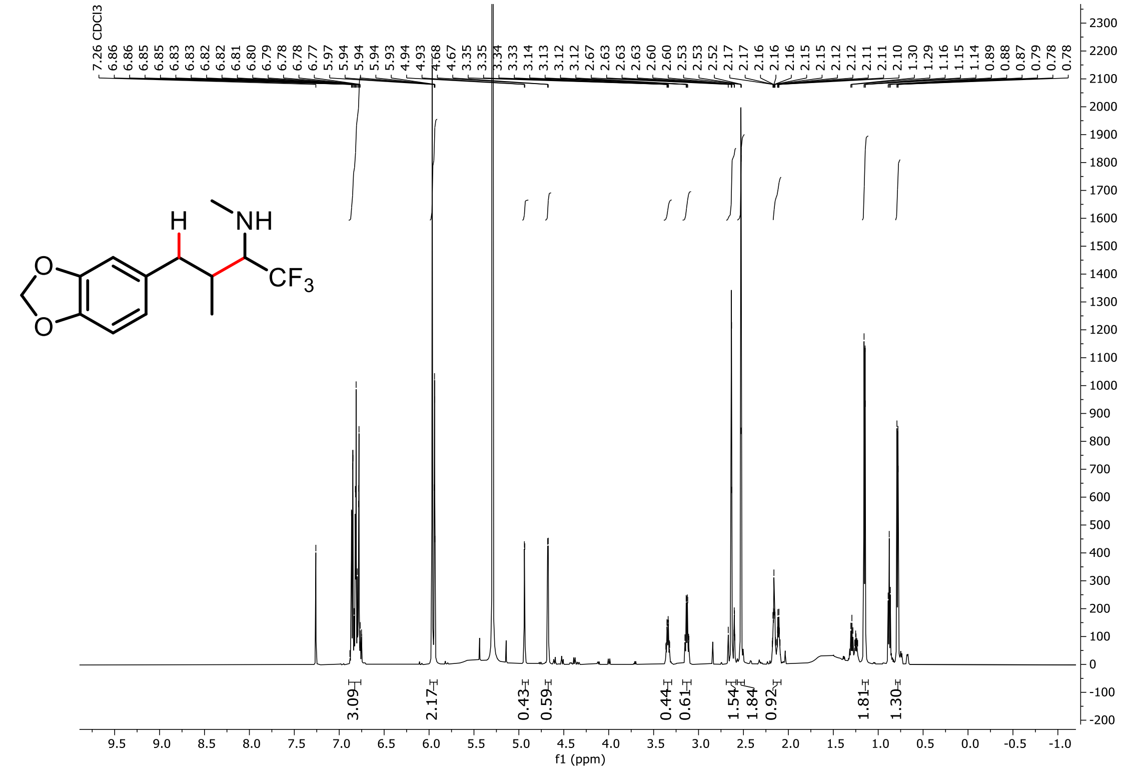

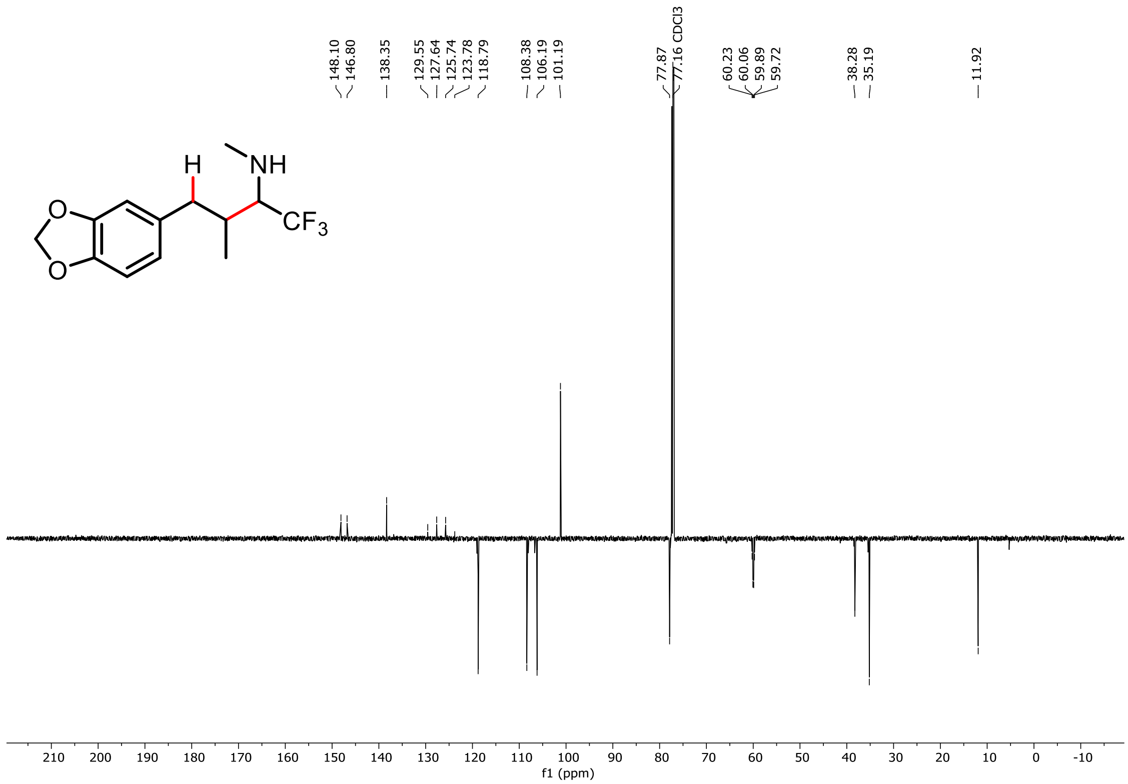

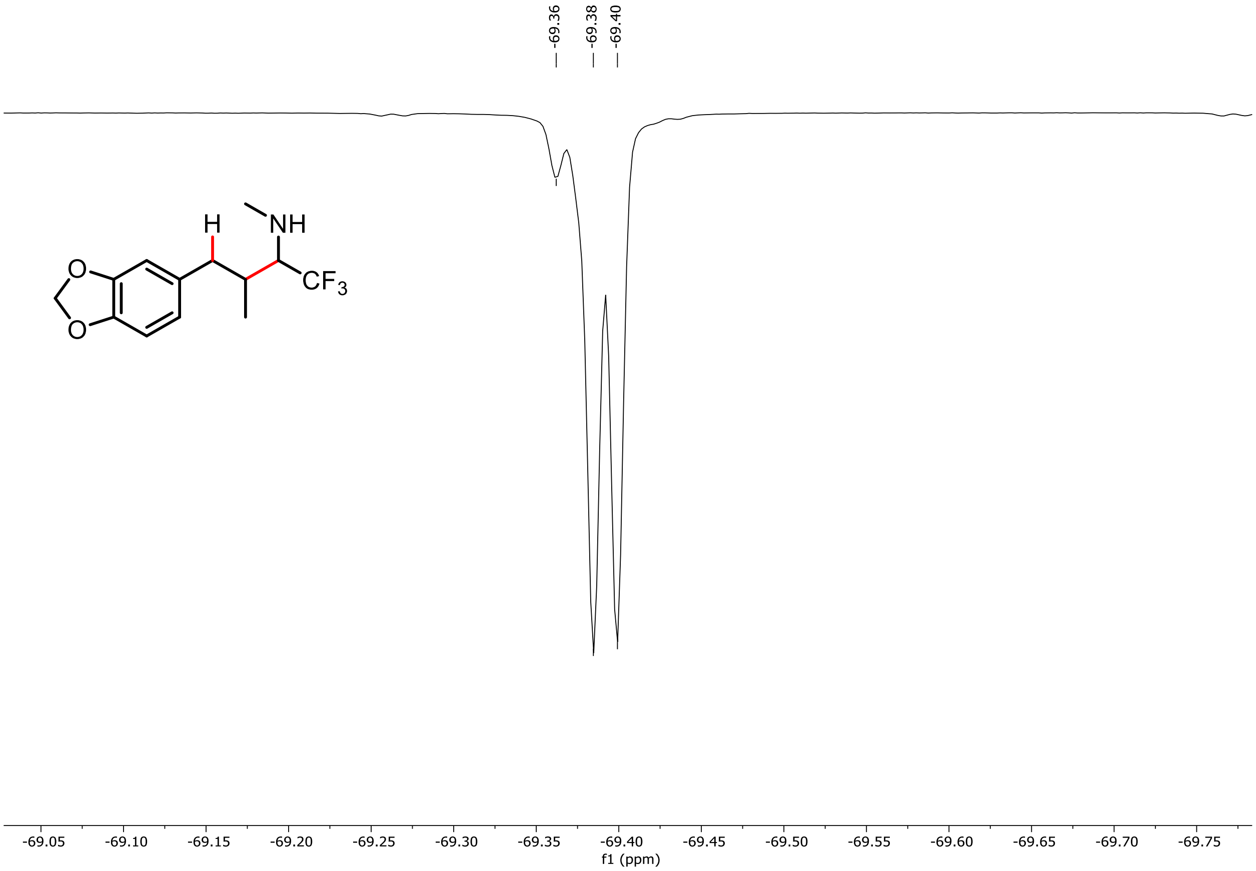

-69.36
-69.38
-69.40
H
NH
CF3
O
O
-69.05
-69.10
-69.15
-69.20
-69.25
-69.30
-69.35
-69.40
-69.45
-69.50
-69.55
-69.60
-69.65
-69.70
-69.75
f1 (ppm)

10-bromo-1,1,1-trifluoro-*N*-methyldecan-2-amine (5a)

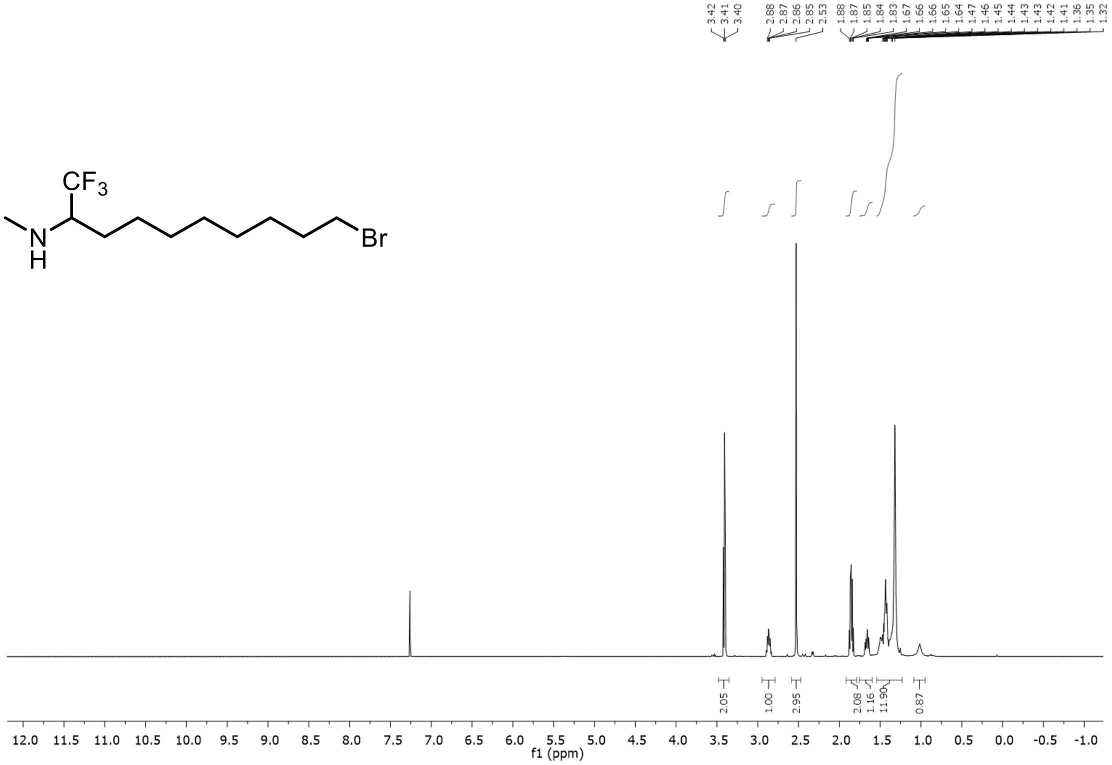

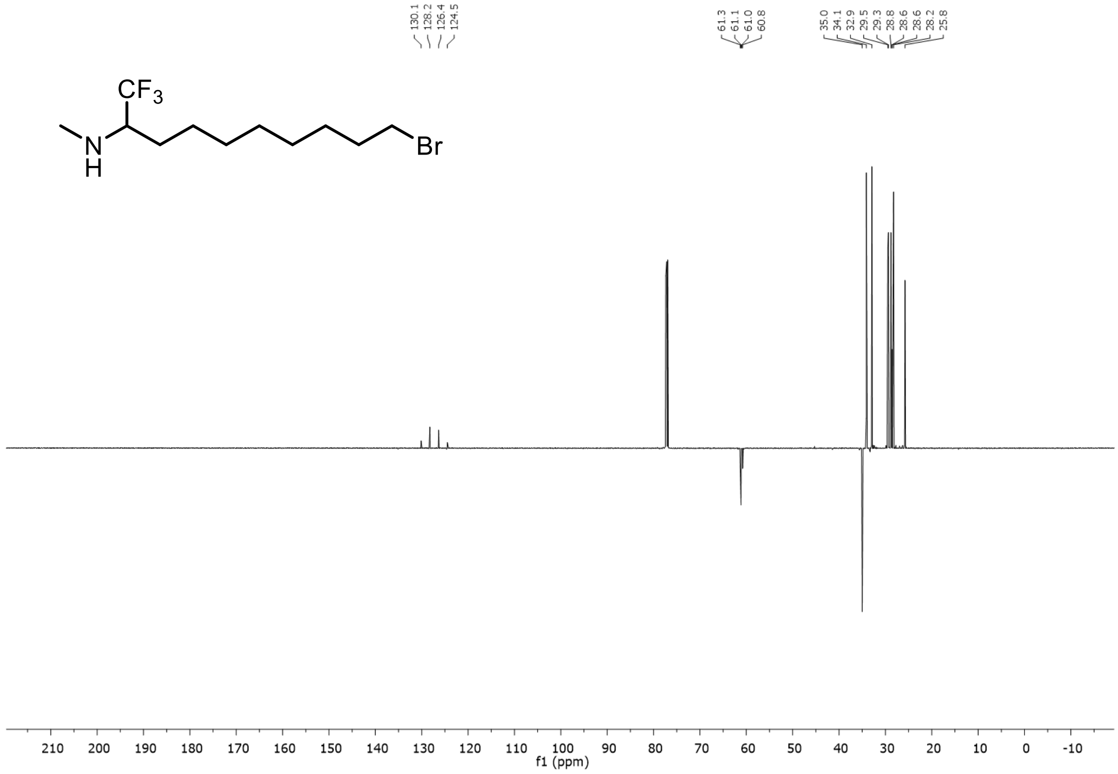

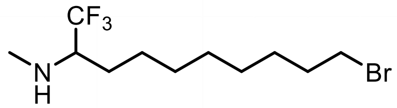
CF3
N
H
Br

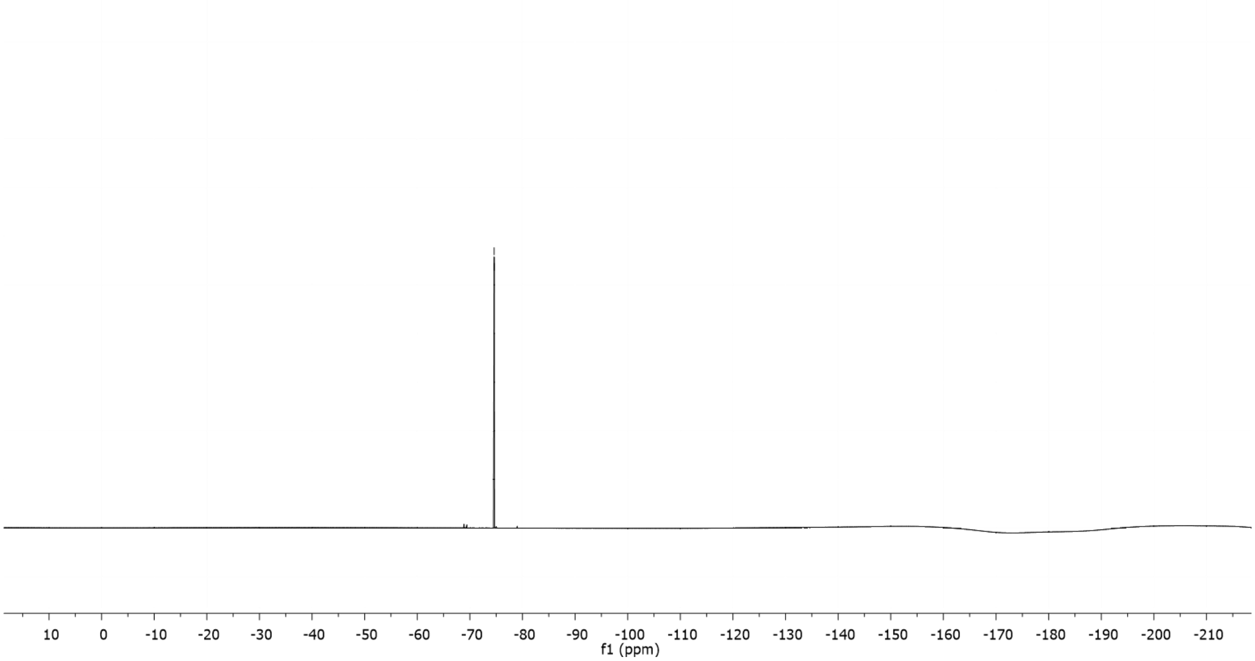
-74.62
-74.63
10
0
-10
-20
-30
-40
-50
-60
-70
-80
-90
-100
-110
-120
-130
-140
-150
-160
-170
-180
-190
-200
-210
f1 (ppm)

Methyl 8,8,8-trifluoro-7-(methylamino)octanoate (6a)

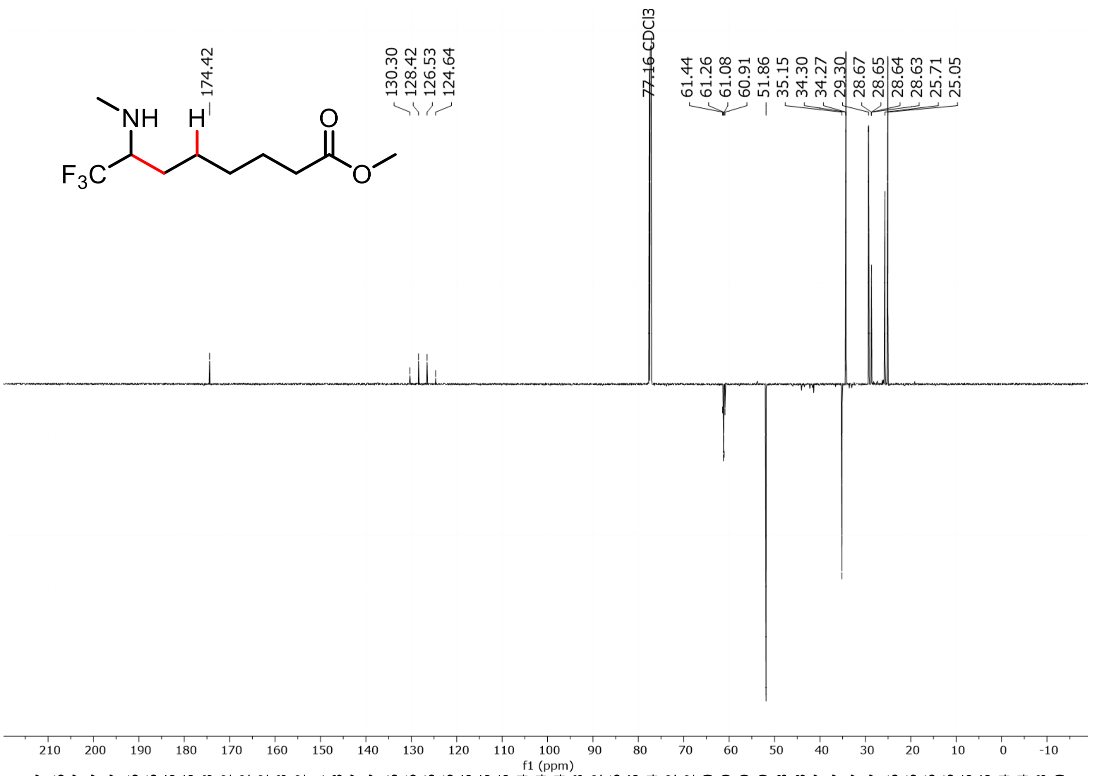

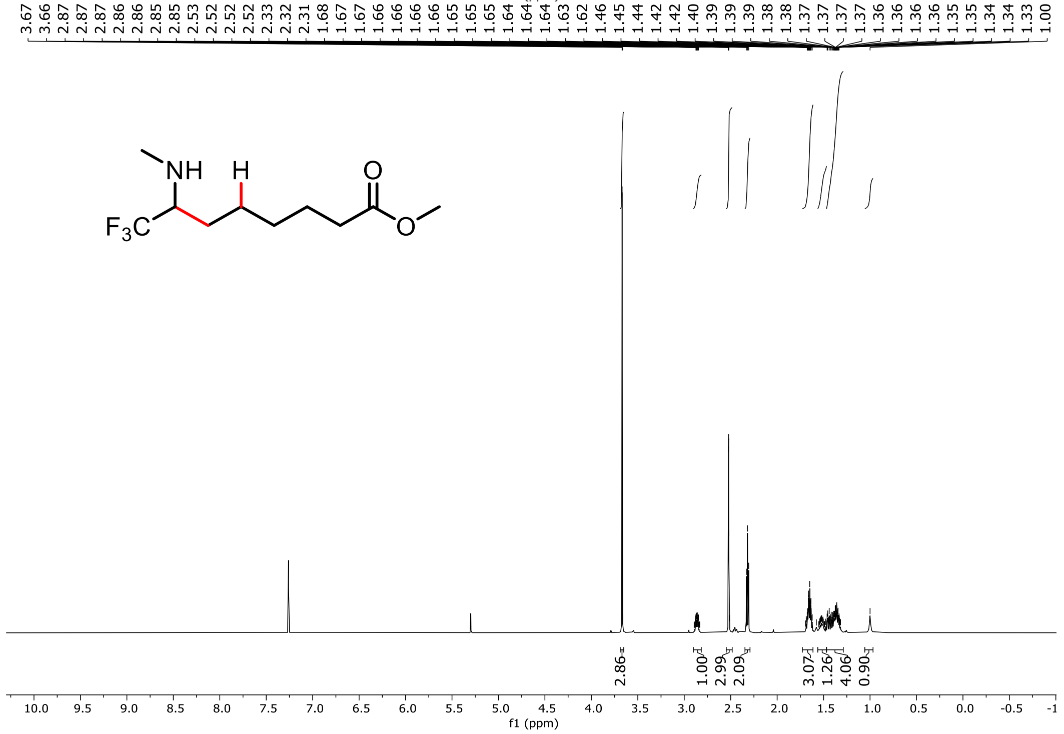

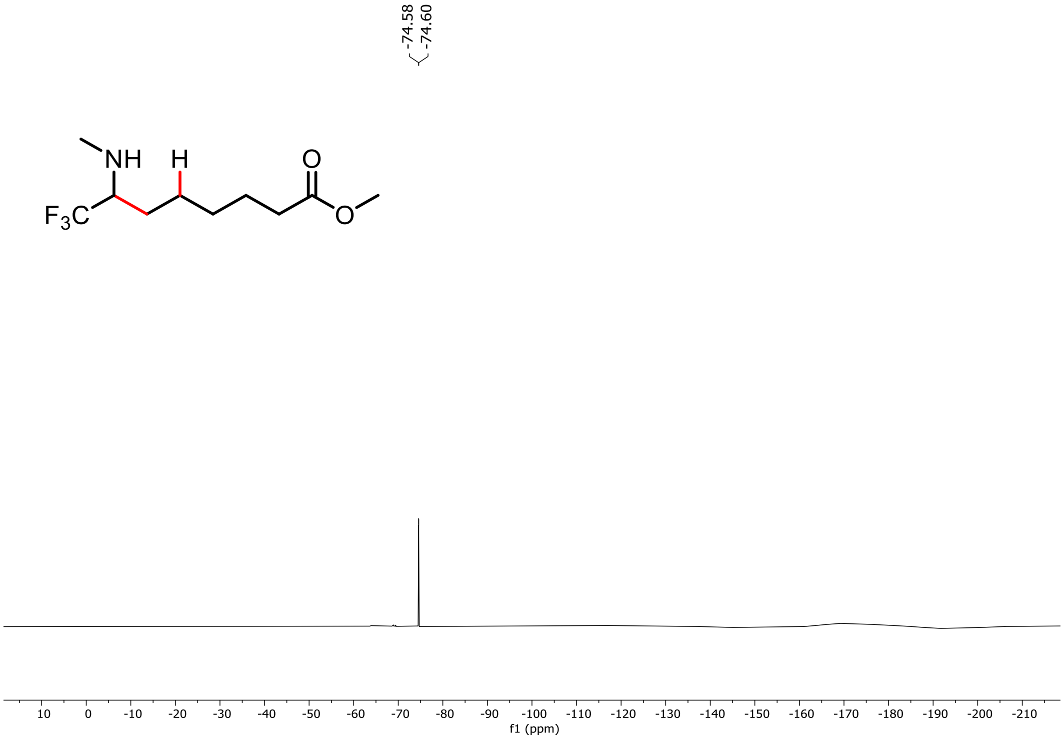
-74.58
-74.60
NH
H
O
F3C
O
10
0
-10
-20
-30
-40
-50
-60
-70
-80
-90
-100
-110
-120
-130
-140
-150
-160
-170
-180
-190
-200
-210
f1 (ppm)

12,12,12-trifluoro-11-(methylamino)dodecan-1-ol (7a)

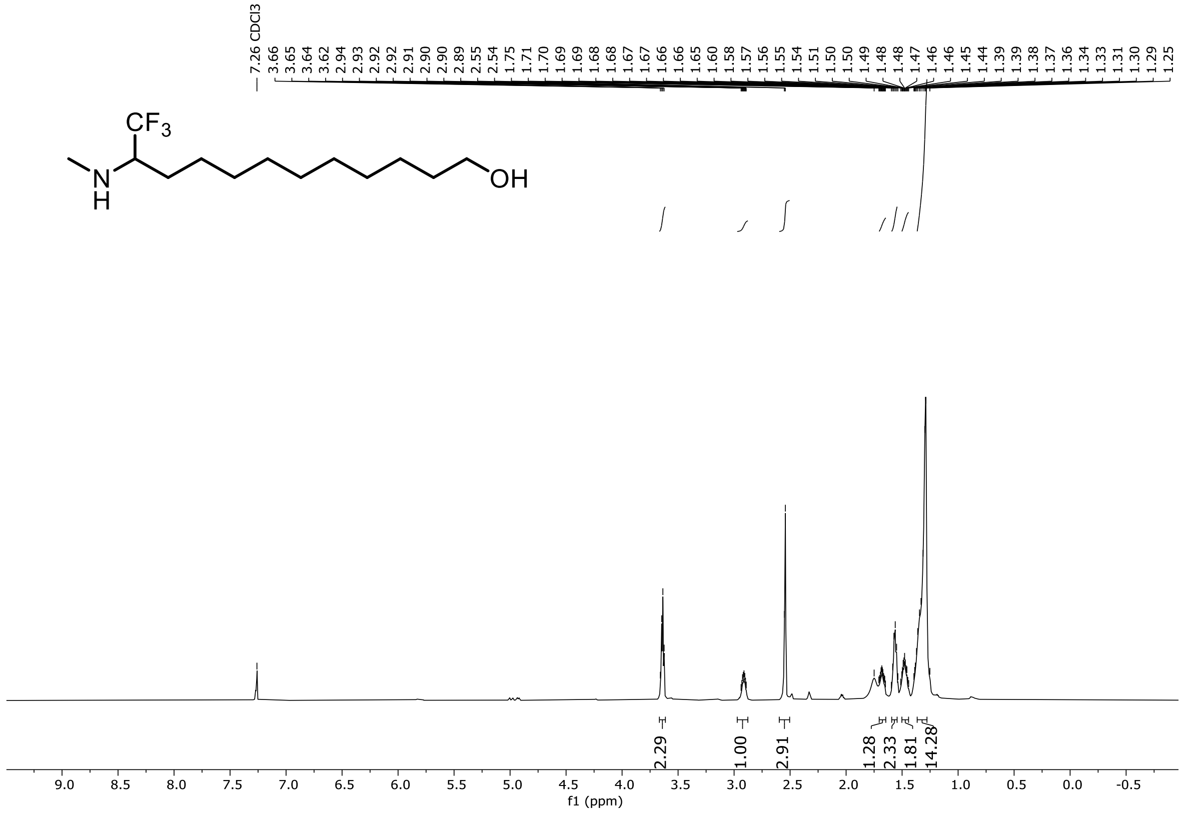

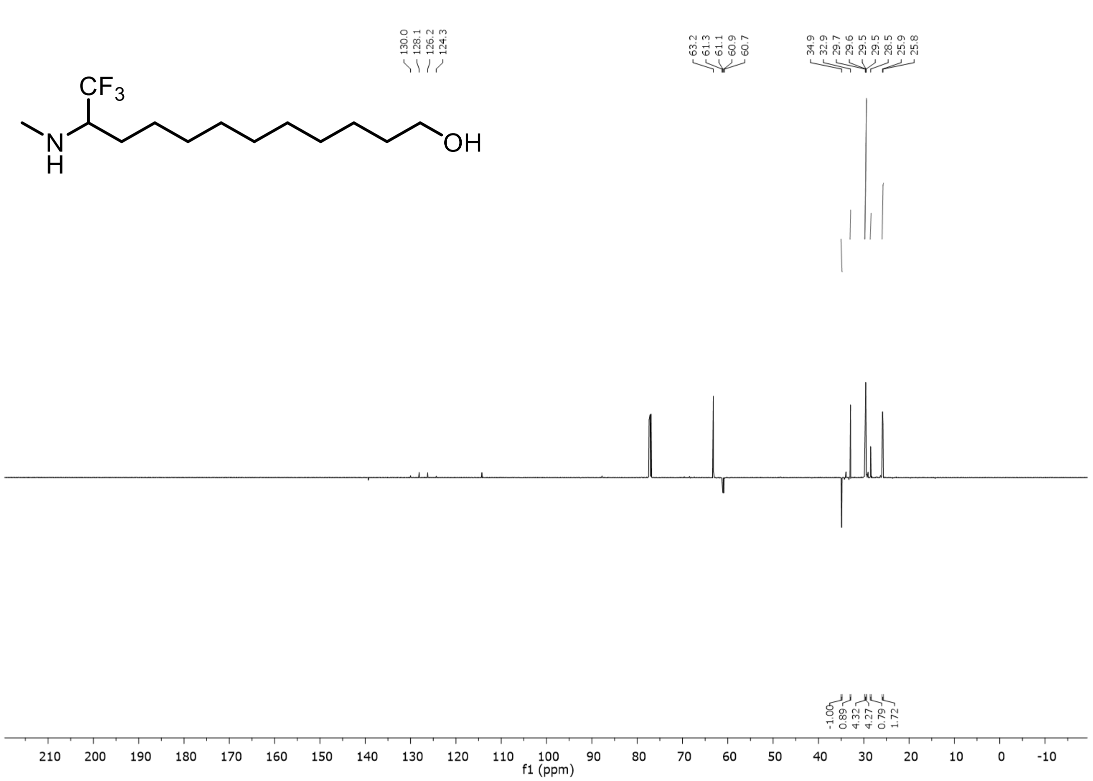

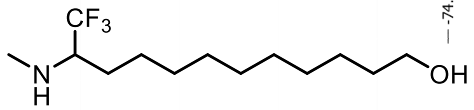
CF3
N
H
OH

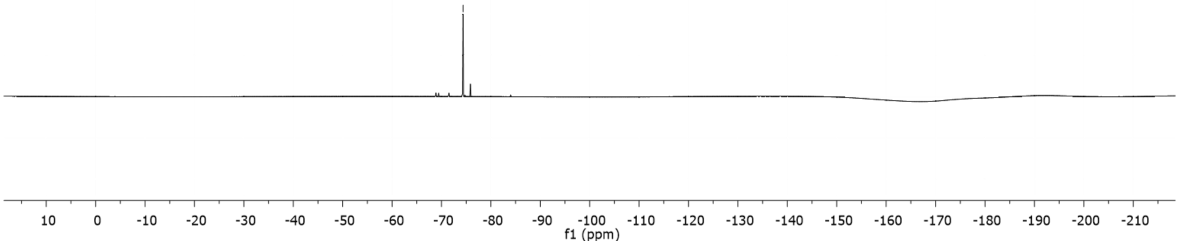
-74.36
10
0
-10
-20
-30
-40
-50
-60
-70
-80
-90
-100
-110
-120
-130
-140
-150
-160
-170
-180
-190
-200
-210
f1 (ppm)

(*E*)-4-cyclohexyl-1,1,1-trifluoro-*N*-methylbut-3-en-2-amine (8a)

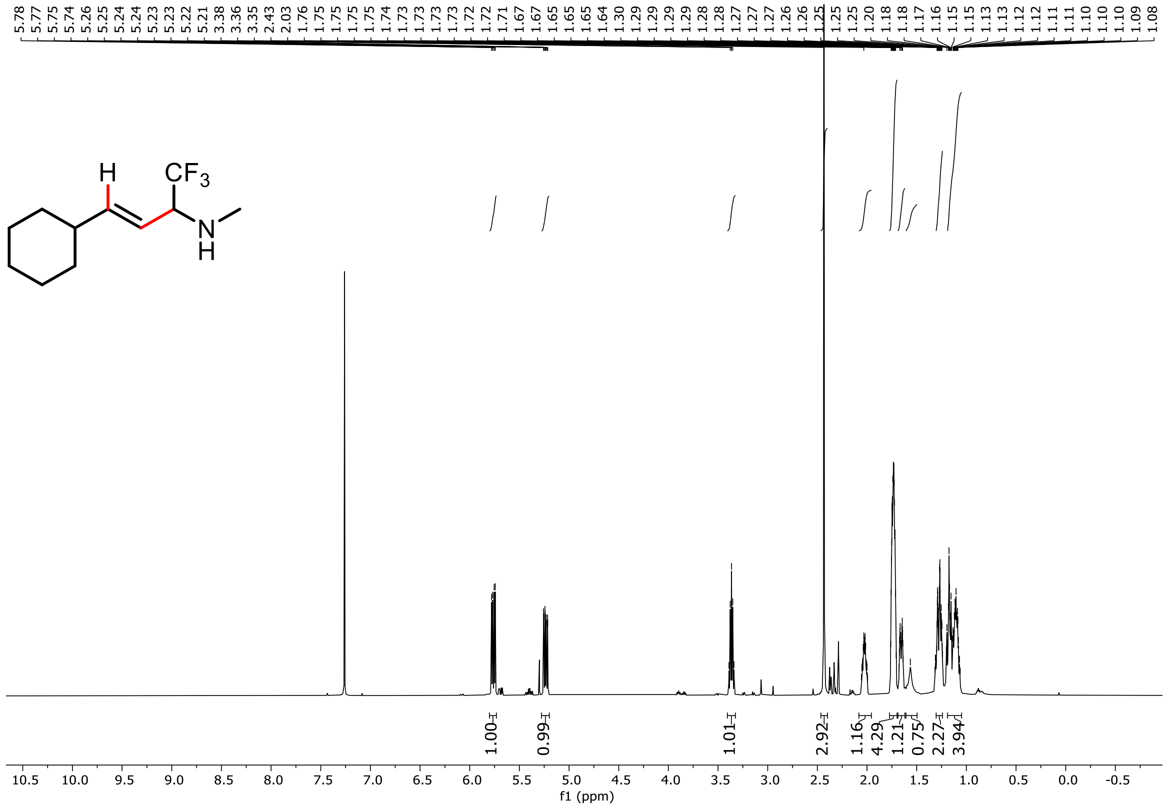

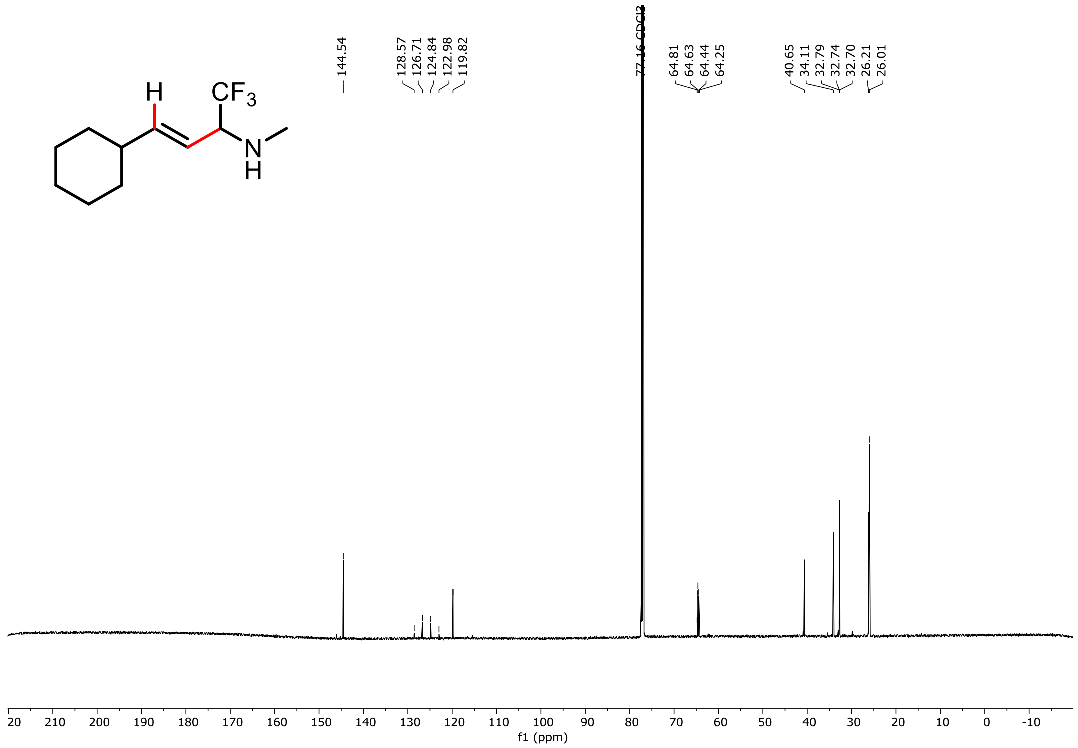

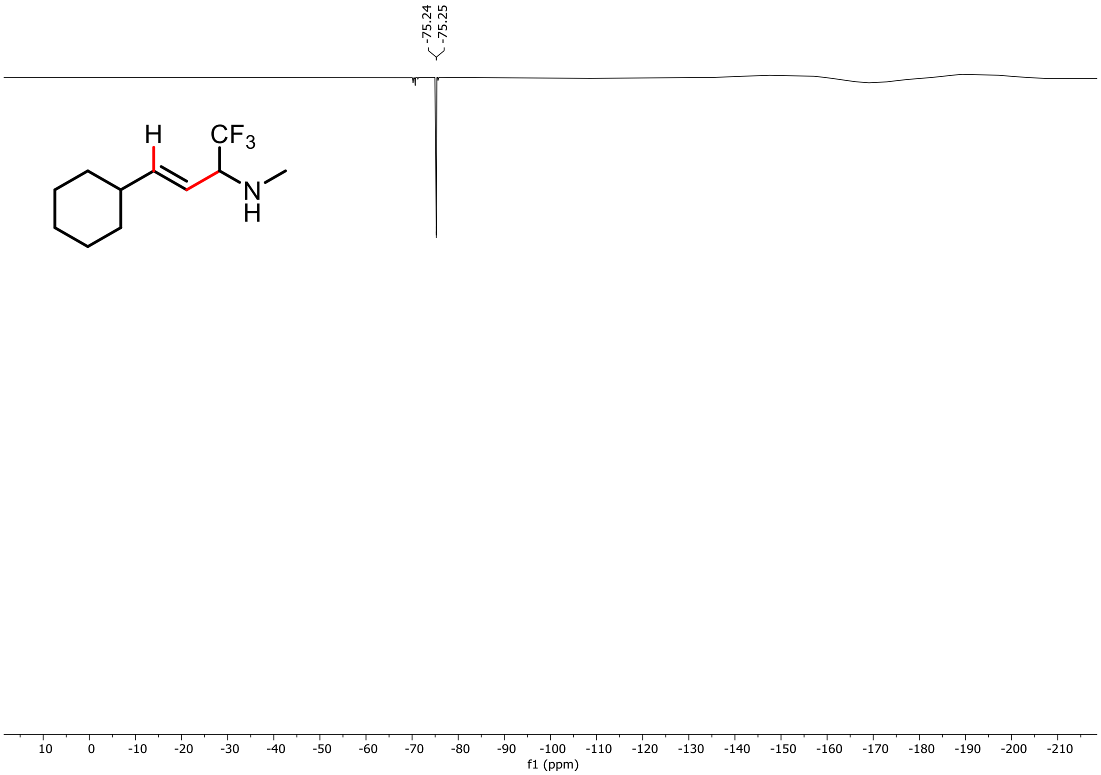

-75.24
-75.25
H
CF_3
N
H
10 0 -10 -20 -30 -40 -50 -60 -70 -80 -90 -100 -110 -120 -130 -140 -150 -160 -170 -180 -190 -200 -210
f1 (ppm)

(*E*)-1,1,1-trifluoro-*N*,3-dimethyl-4-phenylbut-3-en-2-amine (9a)

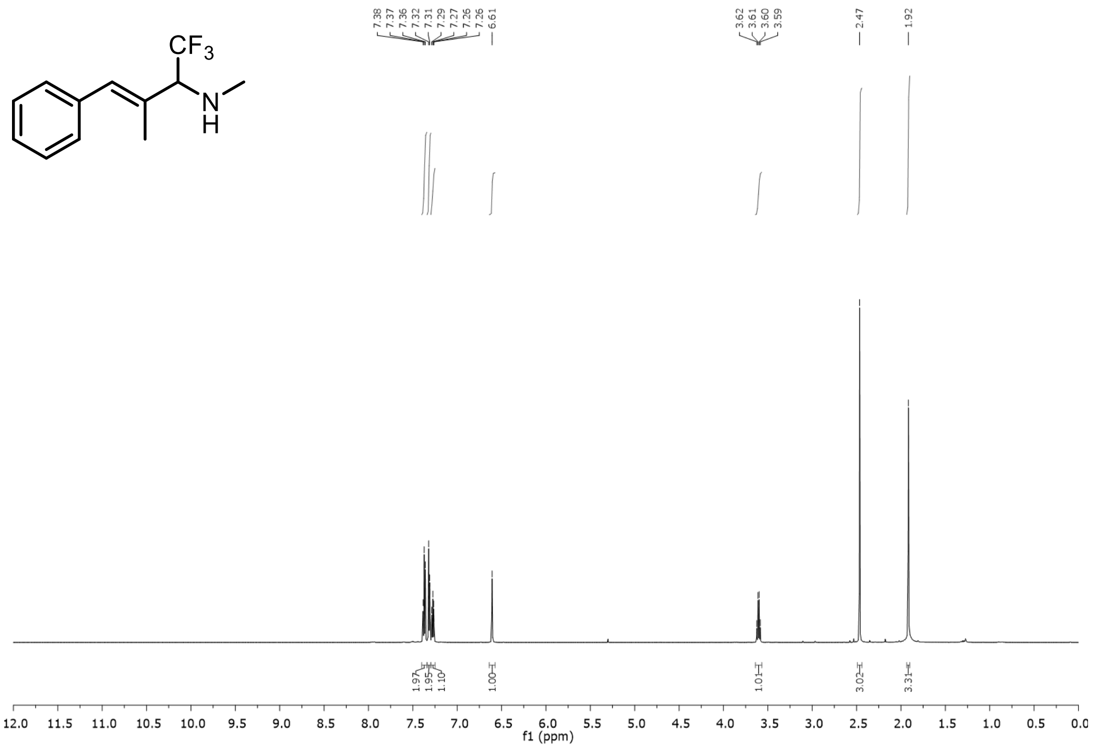

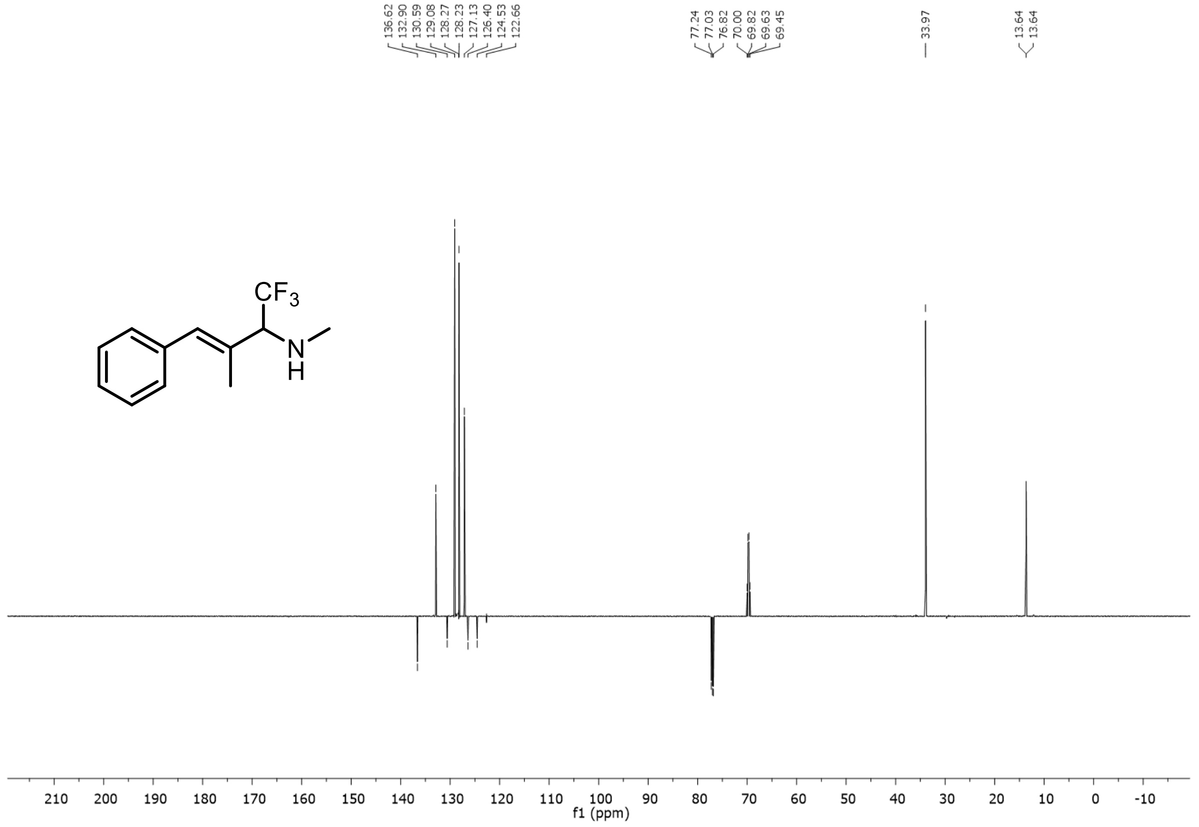

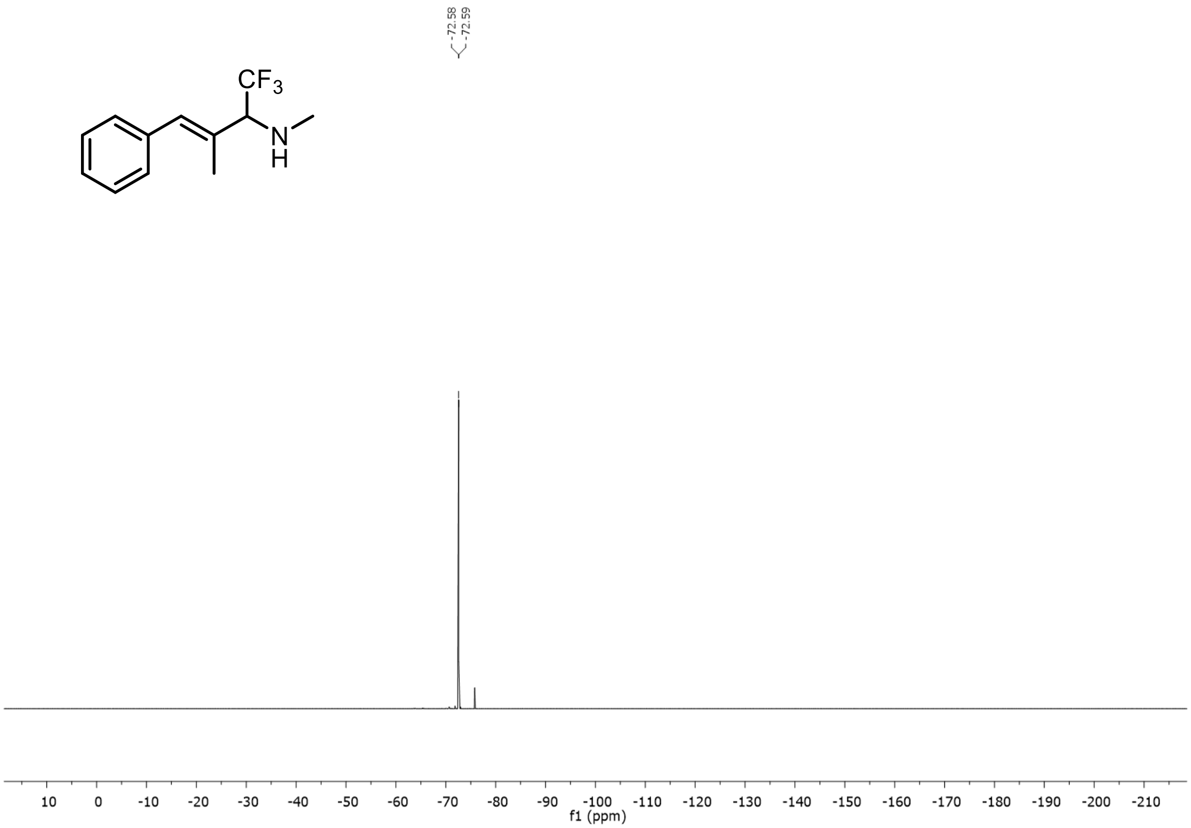
-72.58
-72.59
10
0
-10
-20
-30
-40
-50
-60
-70
-80
-90
-100
-110
-120
-130
-140
-150
-160
-170
-180
-190
-200
-210
f1 (ppm)

(*E*)-4-(cyclohex-1-en-1-yl)-1,1,1-trifluoro-*N*-methylbut-3-en-2-amine (10a)

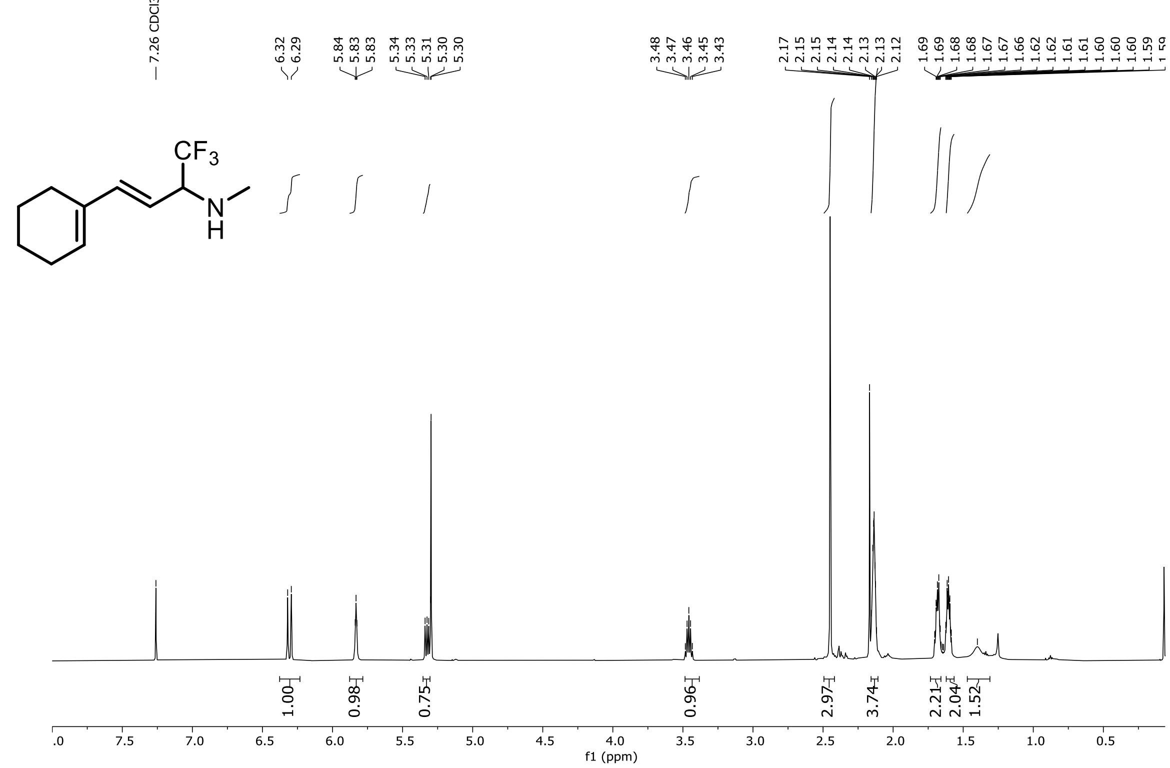

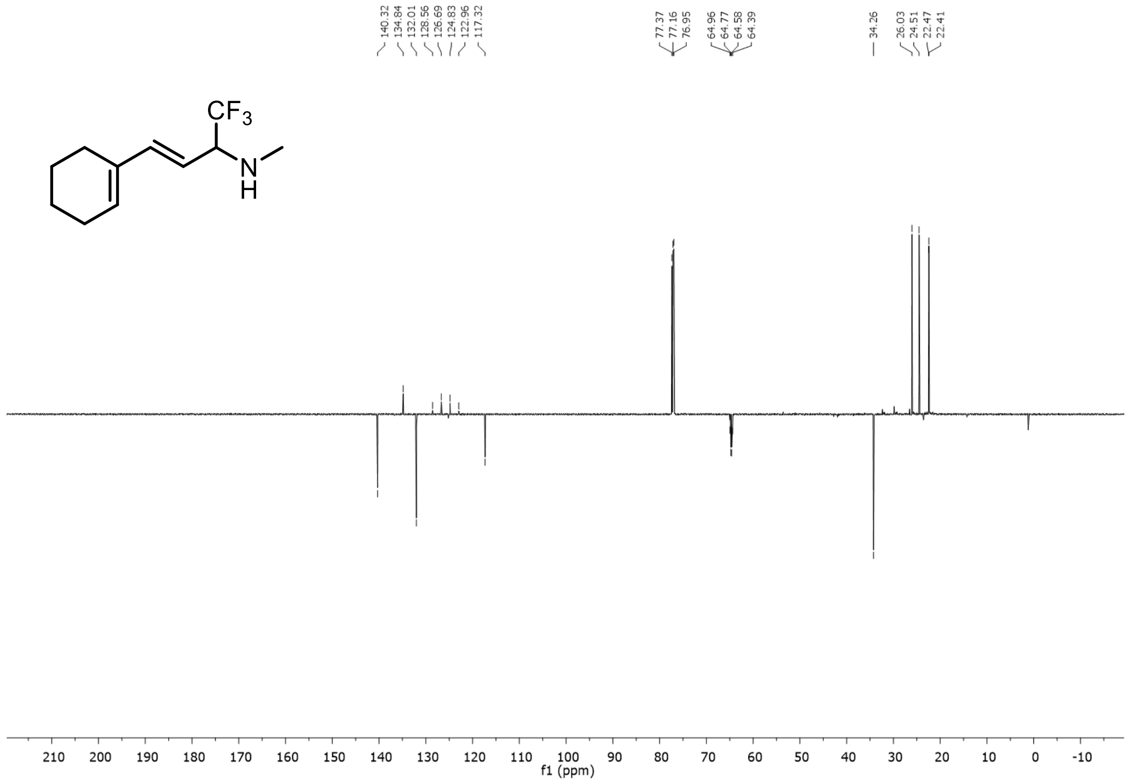

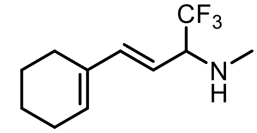
CF3
N
H

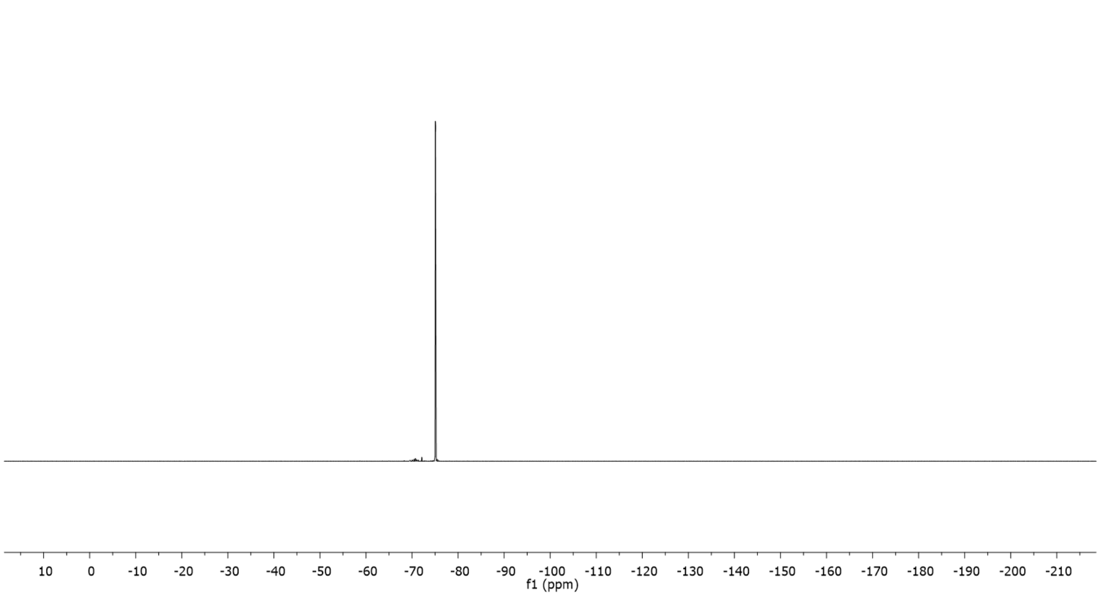
-75.06
-75.07
10 0 -10 -20 -30 -40 -50 -60 -70 -80 -90 -100 -110 -120 -130 -140 -150 -160 -170 -180 -190 -200 -210
f1 (ppm)

(*Z*)-1,1,1-Trifluoro-*N*-methyl-3-propylhept-3-en-2-amine (11a)

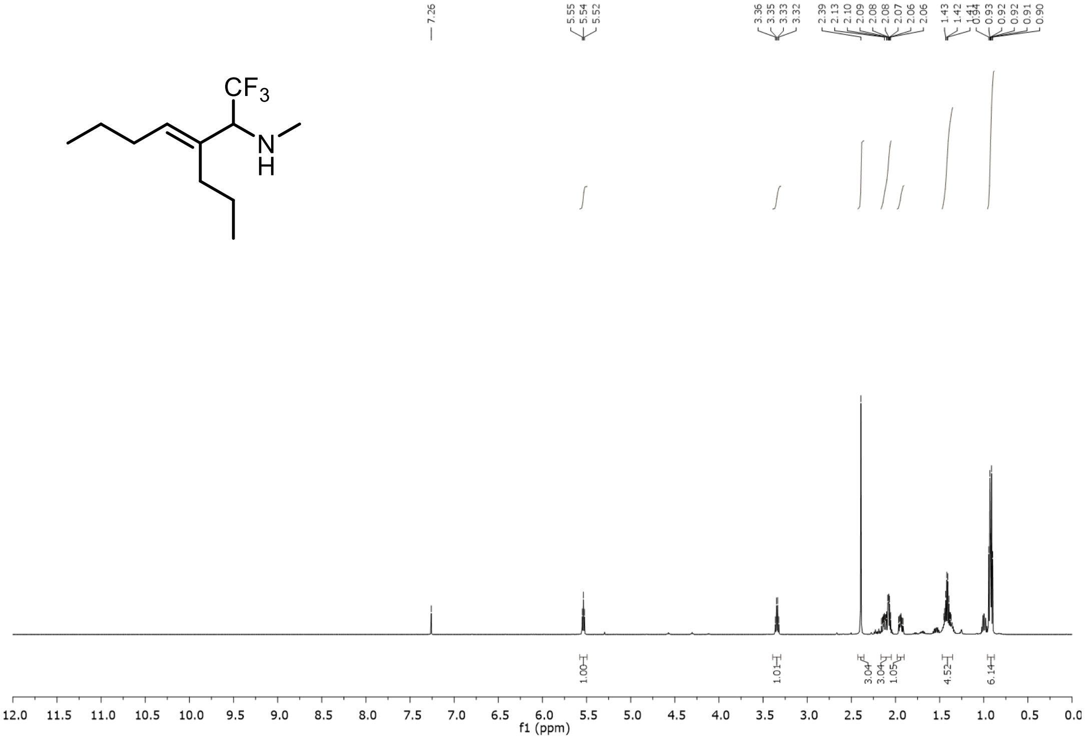

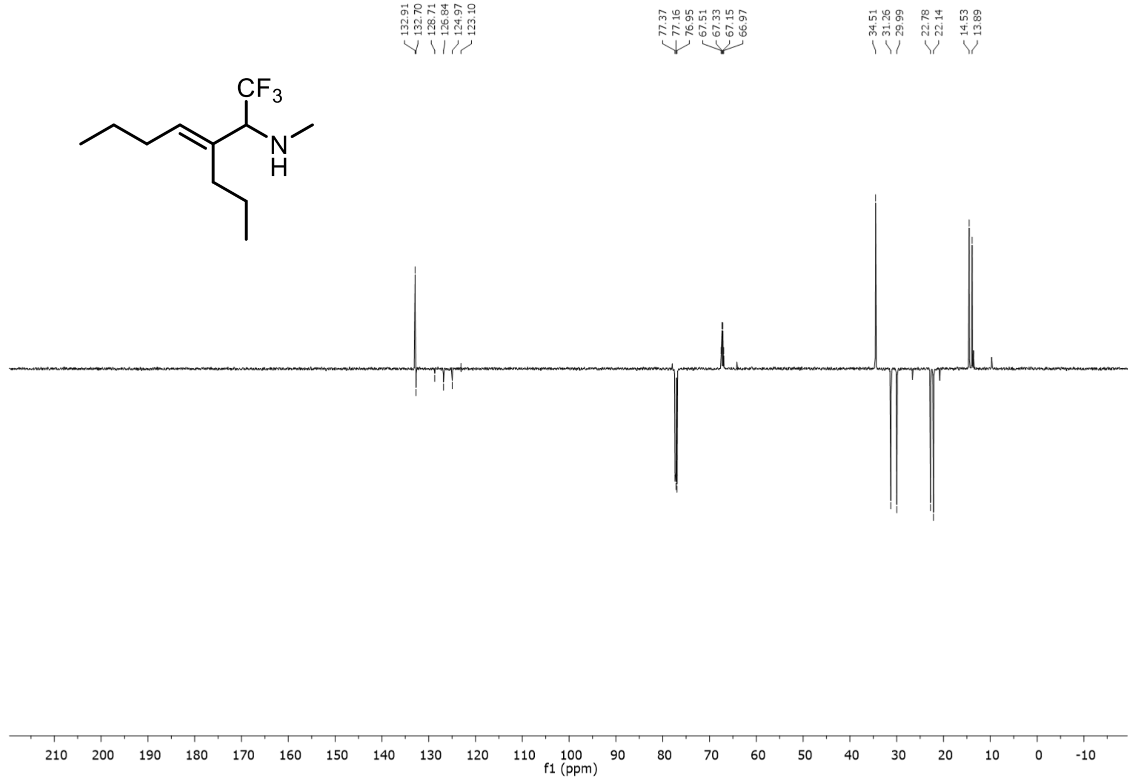

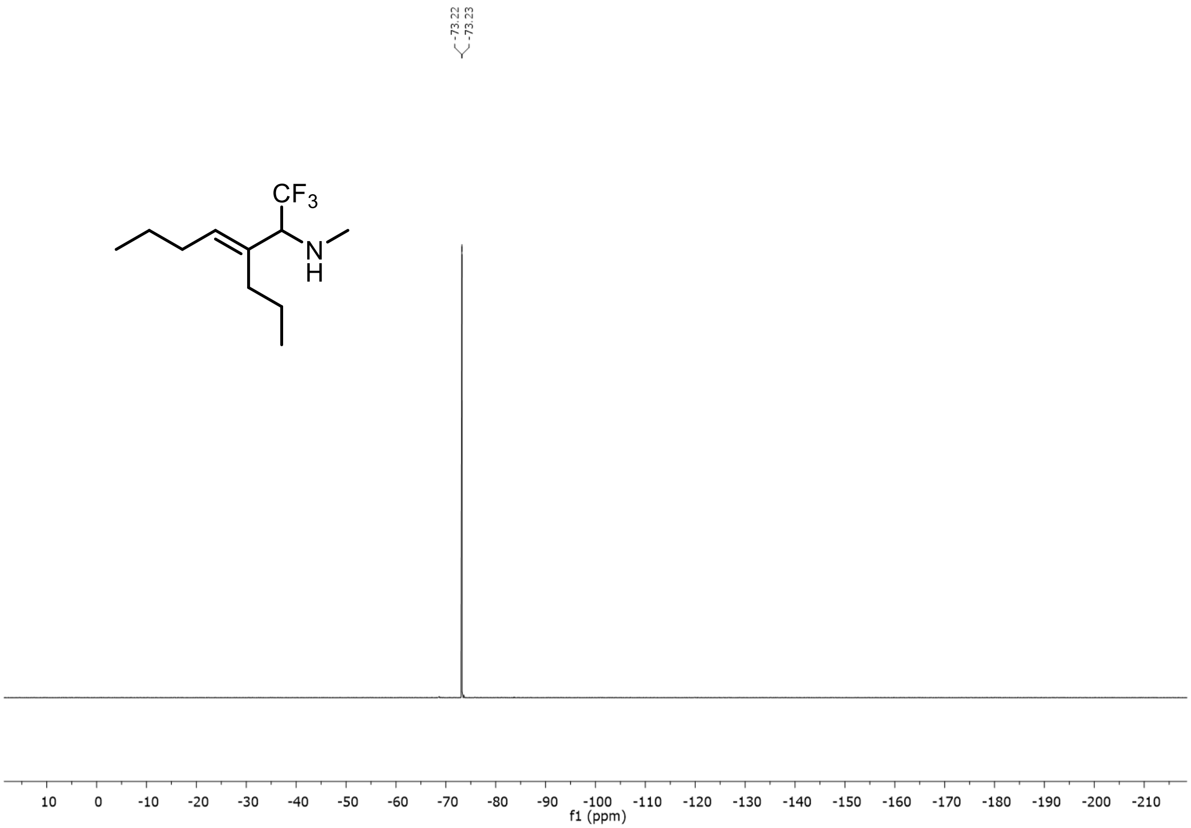
CF3
N
H
-73.22
-73.23
10 0 -10 -20 -30 -40 -50 -60 -70 -80 -90 -100 -110 -120 -130 -140 -150 -160 -170 -180 -190 -200 -210
f1 (ppm)

(*E*)-1,1,1-Trifluoro-N-methyldodec-3-en-7-yn-2-amine (12a)

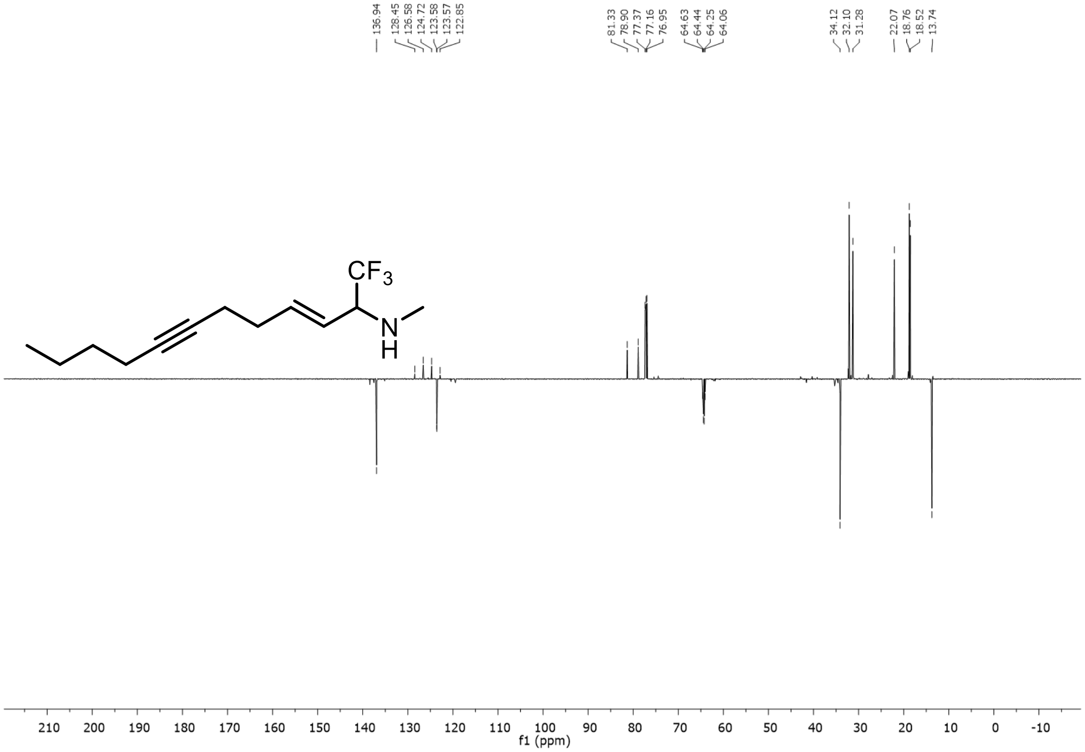

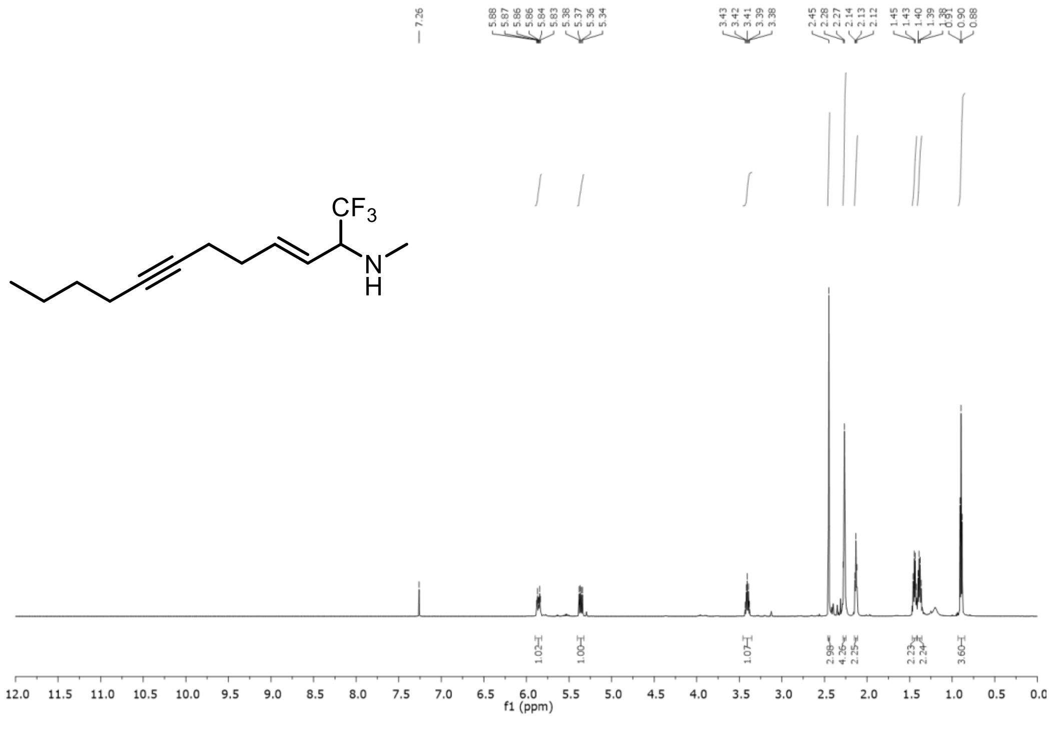

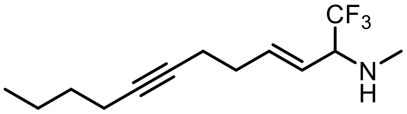

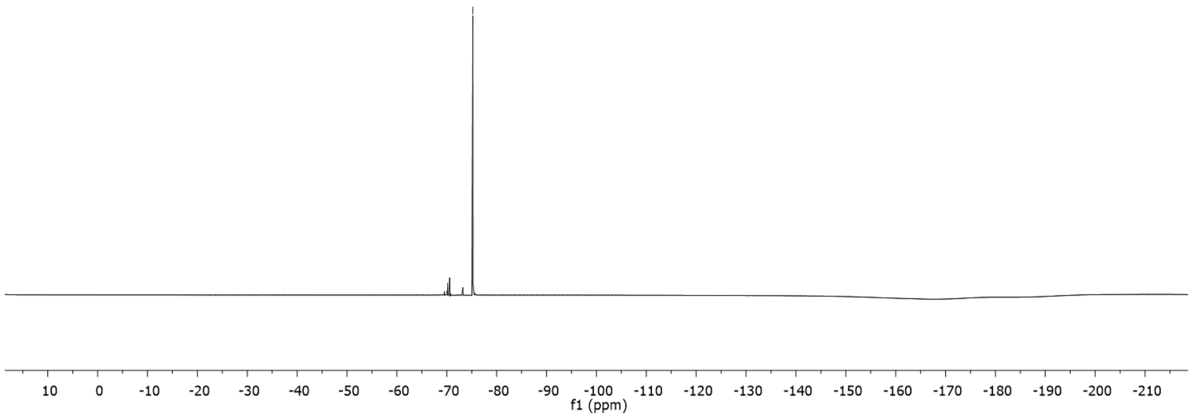
-75.21
-75.22
10
0
-10
-20
-30
-40
-50
-60
-70
-80
-90
-100
-110
-120
-130
-140
-150
-160
-170
-180
-190
-200
-210
f1 (ppm)

Methyl (*E*)-4-(4,4,4-trifluoro-3-(methylamino)but-1-en-1-yl)benzoate (13a)

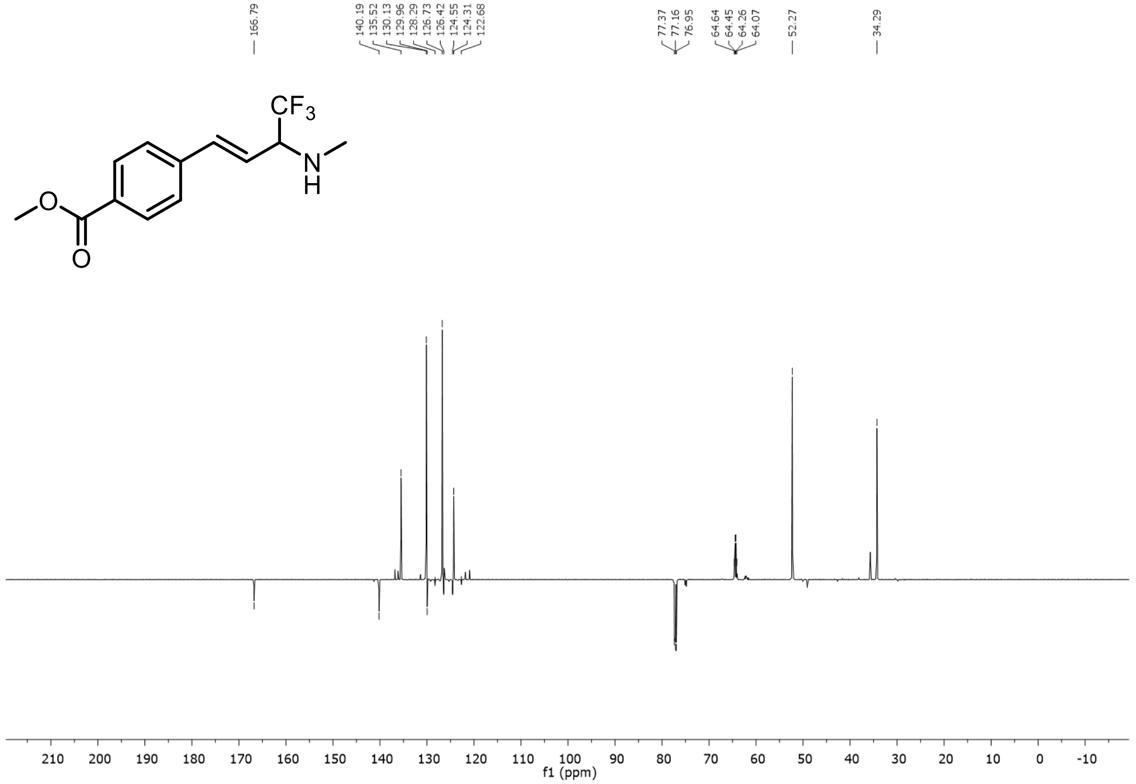

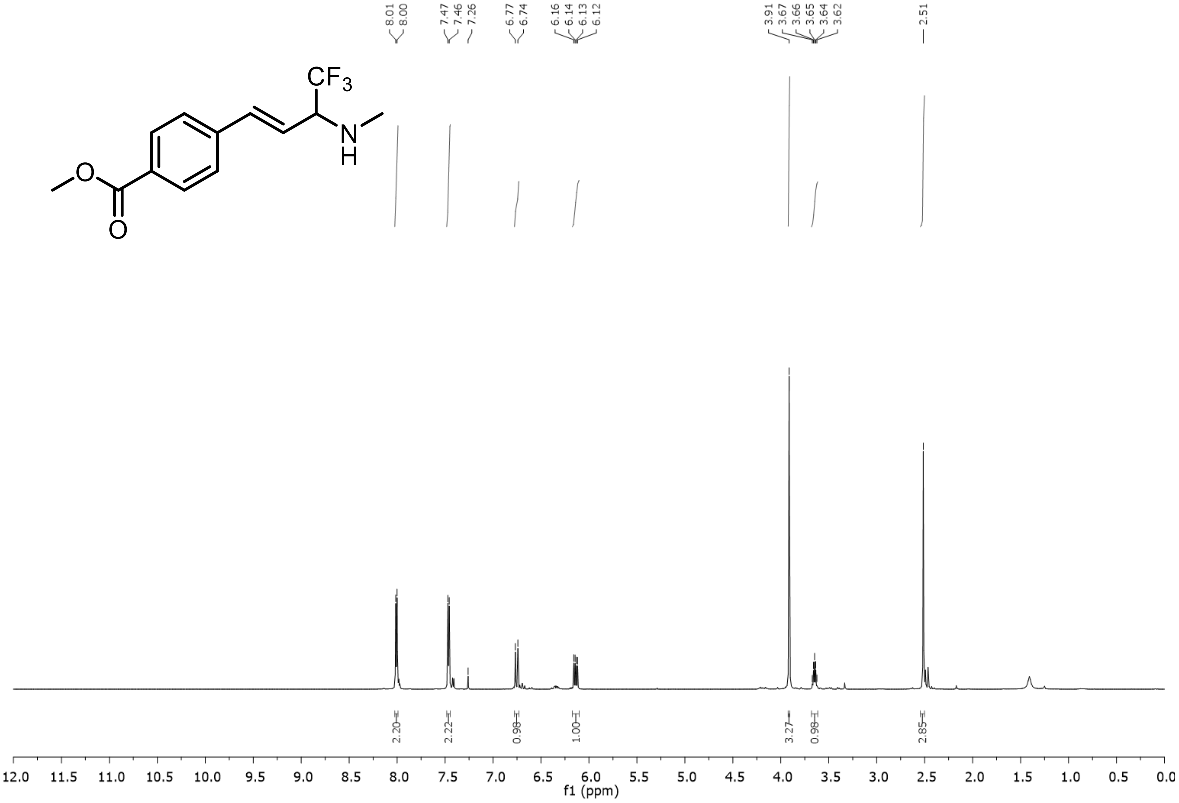

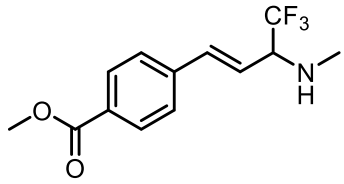

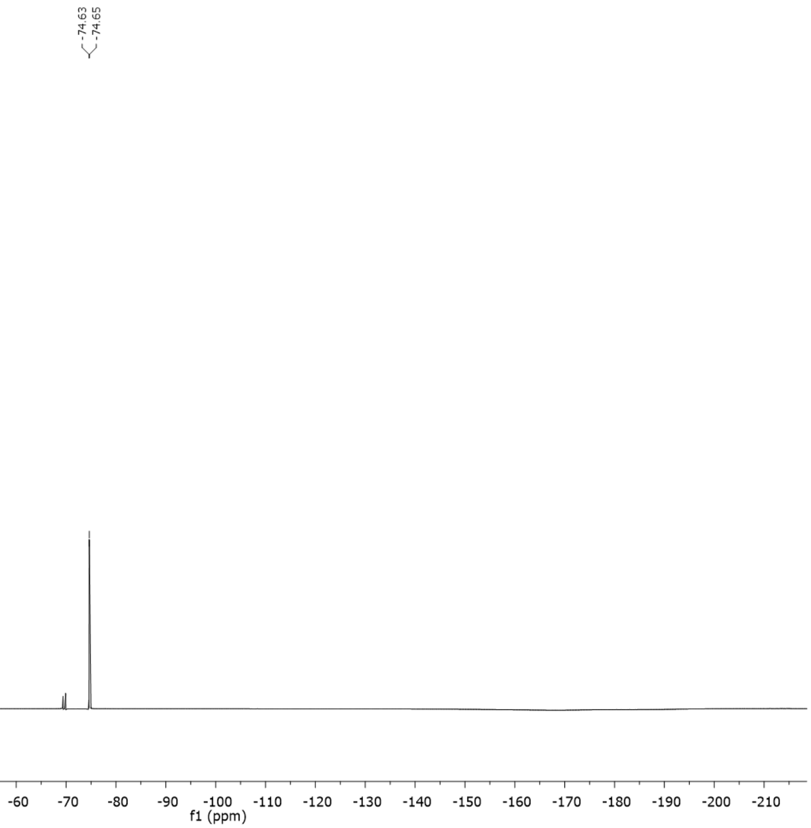
-74.63
-74.65
10
0
-10
-20
-30
-40
-50
-60
-70
-80
-90
-100
-110
-120
-130
-140
-150
-160
-170
-180
-190
-200
-210
f1 (ppm)

(E)-2-(8,8,8-Trifluoro-7-(methylamino)oct-5-en-1-yl)isoindoline-1,3-dione (14a)

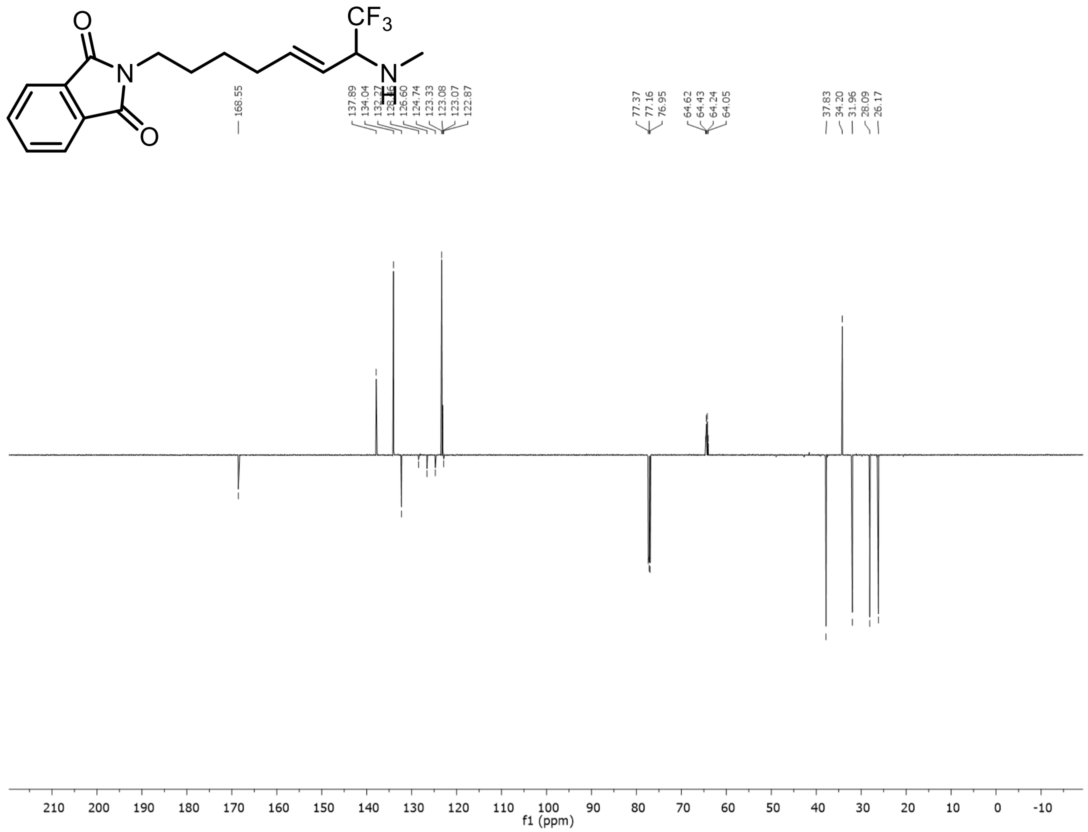

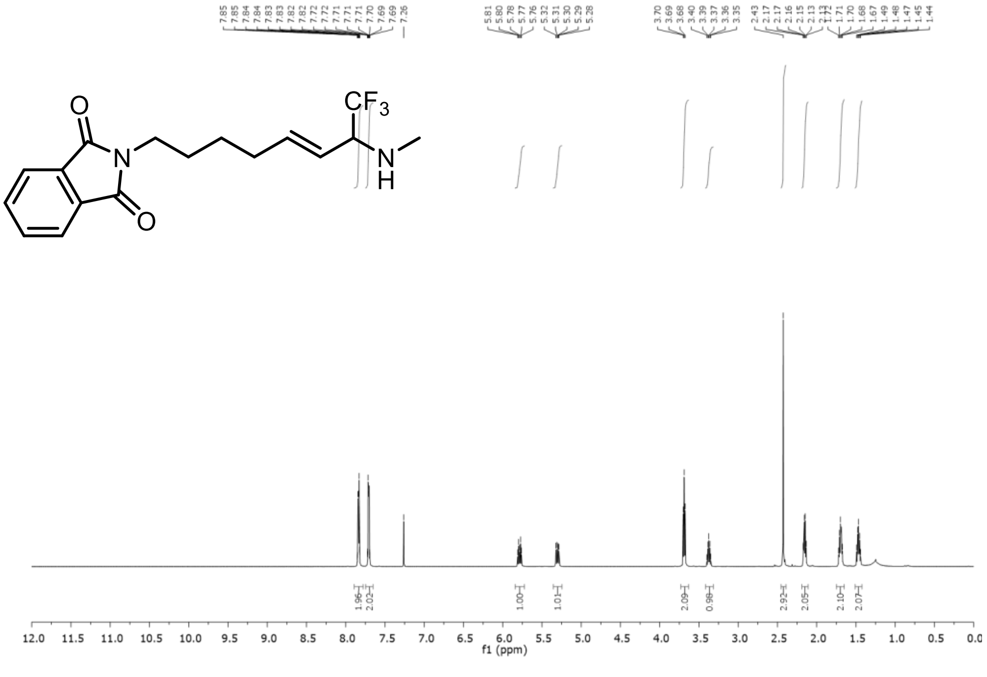

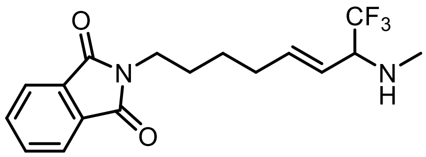

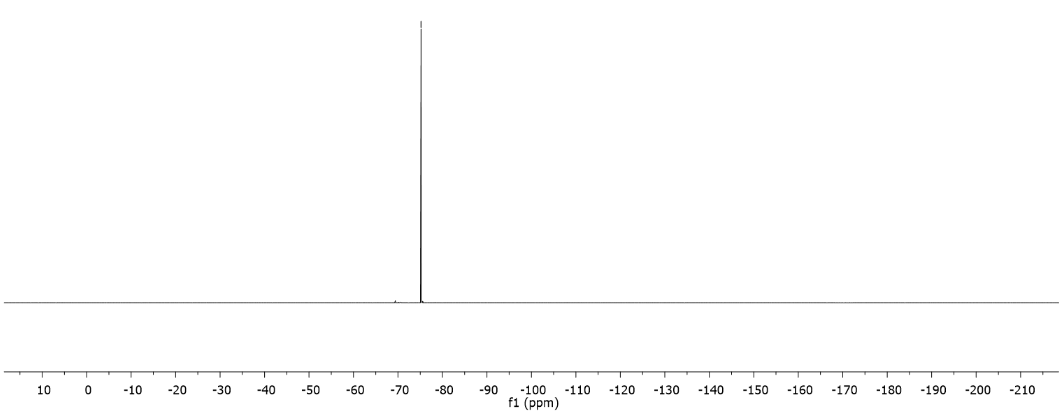

-75.18
-75.19
10
0
-10
-20
-30
-40
-50
-60
-70
-80
-90
-100
-110
-120
-130
-140
-150
-160
-170
-180
-190
-200
-210
f1 (ppm)

N^1-(7-chloroquinolin-4-yl)-5,5,5-trifluoro-N^4-methylpentane-1,4-diamine (15a)

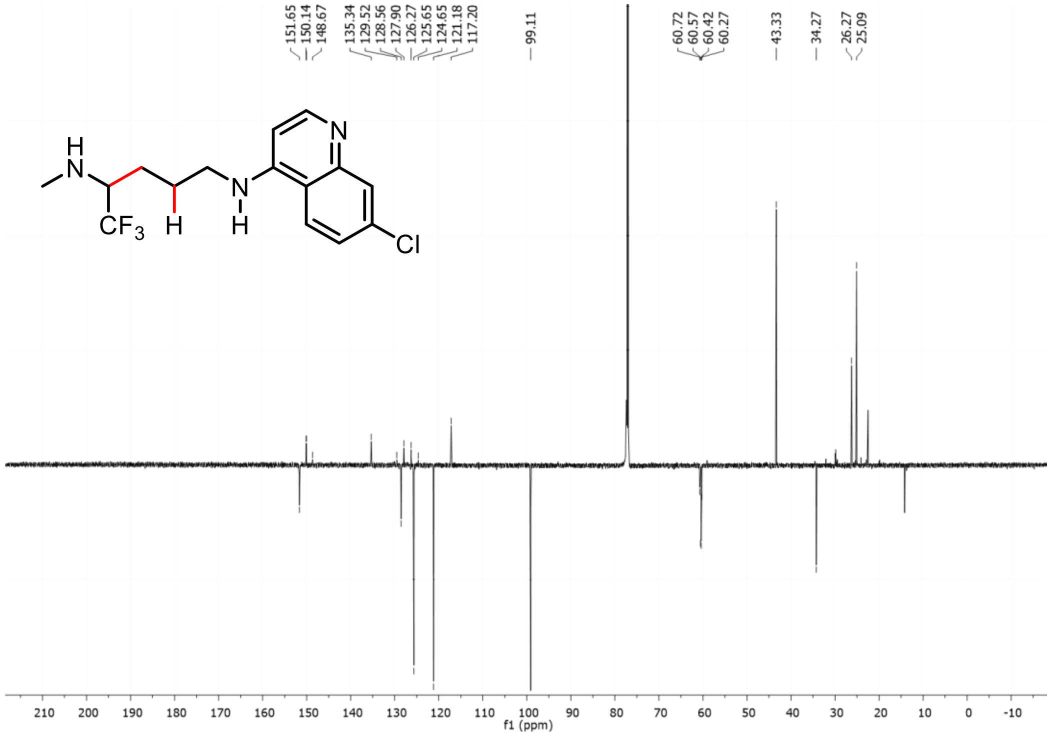

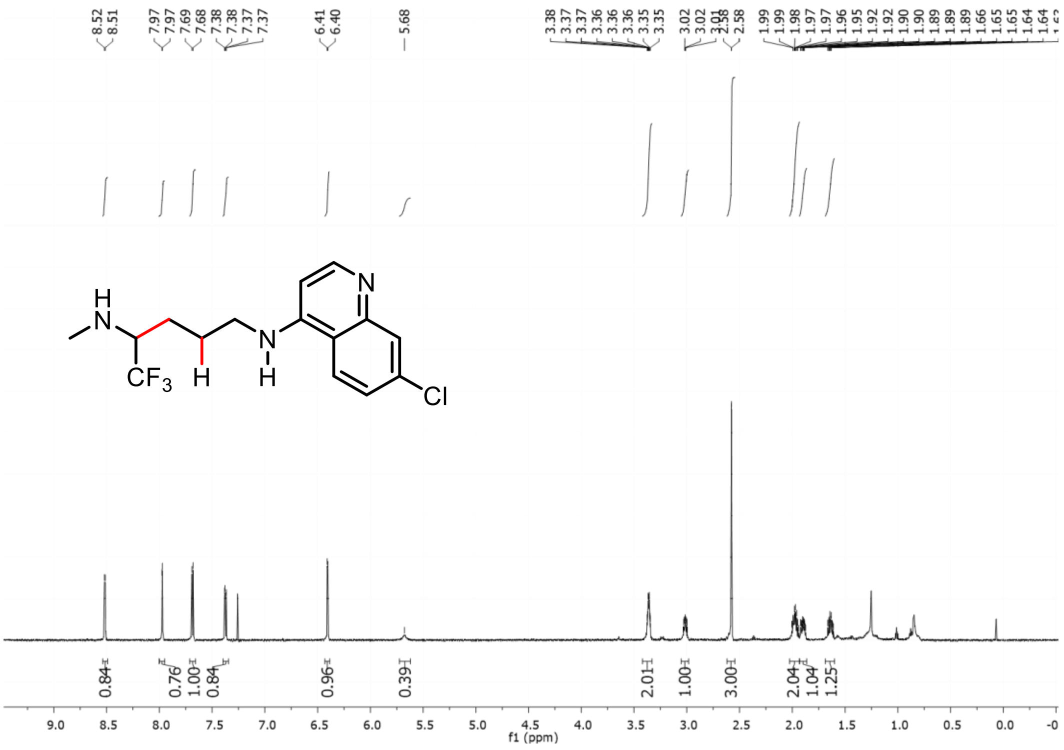

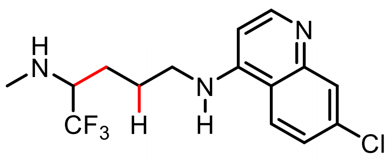

H
N
CF3
H
N
H
N
Cl

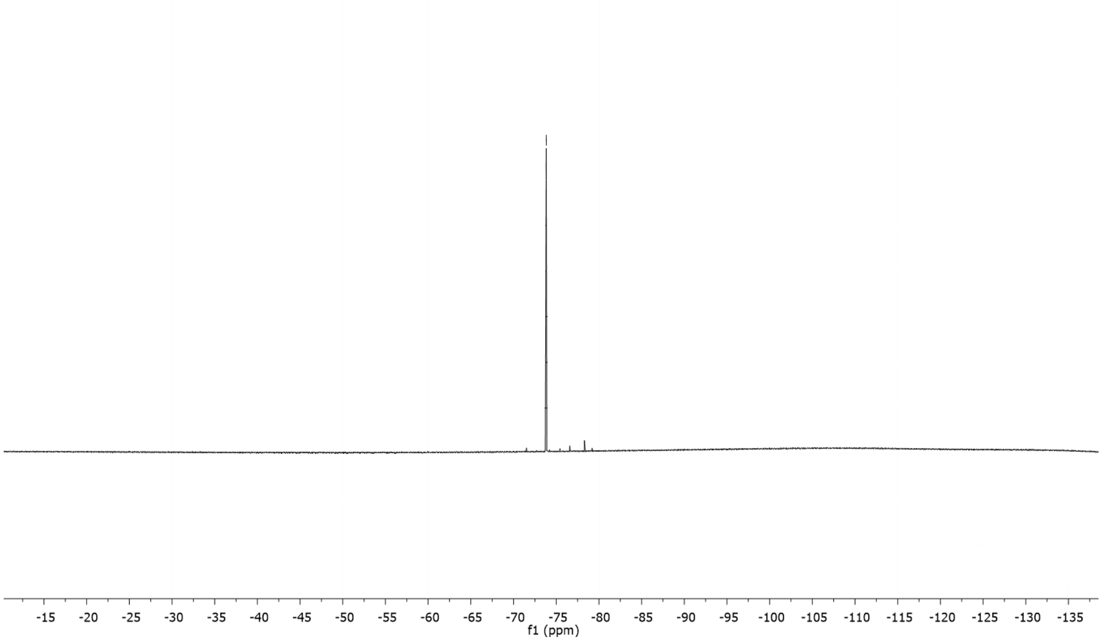

-73.83
-73.85
-15 -20 -25 -30 -35 -40 -45 -50 -55 -60 -65 -70 -75 -80 -85 -90 -95 -100 -105 -110 -115 -120 -125 -130 -135
f1 (ppm)

4-(4-chlorophenyl)-1,1,1-trifluoro-*N,N*-dimethyl-4-(pyridin-2-yl)butan-2-amine (16a)

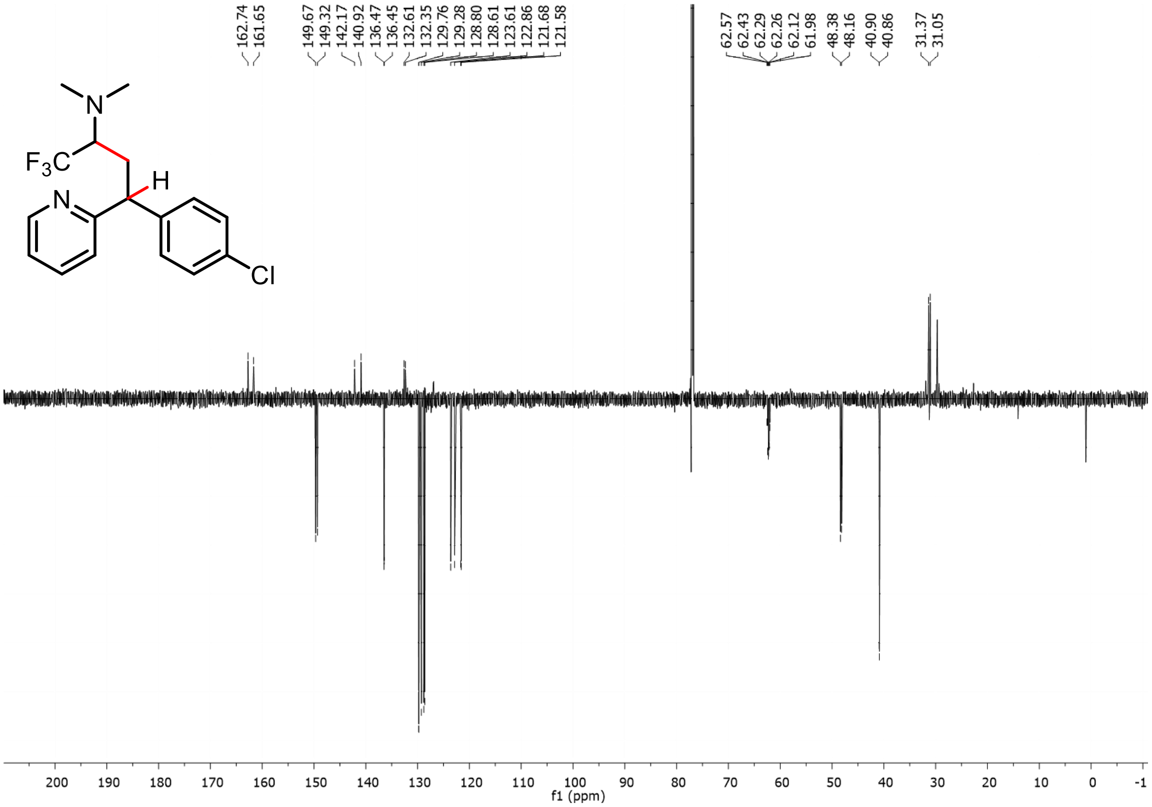

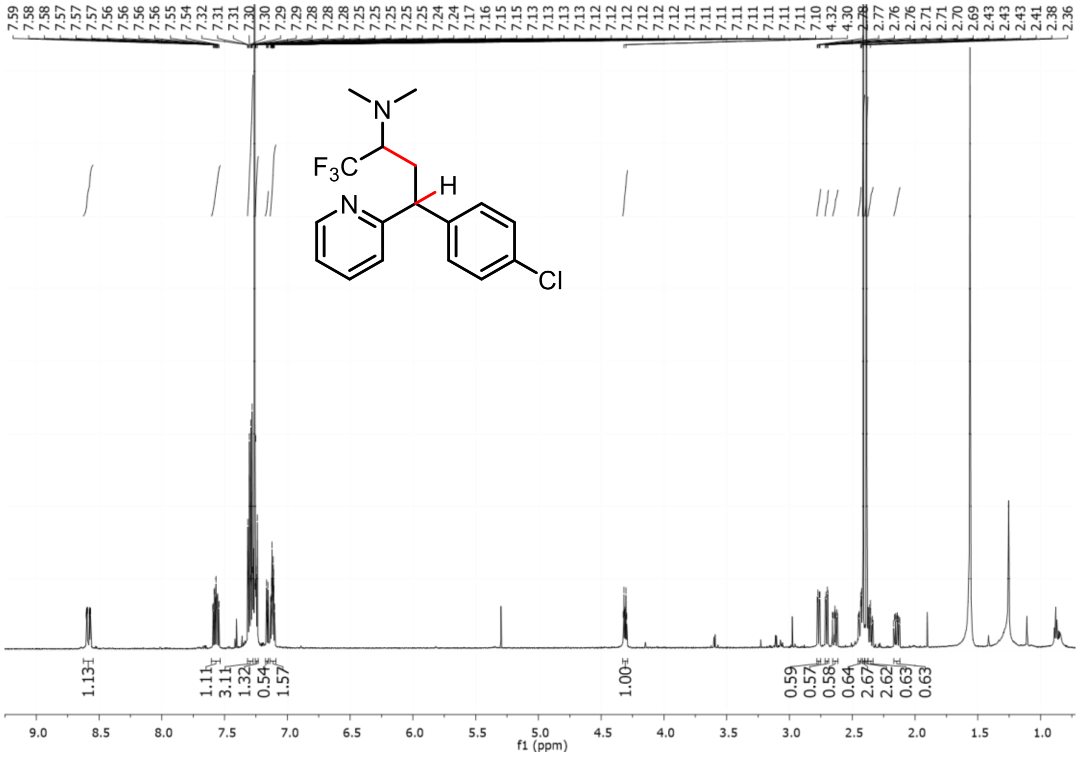

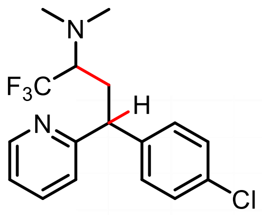

F3C
N
H
N
Cl

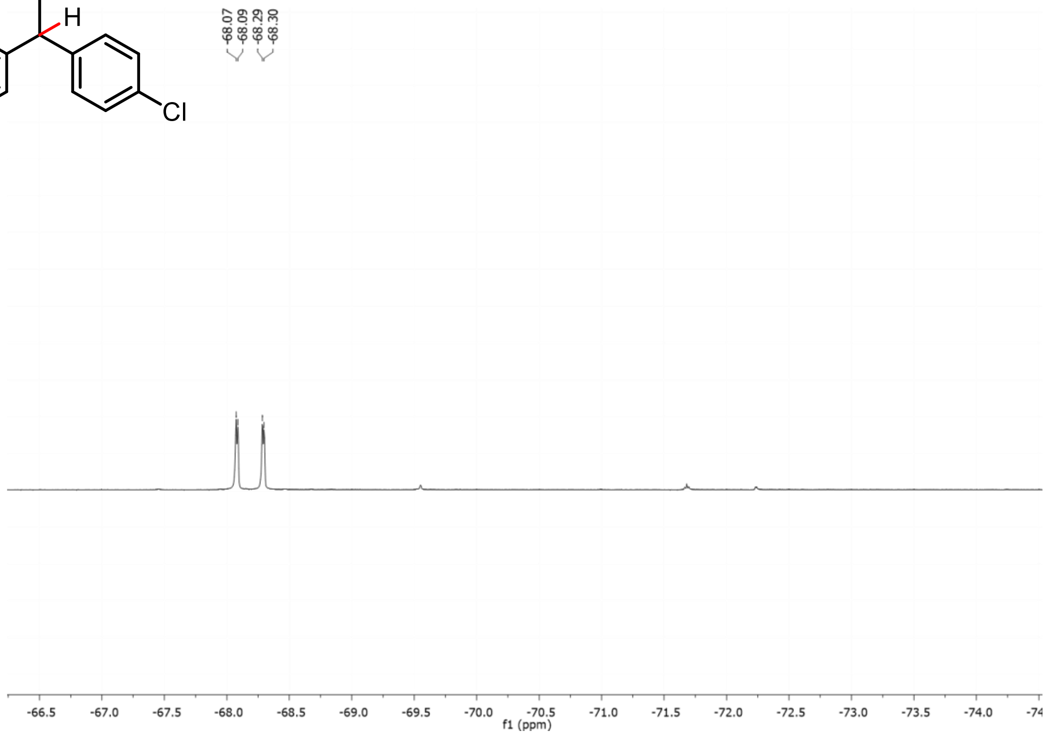

-68.07
-68.09
-68.29
-68.30
-66.5
-67.0
-67.5
-68.0
-68.5
-69.0
-69.5
-70.0
-70.5
-71.0
-71.5
-72.0
-72.5
-73.0
-73.5
-74.0
f1 (ppm)

***(1R)*-(6-methoxyquinolin-4-yl)((1S,2R,4S,5R)-5-(4,4,4-trifluoro-3-(methylamino)butyl)quinuclidin-2-yl)methanol (17a)**

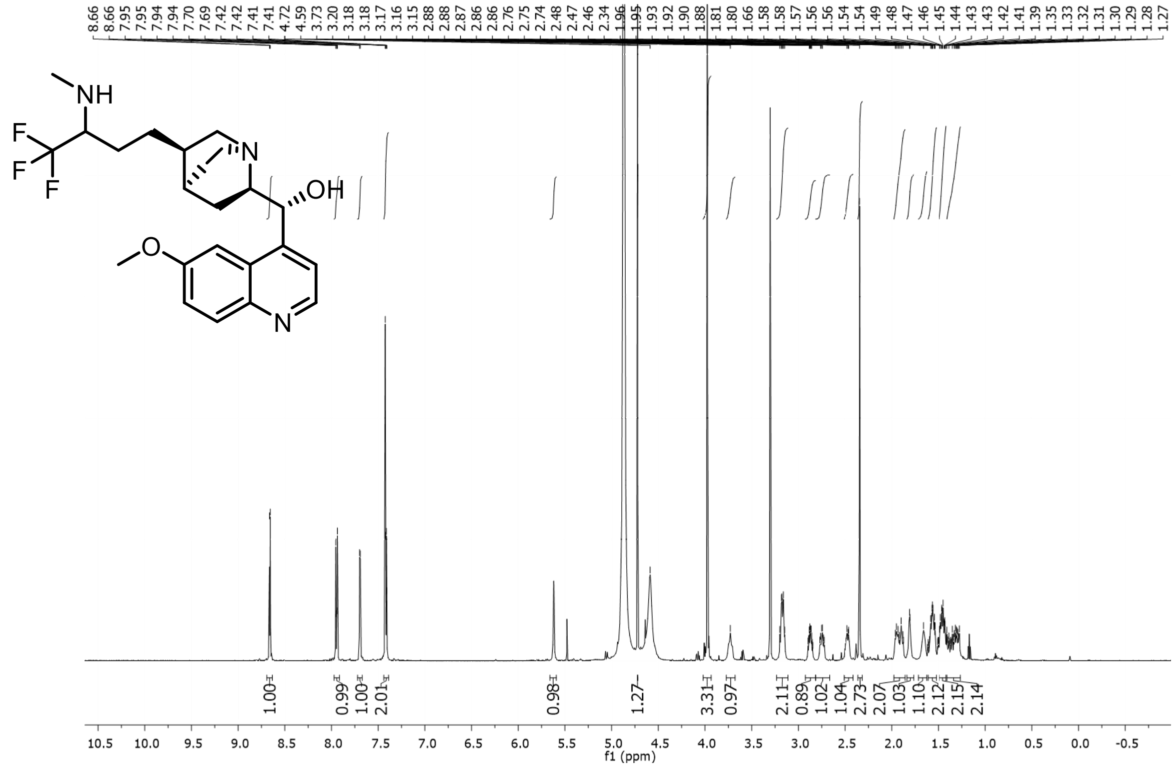

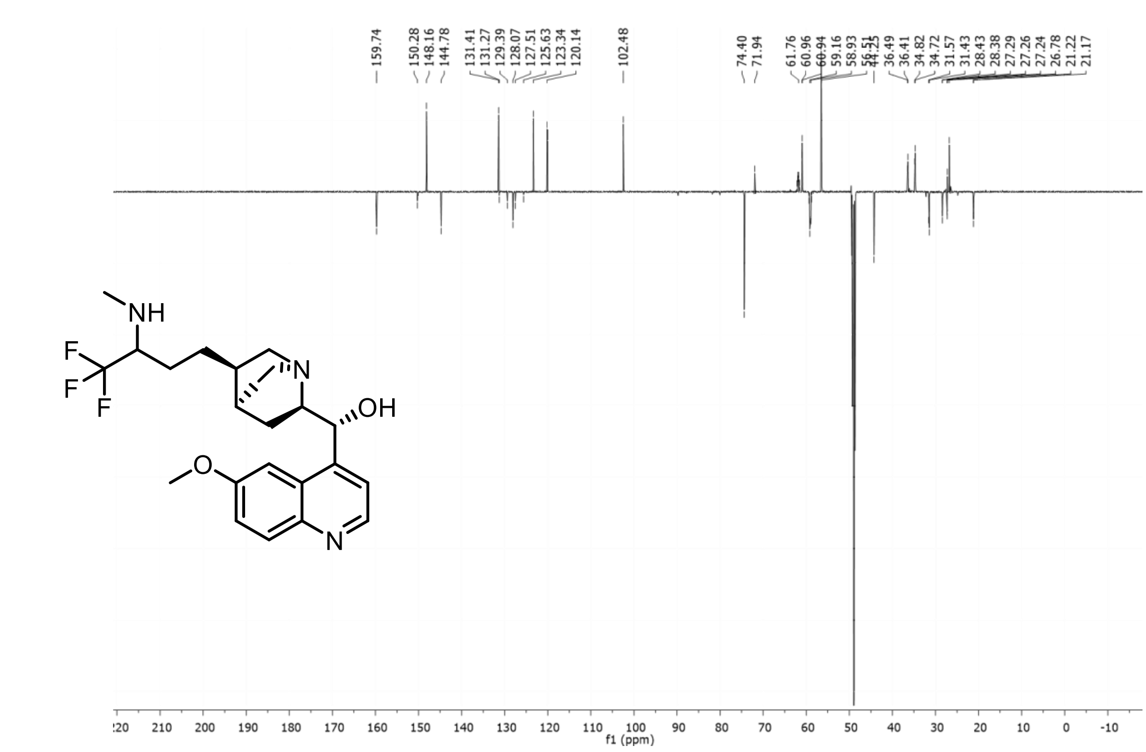

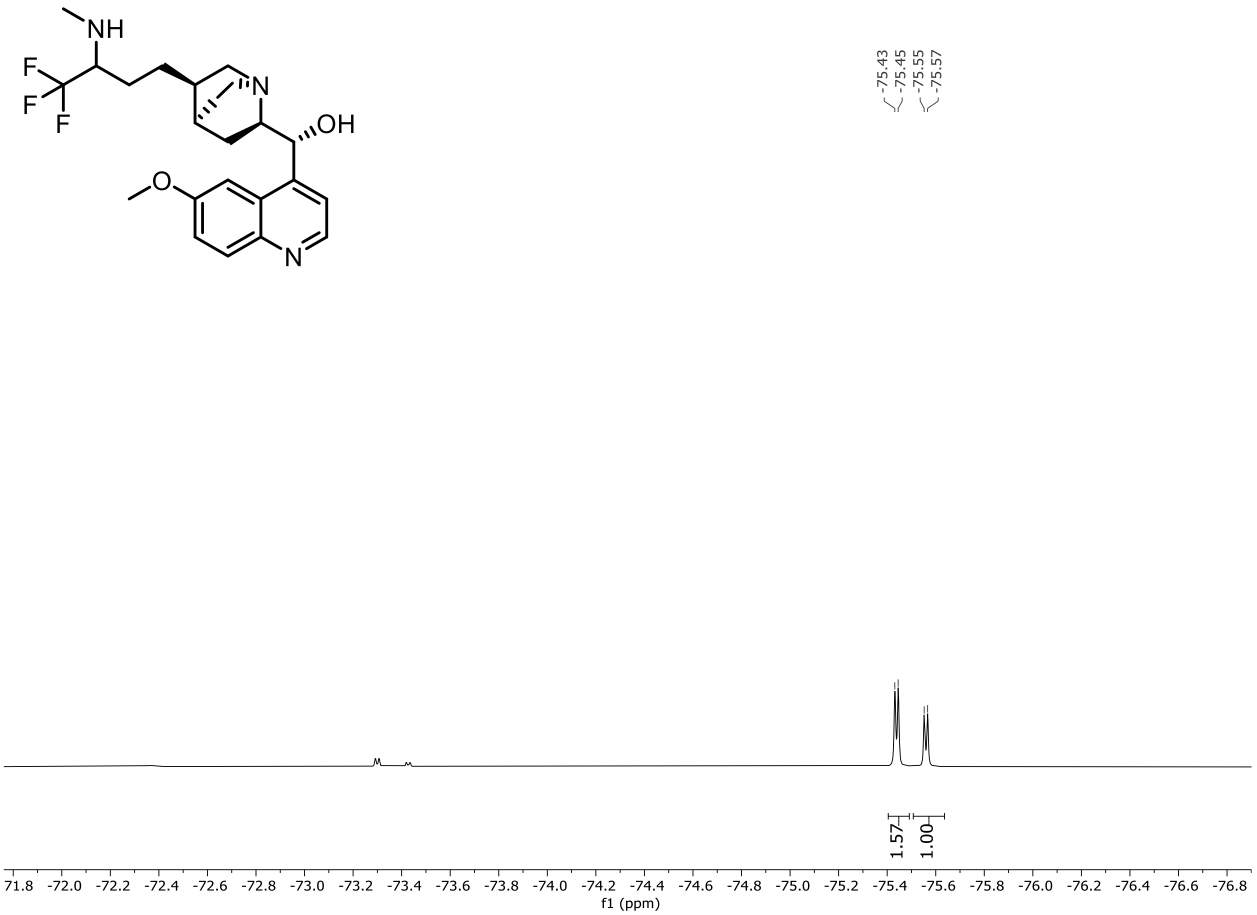
NH
F
F
F
N
OH
O
N
-75.43
-75.45
-75.55
-75.57
1.57
1.00
71.8 -72.0 -72.2 -72.4 -72.6 -72.8 -73.0 -73.2 -73.4 -73.6 -73.8 -74.0 -74.2 -74.4 -74.6 -74.8 -75.0 -75.2 -75.4 -75.6 -75.8 -76.0 -76.2 -76.4 -76.6 -76.8
f1 (ppm)

Ethyl 2-(methylamino)-4-phenylbutanoate (1b)

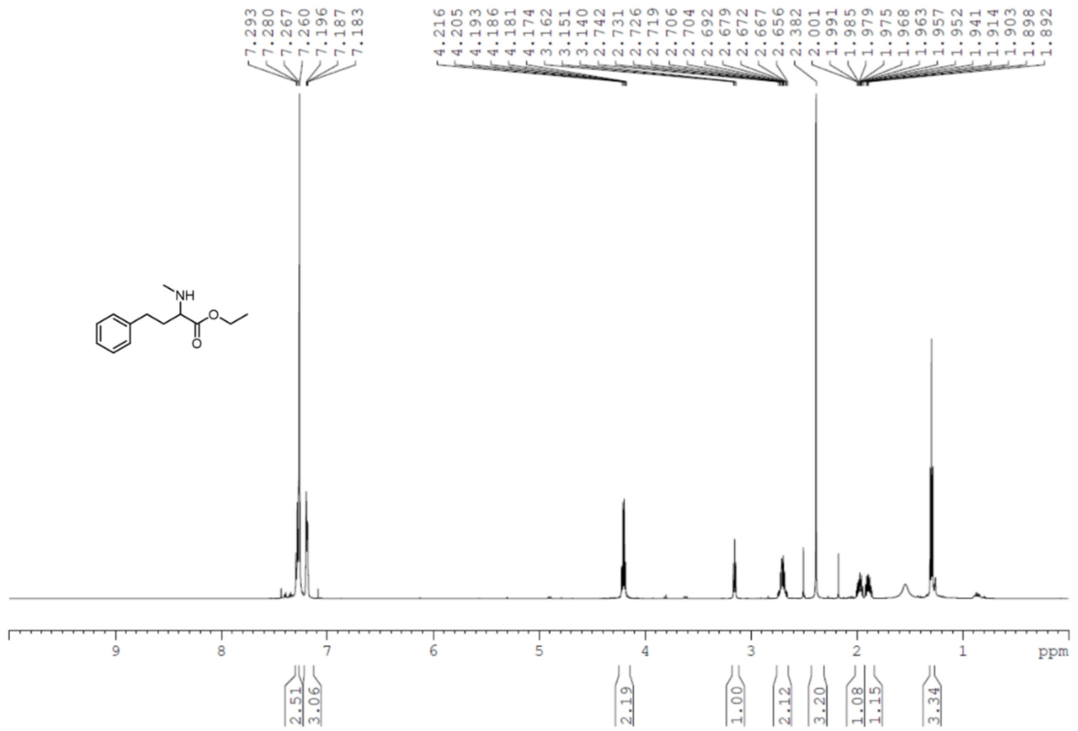

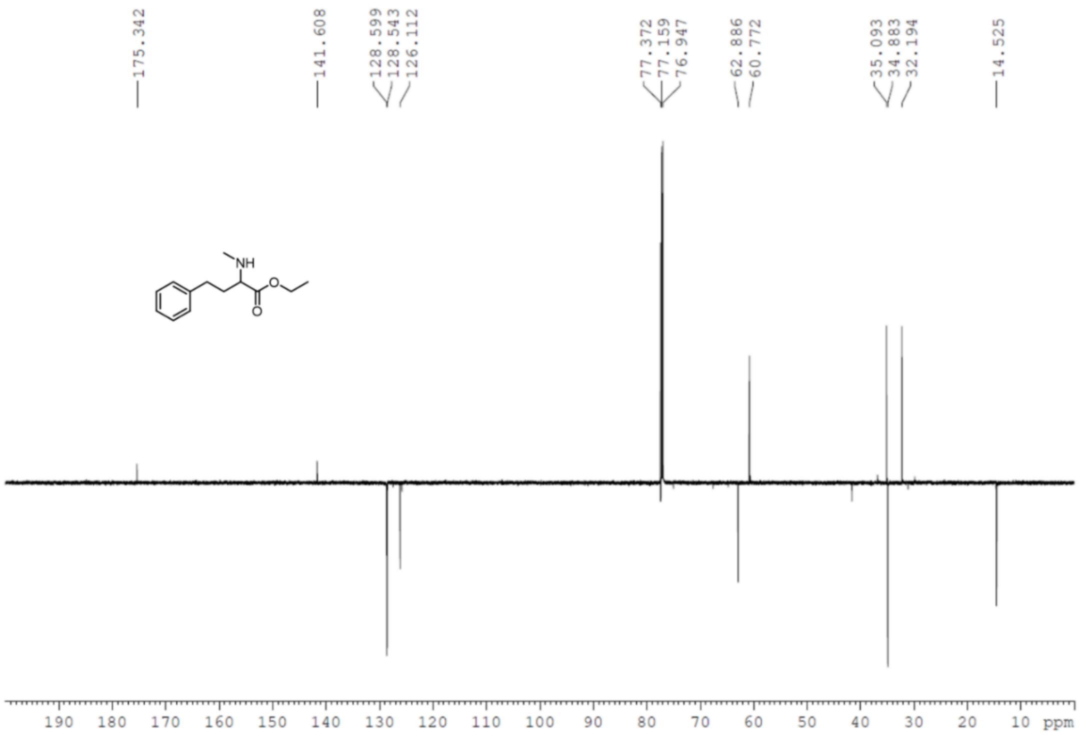

ethyl 2-(methylamino)-4-(p-tolyl)butanoate (2b)

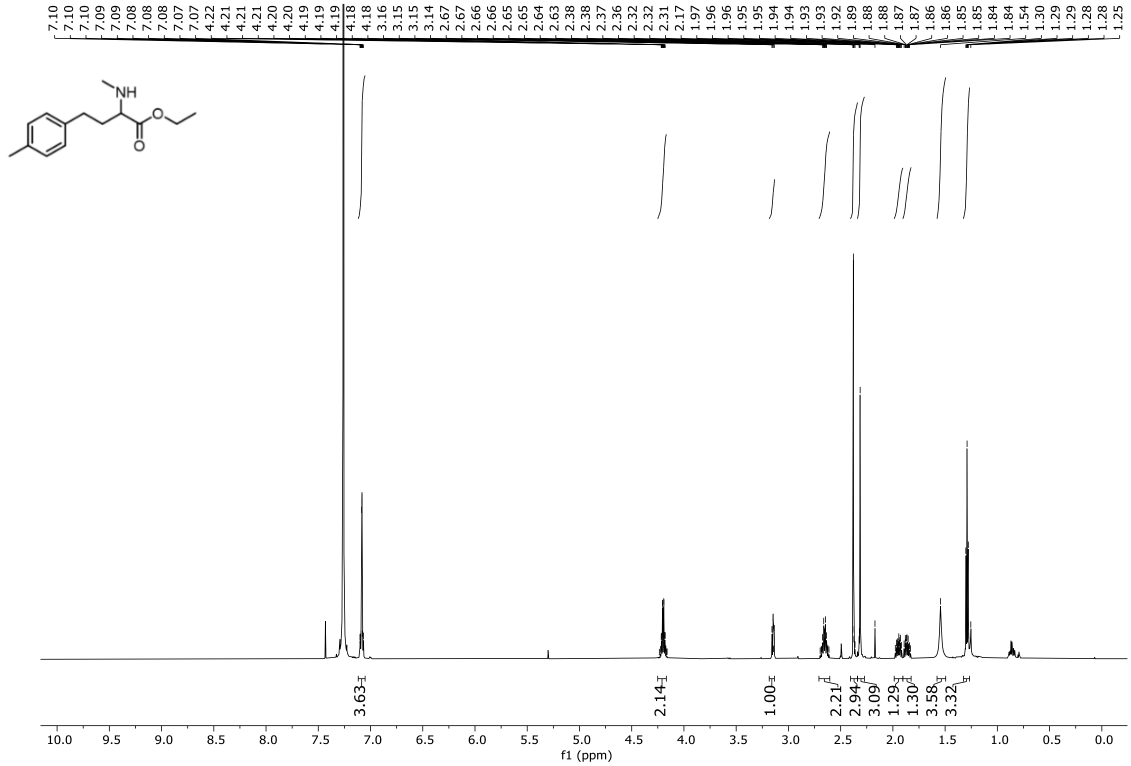

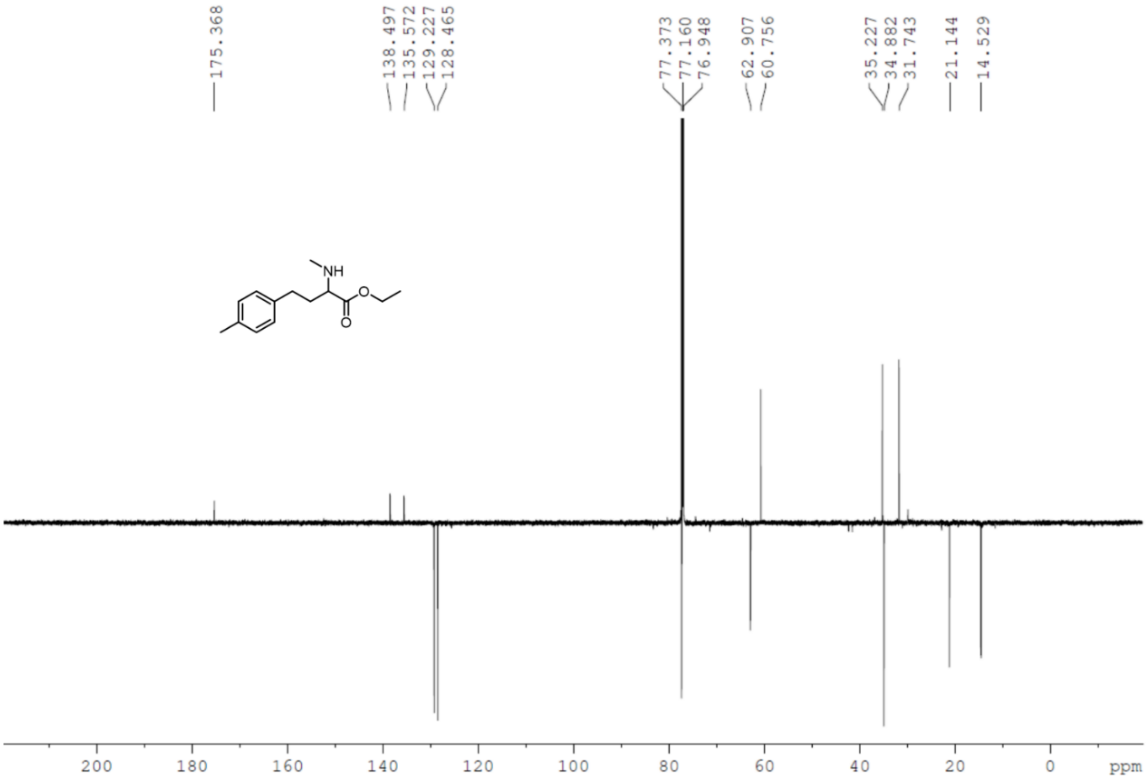

ethyl 4-(4-bromophenyl)-2-(methylamino)butanoate (3b)

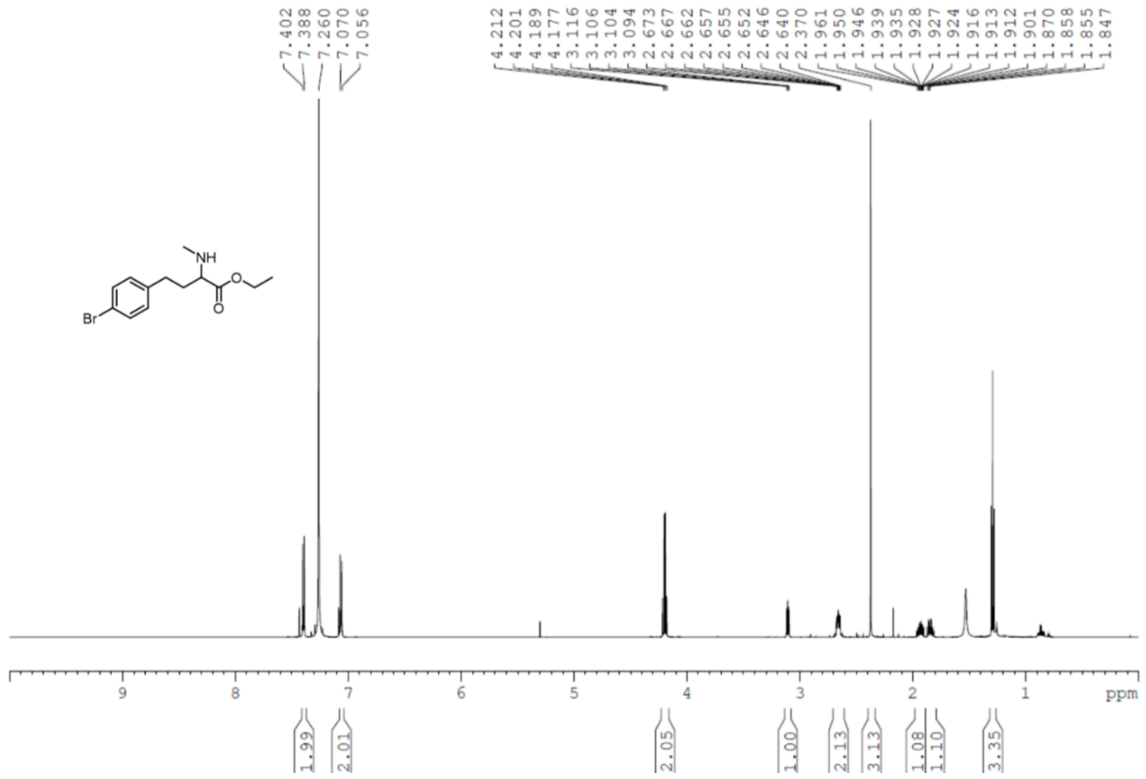

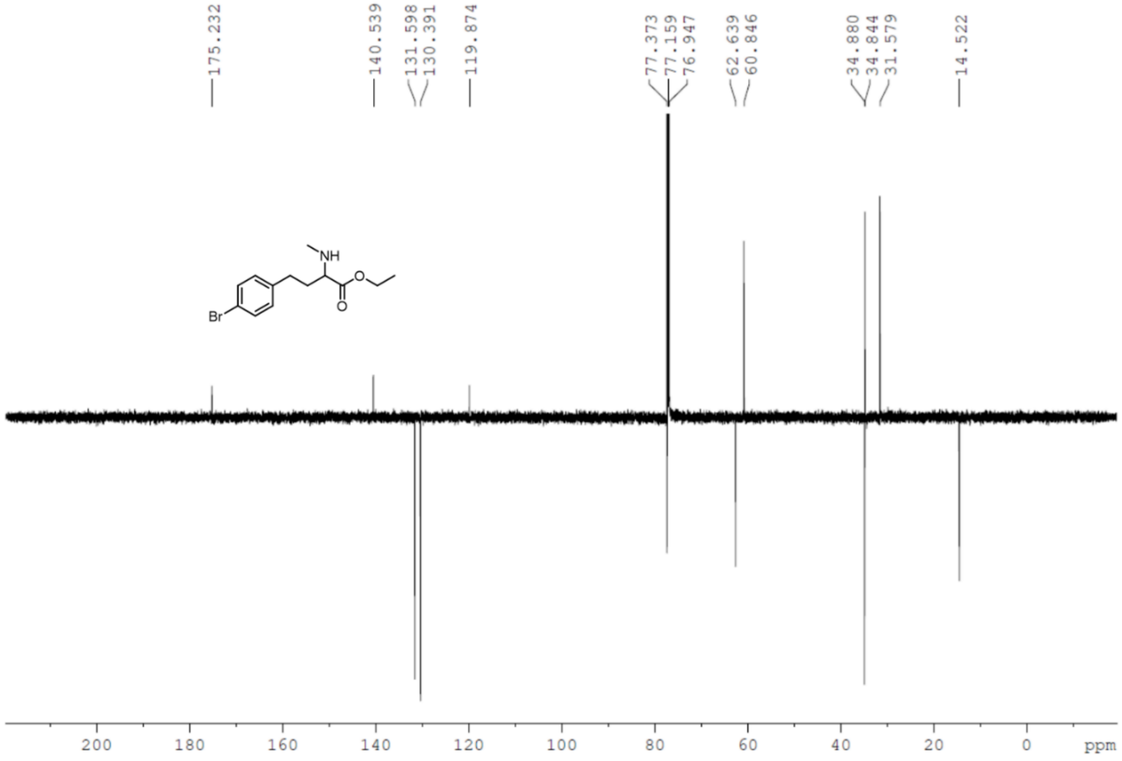

ethyl 2-(methylamino)undecanoate (4b)

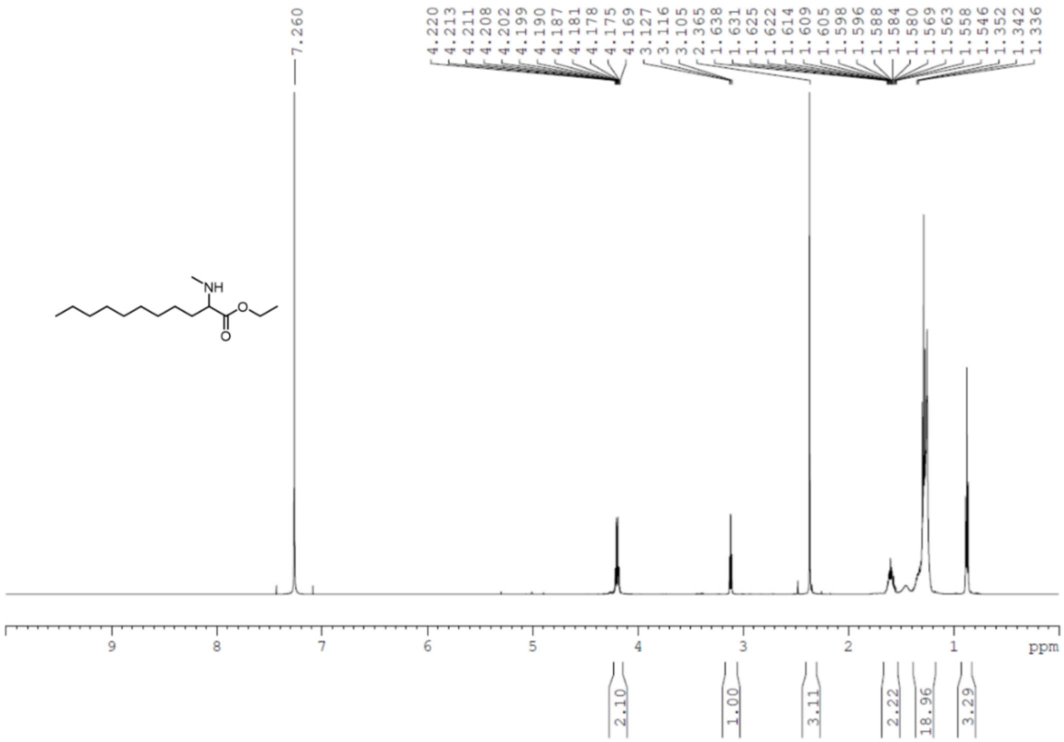

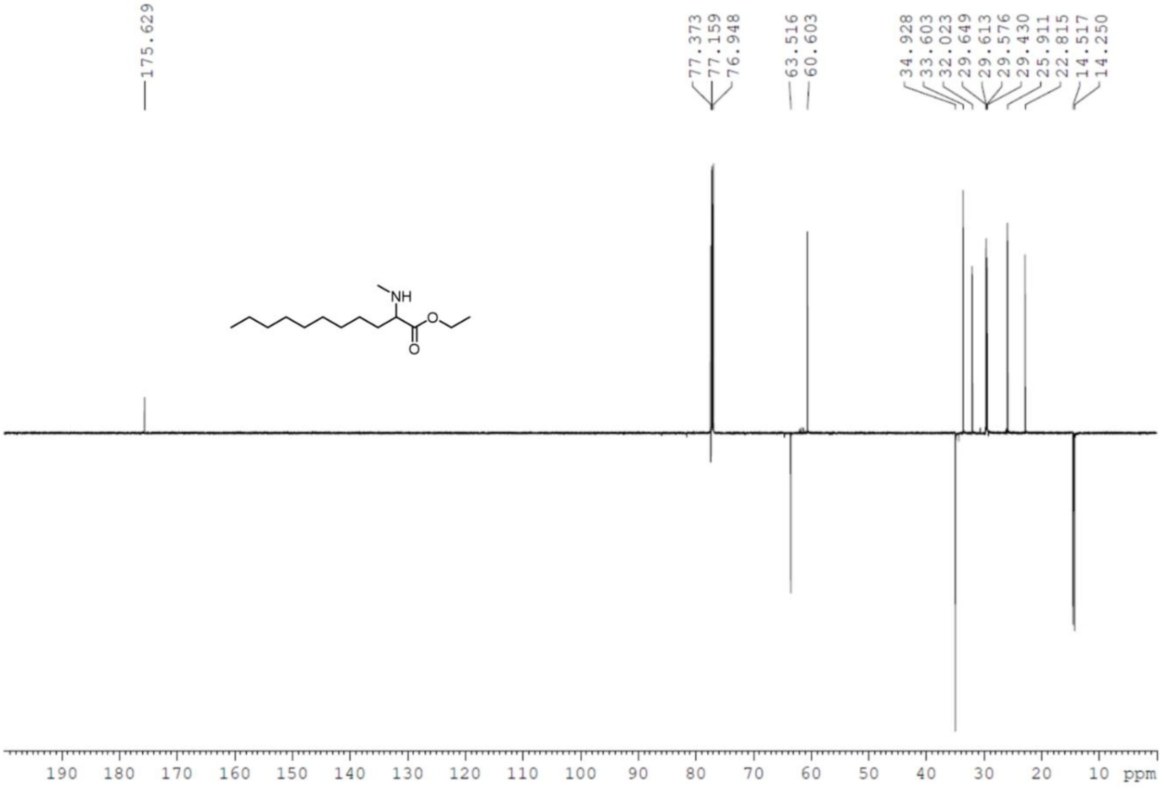

ethyl 12-hydroxy-2-(methylamino)dodecanoate (5b)

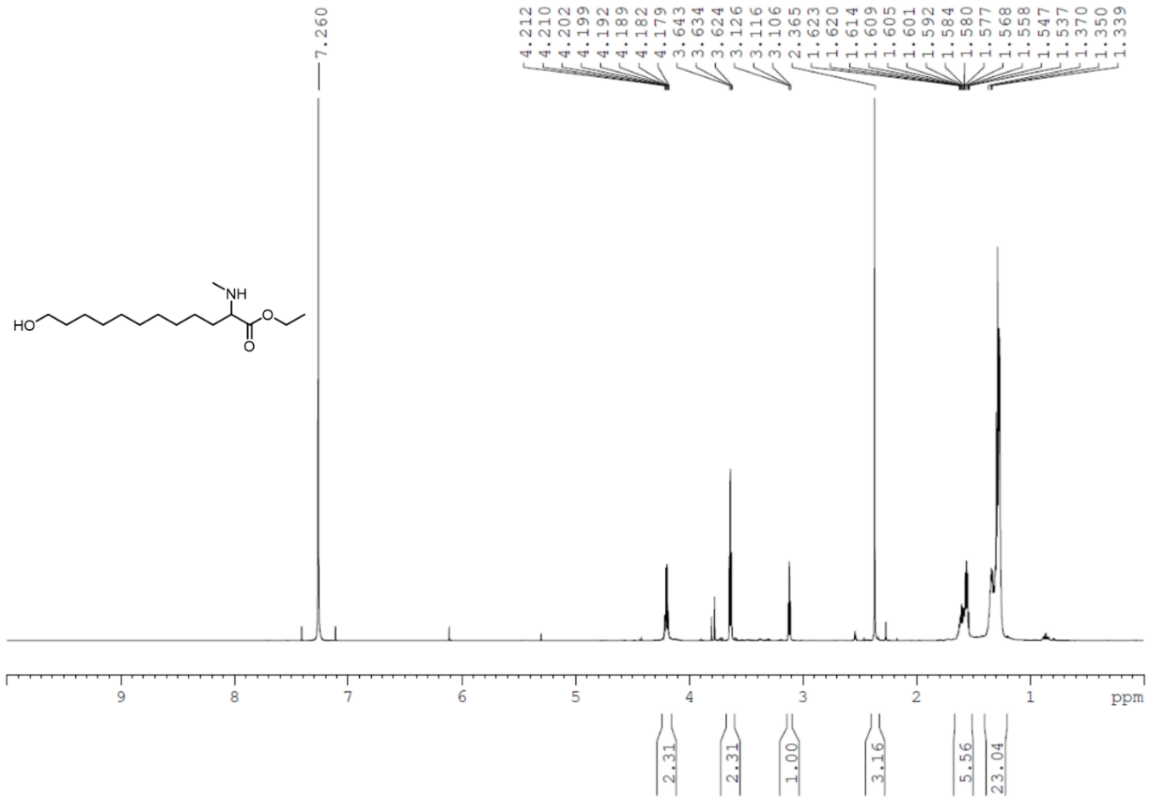

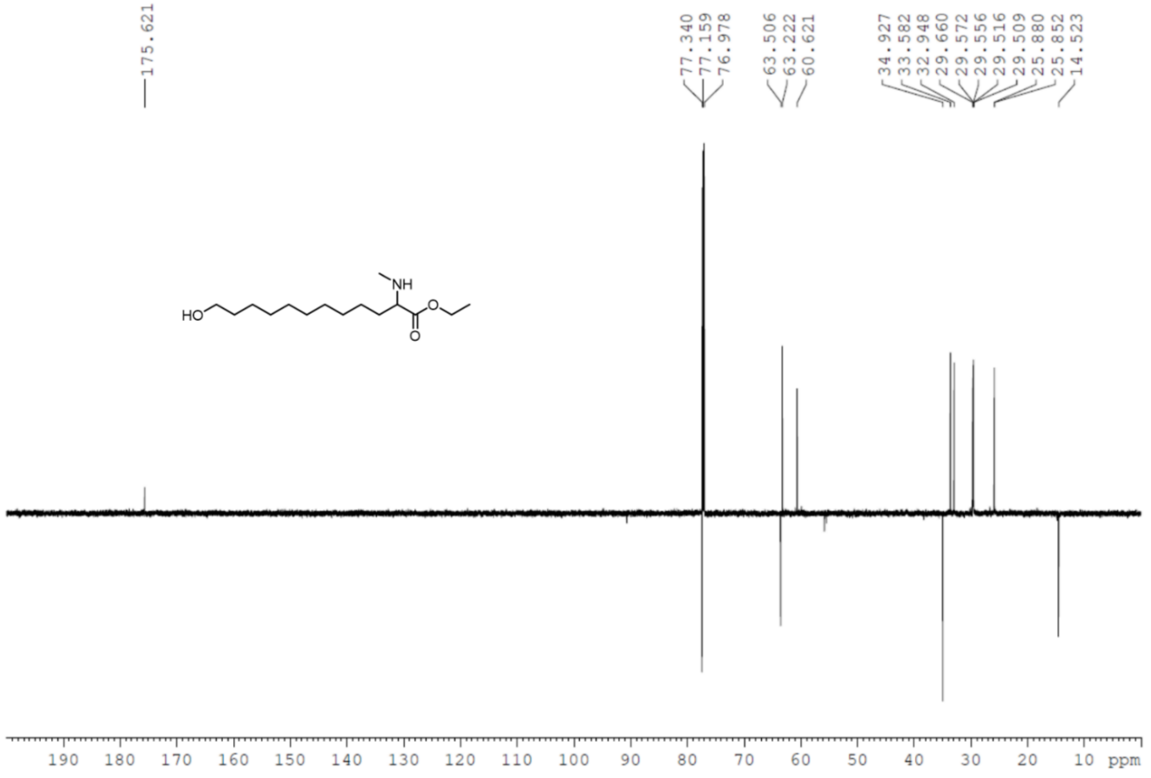

Ethyl 2-(methylamino)-6-phenylhexanoate (6b)

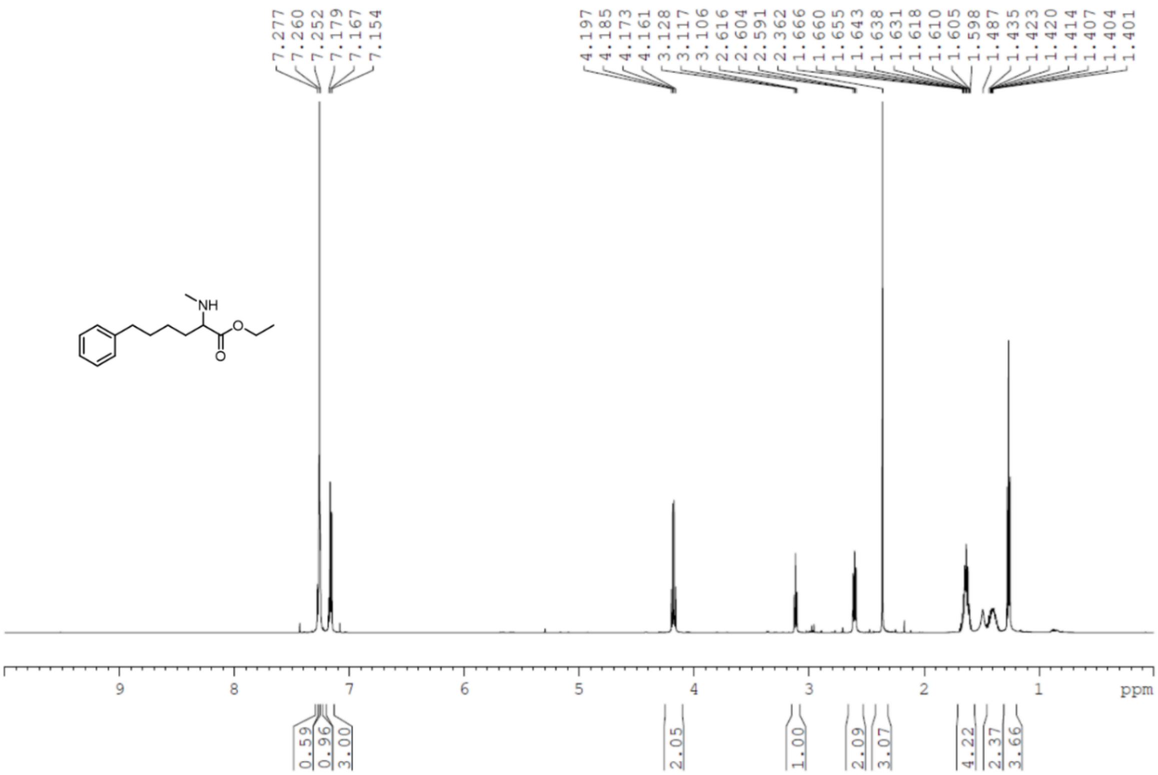

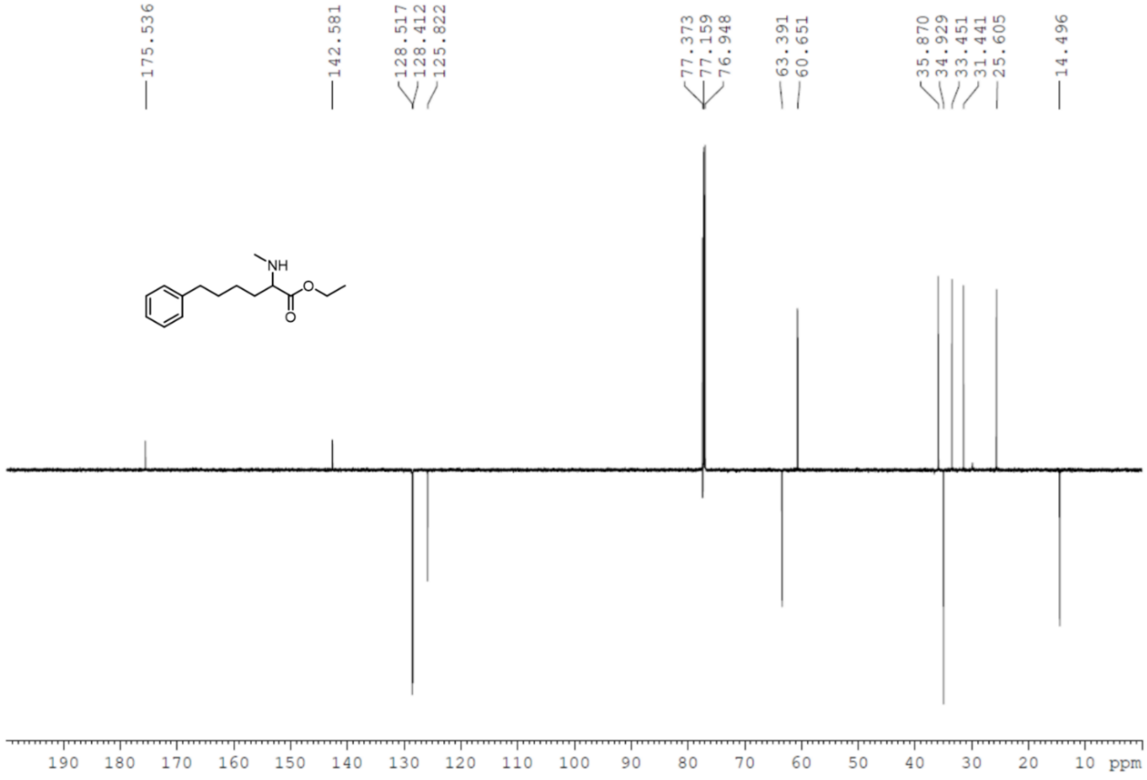

1-ethyl 8-methyl 2-(methylamino)octanedioate (7b)

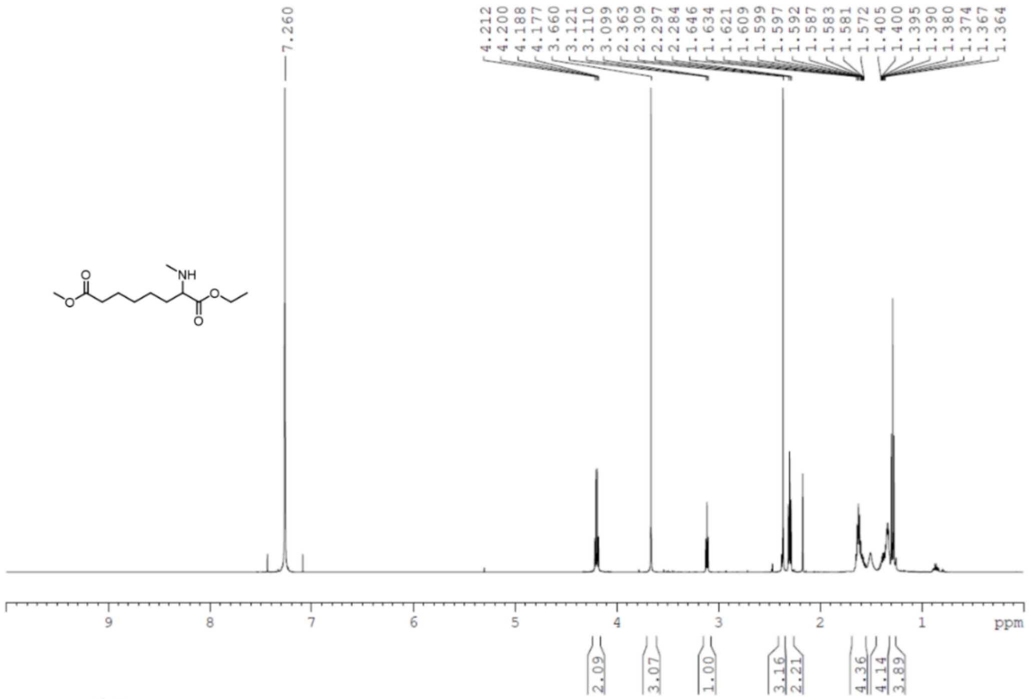

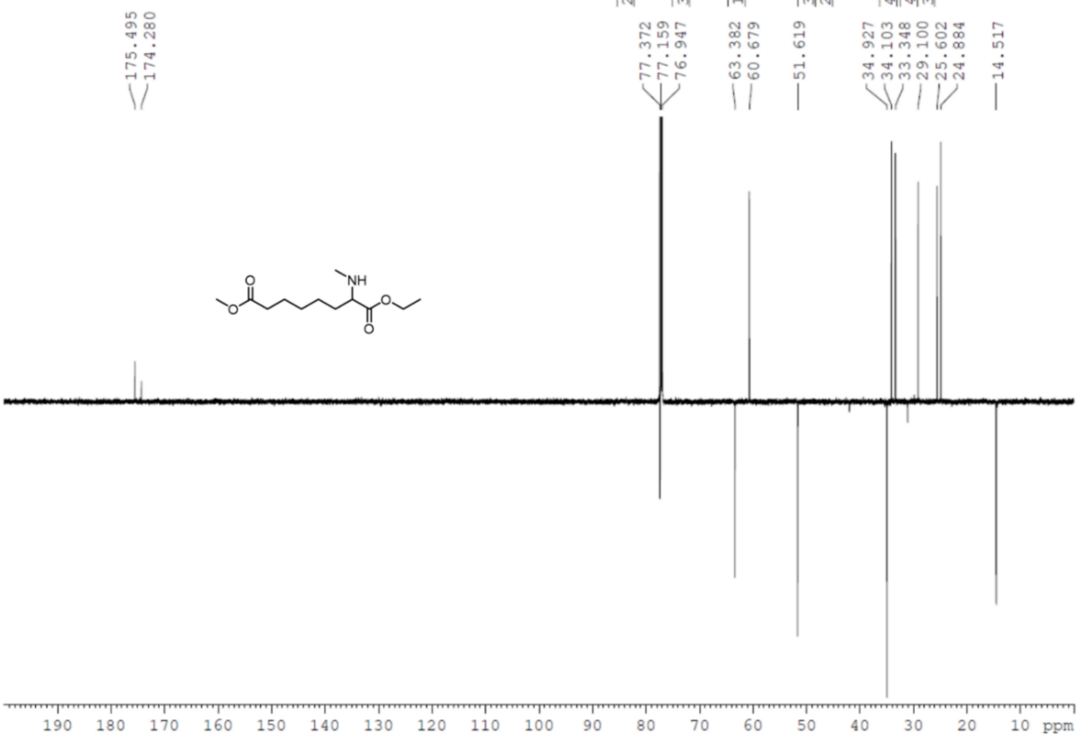

ethyl 6-acetoxy-2-(methylamino)hexanoate (8b)

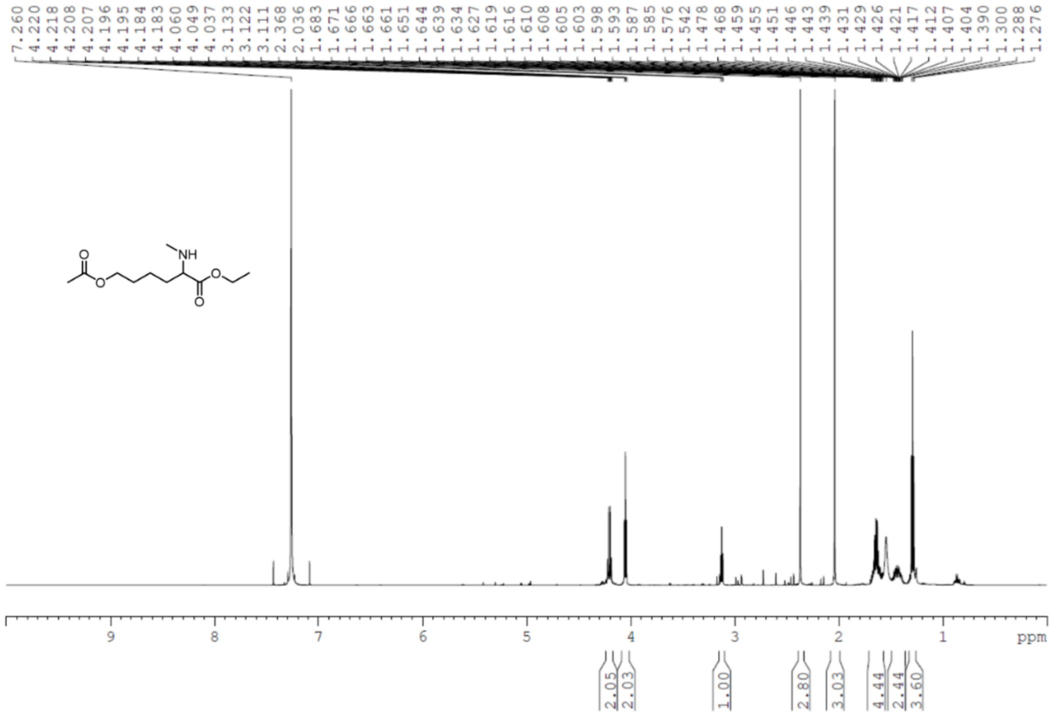

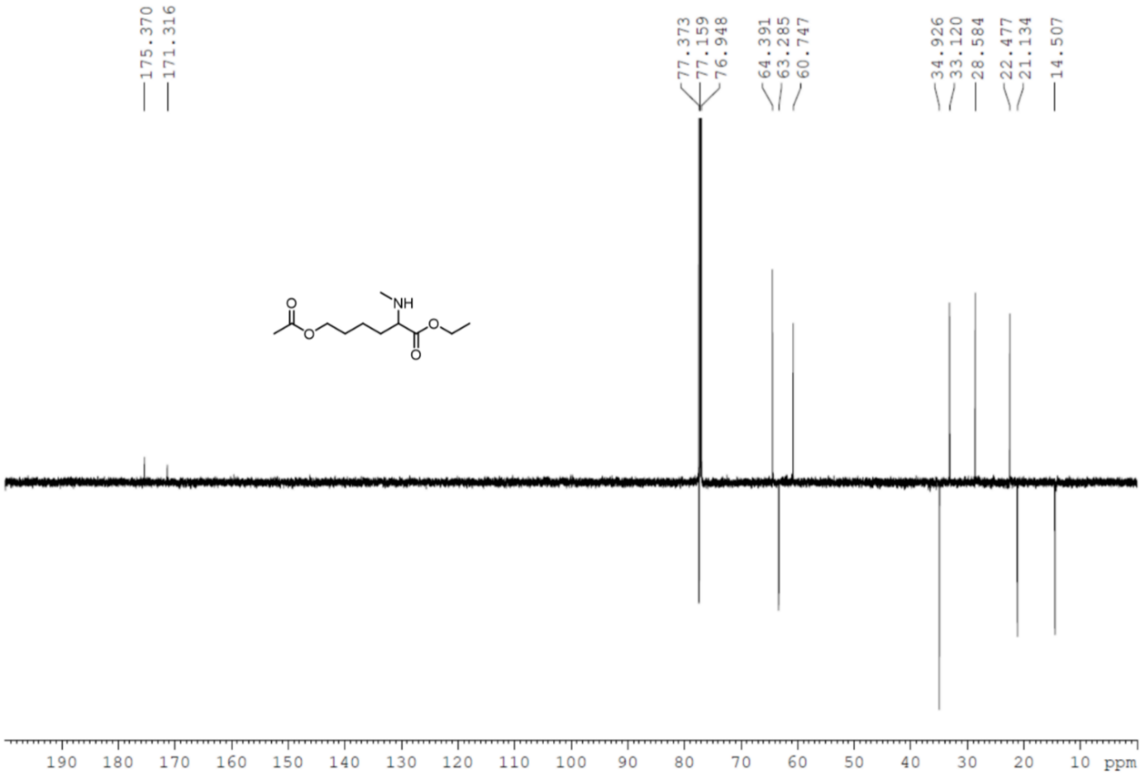

ethyl 10-bromo-2-(methylamino)decanoate (9b)

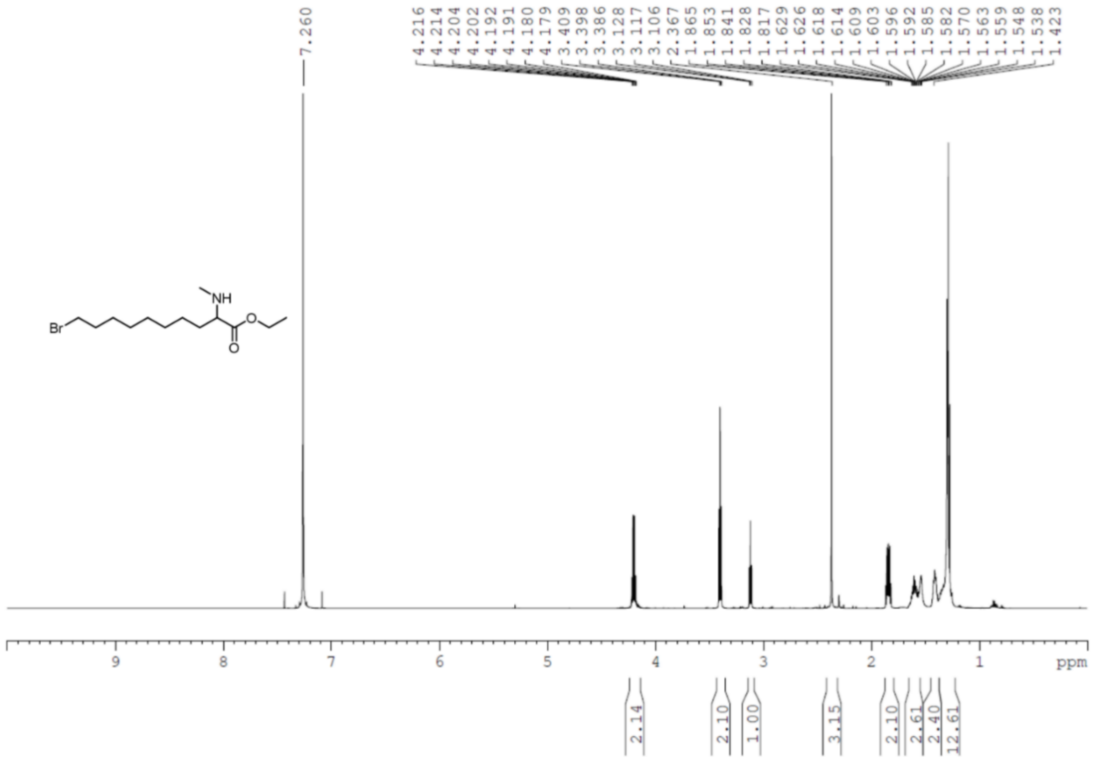

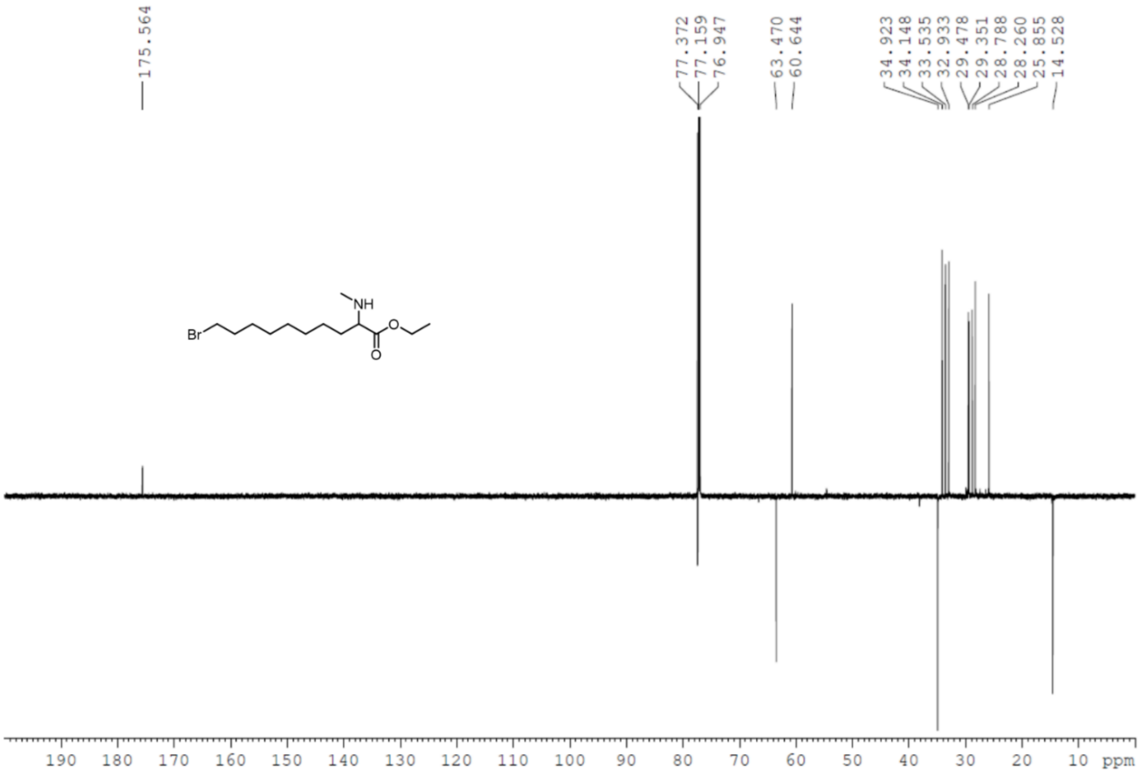

ethyl 2-(methylamino)-10-(phenylsulfonyl)decanoate (10b)

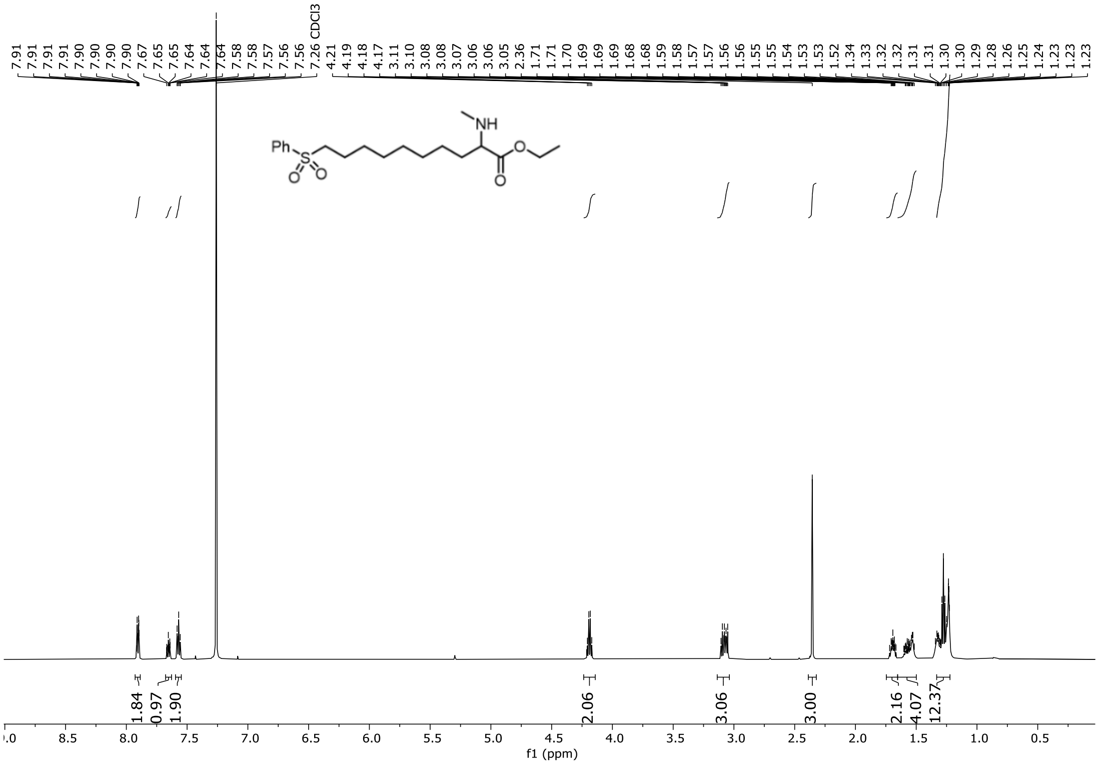

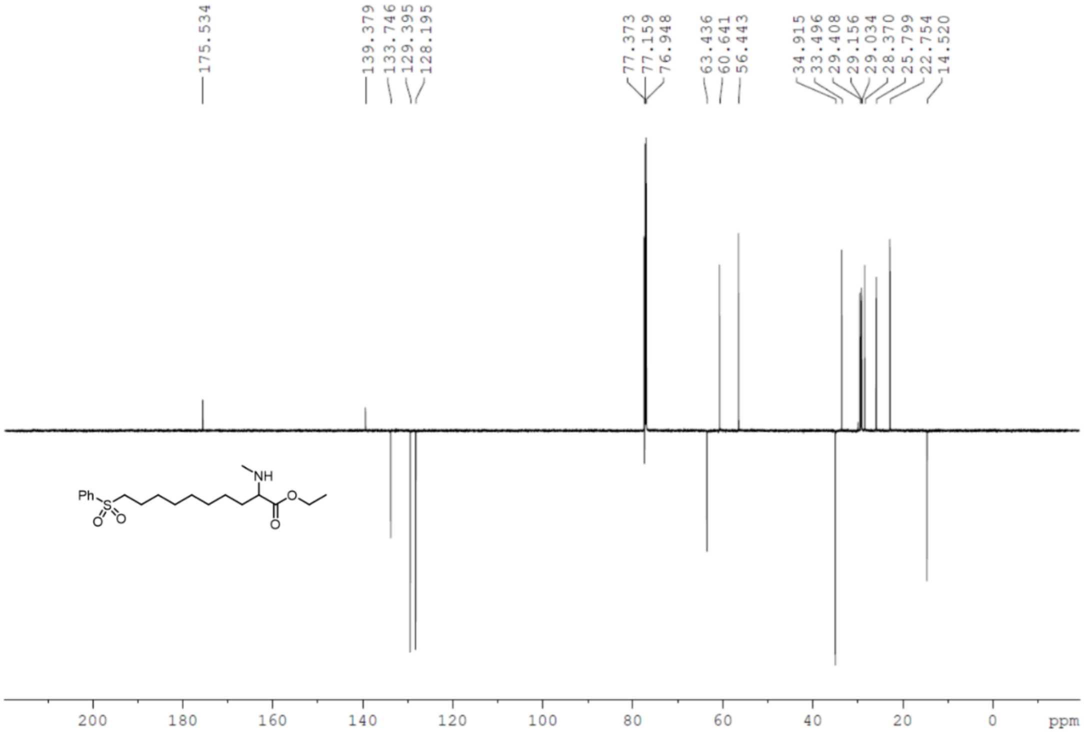

ethyl 2-(methylamino)-11-oxo-11-(pyrrolidin-1-yl)undecanoate (11b)

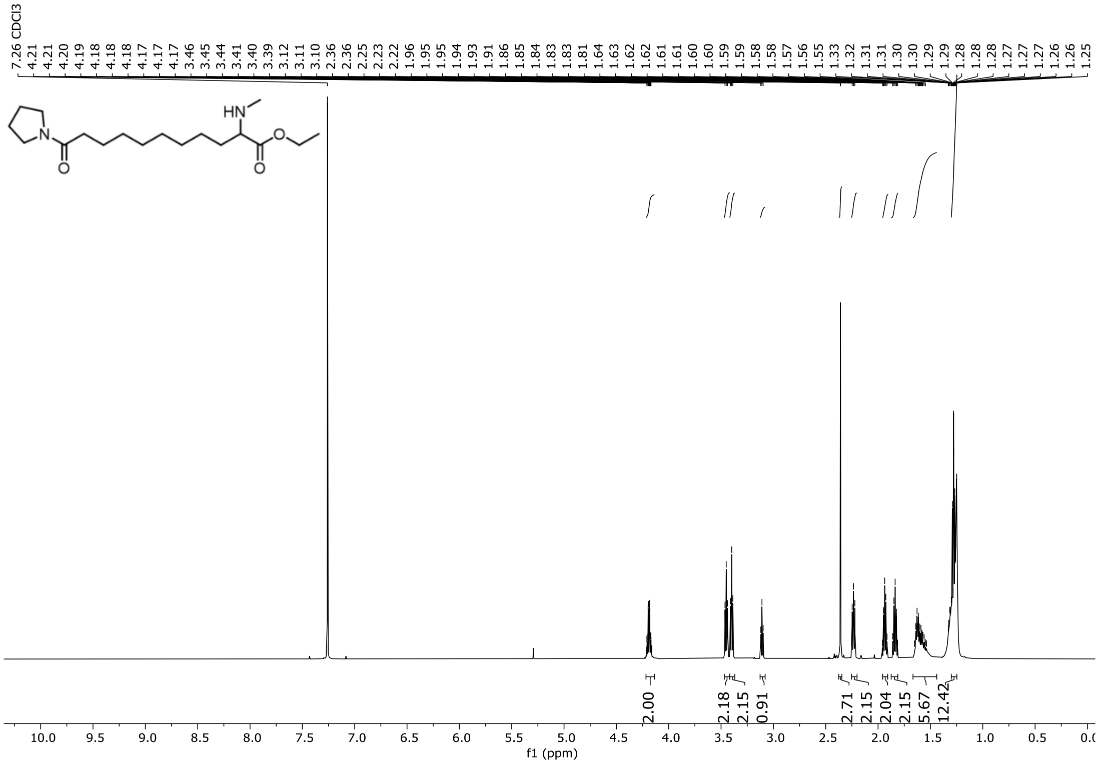

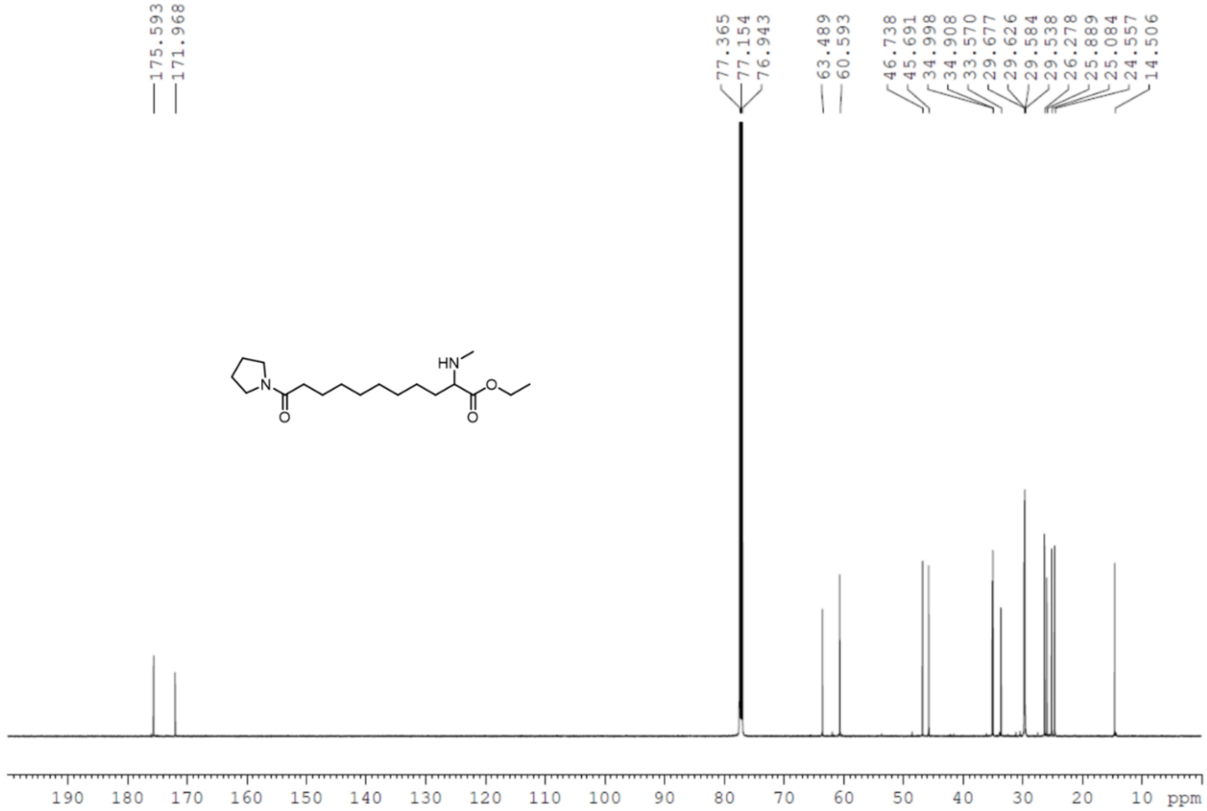

ethyl 7-hydroxy-2-(methylamino)heptanoate (12b)

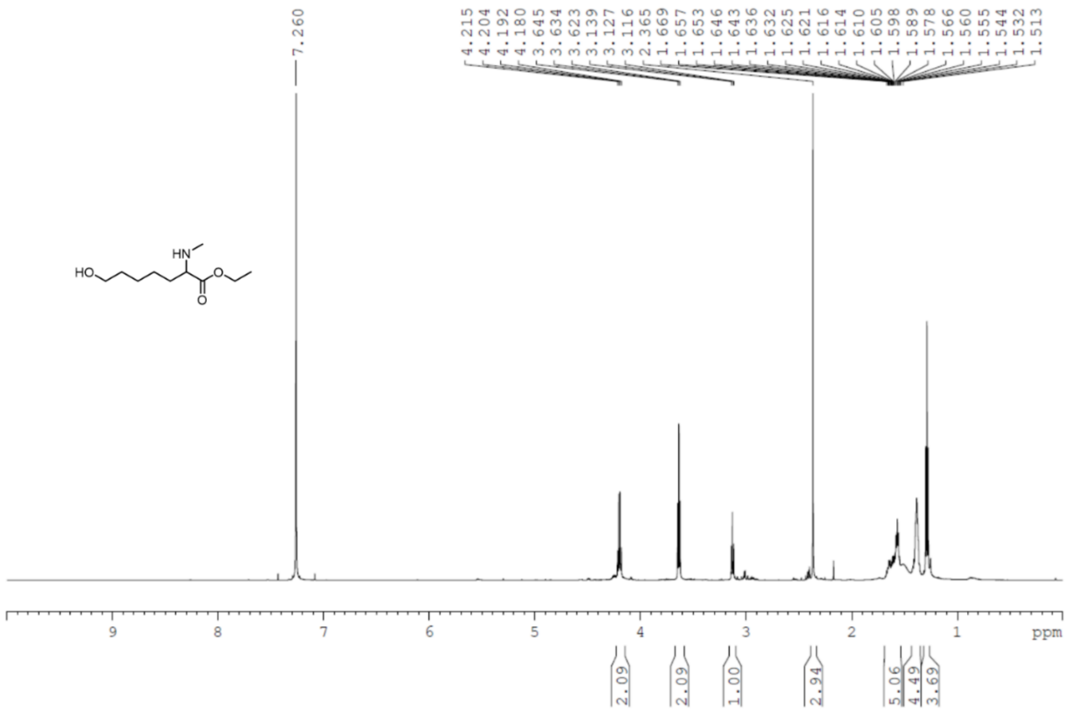

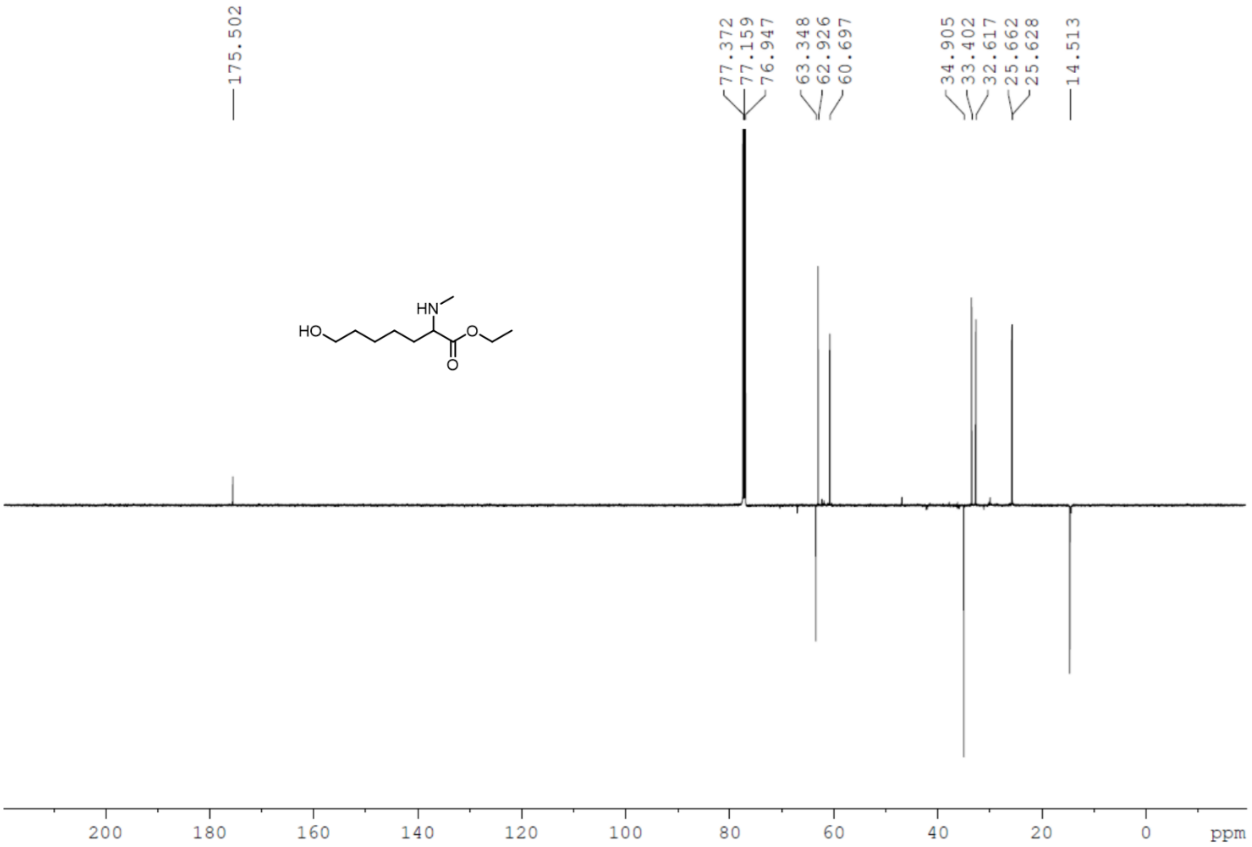

ethyl 10-(1,3-dioxoisoindolin-2-yl)-2-(methylamino)decanoate (13b)

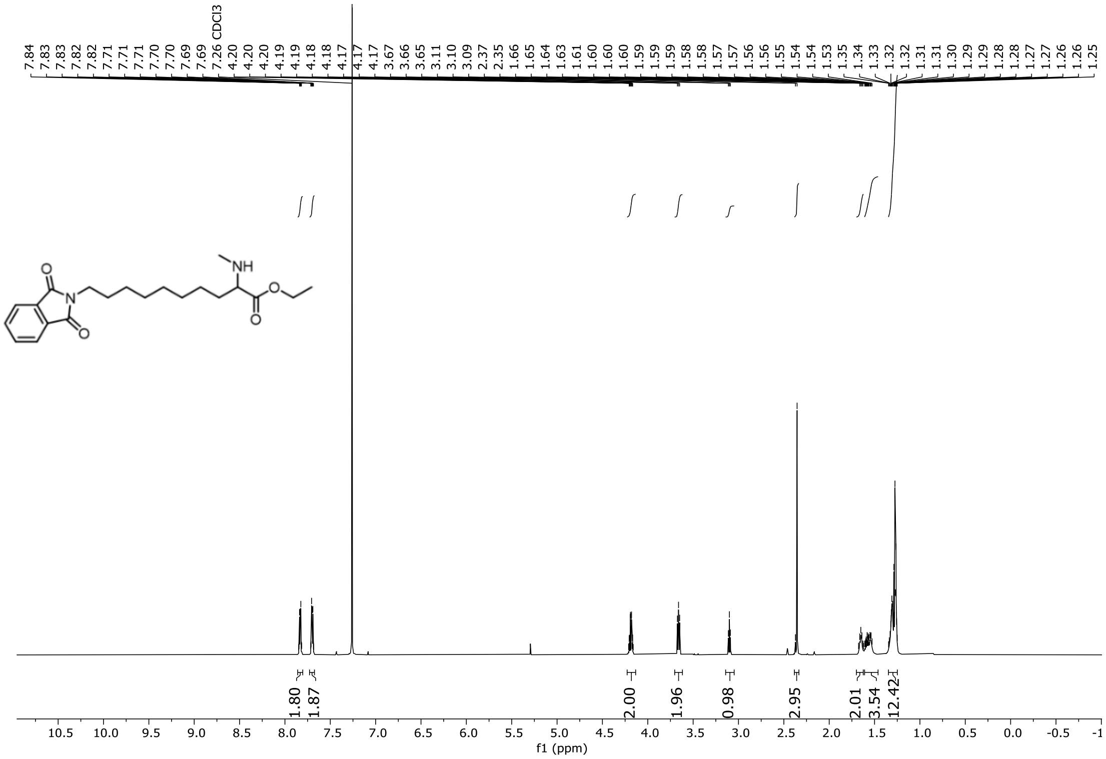

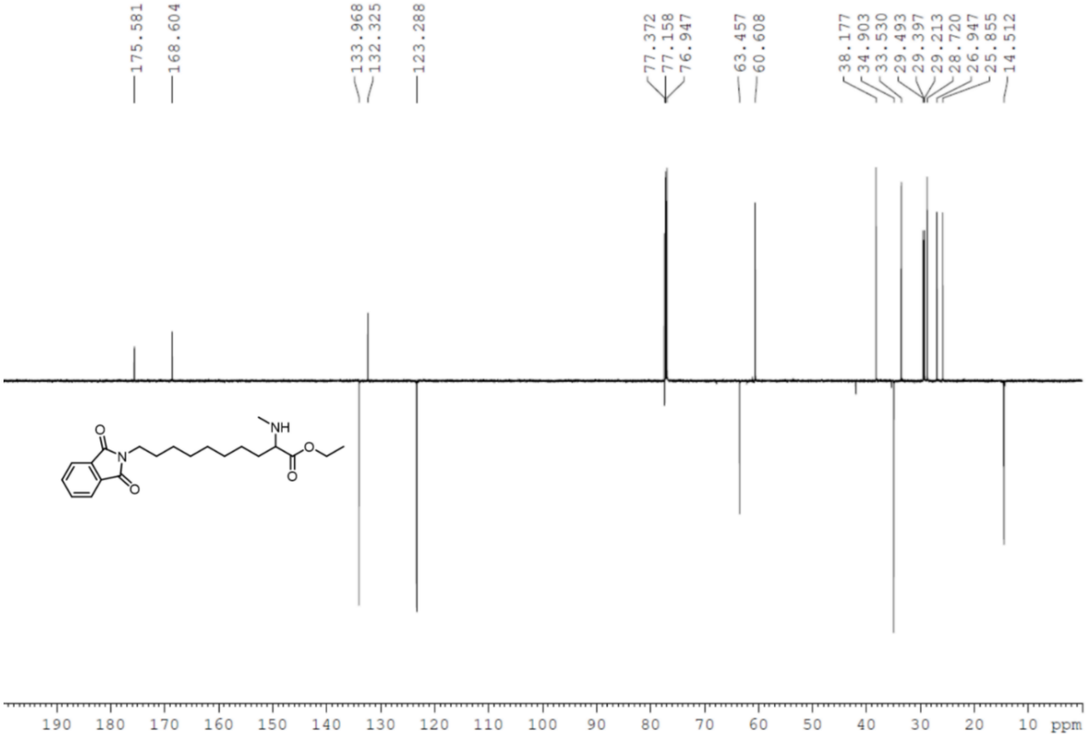

ethyl 10-azido-2-(methylamino)decanoate (14b)

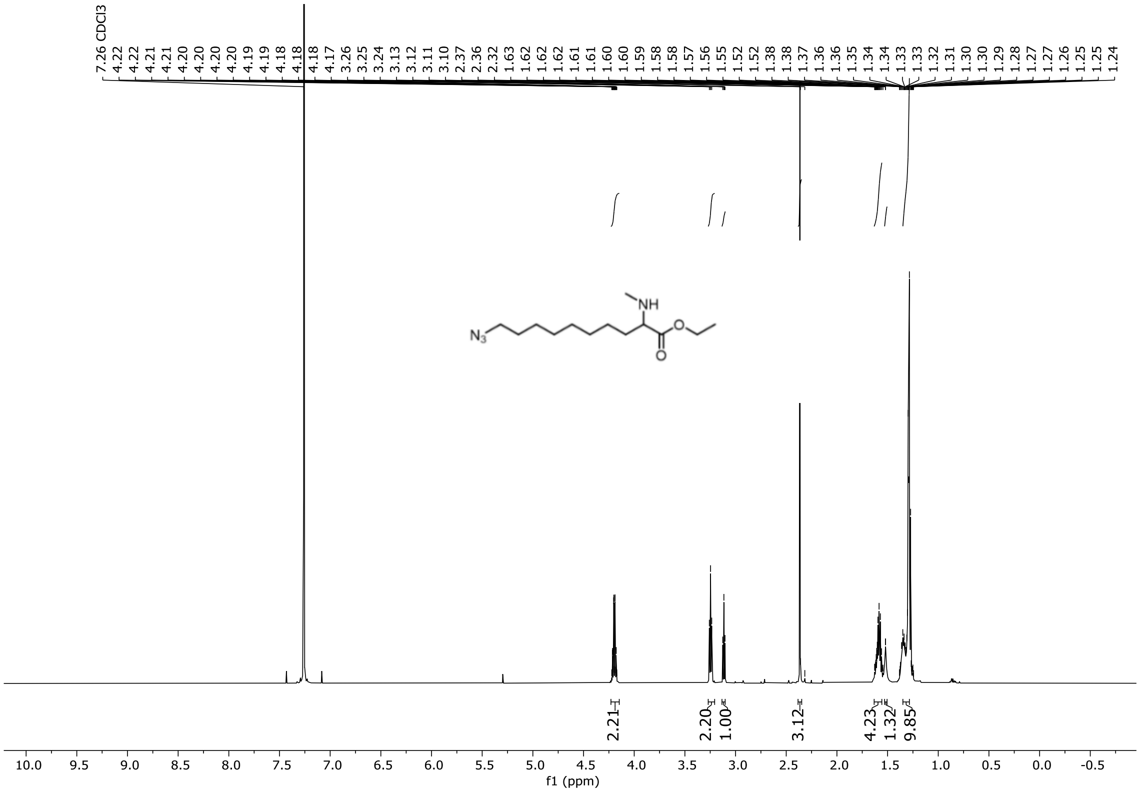

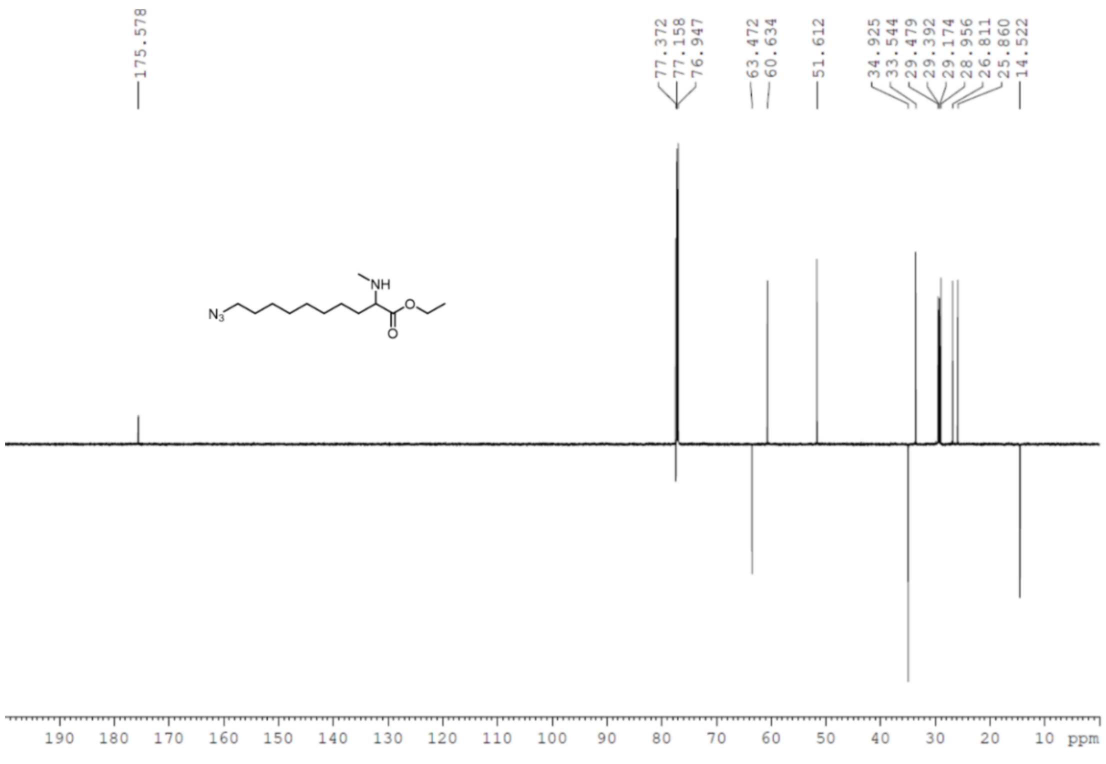

ethyl 2-cycloheptyl-2-(methylamino)acetate (15b)

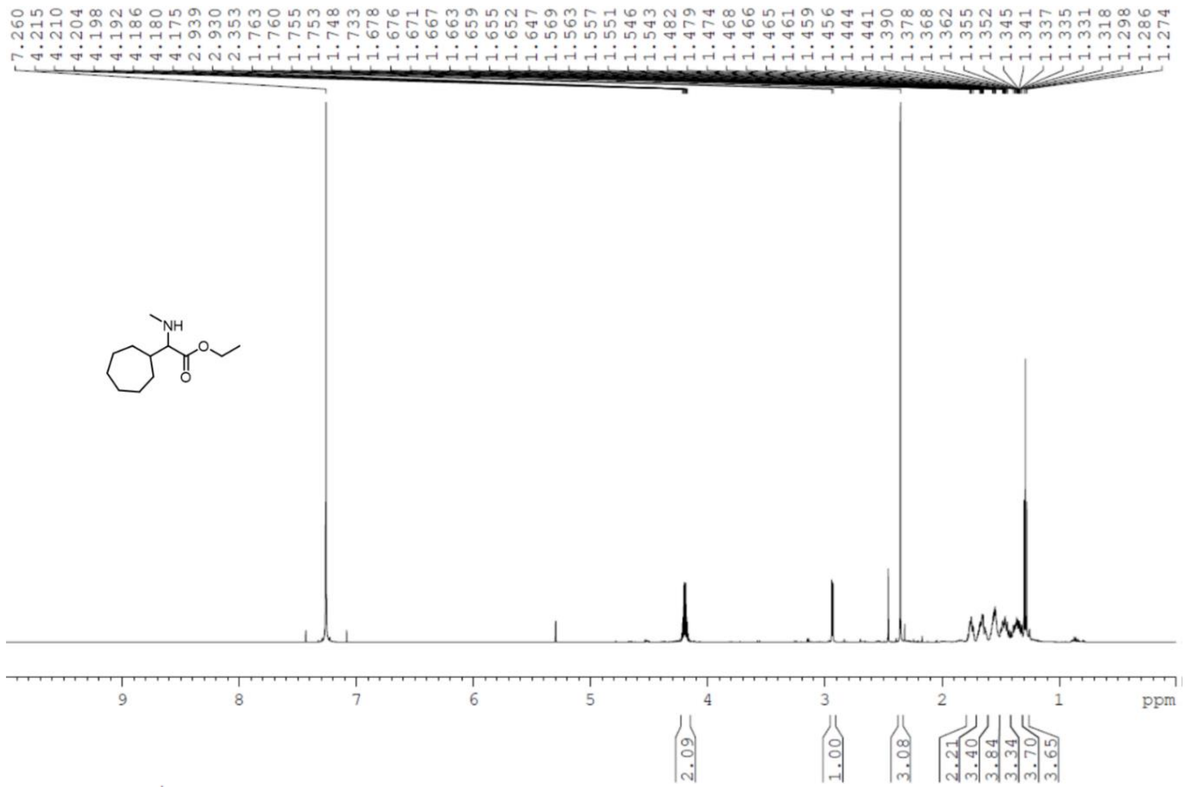

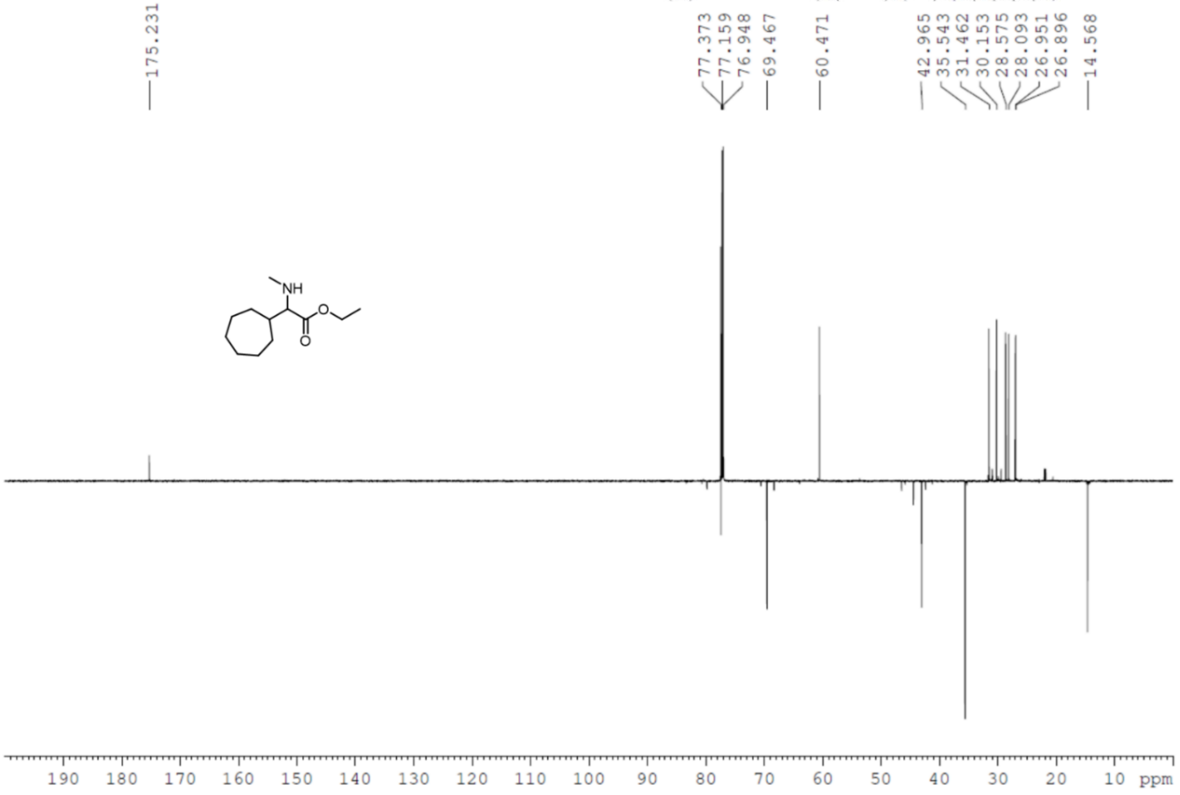

ethyl 2-(benzylamino)-4-phenylbutanoate (16b)

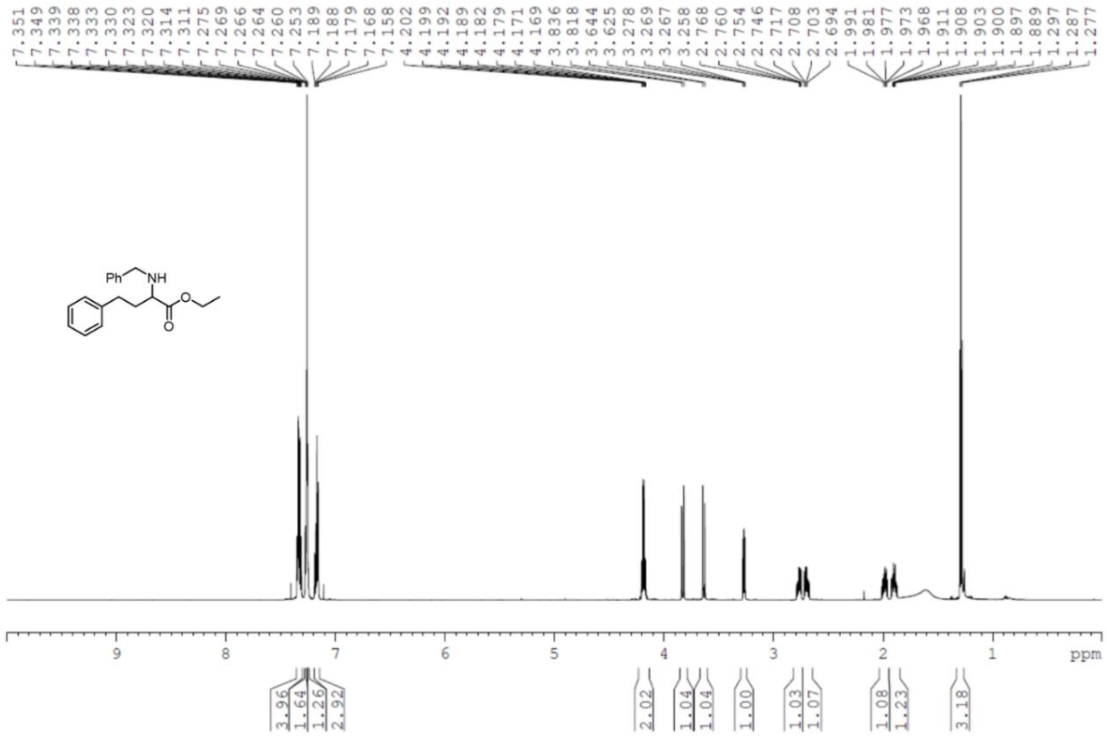

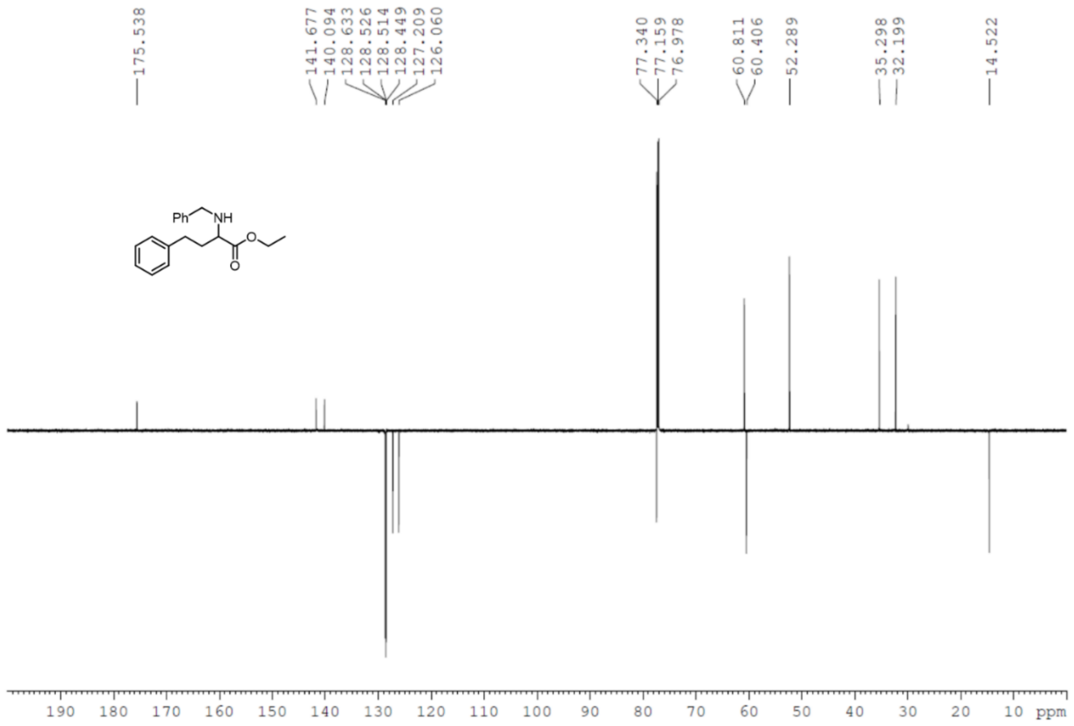

Homophenylalanine (17b)

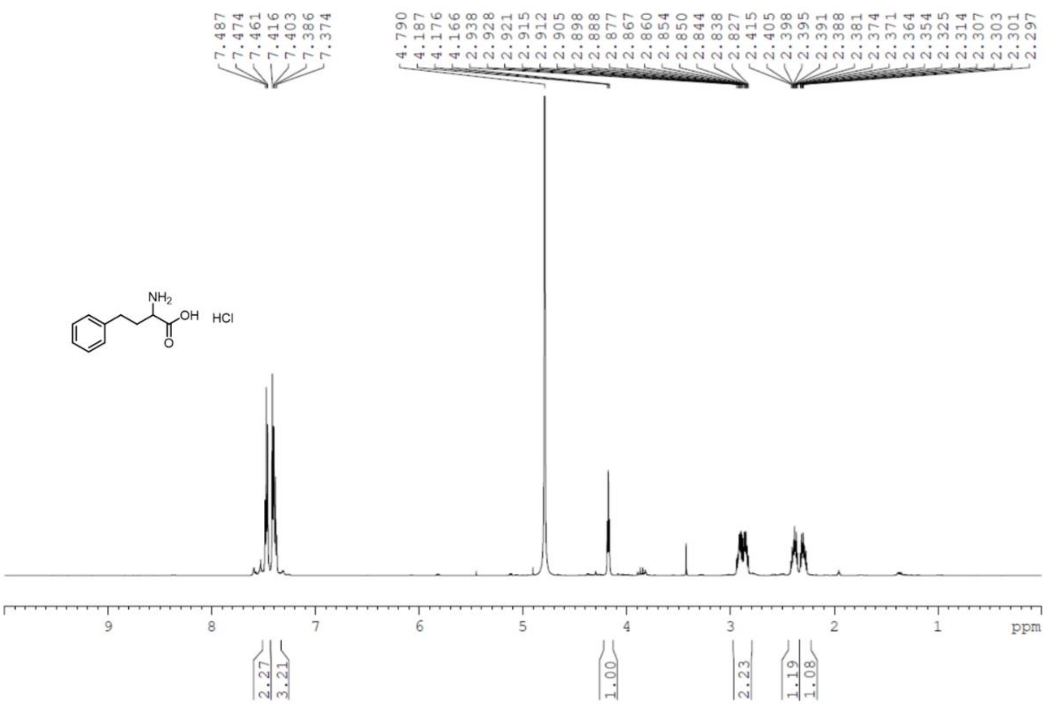

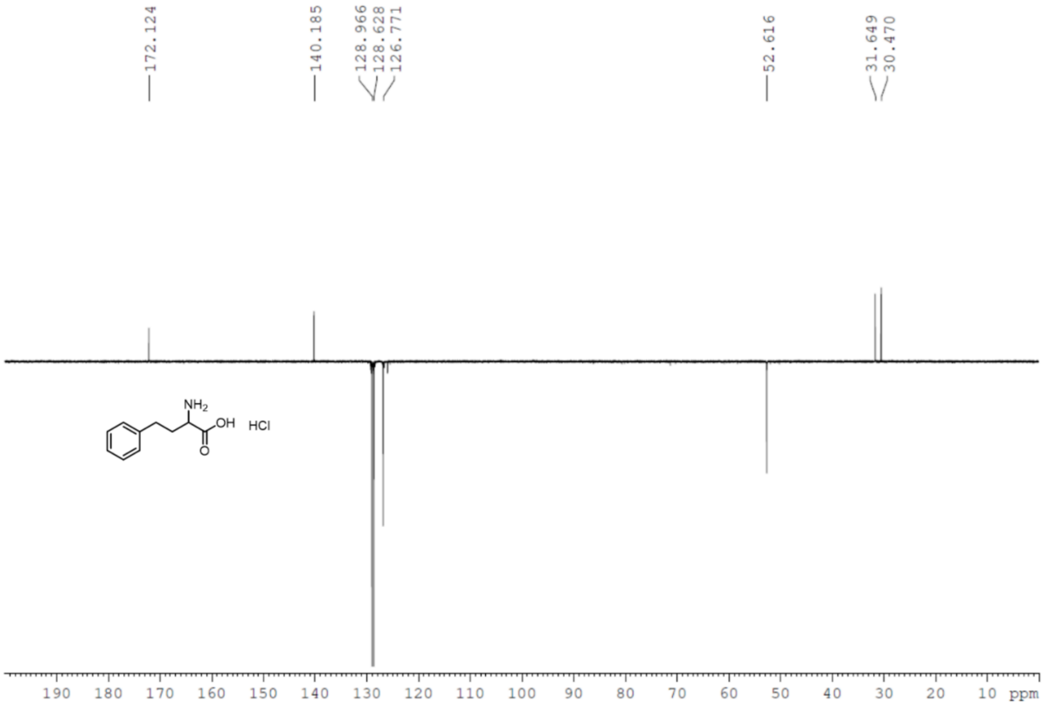

Diethyl (1-(methylamino)-3-phenylpropyl)phosphonate (1c)

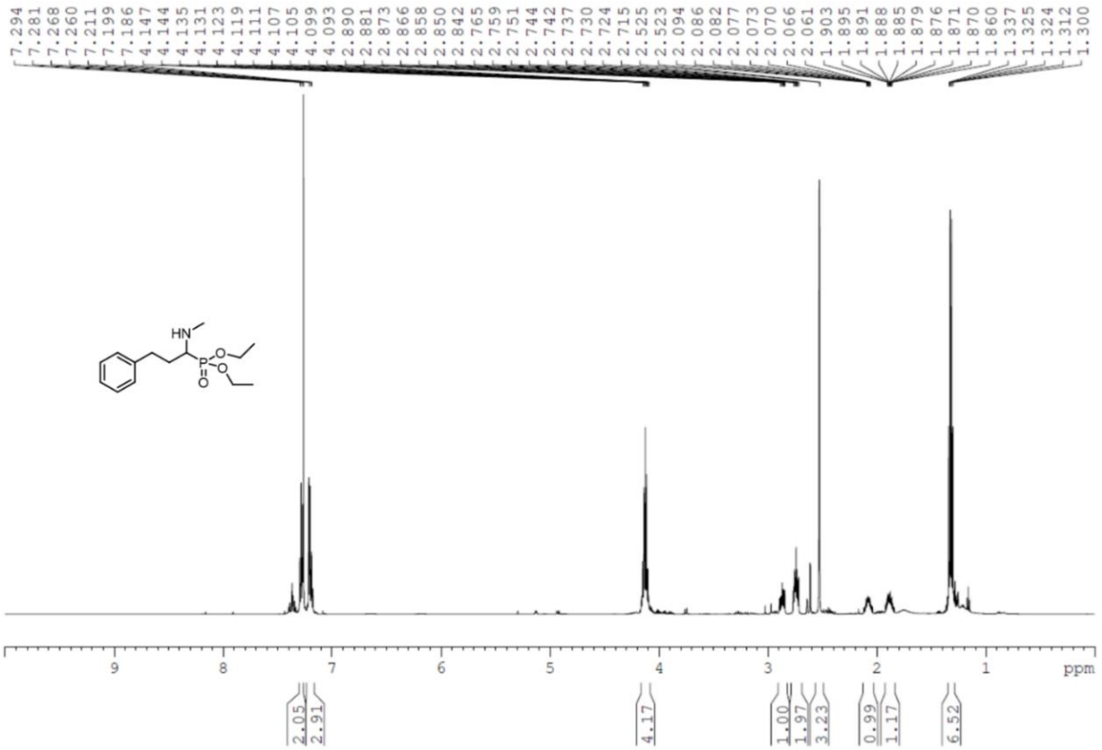

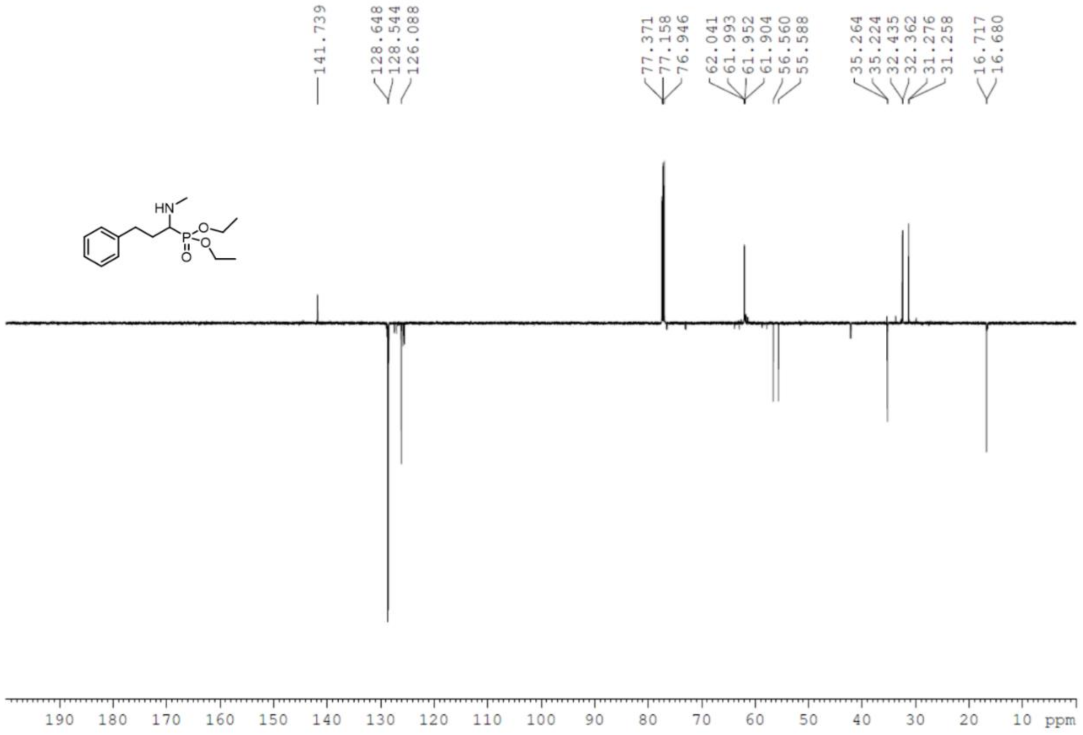

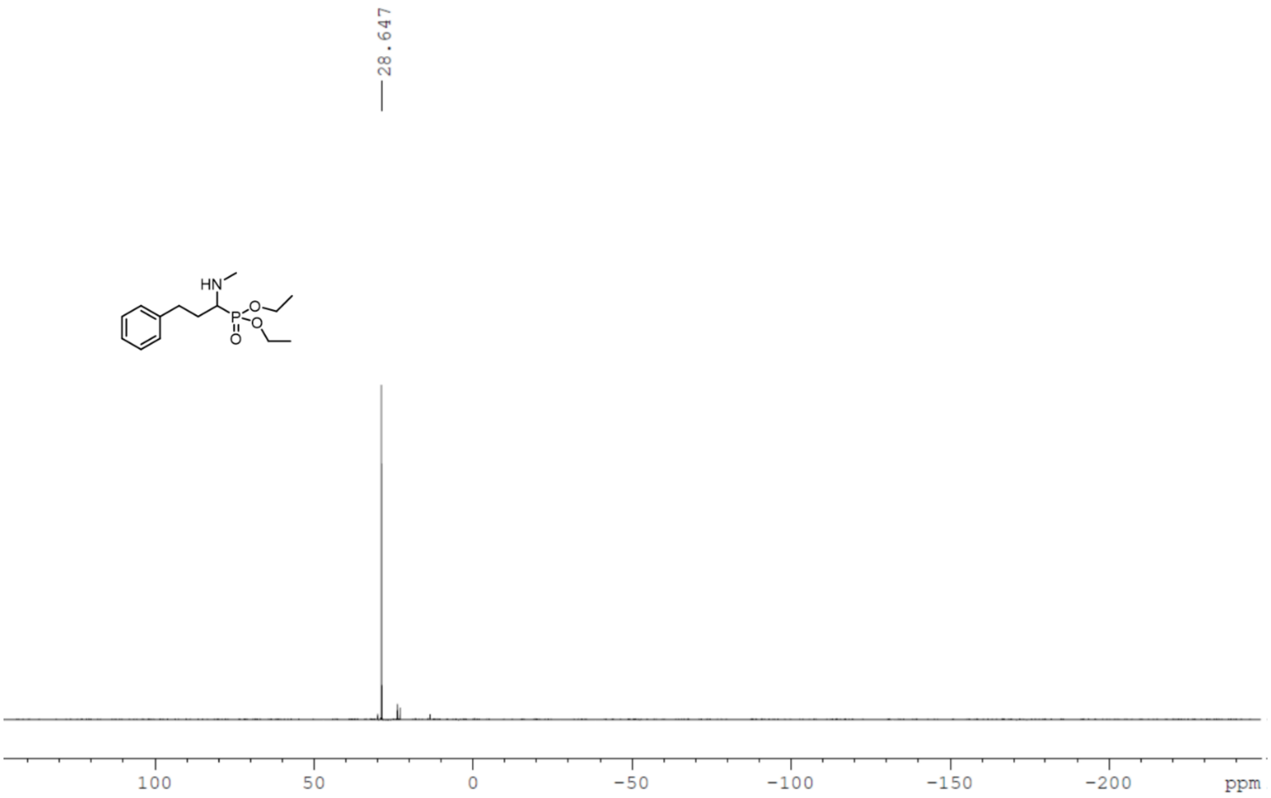

28.647
100
50
0
−50
−100
−150
−200
ppm

Diethyl (1-(methylamino)-3-phenylpropyl)phosphonate (2c)

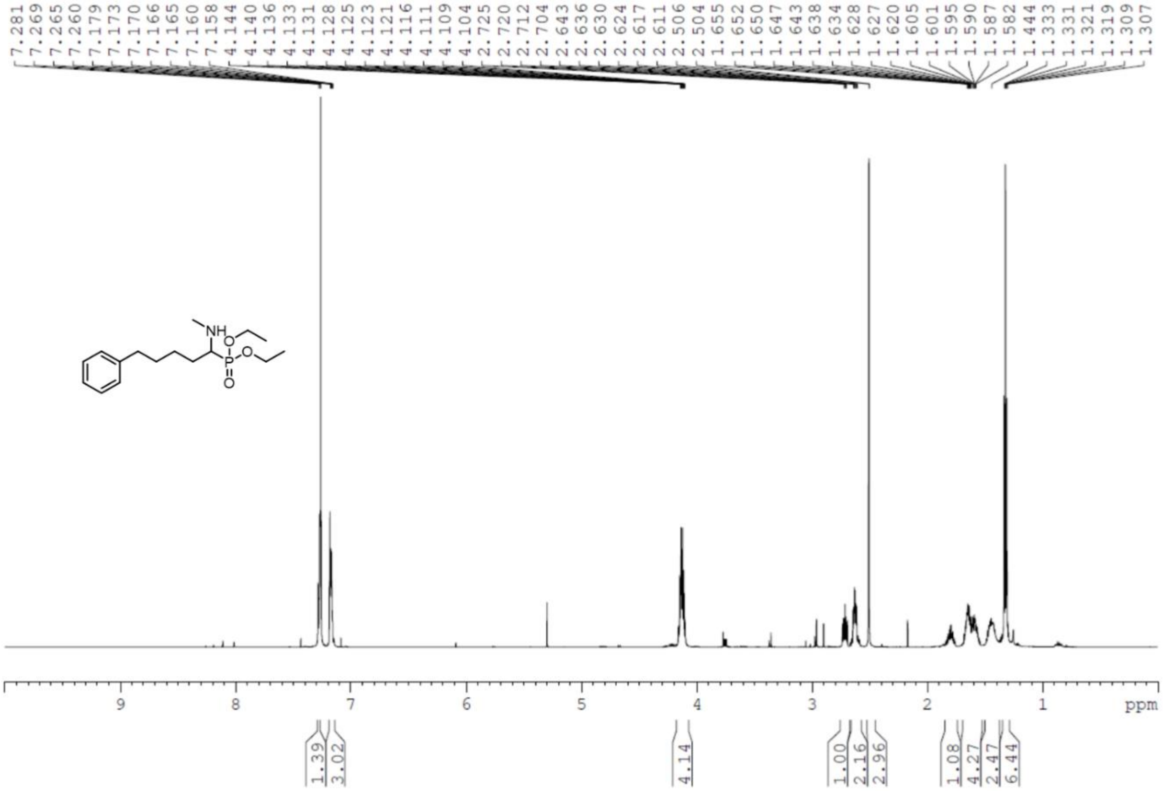

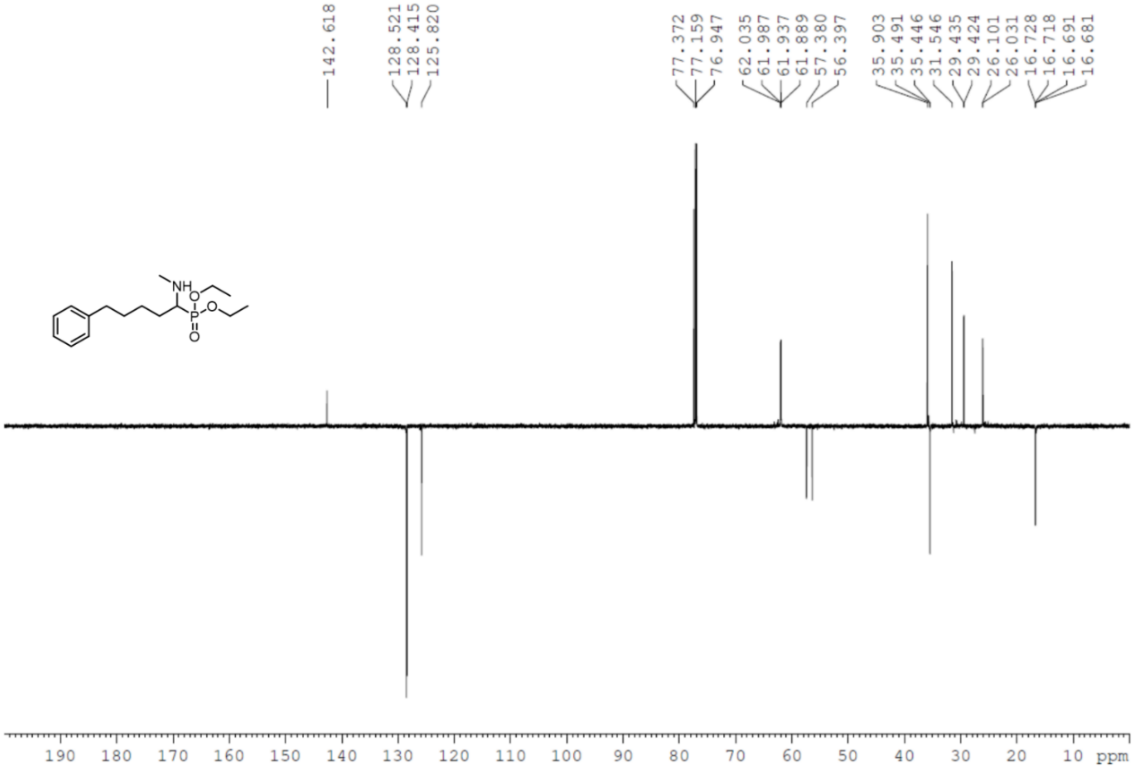

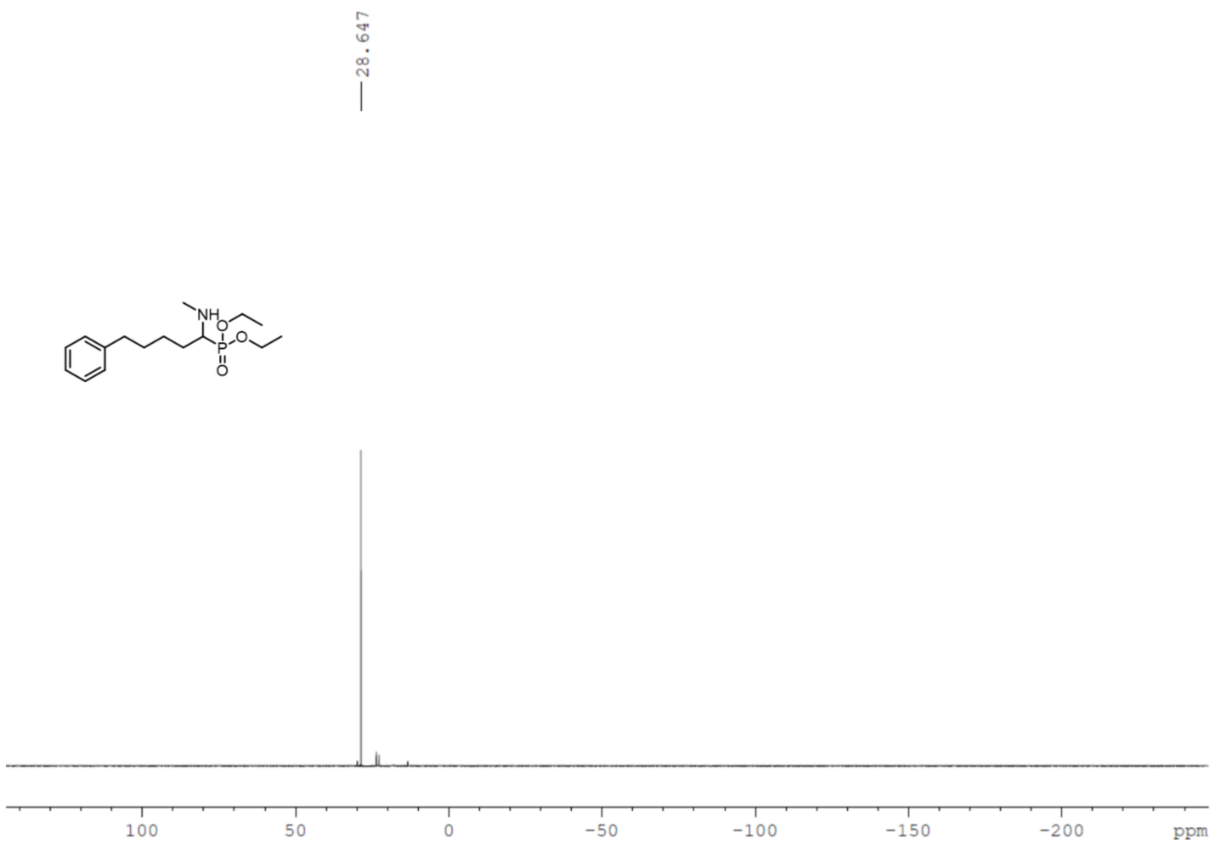
28.647
NH
O
P
O
O
100
50
0
−50
−100
−150
−200
ppm

diethyl (1-(methylamino)decyl)phosphonate (3c)

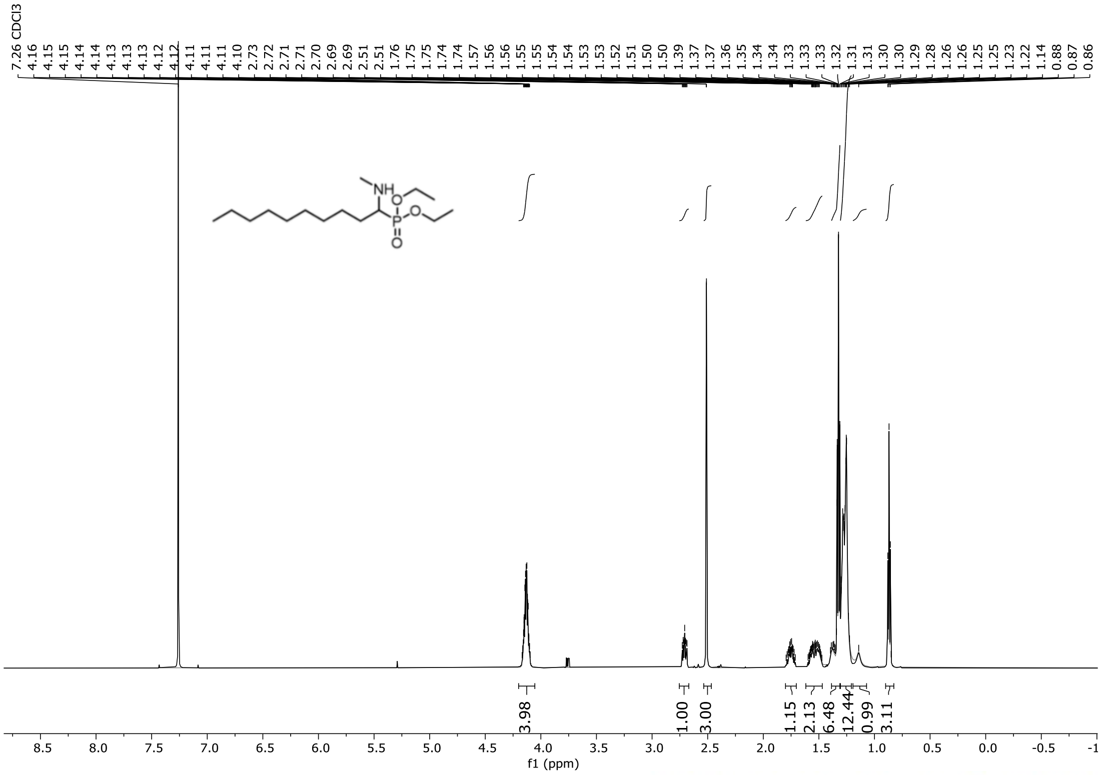

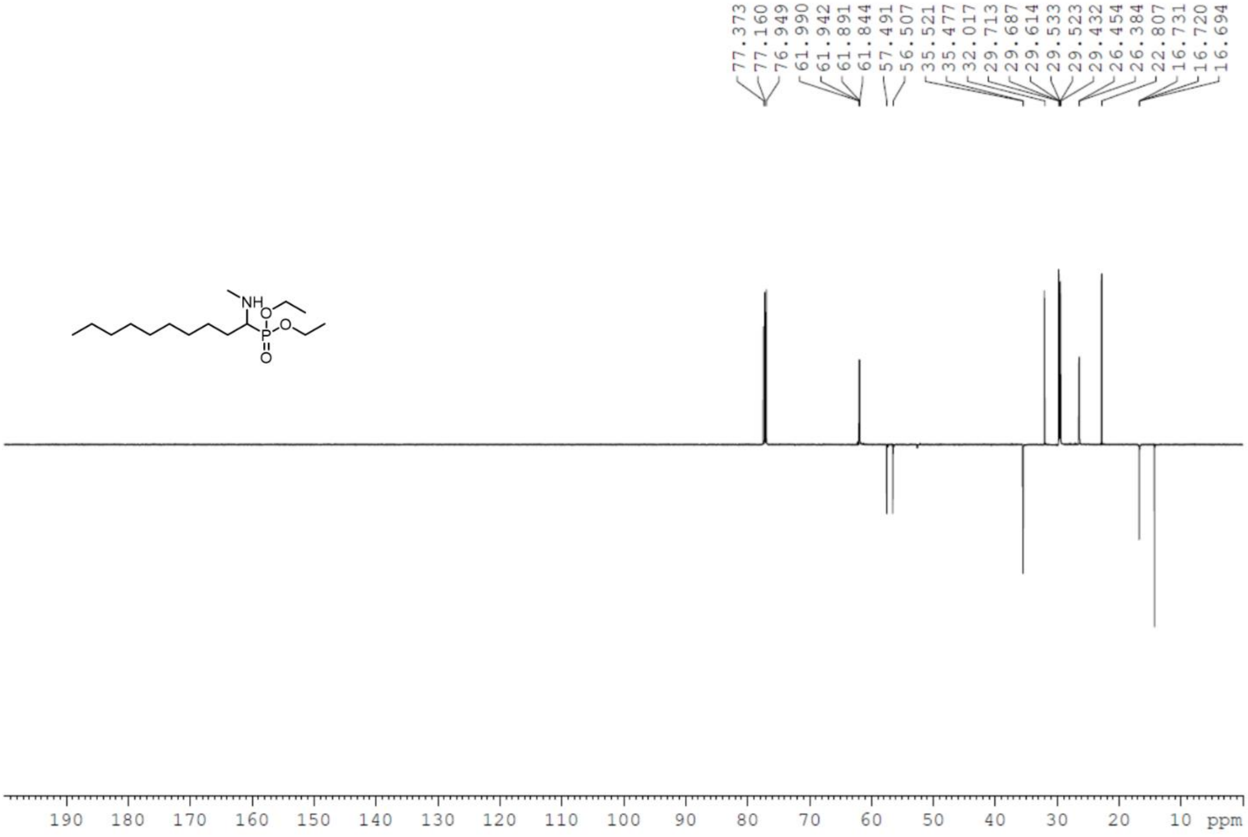

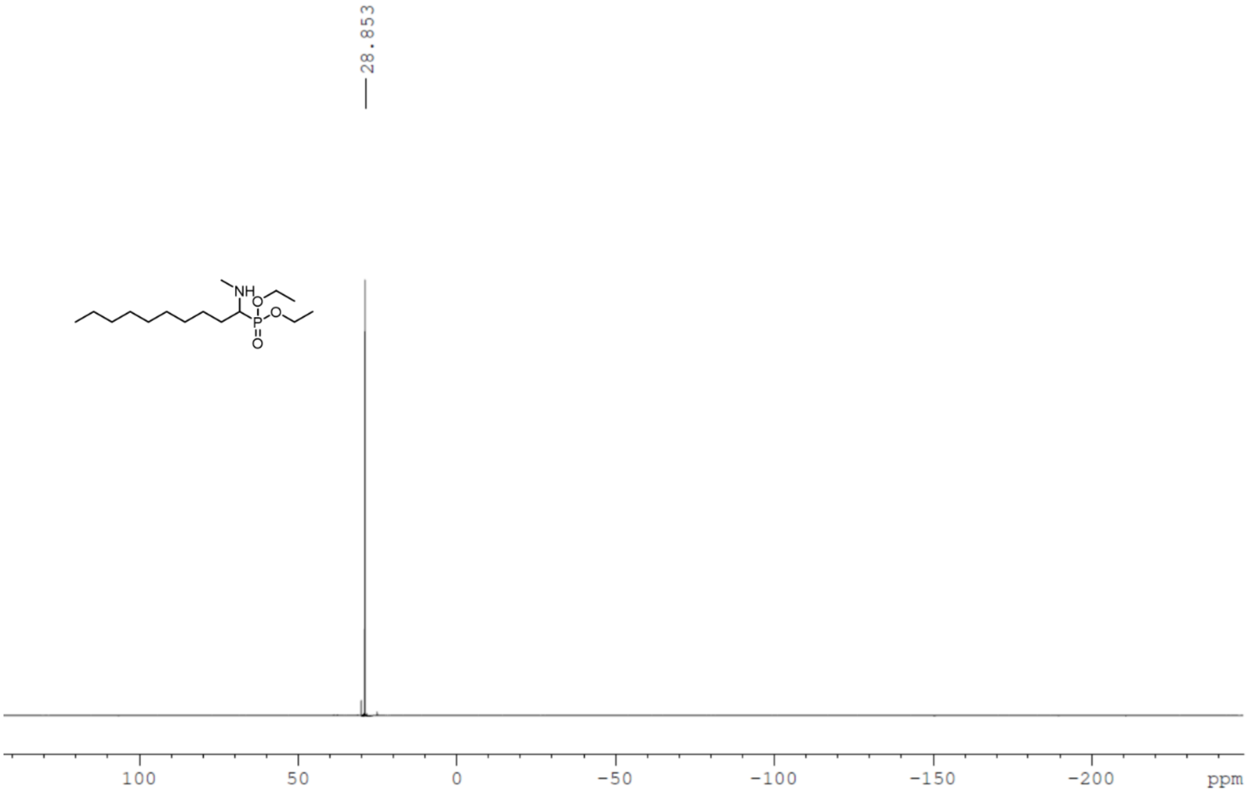

28.853
NH
O
P
O
O
100
50
0
−50
−100
−150
−200
ppm

diethyl (9-bromo-1-(methylamino)nonyl)phosphonate (4c)

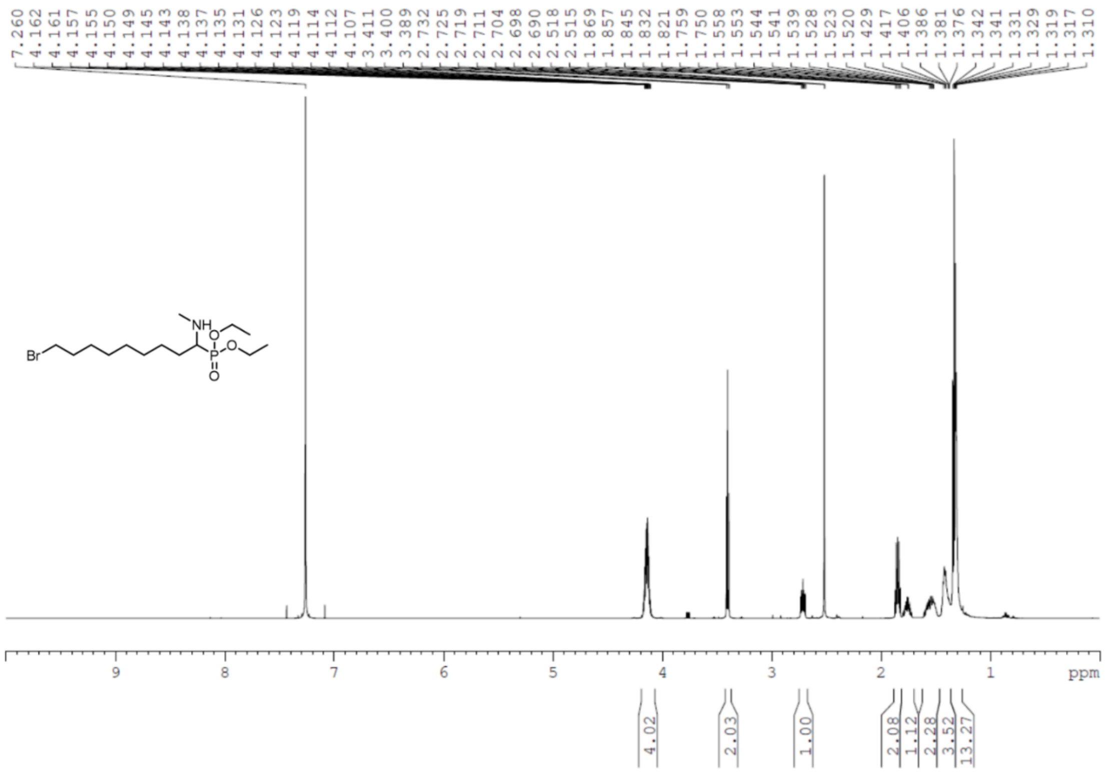

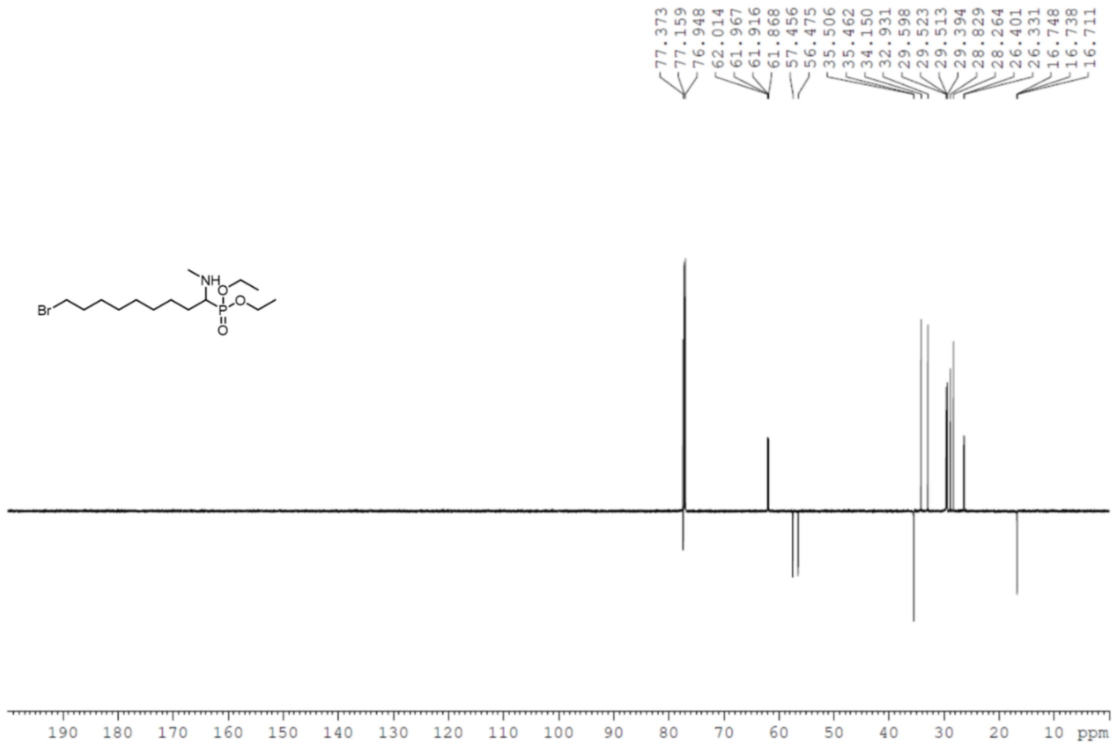

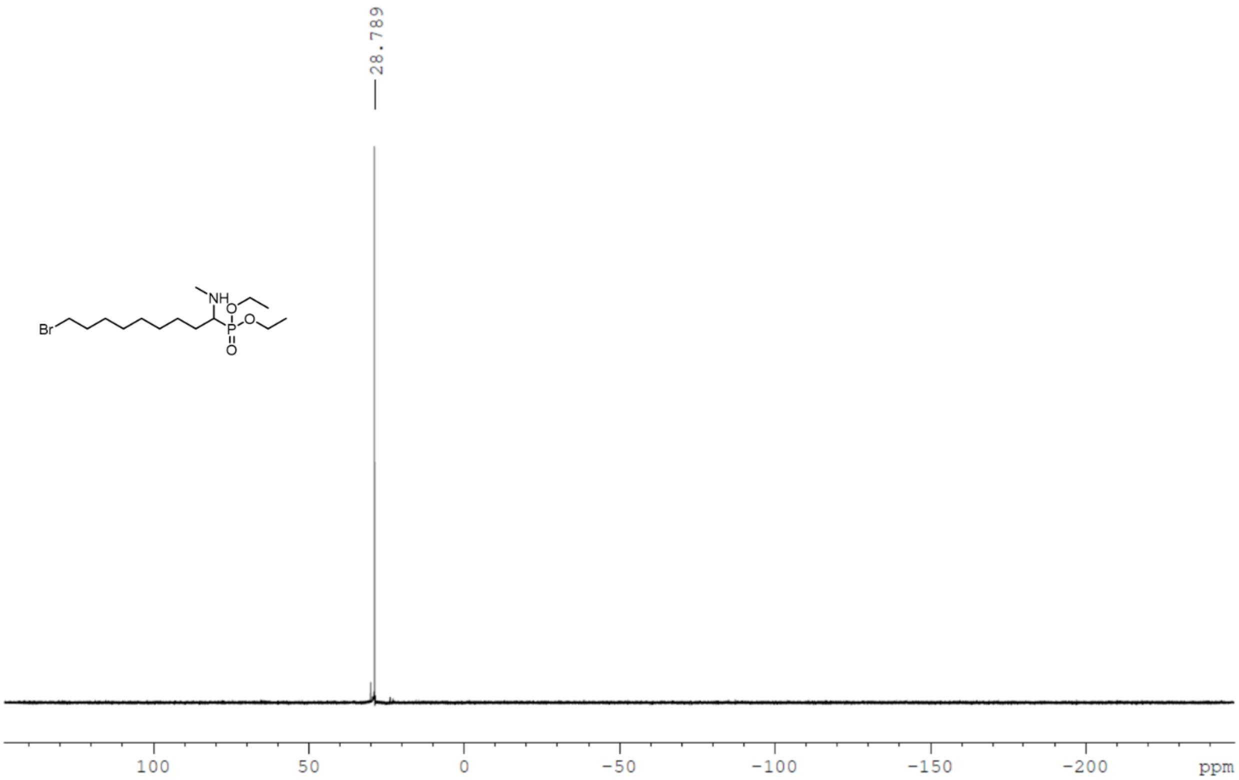

28.789
Br
NH
O
P
O
O
100
50
0
−50
−100
−150
−200
ppm

(*Z*)-4-Chloro-6-(dimethylamino)-7,7,7-trifluorohept-4-en-1-ol (4d)

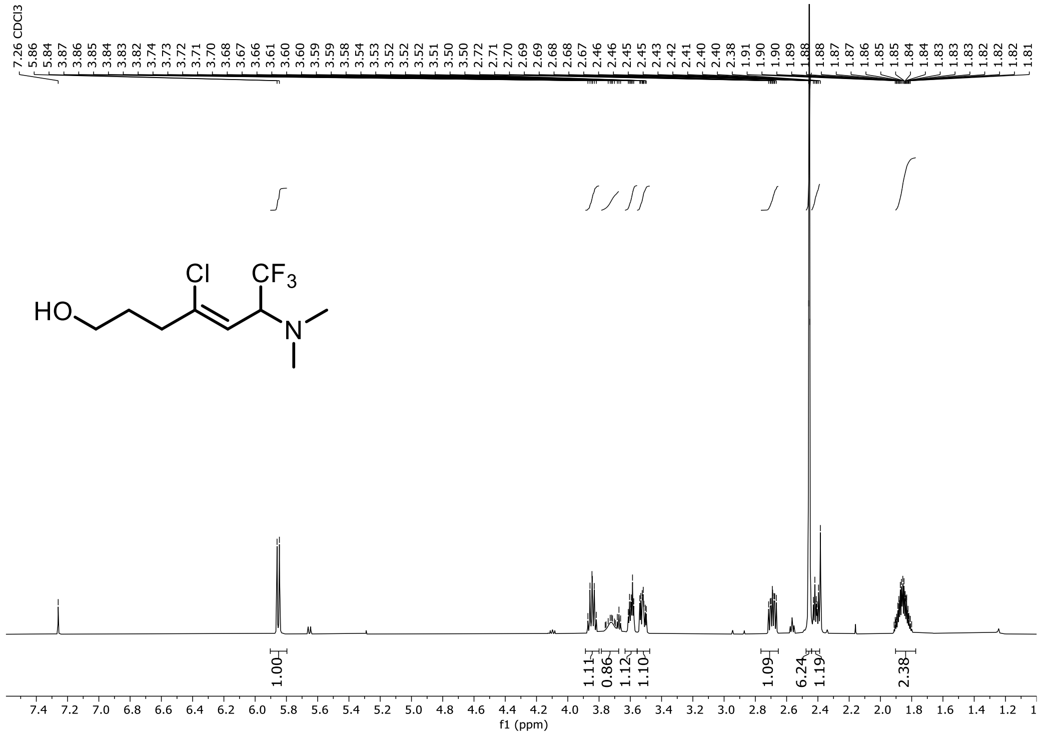

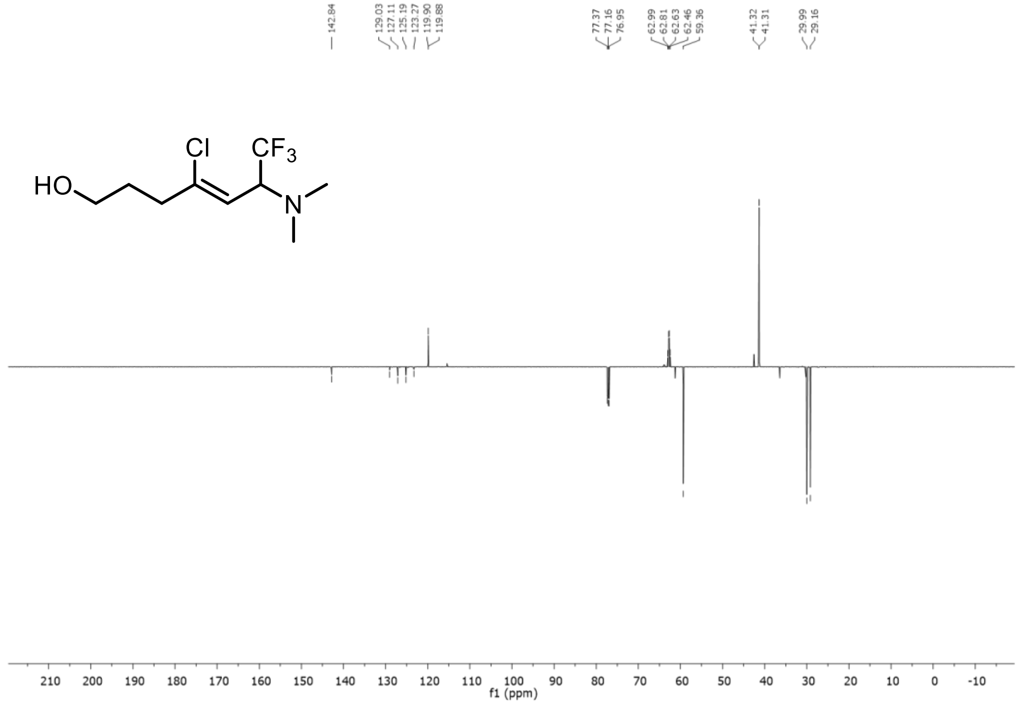

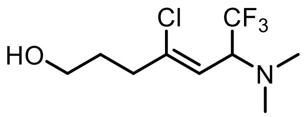

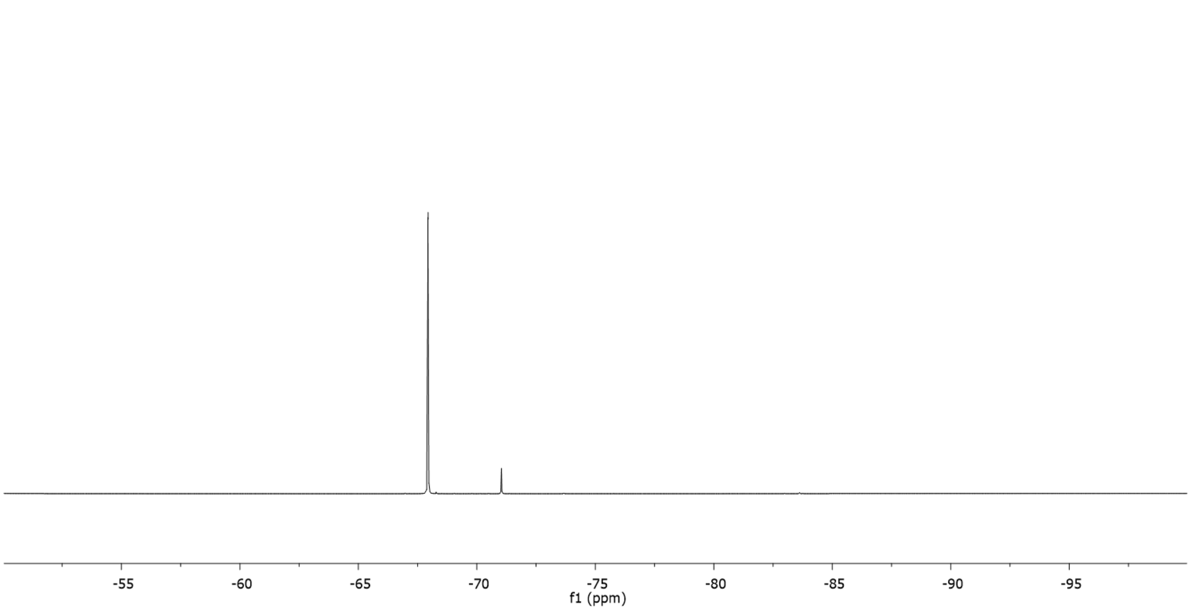

-67.93
-67.94
-55
-60
-65
-70
-75
-80
-85
-90
-95
f1 (ppm)

(*Z*)-3-(3,4-Dihydronaphthalen-1(2H)-ylidene)-1,1,1-trifluoro-*N*,*N*-dimethylpropan-2-amine (7d)

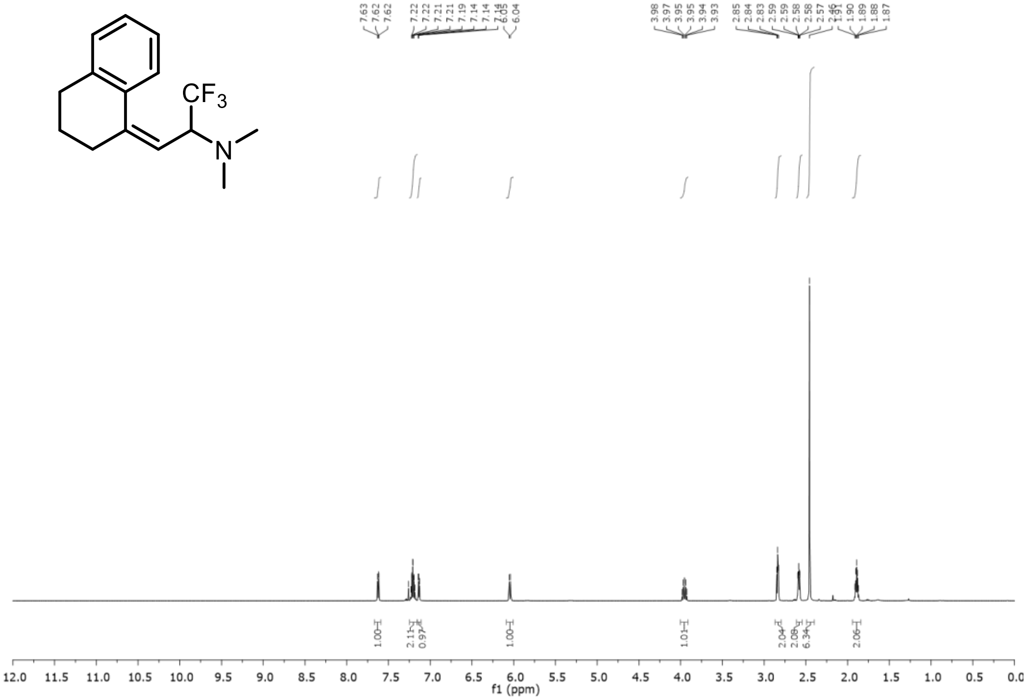

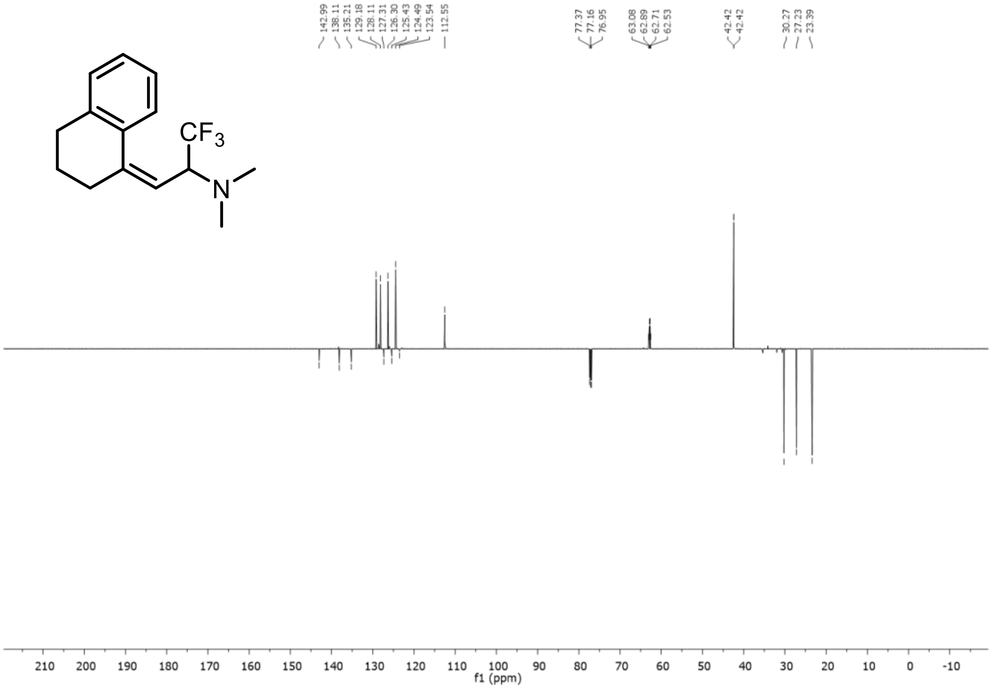

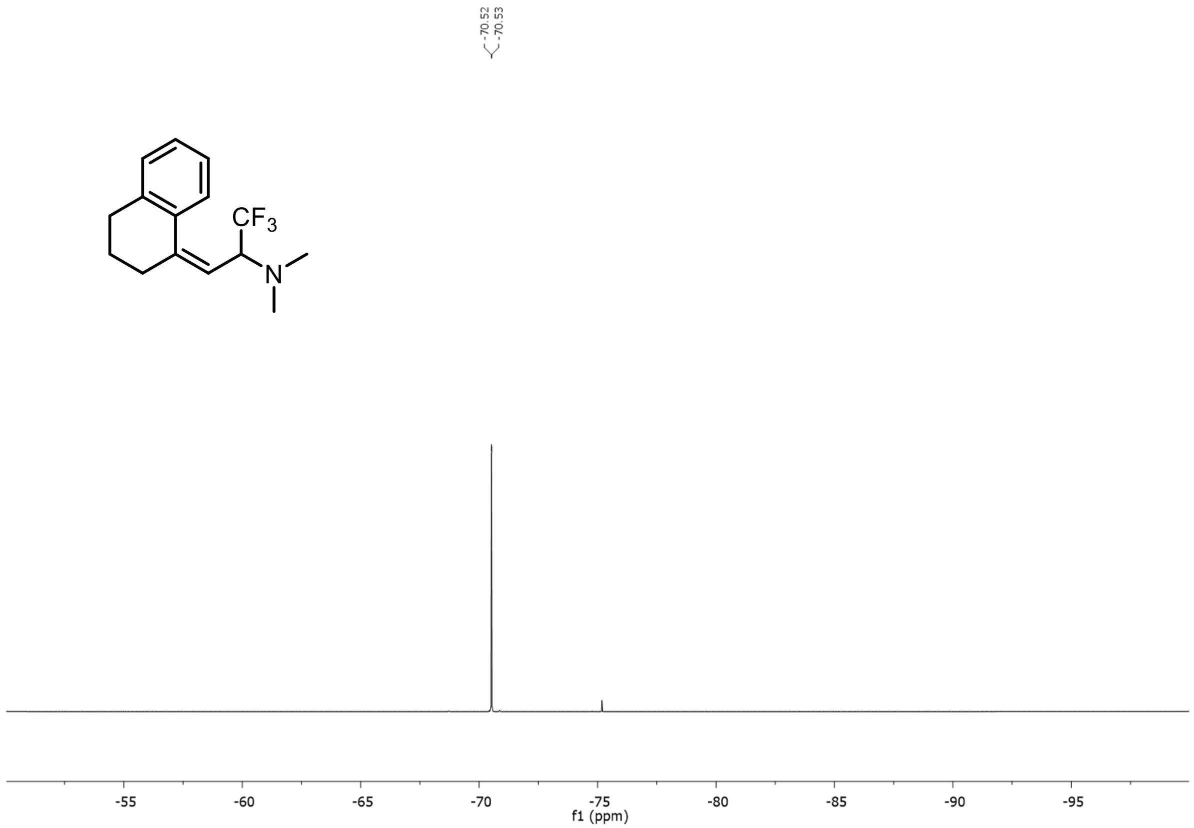
CF3
N
-70.52
-70.53
-55
-60
-65
-70
-75
-80
-85
-90
-95
f1 (ppm)

Ethyl 6-(dimethylamino)-7,7,7-trifluoro-4-oxoheptanoate (11d)

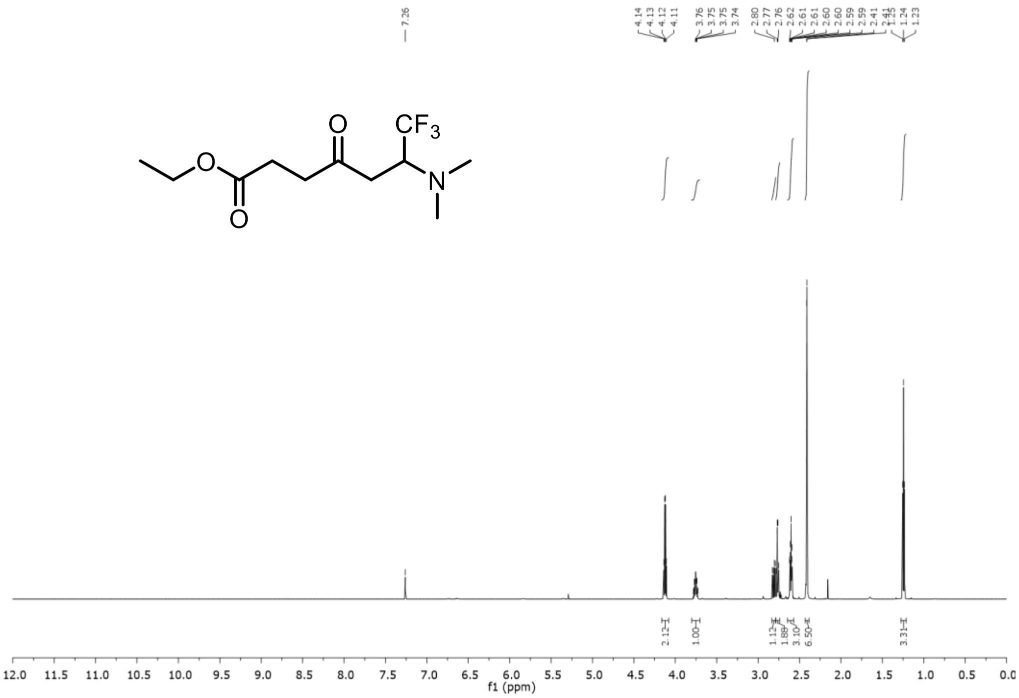

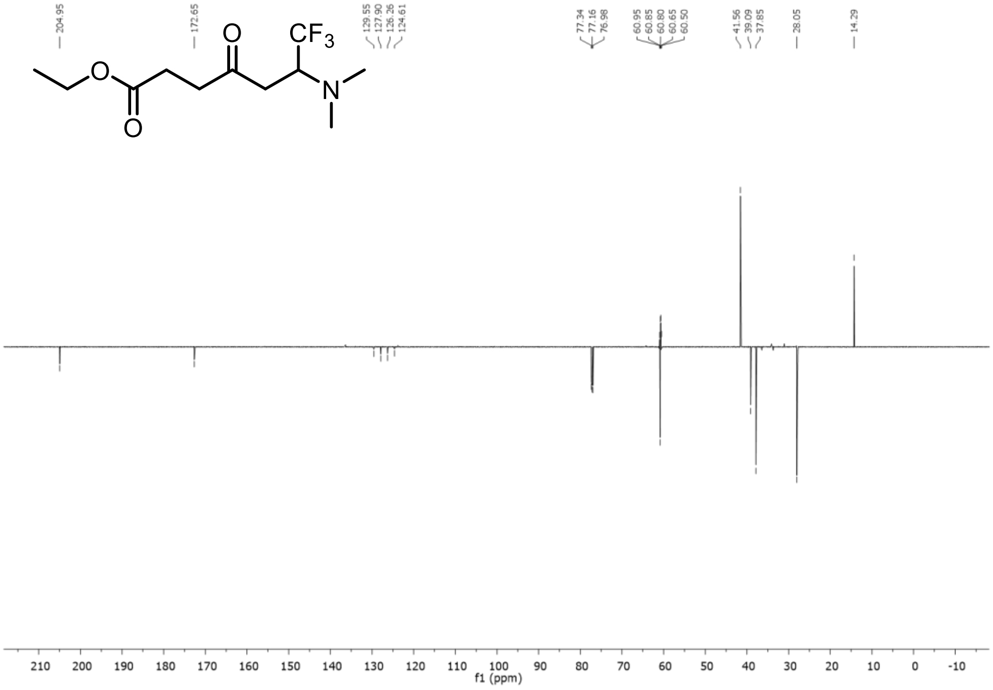

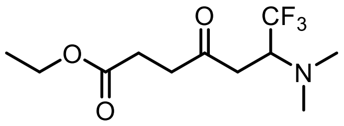

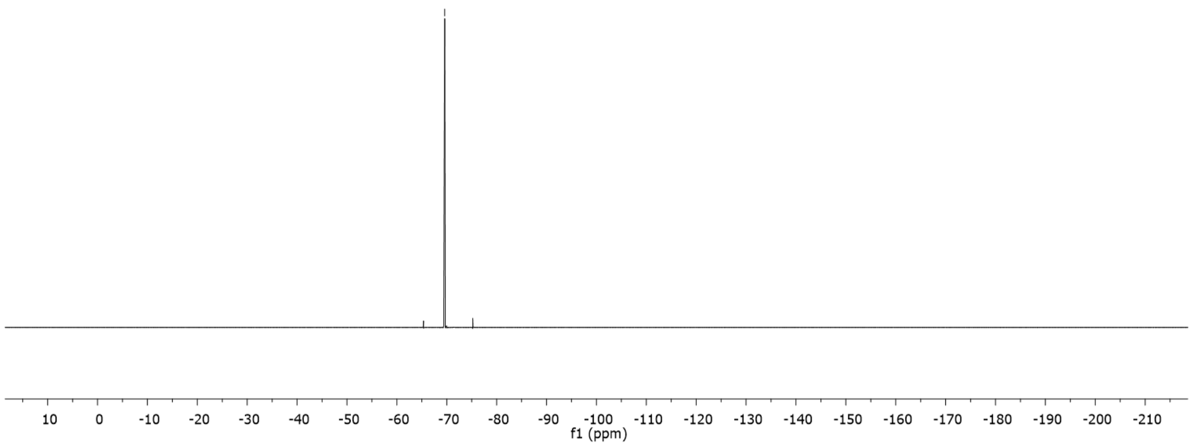
-69.60
10
0
-10
-20
-30
-40
-50
-60
-70
-80
-90
-100
-110
-120
-130
-140
-150
-160
-170
-180
-190
-200
-210
f1 (ppm)
